# Expanding the Repertoire of Photoswitchable Unnatural Amino Acids for Enzyme Engineering

**DOI:** 10.1002/anie.202508562

**Published:** 2025-08-04

**Authors:** Caroline Hiefinger, Michela Marcon, Verena Pape, Guillem Casadevall, Ranit Lahmy, Christoph Haag, Julian Nazet, Michael Bartl, Astrid Bruckmann, Sílvia Osuna, Burkhard König, Andrea Hupfeld

**Affiliations:** ^1^ Institute of Biophysics and Physical Biochemistry and Regensburg Center for Biochemistry University of Regensburg, Universitätsstraße 31 D‐93053 Regensburg Germany; ^2^ Institute of Organic Chemistry University of Regensburg, Universitätsstraße 31 D‐93053 Regensburg Germany; ^3^ Institut de Química Computacional i Catàlisi and Departament de Química Universitat de Girona c/ Maria Aurèlia Capmany 69 Girona 17003 Spain; ^4^ Institute of Biochemistry Genetics and Microbiology, University of Regensburg, Universitatsstrasse 31 D‐93053 Regensburg Germany; ^5^ ICREA, Pg. Lluís Companys 23 Barcelona 08010 Spain

**Keywords:** Diazo compounds, Enzyme catalysis, Photocontrol, Spiro compounds, Unnatural amino acids

## Abstract

Photoswitchable unnatural amino acids (psUAAs) play a crucial role in the engineering of light‐sensitivity in enzymes, which holds significant promise for diverse applications such as biotherapy and biocatalysis. Besides near‐quantitative photoconversion, the success and expediency of a psUAA for a certain application is defined by its interaction potential with the enzyme, its thermal stability and its effective wavelength of irradiation. To establish high versatility in the current repertoire, we have designed and synthesized six psUAAs based on azobenzene, arylazopyrazole, arylazothiazole, hemithioindigo and spiropyran photoswitches. The resulting psUAAs exhibit an enhanced interaction potential within an enzyme owing to their capacity for hydrogen bonding, ionic interactions and metal ion coordination. Moreover, we observed diverse photochemical behaviors among the psUAAs, with four of them reversibly switching between the isomers with purely visible light. Notably, we identified orthogonal aminoacyl‐tRNA synthetases that facilitate the incorporation of five of the six psUAAs co‐translationally and computationally analyzed the synthetase‐psUAA interactions. Finally, we evaluated the photochemical behavior of the five psUAAs within an enzymatic model and tested the photocontrol of catalysis confirming their diversity. Ultimately, our findings significantly expanded the repertoire of psUAAs and demonstrated their feasibility for enzyme engineering studies.

## Graphical abstract

Enzyme engineering with photoswitchable unnatural amino acids (psUAAs) has a high potential for applications such as biocatalysis. Here, we extend the current repertoire of psUAAs to include more versatile properties paving the way to more advanced photocontrol engineering. We report on the synthesis and photochemical behavior, suitable aminoacyl‐tRNA synthetases for co‐translational incorporation and initial enzymatic studies of these psUAAs.

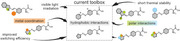

## Introduction

Reversible spatiotemporal control by light is gaining increasing significance in enzyme engineering applications.^[^ ^1^, ^2^, ^3^, ^4^ ^]^ In this regard, protein engineering with photoswitchable unnatural amino acids (psUAAs) facilitated by amber suppression using orthogonal aminoacyl‐tRNA synthetase (aaRS)/tRNA pairs, has emerged as a powerful method.^[^ ^5^, ^6^, ^7^ ^]^ Its key advantage is the genetic encoding of light‐sensitivity whilst minimally altering the enzyme via a single amino acid exchange. Generally, psUAAs comprise a synthetic photoswitch^[^ ^8^, ^9^, ^10^ ^]^ that can adopt two configurations, for example, *E* and *Z*, which are approximating 100% of the more stable configuration, mostly *E*, in thermal equilibrium (TEQ). Photocontrol is achieved by irradiation with a specific wavelength *λ* that shifts the equilibrium towards the less stable configuration, thereby establishing a so‐called photostationary state (PSS^λ^) with a defined isomer composition. Moreover, the higher the fraction of the less stable isomer at the PSS^λ^, the more quantitative is the photoconversion providing a solid basis for the photocontrol of enzymes. Ultimately, reversibility is facilitated by either the photoinduced reestablishment of a PSS^λ^ similar to TEQ or relaxation of the less stable isomer to the thermal equilibrium TEQ^post^.

In our previous studies, we could successfully demonstrate efficient reversible photocontrol of catalysis of up to ∼10‐fold with a psUAA in two independent enzymatic systems.^[^ ^11^, ^12^, ^13^ ^]^ In both enzymes, the psUAA was positioned outside the active site but close to catalytically relevant conformational changes. Further investigations revealed that the light‐induced configurational switch of the psUAA induced a conformational shift at the active sites of both enzymes. Consequently, psUAAs appear to be highly effective when they are used as light‐induced allosteric switches. Thus, besides the efficiency of the photoswitch, the successful engineering of photocontrol in enzymes requires a well‐defined communication between the incorporated psUAA and the orthosteric site leading to functional differences initiated by the photoinduced configurational changes.^[^ ^14^ ^]^ This communication is determined by both the position of incorporation and the interaction potential of the psUAA with the adjacent enzyme environment. Considering the vast properties of enzyme targets and incorporation sites, the availability of a broad selection of unpolar and polar interactions would significantly increase the success rate of photocontrol engineering with psUAAs. Moreover, such interactions might extend the functional range of the psUAA beyond the use as allosteric switch, if the respective substituents may allow to directly take part in catalysis.

Furthermore, the diversity of applications for photocontrolled enzymes including biotherapeutic strategies and industrial biocatalysis necessitates a repertoire of psUAAs that is adapted to the specific demands of each approach. This entails psUAAs, which i) either maintain their configuration or thermally relax to their initial state after irradiation stops, and ii) respond to various wavelengths including visible light, which confers reduced toxicity and higher penetration depths.

**Figure 1:**
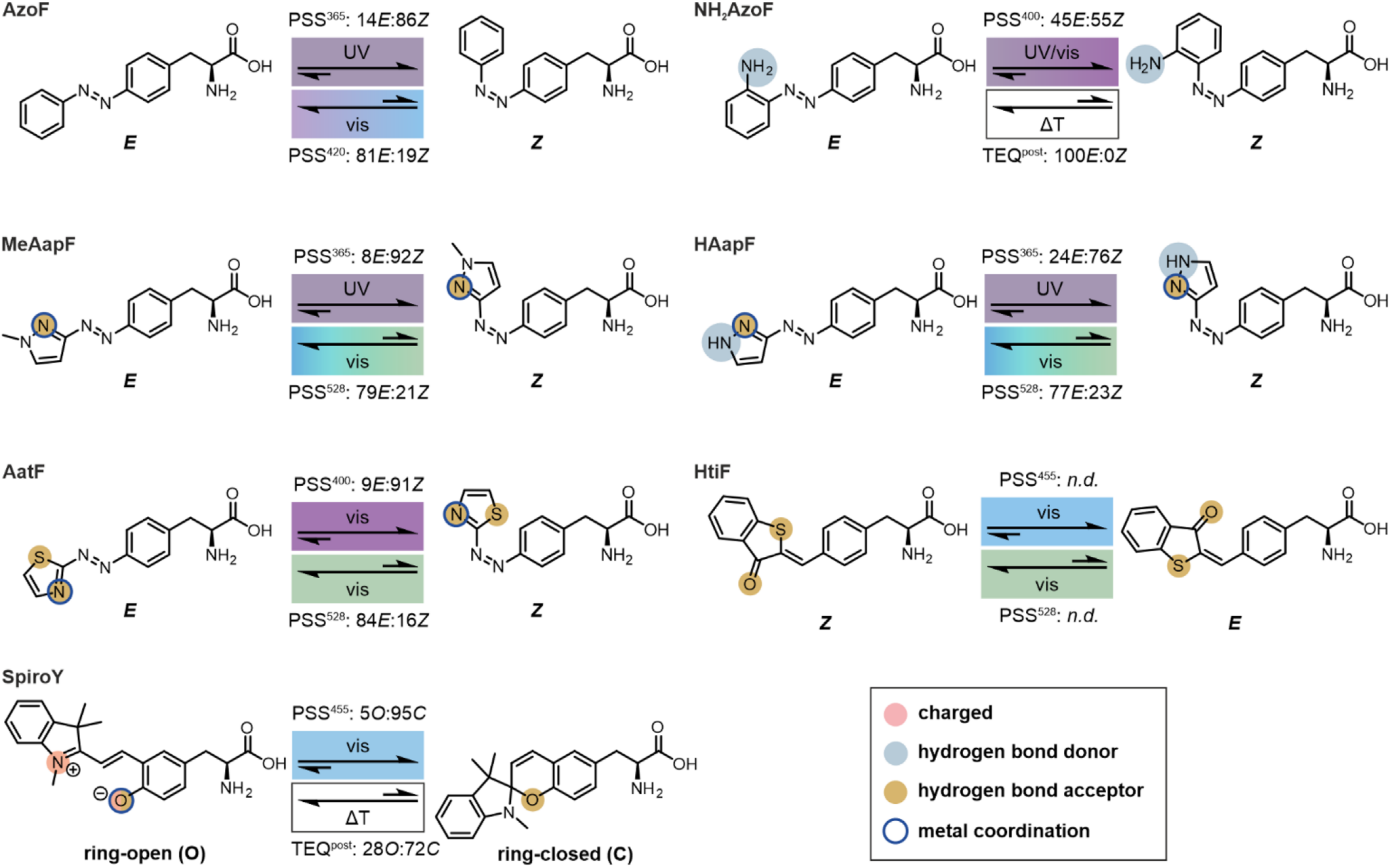
psUAA design and summary of photochemical properties. Photoisomerization upon irradiation with a specific wavelength *λ* in the UV (dirty violet) or visible (violet to green) range, or thermal relaxation (ΔT, white box) induce the formation of either a PSS^λ^ or a TEQ^post^, respectively. Each state is characterized by a certain isomer distribution as determined by HPLC or UV/Vis. Colored circles indicate functional groups for potential polar interactions.

Notably, the current repertoire of psUAAs (Figure S1)^[^ ^15^ ^]^ only covers limited diversity in photoswitch efficiencies, interaction potentials, thermal stabilities and effective wavelengths of irradiation. Most of these psUAAs comprise an azobenzene photoswitch, starting with unsubstituted phenylalanine‐4′‐azobenzene (**AzoF**; Figure 1).^[^ ^16^, ^17^, ^18^ ^]^ Various substitutions allowed for crosslinking **AzoF** with cysteins in a protein via click chemistry,^[^ ^19^, ^20^ ^]^ and for switching using exclusively visible light.^[^ ^21^, ^22^ ^]^ However, these changes came in part at the cost of less quantitative photoconversions. A wider range of diversity can be explored by using different scaffolds from the vast variety of existing photoswitches.^[^ ^8^, ^9^ ^]^ Most recently, two diazocine‐based psUAAs were introduced, which similarly reacted solely to visible light but lost switching efficiency.^[^ ^23^, ^24^ ^]^ Two further studies reported the synthesis and incorporation of an arylazopyrazole‐based psUAA (AapF) with improved quantitative photoconversions but a similar wavelength profile compared to **AzoF**.^[^ ^25^, ^26^ ^]^ While some psUAAs from this current repertoire exhibit hydrogen bond acceptors in addition to the predominantly hydrophobic photoswitch scaffold, hydrogen bond donors or charged groups are hitherto missing. Moreover, all of them display high thermal stabilities necessitating a second irradiation step to achieve reversibility of photocontrol.

In this study, we set out to improve the versatility of the psUAA repertoire for the recombinant production of photocontrolled enzymes using azobenzene, arylazopyrazole, arylazothiazole, hemithioindigo and spiropyran photoswitch scaffolds. We describe the synthesis of six psUAAs, which i) showed improved photoswitch efficiencies, ii) mostly contain hydrogen bond acceptors, donors and/or charged groups, or iii) exhibit variation in thermal stabilities as well as effective wavelengths of irradiation. Remarkably, we were able to identify suitable aaRS/tRNA pairs facilitating the incorporation of five of the six psUAAs into a model enzyme. Photochemical and kinetic characterization of the resulting enzyme variants demonstrated different behavior and photocontrol potential of each psUAA.

## Results and Discussion

### Design and Synthesis

To obtain the intended psUAA diversity regarding photoswitch efficiencies, interaction potentials, thermal stabilities and effective wavelengths of irradiation in our design, we decided to exploit the current variety of photoswitch scaffolds. As a first step, we considered an **AzoF** derivative, **NH_2_AzoF**, that is different from previous designs (Figure S1) due to the presence of a primary amine that acts as a hydrogen bond donor (blue sphere, Figure 1) and nucleophile. *Ortho*‐substitution of azobenzenes with such an electron‐donating group has been shown to considerably increase thermal relaxation of the *Z* isomer and red‐shift the effective wavelength.^[^ ^27^ ^]^ Moreover, similar to the previously reported AapF,^[^ ^25^, ^26^ ^]^ other arylazopyrazole photoswitches displayed equally high photoconversion yields.^[^ ^28^, ^29^ ^]^ Hence, we designed **MeAapF** as a less sterically demanding version of AapF with a retained imine in the pyrazole ring as hydrogen bond acceptor (gold sphere, Figure 1). While exchanging a functional group in the heteroarene ring may significantly alter the photochemical properties of arylazopyrazoles,^[^ ^28^ ^]^ the introduction of a secondary, free amine enhances the interaction potential by serving as hydrogen bond donor or nucleophile. For this reason, we included **HAapF** in our design, which contains such a secondary amine in addition to the imine in the pyrazole ring (blue and gold sphere, Figure 1). In general, arylazopyrazoles as well as the structurally related arylazothiazoles are known to coordinate metal ions via their imine (blue circles, Figure 1) in the heteroaromatic moiety,^[^ ^30^, ^31^, ^32^ ^]^ which is appealing for metalloenzymes. Furthermore, the latter have recently emerged as attractive scaffolds with fast thermal relaxation of the *Z* isomer and irradiation wavelengths in the visible range.^[^ ^33^ ^]^ Hence, we tried to capture these properties in the design of **AatF**, which comprises a thioether that acts as additional hydrogen bond acceptor to the imine of the thiazole ring (gold spheres, Figure 1). Furthermore, we included photoswitches, which are based on other isomerization mechanisms than diazo compounds. To this end, we selected **HtiF** that has previously been incorporated into an ion channel via chemical acylation, but has so far not been photochemically characterized or incorporated via amber suppression.^[^ ^34^ ^]^ The olefinic bond of this hemithioindigo‐based psUAA confers the ability to switch between *E* and *Z* isomers with visible light bidirectionally. **HtiF** is not only capable of interacting with other amino acids in the protein environment through π‐stacking interactions with its conjugated system similar to **AzoF**, **NH_2_AzoF**, **MeAapF**, **HAapF** and **AatF**, but may also participate in hydrogen bonding with its carbonyl and thioether groups (gold spheres, Figure 1).^[^ ^35^ ^]^ Finally, we included a psUAA that is able to undergo an electronical rearrangement resulting in the interconversion between an open (*O*) and a closed (*C*) state. To simultaneously increase the interaction potential, we chose **SpiroY**, which isomerizes between a flexible, charged merocyanine (*O*) and a rigid, neutral spiropyran (*C*). The open isomer can participate in ionic interactions with its positively charged iminium ion and its negatively charged phenolate (pink spheres, Figure 1), which can further serve as hydrogen bond acceptor (gold sphere), Brønsted base or complexation site for metal ions (blue circle).^[^ ^36^, ^37^ ^]^ In contrast, the closed form is largely inert with only an ether group as potential hydrogen bond acceptor (gold sphere).

**Figure 2:**
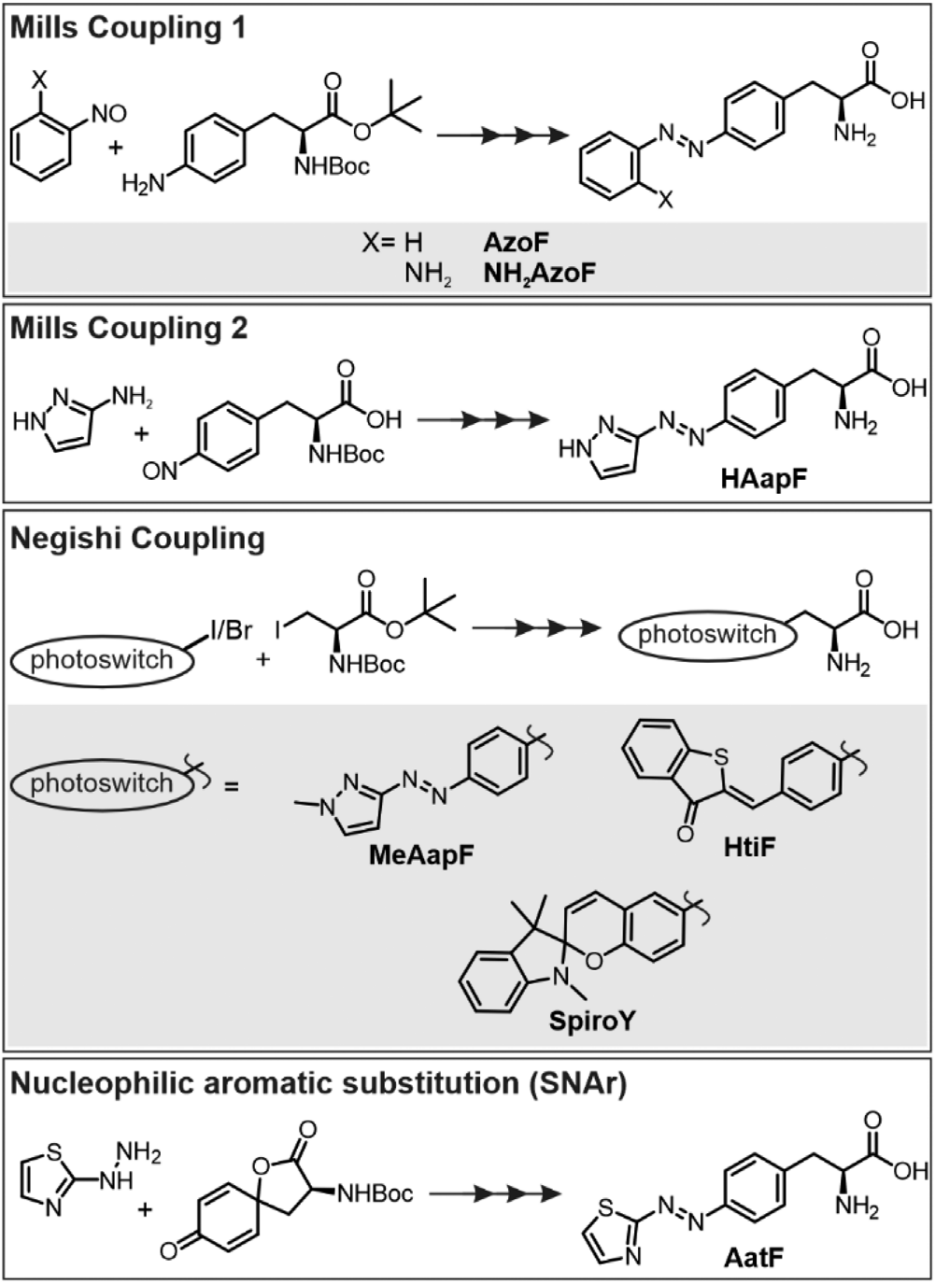
Schematic overview of the four main synthetic routes employed for the synthesis of psUAAs based on azobenzene, arylazopyrazole, arylazothiazole, hemithioindigo and spiropyran photoswitches. Boc, tert‐butyloxycarbonyl.

Next, we synthesized the six psUAAs and **AzoF**, which we used for comparison in the subsequent characterization. To this end, four main synthetic routes have been established (Figure 2): Mills Coupling 1 of protected 4‐amino‐l‐phenylalanine to nitroso derivatives (**AzoF**, **NH_2_AzoF**), Mills Coupling 2 of 3‐aminopyrazole to protected 4‐nitroso‐l‐phenylalanine (**HAapF**),^[^ ^38^, ^39^, ^40^ ^]^ Negishi Coupling of the iodo‐ or bromo‐ derivatives of the photoswitches to protected iodo‐l‐alanine (**MeAapF**, **HtiF**, **SpiroY**)^[^ ^41^ ^]^ and nucleophilic substitution of 2‐hydrazineylthiazole to the oxaspiro[4,5]decadien derivative (**AatF**). A detailed description of each synthesis can be found in the Supporting Information (Schemes S1–S6). After flash chromatography purification, the psUAAs were isolated as colored solids with overall yields of 3%–74%.

### Photochemical Characterization

After successful synthesis, we performed an extensive photochemical characterization of each psUAA. Because psUAAs generally show poor water solubility but need to comply with aqueous conditions used for biological applications, we compared the properties in aqueous solution (50 mM Tris/HCl pH 7.5) and solvent (DMSO). Notably, most of the psUAAs demonstrated similar or even better water solubility than **AzoF**. Only **HtiF** demonstrated low solubility in buffer as expected from recent observations of high aggregation tendencies of hemithioindigos in the absence of organic (co)solvents.^[^ ^42^ ^]^ In all cases, aggregate formation could be prevented in photochemical measurements by adjusting the compound concentration and we only point out exceptions thereof in the following description.

**Figure 3:**
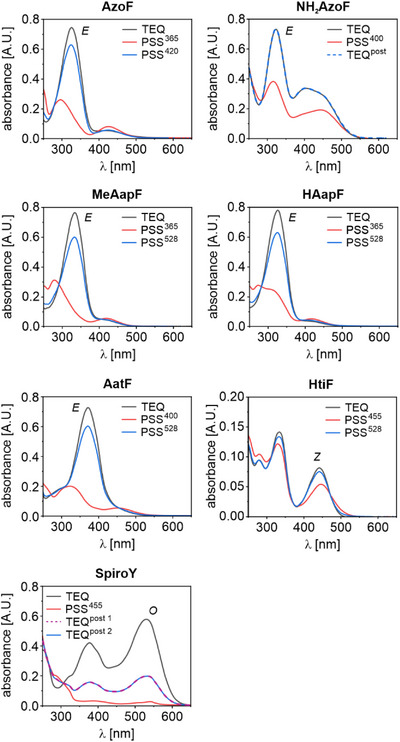
Absorbance spectra of the psUAAs in buffer. Real‐time tracking of the absorbance signal assured the complete establishment of the respective PSS^λ^ or TEQ^post^ prior to spectrum acquisition (Table S2 and Figure S6). The most abundant isomer in the TEQ is indicated next to the grey spectrum.

As part of the photochemical characterization, we acquired absorbance spectra of the TEQs, compared various PSS^λ^ spectra, determined the isomer distributions at the most relevant PSS^λ^, measured the half‐lives of thermal relaxation and evaluated the photostabilities over various switching cycles. While we have focused on a comprehensive comparison of the six psUAAs and their properties in the following, we have also provided a more detailed analysis of the structure‐activity relationship of each psUAA in the Supporting Information (Extended Texts S1–S6). We started our analysis by recording absorbance spectra of the TEQs (Figures 3, S2) and identifying typical maxima for each photoswitch (Table S1). The TEQ spectra of **AzoF**, **NH_2_AzoF**, **MeAapF** and **HAapF** were characteristic of the respective *E* isomers^[^ ^12^, ^18^, ^28^, ^29^ ^]^ revealing pronounced π→π* transitions in the range of 290 nm to 335 nm and smaller n→π* transitions at 400–490 nm. Remarkably, the n→π* transition of **NH_2_AzoF** was broadened, which opens the possibility of red‐shifted effective wavelengths for *E*→*Z* isomerization. While spectra in DMSO were similar to those in buffer for **AzoF**, **MeAapF** and **HAapF**, we observed a slight shift of the n→π* transition towards higher wavelengths for **NH_2_AzoF**. As reported for arylazothiazoles,^[^ ^33^ ^]^ **AatF** exhibited one major band in both buffer and DMSO for the π→π* transition at ∼370 nm indicative of the *E* isomer. Similar to **NH_2_AzoF**, the pronounced red‐shift of this band promises effective isomerization wavelengths in the visible range. The absorbance spectra of the thermally stable *Z* isomer of **HtiF** in both buffer and DMSO demonstrated two absorbance maxima at ∼330  and ∼440 nm, which is in agreement with reported hemithioindigo spectra.^[^ ^43^ ^]^ Interestingly, we observed a special scenario for **SpiroY**. Previous studies have shown that spiropyrans are stabilized in their positively charged *O* isomer by high salt concentrations in organic solvents.^[^ ^44^ ^]^ Accordingly, the TEQ absorbance spectrum of **SpiroY**, recorded directly after dilution into buffer from the salt‐enriched DMSO stock solution, exhibited absorbance characteristics of the conjugated *O* isomer. However, the absorbance signal decreased towards a new TEQ^post1^ with *t_½_^post1^*  =  1.9 min (Figure S3a) indicating the deprotonation of *O* due to the lower salt concentration and thus isomerization to the colorless *C* isomer. We observed similar, however, significantly slower (*t_½_^post1^*  =  17.3 h) spectral changes upon dilution in DMSO (Figure S3b).

To initiate the photoinduced isomerization of each psUAA, we tested different wavelengths in the range of 365  and 567 nm (Figures S4, S5) but excluded wavelengths <365 nm owing to their ineptitude for biological applications. We thereby monitored the absorbance at the defined maximum during irradiation until each PSS^λ^ was fully established (Tables S2, S3, Figures S6, S7) and then recorded spectra. In accordance with the spectral properties evident from the TEQ spectra, the most effective irradiation wavelengths towards a PSS^λ^ enriched in the thermally less stable isomer were in the UV range for **AzoF**, **MeAapF** and **HAapF**, and in the near‐visible to green range for **NH_2_AzoF**, **AatF**, **HtiF** and **SpiroY** (Figure 1). Instead, photoinduced reestablishment of a PSS^λ^ similar to TEQ was accomplished exclusively with visible light for all psUAAs except **NH_2_AzoF** and **SpiroY**. However, both psUAAs exhibited a thermally unstable *Z* and *C* isomer, which allowed them to relax spontaneously to TEQ^post^ and TEQ^post2^, which are equivalent to TEQ and TEQ^post1^, respectively. For both isomerization processes, the most effective wavelengths in buffer (Figure 3) and DMSO (Figure S2) were either the same or similar for each of the six psUAAs.

We then evaluated the thermal relaxation of the less stable isomers for each psUAA by determining the thermal half‐lives *t_½_* (ΔT) in buffer at 25 °C (Table 1). While **AzoF**, **MeAapF**, **HAapF** and **AatF** offer bistability with *t_½_* (ΔT) values between hours to several days, **NH_2_AzoF**, **HtiF** and **SpiroY** showed rapid full reversibility within <20 min (Figures S8, S9).

**Table 1:** Half‐lives *t_½_* (ΔT) of thermal relaxation for each psUAA in buffer at 25 °C.

psUAA	*t_½_* (ΔT)^a)^	Unit
**AzoF**	16.4 ± 0.40^b)^	d
**NH_2_AzoF**	9.91 ± 0.02^c)^	s
**MeAapF**	45.6 ± 1.70^b)^	d
**HAapF**	10.3 ± 0.20^b)^	d
**AatF**	1.80 ± 0.004^c)^	h
**HtiF**	2.04 ± 0.003^c)^	min
**SpiroY**	1.90 ± 0.0001^c)^	min

^a)^ Calculated from the rates *k* (Figure S8) of thermal relaxation from a PSS^λ^ enriched in the thermally less stable isomer to the TEQ^post^.

^b)^ Measured via HPLC.

^c)^ Measured via UV/Vis spectroscopy.

Next, we assessed the quantity of photoconversion by separating the isomers via HPLC following the absorbance at specific isosbestic points (Table S4, Figures S10, S11). As a result, we obtained isomer distributions for TEQ, PSS^λ^ and TEQ^post^ in buffer (Figure 1, Table S5) and in DMSO (Table S6). In buffer, all psUAAs except **AzoF** (97*E*:3*Z*) and **SpiroY** (93*O*:7*C*) revealed 100% of the thermodynamically more stable isomer in TEQ. The latter can be explained by the above‐described establishment of TEQ^post1^ (28*O*:72*C*) after dilution. We first evaluated the photoinduced switch of the bistable psUAAs, obtaining isomer distributions of 14*E*:86*Z* for **AzoF** (PSS^365^), 8*E*:92*Z* for **MeAapF** (PSS^365^), 24*E*:76*Z* for **HAapF** (PSS^365^) and 9*E*:91*Z* for **AatF** (PSS^400^). Moreover, the photoinduced backswitch established 81*E*:19*Z* for **AzoF** (PSS^420^), 79*E*:21*Z* for **MeAapF** (PSS^528^), 77*E*:23*Z* for **HAapF** (PSS^528^) and 84*E*:16*Z* for**AatF** (PSS^528^). Conclusively, **MeAapF**, and **AatF** achieved nearly quantitative switching in the *E*→*Z* isomerization and mediocre to good photoconversion in the *Z*→*E* isomerization, outperforming **AzoF** and **HAapF**. HPLC analysis of **NH_2_AzoF** and **HtiF** was unsuccessful owing to the extremely short *t_½_* (ΔT) and solubility issues,^[^ ^42^ ^]^ respectively. **SpiroY** demonstrated a nearly quantitative photoinduced switch to 5*O*:95*C* (PSS^455^) and a thermal relaxation to 28*O*:72*C* (TEQ^post2^) indicating a complete reestablishment of TEQ^post1^. In DMSO, isomer distributions of all psUAAs coincided with those in buffer with slight deviations for **SpiroY,** which has been reported to be sensitive to solvent changes.^[^ ^44^, ^45^ ^]^ While the measurements for **NH_2_AzoF** were again inconclusive, **HtiF** showed isomer distributions of 77*E*:23*Z* (PSS^420^) and 2*E*:98*Z* (PSS^505^).

Although HPLC provides an exact method for the determination of isomer distributions, it is not applicable for the evaluation of psUAAs incorporated into a protein. Hence, we further examined the isomer ratios using UV/Vis spectra, an approach that is commonly used for diazo photoswitches.^[^ ^28^ ^]^ Overall, the estimated values were in good agreement with the HPLC results (Tables S5, S6). In this way, we were also able to yield an isomer distribution upon irradiation in buffer for **NH_2_AzoF** of 45*E*:55*Z* (PSS^400^) and fully recover 100*E*:0*Z* in TEQ^post^. It should be noted that the fast thermal relaxation of **NH_2_AzoF** counters its photoinduced isomerization. As a result, the isomer distribution depends on the intensity of irradiation as is observed for photoreceptors used in optogenetics,^[^ ^46^ ^]^ opening up the possibility to further tune the PSS^λ^ composition. While there are currently no UV/Vis‐based methods to estimate the isomer ratio for **HtiF**, we obtained similar values for **SpiroY** compared to the HPLC evaluation by exploiting the lack of an absorbance signal in *C* using the rule of three.

Finally, we assessed the photostability of each psUAA over ten switching cycles (Figures S12, S13). Except **HtiF**, which indicated slight photobleaching in buffer, all psUAAs maintained high photostability without fatigue in both buffer and DMSO.

In conclusion, we developed a repertoire of psUAAs with high diversity (Figure 1). Moreover, the psUAAs demonstrated the anticipated photochemical properties that are typical for each photoswitch in both aqueous solution and solvent,^[^ ^28^, ^33^, ^43^, ^47^, ^48^, ^49^ ^]^ which constitutes beneficial knowledge for the rational chemical design of psUAAs.

### Screening of Aminoacyl‐tRNA Synthetases (aaRSs) for Selective Incorporation of each psUAA

We further explored the incorporation of the psUAAs during recombinant protein production via amber suppression. To this end, we have screened a selection of aaRSs for their ability to efficiently aminoacylate the orthogonal tRNA with each synthesized psUAA. All chosen aaRSs were based on the tyrosyl‐tRNA synthetase from *Methanocaldococcus jannaschii* (*Mj*Tyr‐RS). Although *Mj*Tyr‐RSs are only orthogonal in prokaryotic cells, they are more adapted to binding aromatic amino acids and have previously achieved exceptionally high incorporation efficiencies.^[^ ^50^, ^51^, ^52^, ^53^ ^]^ Accordingly, for the incorporation of our aromatic psUAAs, we used established *Mj*Tyr‐RS variants that contain amino acid exchanges at defined positions (Y32, L65, E107, F108, Q109, Q155, D158, I159, L162, A167, V188, R257 and D286).^[^ ^18^, ^26^, ^50^, ^54^ ^]^ Moreover, we performed a computational design based on the original AzoF‐RS^[^ ^18^ ^]^ (aaRS‐a), from which we selected further variants. The resulting library contained 22 *Mj*Tyr‐RSs (a‐v; Tables S7,S8).

**Figure 4:**
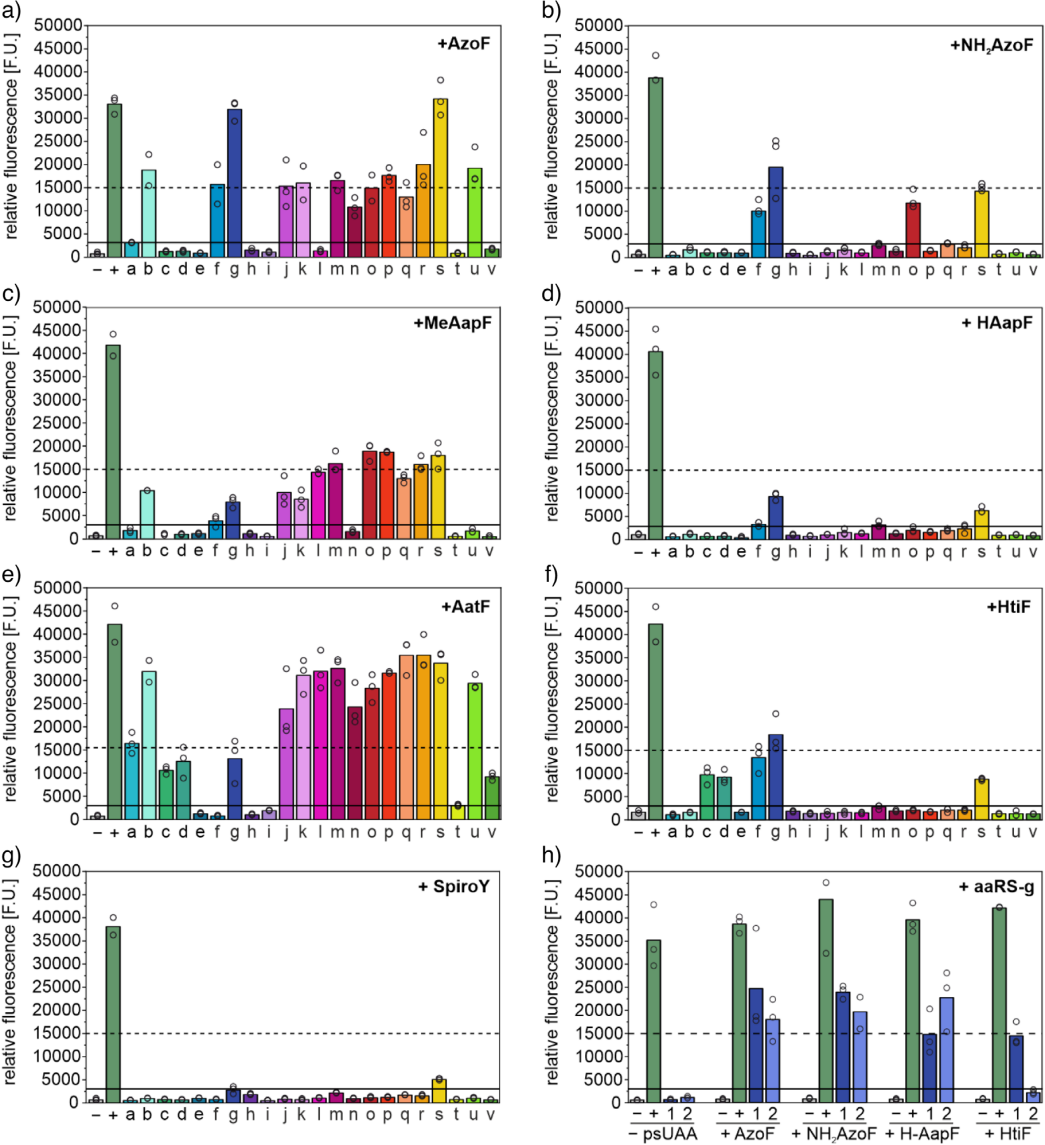
Screening of *Mj*Tyr‐RS variants (a‐v) using pGLNS for the orthogonal incorporation of psUAAs in sfGFP_Y151TAG using pETBAD_sfGFP_Y151TAG (a–g) and influence of aaRS copy number (h). Empty circles: fluorescence intensity of three biological replicates; columns: mean fluorescence intensity; “−”: pGLNS without aaRS; “+”: wild‐type sfGFP; 1: pGLNS_aaRS‐g; 2: pEVOL_aaRS‐g.

Screening of the synthetases was accomplished using two plasmids: i) pETBAD encoding the reporter sfGFP with an amber stop codon (TAG) at position Y151 (pETBAD_sfGFP_Y151TAG), and ii) pGLNS harboring an aaRS variant and the respective tRNA. We initially optimized the screening procedure using **AzoF** (Figure S14), which resulted in low background signals when pGLNS without aaRS was employed as negative control (−; Figure 4a). sfGFP expression in the presence of **AzoF** was examined by measuring fluorescence intensities for each culture containing aaRSs a–v in biological triplicates. Interestingly, aaRS‐a yielded relatively low intensities, probably due to the use of only one gene copy compared to the commonly used two copies in the pEVOL plasmid system.^[^ ^55^ ^]^ Since pEVOL_aaRS‐a usually facilitates reasonable incorporation efficiencies in large scale,^[^ ^12^, ^13^ ^]^ we set the respective fluorescence value as lower threshold (solid line) for the identification of suitable aaRSs for the other psUAAs.

Moreover, we defined a second threshold (dashed line) at 30% of the wild‐type sfGFP signal of the positive control (+) to determine aaRSs with high incorporation efficiencies. Importantly, we excluded misincorporation of natural amino acids for each aaRS, except aaRS‐s, by performing the screening experiment in the absence of psUAAs (Figure S15).

To our astonishment, the codon‐optimized variant aaRS‐b harboring the additional R257G mutation significantly outperformed aaRS‐a regarding **AzoF** incorporation, and expression was even further enhanced with aaRS‐g, another previous AzoF‐RS design (Figure 4a).^[^ ^50^ ^]^ Remarkably, we observed fluorescence intensities above the second threshold for **NH_2_AzoF**, **MeAapF**, **AatF** and **HtiF** with the highest signals obtained using aaRS‐g, aaRS‐p, aaRS‐q and aaRS‐g, respectively (Figure 4b–g). While aaRS‐g yielded fluorescence intensities in the presence of **HAapF** between both thresholds, no aaRS could be identified for the incorporation of **SpiroY**. **SpiroY**‐*C* is structurally rigid and bulky, which may significantly hinder binding to an aaRS. Moreover, the charged state of **SpiroY**‐*O*, which is also present in large amounts within the expression host (cf. TEQ^post1^), may interfere with the hydrophobic binding pocket of the *Mj*Tyr‐RSs. Hence, we anticipate that successful incorporation of **SpiroY** will require more extensive engineering efforts of aaRSs.

To further boost the incorporation efficiency of **HAapF**, we compared sfGFP expression yields obtained using either one copy of aaRS‐g in pGLNS or two copies in pEVOL (Figure 4h).^[^ ^55^ ^]^ Again, misincorporation of natural amino acids in the absence of psUAA could be excluded for both expression systems. Interestingly, the new biological replicates demonstrated fluorescence intensities approximating the second threshold in the presence of pGLNS_aaRS‐g, and even surpassed it with pEVOL_aaRS‐g. We also tested the pEVOL system for **AzoF**, **NH_2_AzoF** and **HtiF**, which showed the highest incorporation yields with aaRS‐g. However, the fluorescence and conclusively, the expression level remained similar or decreased with pEVOL_aaRS‐g compared to pGLNS_aaRS‐g. The overall observed differences and fluctuations in expression levels might be associated with off‐target interactions of the psUAAs and aaRSs with host cell components or with perturbations in energy metabolism.^[^ ^56^, ^57^ ^]^ Previous studies have particularly shown that overexpression of both an aaRS and a gene of interest can deplete translational factors and metabolites, especially if the aaRS/tRNA pair is highly efficient.^[^ ^58^ ^]^

**Figure 5:**
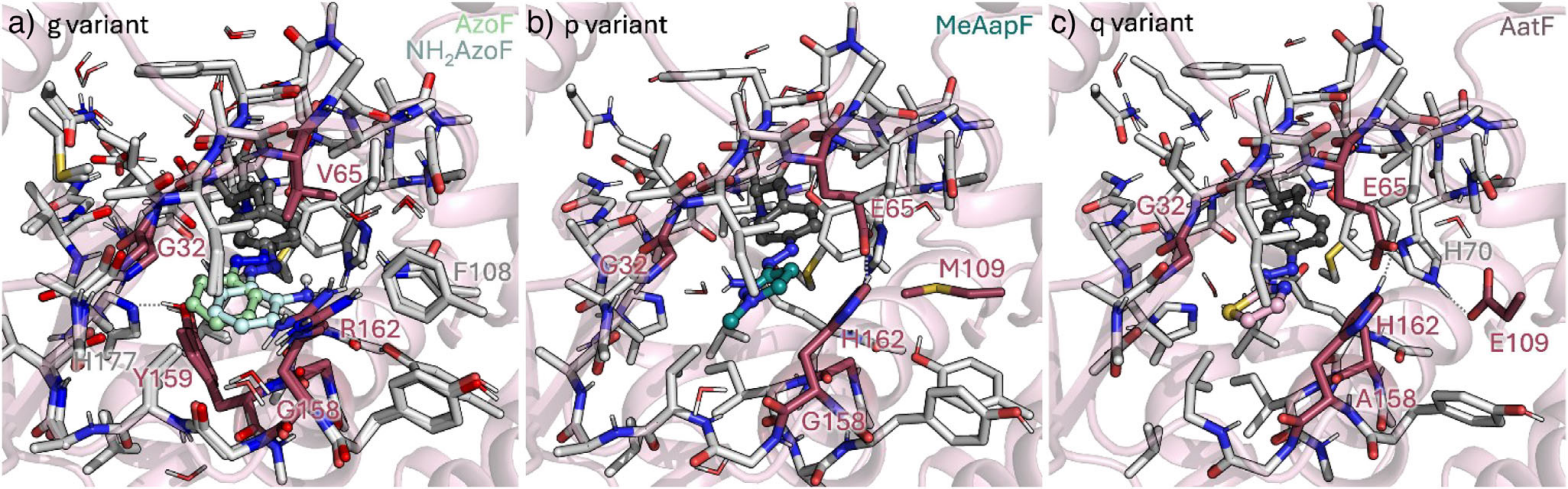
Optimized cluster models of *Mj*Tyr‐RS variants aaRS‐g, ‐p and ‐q. a) Overlay of the optimized structures of aaRS‐g containing exemplarily either **AzoF** (shown in dark grey and green spheres) or **NH_2_AzoF** (in grey and cyan). b) Optimized structure of the aaRS‐p containing **MeAapF** bound in the pocket. c) Optimized structure of aaRS‐q containing **AatF** bound in the pocket. All mutations are colored in raspberry.

Finally, we were interested in the binding mode of **AzoF**, **NH_2_AzoF**, **MeAapF**, **HAapF**, **AatF** and **HtiF** with their respective aaRSs. To that end, we constructed a cluster model of the following combination of *Mj*Tyr‐RS variants and psUAAs: **AzoF**, **NH_2_AzoF**, **HAapF**, **HtiF** in aaRS‐g, **MeAapF** in aaRS‐p and **AatF** in aaRS‐q (Table S9). The studied *Mj*Tyr‐RS variants contain mutations mostly in the psUAA‐binding region. In all three aaRS‐g, ‐p and ‐q, mutation Y32G is introduced, which replaces the sterically bulky Y32 for a substantially smaller and more flexible glycine (Figure 5). This mutation is clearly needed to generate additional space to allow for binding of all psUAAs. In aaRS‐g, the space left by Y32G substitution can be potentially occupied by the new arginine residue included at position 162 (L162R, Figure S16). However, for **AzoF**, **NH_2_AzoF**, **HtiF** and **HAapF** binding, R162 needs to be displaced from the pocket (Figure 5a). It should be noted that such conformation of R162 has also been reported in previous X‐ray structures (PDB: 6WRT) and is also predicted by AlphaFold2 (Figure S16). Both aaRS‐p and ‐q present a histidine residue at position 162 instead (L162H), which establishes a hydrogen bond with the new glutamate residue included at position 65 (L65E). This hydrogen bond is also maintained when **AatF** and **MeAapF** are bound in the pocket (Figure 5b,c). In aaRS‐g position 65 contains instead a valine (L65V) to allow enough space for **AzoF**, **NH_2_AzoF**, **HtiF**, and **HAapF** binding (Figure S17). Another important mutation for providing additional space is D158 to either glycine (in aaRS‐g and ‐p) or alanine (in aaRS‐q). Only in aaRS‐g a tyrosine residue is introduced at position 159 (i.e., I159Y), which establishes a hydrogen bond with H177 and hydrophobic interactions with the aromatic rings of the different psUAAs (Figure 5a). In aaRS‐p and ‐q, two additional mutations are included: the bulky F108 is replaced by a smaller alanine (F108A), and Q109 is replaced by either a methionine (Q109M) or glutamate (Q109E), respectively. Our calculations indicated that E109 establishes a hydrogen bond with H70, altering the sidechain conformation of H70 and thus, providing additional space for efficient **AatF** binding.

Ultimately, our findings make it possible to effectively use **NH_2_AzoF**, **MeAapF**, **HAapF**, **AatF** and **HtiF** for the recombinant production of potentially light‐sensitive enzymes in prokaryotes. For studies in eukaryotic cells, that are beyond the scope of this work, we recommend to test orthogonal aaRSs based on pyrrolysyl‐tRNA synthetases, which have been evolved for incorporation of **AzoF** derivatives,^[^ ^20^, ^21^, ^22^ ^]^ or tyrosyl‐tRNA synthetases from *Geobacillus stearothermophilus*.^[^ ^59^ ^]^

### Incorporation into an Enzyme and Characterization

The identification of aaRSs for the co‐translational incorporation allowed us to further characterize **NH_2_AzoF**, **MeAapF**, **HAapF**, **AatF** and **HtiF** in comparison to **AzoF** within an enzymatic environment. For this purpose, we selected HisF from *Thermotoga maritima*, which catalyzes the reaction of *N'*‐[(5′‐phosphoribulosyl)formimino]‐5‐amino‐imidazole‐4‐carboxamide ribonucleotide (PrFAR) and NH_3_ to imidazole glycerol phosphate and 5‐aminoimidazol‐4‐carboxamidribotide (Figure S18), because of several advantageous aspects. Its heat‐stability allows for a highly efficient purification by performing a heat‐step and more importantly, the catalyzed reaction can be monitored directly by following the decline in absorbance of PrFAR. Moreover, hitherto unpublished data of HisF variants containing **AzoF** at positions close to a catalytically essential induced fit closure of an active site flexible loop^[^ ^60^ ^]^ was available from our previous project.^[^ ^13^ ^]^ This data provided a comparison of expression yields and activities before and after irradiation for each HisF‐AzoF variant and demonstrated that wild type HisF (HisF‐wt) is largely unaffected by irradiation (Figure S19). For the incorporation of our novel psUAAs, we aimed for a position, in which **AzoF** incorporation facilitated >50% of wildtype activity, and potentially a slight activation of catalysis upon irradiation. Only two positions, K13 and L35, met these criteria, of which we chose K13 owing to apparently higher expression yields.

Using the corresponding aaRS identified in the screening, we produced HisF variants containing either **AzoF**, **NH_2_AzoF**, **MeAapF**, **HAapF**, **AatF** or **HtiF** at position K13, delineated as “K13psUAA”. The enzymes were derived in moderate to high yields and in moderate to excellent purities (Table 2; Figure S20). It should be noted that differences in yield may not only result from distinct efficiencies of the respective aaRSs, but also from the compatibility between the psUAA and the site of incorporation.^[^ ^61^, ^62^ ^]^ Tryptic digest coupled to mass spectrometry confirmed the identity of each K13psUAA variant (Figure S21).

**Table 2:** Properties of HisF variants containing psUAAs at position K13 and L35.

property	K13AzoF	K13NH_2_AzoF	K13MeAapF	K13HAapF	K13AatF	K13HtiF	L35HtiF
Expression yields	29 mg L^−1^ ^a)^	8 mg L^−1^	3 mg L^−1^	35 mg L^−1^	66 mg L^−1^	6 mg L^−1^	14 mg L^−1^
Purity	>95%	>90%	>70%	>90%	>95%	>50%	>95%
*E*→*Z* isomerization	PSS^365^: 13*E*:87*Z*	PSS^400^: 73*E*:27*Z* ^c)^	PSS^365^: 34*E*:66*Z*	PSS^365^: 16*E*:84*Z*	PSS^400^: 10*E*:90*Z*	—^d)^	—^d)^
*Z*→*E* isomerization	PSS^420^: 82*E*:18*Z*	TEQ^post^: 96*E*:4*Z*	PSS^528^: 89*E*:11*Z*	PSS^528^: 79*E*:21*Z*	PSS^528^: 87*E*:13*Z*	—^d)^	—^d)^
LRF1^b)^ (TEQ^a.i.^→ PSS^λ1^)	↑ 1.32 ± 0.07	↑ 1.19 ± 0.01	↑ 1.37 ± 0.02	↑ 1.48 ± 0.03	↑ 1.64 ± 0.02	↑ 1.05 ± 0.04	↑ 1.00 ± 0.01
LRF2^b)^(PSS^λ1^→ PSS^λ2^/TEQ^post^)	↓ 1.26 ± 0.08	↓ 1.15 ± 0.01	↓ 1.37 ± 0.03	↓ 1.38 ± 0.03b	↓ 1.37 ± 0.02	↓ 1.02 ± 0.05	↓ 1.01 ± 0.01

^a)^ In mg per liter expression medium.

^b)^ LRF delineates the activity ratio $\frac{v1}{v2}$ with v_1 _> v_2_ with arrows indicating an activity increase (↑) or decrease (↓). Note: data are provided as mean fit value ± standard error of fit (S.E.) of three technical replicates.

^c)^ Inaccuracy expected due to light scattering in the sample (Figure 6).

^d)^ Cannot be determined using UV/Vis spectra.

Since the chemical environment within a protein strongly diverges from that in buffer or solvent, we performed a photochemical characterization of the psUAAs within each HisF variant. Similar to the isolated psUAAs, we acquired absorbance spectra of the thermally equilibrated, as‐isolated enzymes (TEQ^a.i.^), compared PSS^λ^ or TEQ^post^ absorbance spectra, estimated the respective isomer distributions at PSS^λ^, and evaluated the photostabilities over various switching cycles.

**Figure 6:**
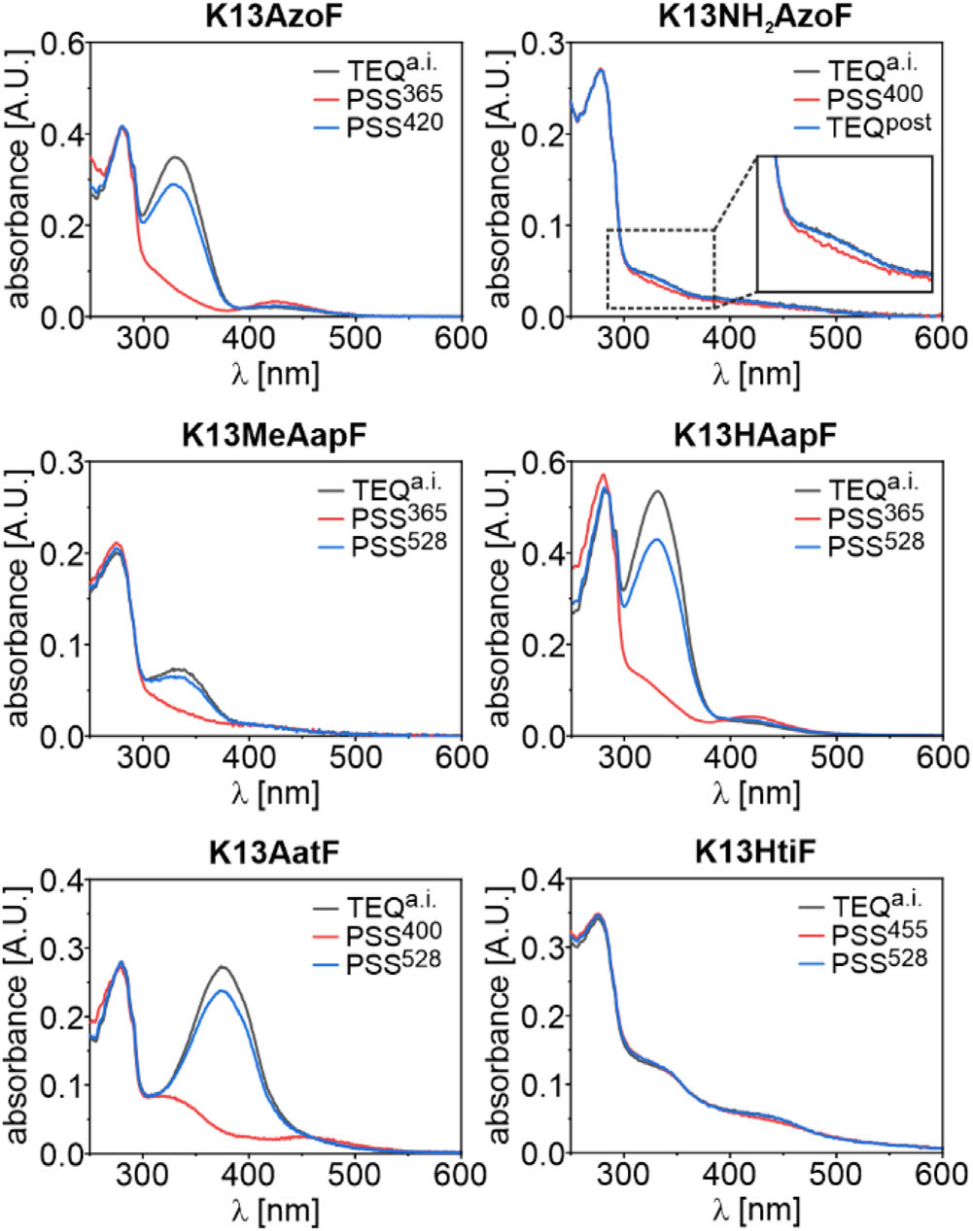
Absorbance spectra of HisF‐psUAA variants in buffer. Real‐time tracking of the absorbance signal assured the complete establishment of the respective PSS^λ^ or TEQ^post^ prior to spectrum acquisition (e.g., Figure S22).

Absorbance spectra of each K13psUAA variant in TEQ^a.i.^ comprised protein‐related maxima at ∼280 nm in addition to the characteristic maxima of each photoswitch (Figure 6). The absorbance maxima of the π→π* and n→π* transitions (Table S10) thereby coincided well with those of the isolated psUAAs (Table S1). General differences in signal strength of the photoswitches, particularly compared to the protein signal, might be attributed to quenching effects by the adjacent protein environment. Photoinduced isomerization in both directions was then monitored in real‐time using the most effective wavelengths of the isolated psUAAs in buffer and DMSO (Figure S22; Table S11). K13AzoF, K13MeAapF, K13HAapF and K13AatF showed well resolved isomerization profiles. However, K13NH_2_AzoF and K13HtiF, which already exhibited weak signals in TEQ^a.i.^, demonstrated only mediocre or even minor changes upon irradiation. Considering the higher absorbance values close to the protein signal (Figure 6), we assume that both enzymes contained soluble aggregates that could not by removed by centrifugation and that caused light scattering. We further obtained the thermal half‐lives of K13NH_2_AzoF (*t_½_* = 14 s) and K13AatF (*t_½_* = 80 min) (Figure S22) as well as the spectra of each PSS^λ^ or TEQ^post^, which aligned well with those of the isolated psUAAs, particularly regarding the spectral behavior of the π→π* and n→π* transitions (Figure 6).

In the next step, we estimated the isomer distributions at the PSS^λ^ and TEQ^post^ for the HisF variants containing diazo‐based psUAAs from UV/Vis spectra. For this, TEQ^a.i.^ was assumed to contain 100% *E*.^[^ ^28^ ^]^ For the *E*→*Z* isomerization, we observed nearly quantitative (K13AatF), good (K13AzoF, K13HAapF) and mediocre (K13NH_2_AzoF, K13MeAapF) photoconversion yields (Table 2). Likewise, *Z*→*E* isomerization obtained either quantitative (K13NH_2_AzoF), good (K13AzoF, K13MeAapF, K13AatF) or mediocre (K13HAapF) *E* isomer yields. Notably, the highest isomer distributions for K13MeAapF and K13HAapF, for which the most effective *Z*→*E* isomerization wavelengths in buffer and DMSO differed, were obtained with 528 nm (Figure S23). Likewise, we tested all isomerization wavelengths that were effective in buffer and DMSO for K13HtiF. However, since potential differences in photoconversion remained elusive owing to pronounced light scattering, we chose to use 455 nm and 528 nm, which were most effective in buffer, for further activity studies.

Overall, the determined isomer distributions aligned well with those obtained from the isolated psUAAs for **AzoF** and **AatF**, whereas **MeAapF** and **HAapF** demonstrated partly less or more quantitative photoconversions. **NH_2_AzoF** appeared to switch only inefficiently in the enzyme environment, however, the isomer distributions might be significantly underestimated due to light scattering. The photochemical characterization of **HtiF** was even more difficult and less informative. To assess whether these two enzymes are still folded properly after incorporation of **NH_2_AzoF** and **HtiF**, we performed circular dichroism spectroscopy (Figure S24). While HisF‐wt, K13AzoF, K13MeAapF, K13HAapF and K13AatF showed an intact structural integrity, the presence of **NH_2_AzoF** and **HtiF** appeared to disturb the secondary structure of HisF. This explains the light scattering effect and indicates that position K13 is unsuited to harbor **NH_2_AzoF** or **HtiF**. We were puzzled by the misfit of **NH_2_AzoF**, because **AzoF** appeared to be well accepted in this position and because the lysine native to this position also carries a primary amine. However, we considered the exposure of position K13 to the solvent to be the reason for difficulties with the hydrophobic **HtiF**. Hence, we incorporated **HtiF** within position L35, which is part of a hydrophobic pocket (Figure S25). However, although L35HtiF was properly folded (Figure S24), it also exhibited light scattering, an even less pronounced absorbance signal compared to K13HtiF, and no signal change upon irradiation (Figure S26). Further studies will show which positions, and more specifically which chemical environment, will be more suited for the incorporation of **HtiF**.

Finally, cycle performance measurements revealed that each HisF‐psUAA variant, except K13HtiF, was able to withstand ten cycles of repeated photoswitching without any obvious signs of photobleaching or light scattering (Figure S27).

With this, we confirmed that the diversity of properties regarding the photoswitch efficiencies, the thermal stabilities and the effective wavelengths of irradiation was largely maintained after the psUAAs were incorporated into an enzyme.

### Photocontrol of HisF Activity

In a first approach to assess the capability for enzymatic photocontrol, we measured HisF activities in the TEQ^a.i.^, PSS^λ^, and/or TEQ^post^ for each HisF‐psUAA variant. The light‐regulation factor (LRF), which constitutes the ratio of activities *v* before and after irradiation ($\frac{v1}{v2}$ with *v_1 _* > *v_2_*), thereby defines the photocontrol efficiency. After reconfirming that HisF‐wt is not affected by irradiation (LRF ≤ 1.05; Figure S28), we initially determined the LRF of fK13AzoF at PrFAR concentrations either in saturation (LRF < 1.2) or close to the *K_m_* value (2.5 µM)^[^ ^13^ ^]^ of HisF‐wt (LRF > 1.2) and chose the latter for all subsequent measurements (Figure S29). Based on this result, we expected modest LRF values in the range of 1.2–2.0. Hence, we used an interaction model in a global fit analysis of two data sets, for example, TEQ^a.i.^ and PSS^365^, to obtain LRF values ± standard errors (S.E.) (Table S12). This allowed us to evaluate whether differences in LRF values between the HisF‐psUAA variants are reliable. Upon *E*→*Z (Z*→*E* for **HtiF**) isomerization we observed either a relatively high (K13AatF), good (K13AzoF, K13MeAapF, K13HAapF), minor (K13NH_2_AzoF) or insignificant (K13HtiF, L35HtiF) increase in activity (Table 2). Remarkably, activities returned to initial values either with nearly full (K13MeAapF), good (K13AzoF, K13NH_2_AzoF, K13HAapF) or mediocre (K13AatF) reversibility. Notably, despite the light scattering effects and in part partial unfolding, K13NH_2_AzoF, K13HtiF and L35HtiF retained well measurable enzymatic activities.

Finally, we evaluated the correlation between the LRFs and the photochemical conversion of each psUAA. K13AatF demonstrated the most efficient photocontrol and the most quantitative *E*→*Z* switching. The other HisF‐psUAA variants did, however, not follow this pattern, for example, K13MeAapF obtained only mediocre *E*→*Z* isomerization but achieved a better LRF than K13AzoF, which exhibited the second best photoconversion after K13AatF. Similar discrepancies can be found in terms of the reversibility of photocontrol, for example, K13AatF reestablishes a higher *E*‐content in its PSS than K13HAapF but shows only mediocre reversibility. These results confirm our observation in previous studies^[^ ^11^, ^12^, ^13^ ^]^ that effective photoconversion of the psUAA only sets the foundation for photocontrol. The mechanism of photocontrol itself depends on the interaction of the psUAA with the enzyme, which causes a conformational shift upon the light‐induced isomerization of the photoswitch. As a result, different psUAAs can facilitate differently strong photocontrol effects in the same incorporation position within an enzyme as showcased in this study. With this, we could substantiate that the extended psUAA repertoire, with its broadened interaction potential, has the capacity to significantly improve the engineering efforts of reversibly photocontrolled enzymes.

## Conclusion

Protein engineering with psUAAs has become a highly promising method to design photocontrolled enzymes, particularly for the two pioneering applications biotherapy and biocatalysis. However, the current repertoire of psUAAs has been limited in terms of quantitative switching, whether through irradiation or thermal relaxation, interaction potential with the enzyme environment, and effective wavelengths of irradiation. In this work, we have enhanced the versatility of the repertoire by designing and synthesizing six psUAAs with diverse photochemical behaviors, five of which can be incorporated to facilitate advanced photocontrol of enzymatic reactions.

We have used **AzoF**, the best‐known psUAA to date, as the point of reference. This bistable compound achieves good photoconversions in both directions with UV and visible light, but only offers hydrophobic interactions with the enzyme, with which it is possible to induce catalytically relevant conformational changes. In comparison, the visible light and thermally induced switch between a charged and neutral state in **SpiroY** is extremely interesting for the photocontrol of enzyme chemistry, not least due to its ability to coordinate metal ions in one of its isomeric forms, which is highly significant for metalloenzymes. However, its unique behavior also results in non‐quantitative switching that greatly depends on the presence of charge stabilizers in close proximity. Moreover, it is the only psUAA for which further engineering studies, likely involving directed evolution and/or computational design, are required to identify an aaRS for the co‐translational incorporation. Similar to **SpiroY**, **HtiF** appears to be a blackbox regarding photoconversion yields, although full recovery of the TEQ is possible by thermal relaxation. Nevertheless, **HtiF** offers a hydrogen bond acceptor and can be switched purely with visible light. Furthermore, while the two bistable arylazopyrazole‐based psUAAs, **MeAapF** and **HAapF**, showed only mediocre to good photoconversion yields upon irradiation with UV and visible light, they can act as hydrogen bond acceptors and coordinate metals. Notably, **HAapF** and **NH_2_AzoF** can potentially participate as nucleophiles in catalysis, for example, in Friedel‐Crafts alkylation reactions and are, thus, quite valuable for biocatalysis. Additionally, **NH_2_AzoF** is a hydrogen bond donor and can be switched purely with violet light. Although we merely observed mediocre photoconversions for this psUAA, its switching efficiency may allow for further fine‐tuning by modulating the intensity of irradiation to effectively compete with its fast thermal relaxation. This also ensures full reversibility to TEQ, allowing for exact spatial control with automatic deactivation of the enzyme once it exits the target site. All of these attributes make **NH_2_AzoF** one of the most interesting psUAAs of this study. Finally, another highly appealing psUAA for enzyme engineering is **AatF**. It demonstrates excellent photochemical performance by revealing good to nearly quantitative photoconversion in both directions with visible light and short‐time bistability with the option to recover TEQ by thermal relaxation within hours. Most remarkably, it offers a versatile interaction potential with enzymes provided by its capability to act as hydrogen bond acceptor and to coordinate metals.

In conclusion, we expect the here described psUAAs to significantly increase the success rate of photocontrol engineering in enzymes. The extended repertoire promises to ease the identification of positions, in which enzyme conformation and hence catalysis can be controlled by the isomer switch. Moreover, it paves the way for photocontrolled catalysis via photoswitchable catalytic or metal‐coordinating residues in the redesign of existing enzymes or in the de novo design of novel enzymes.

## Supporting Information

The authors have cited additional references within the Supporting Information file.^[^ ^63^, ^64^, ^65^, ^66^, ^67^, ^68^, ^69^, ^70^, ^71^, ^72^, ^73^, ^74^, ^75^, ^76^, ^77^, ^78^, ^79^, ^80^, ^81^, ^82^, ^83^, ^84^, ^85^, ^86^, ^87^, ^88^, ^89^, ^90^, ^91^, ^92^ ^] 1^H‐NMR and ^13^C‐NMR as well as all mass spectrometry reports for all newly synthesized compounds are provided in DOI: 10.22000/41f30q9xz9mmznqp.

## Conflict of Interests

The authors declare no conflict of interest.

## Supporting information



Supporting Information

Supporting Information

## Data Availability

The data that support the findings of this study are available in the Supporting Information of this article.
