# Supplementary material for: Expanding the Repertoire of Photoswitchable Unnatural Amino Acids for Enzyme Engineering
Source: Angew Chem Int Ed Engl. 2025 Aug 4;64(38):e202508562. doi: 10.1002/anie.202508562 (PMC12435433; doi:10.1002/anie.202508562)
Supplement: Supplementary file 1 — Supporting Information [file ANIE-64-e202508562-s001.pdf]

Supporting Information

Expanding the Repertoire of Photoswitchable Unnatural Amino Acids for Enzyme Engineering

Caroline Hiefinger,†[a] Michela Marcon,†[b] Verena Pape,[a] Guillem Casadevall,[c] Ranit Lahmy,[b] Christoph Haag,[b] Julian Nazet,[a] Michael Bartl,[a] Astrid Bruckmann,[d] Sílvia Osuna,[c,e] Burkhard König,[b] and Andrea Hupfeld*[a]

[a] C. Hiefinger, M. Bartl, V. Pape, Dr. A. Hupfeld (née Kneuttinger)
Institute of Biophysics and Physical Biochemistry and Regensburg Center for Biochemistry
University of Regensburg
Universitätsstraße 31, D-93053 Regensburg (Germany)
E-mail: andrea.hupfeld@ur.de

[b] M. Marcon, Dr. R. Lahmy, C. Haag, Prof. Dr. B. König
Institute of Organic Chemistry
University of Regensburg
Universitätsstraße 31, D-93053 Regensburg (Germany)

[c] G. Casadevall, S. Osuna
Institut de Química Computacional i Catàlisi and Departament de Química
Universitat de Girona
c/ Maria Aurèlia Capmany 69, 17003 Girona (Spain)

[d] A. Bruckmann
Institute of Biochemistry, Genetics and Microbiology,
University of Regensburg,
Universitatsstrasse 31, D-93053 Regensburg (Germany)

[e] S. Osuna
ICREA, Pg. Lluís Companys 23, 08010 Barcelona (Spain)

†The authors contributed equally

Table of Contents

1. Supporting material **4**

Figure S1. 4
Extended Text S1. 5
Extended Text S2. 6
Extended Text S3. 6
Extended Text S4. 8
Extended Text S5. 8
Extended Text S6. 9
Figure S2. 11
Table S1. 11
Figure S3. 12
Figure S4. 12
Figure S5. 13
Table S2. 14
Table S3. 15
Figure S6. 16
Figure S7. 17
Figure S8. 18
Figure S9. 18
Table S4. 19
Figure S10. 20
Figure S11. 21
Table S5. 22
Table S6. 23
Figure S12. 24
Figure S13. 24
Table S7. 25
Table S8. 27
Figure S14. 27
Figure S15. 27
Table S9. 28
Figure S16. 28
Figure S17. 29
Figure S18. 29
Figure S19. 30
Figure S20. 30
Figure S21. 31
Table S10. 32
Figure S22. 33
Table S11. 34
Figure S23. 34
Figure S24. 35
Figure S25. 35
Figure S26. 35
Figure S27. 36
Figure S28. 36
Figure S29. 37
Table S12. 38
Table S13. 39

2. Experimental section **41**

2.1 Synthesis of photoswitchable UAAs *41*
2.1.1 Schemes 42
Scheme S1. 42

Scheme S2. 42
Scheme S3. 42
Scheme S4. 42
Scheme S5. 43
Scheme S6. 43
Scheme S7. 43
2.1.2 Procedures 44
2.1.3 ^{1}H-NMR Spectra 53
2.1.4 13C-NMR Spectra 66

2.2 Irradiation Devices *74*
Table S14. 74

2.3 UV/Vis absorbance spectra *74*

2.4 Wavelength screen and determination of isomerization rates *75*

2.5 Cycle performance *76*

2.6 Determination of isomer ratios via high pressure liquid chromatography *76*

2.7 Estimation of isomer ratios from UV/Vis spectra *77*

2.8 Thermal stability *78*

2.9 Computational Design of aaRS *79*

2.10 Subcloning and site-directed mutagenesis *79*

2.11 Synthetase screening *81*

2.12 Computational methods *81*
Table S15. 82

2.13 Mass spectrometry *83*

2.14 Expression of HisF-psUAA variants in midi-scale *84*

2.15 Heterologous expression of HisA *85*

2.16 CD spectroscopy *85*

2.17 Biochemical synthesis of ProFAR *85*

2.18 Activity measurements *86*
Scheme S8. 86

References 88

1. Supporting material

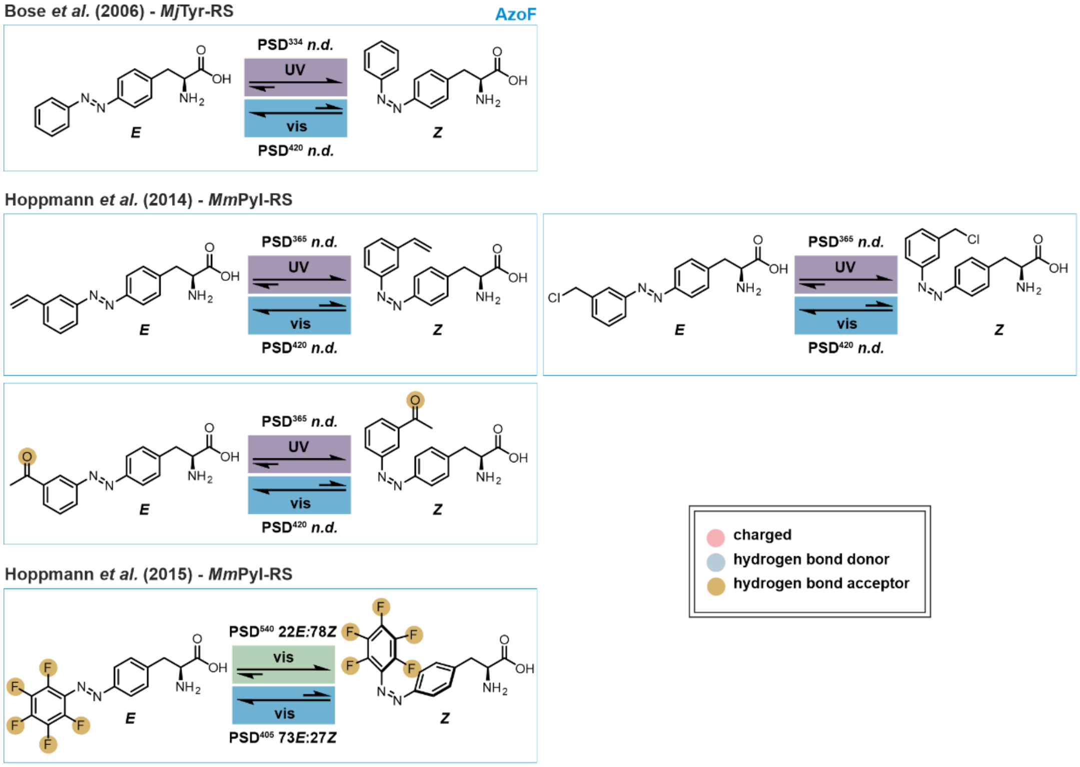

Photochemical characterization of Boc-protected UAAs

X	R	PSD530	PSD448
H	3-CN	n.d.	n.d.
H	2,6-F_2	34E:66Z	62E:38Z
H	2,4,6-F_3	38E:62Z	61E:39Z
H	2,3,4,5,6-F_5	43E:57Z	64E:36Z
F	2,6-F_2	36E:64Z	60E:40Z
F	2,4,6-F_3	24E:76Z	61E:39Z
F	2,3,4,5,6-F_5	35E:65Z	62E:38Z
Cl	2,6-F_2	23E:77Z	70E:30Z
Cl	2,4,6-F_3	28E:72Z	71E:29Z

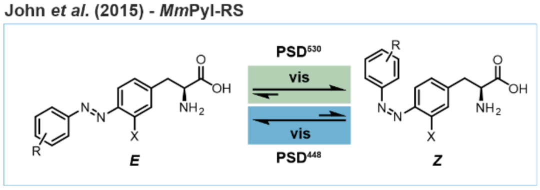

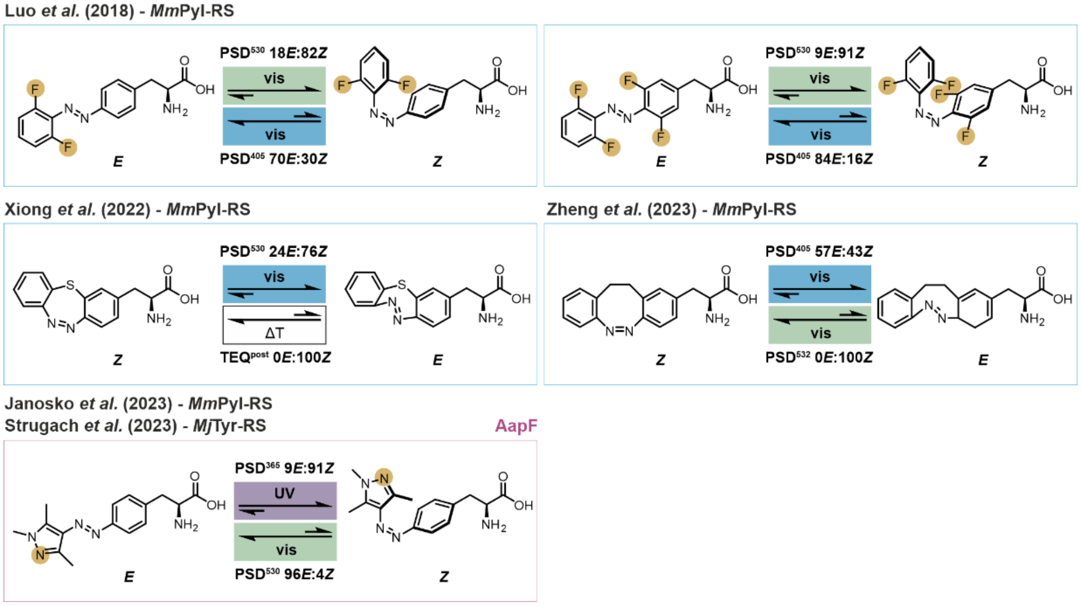

Figure S1. Hitherto available psUAAs and corresponding aaRSs as reported by Bose *et al.*,[1] Hoppmann *et al.*,[2] John *et al.*,[3] Luo *et al.*,[4] Xiong *et al.*,[5] Zheng *et al.*,[6] Janosko *et al.*,[7] and Strugach *et al.*.[8]

Extended Text S1. Structure–activity relationship introduction

In general, the structure–activity relationship of each synthesized psUAA is governed by the nature of the photoswitch scaffold and the presence of functional substituents. For example, **AzoF** contains an unsubstituted azobenzene. The *E* isomer of azobenzene is planar and exhibits C_{2h} symmetry,[9] which favors a strong light absorbance at ~320 nm owing to its π–π* transition, but prohibits light absorbance at the n–π* transition causing a weak signal at ~430 nm.[10] In the TEQ of **AzoF**, which contains nearly 100% *E*, both maxima were mostly unaffected by the additional amino acid moiety in para position to the azo group (**Figure 3**, black; **Table S1**). For all photoswitches, photoconversion rates and yields depend on the extinction coefficient of the isomer at the specified wavelength (ε^{λ}), giving the probability that a molecule is excited, and the quantum yields of both isomerization reactions at the specified wavelength ($\phi^{\lambda}_{E\to Z}$ and $\phi^{\lambda}_{Z\to E}$), representing the probability that the molecule isomerizes. Since the absorbance spectra of the *E* and *Z* isomer overlap, both isomerization reactions occur throughout the UV/Vis spectrum with different extinction coefficients (e.g., $\varepsilon^{365nm}_{E} \gg \varepsilon^{365nm}_{Z}$ for azobenzene) and different quantum yields (e.g., $\phi^{365nm}_{E\to Z}$ ~ 0.16 and $\phi^{365nm}_{Z\to E}$ ~ 0.39 for azobenzene).[11] As a result, the *E*:*Z* composition obtained upon irradiation differs with the chosen wavelength. Hence, to find the photostationary state PSS^{λ} with the most effective photoconversion yields, we tested different wavelengths of irradiation (**Figure S4**). Upon 365 nm irradiation, **AzoF** demonstrated the largest change in the UV/Vis spectrum evident of an enrichment of the *Z* isomer (**Table S5**). This isomer exhibits a non-planar configuration with C_2 symmetry,[12] which weakens and shifts the π–π* transition towards lower wavelengths, but increases the signal at ~430 nm owing to a now allowed n–π* transition (**Figure 3**, red).[13] Upon 420 nm irradiation, **AzoF** reached the PSS with the apparently highest *E*-content (**Figure 3**, blue).

For reasons of clarity, we will only focus on the photochemical properties obtained in buffer in the Extended Texts S1–S6, since they were overall consistent with the results in DMSO.

Extended Text S2. Structure–activity relationship of **NH_2AzoF**

The *ortho*-substitution of azobenzene with an electron-donating amino group in **NH_2AzoF** impacts its switching behavior and photochemical properties. It was previously reported that *ortho*-amination leads to a pronounced red-shift of the absorbance spectrum and markedly decreases the thermal stability of the *Z* isomer.[14] This is reflected in the absorbance profile of **NH_2AzoF**, which shows a broadened and intensified n–π* transition at ~420 nm attributable to the lone-pair participation of the amino group in the conjugated system (**Figure 3**, black).[15] Consequently, **NH_2AzoF** can undergo *E*→*Z* photoisomerization with visible light at 400 nm, which obtained similarly high photoconversion yields than 365 nm and 385 nm (**Figure S4, Table S5**). The resulting PSS^{400} yields a moderate *E*:*Z* ratio of 46:54 (**Table S5**), which not only depends on the wavelength of irradiation but also the intensity of irradiation due to the short thermal half-life ($t_{1/2}$ = 9.9 s) of **NH_2AzoF**. *Z*→*E* switching was accordingly most effective by thermal relaxation, which facilitates rapid and complete thermal recovery of 100% *E* isomer post-irradiation (**Table 1**).

In terms of interaction potential, **NH_2AzoF** retains the hydrophobic and π-stacking capacity of **AzoF**, while the *ortho*-amino group adds opportunities for hydrogen bonding with amino acid side chains and the protein backbone. These features could enhance local residue interactions, particularly in polar or charged microenvironments within enzymes and contribute to catalytically important conformational changes.

Extended Text S3. Structure–activity relationship of **MeAapF and HAapF**

Heteroaryl azo compounds, such as arylazopyrazoles and arylazothiazoles (see **Extended Text S4** for a description of **AatF**), offer a diverse set of functionalities compared to classical azobenzenes while retaining the overall structure-activity relationship as described in **Extended Text S1**.[16] Their modular heteroarene moieties enable precise tuning of thermal stability, photoconversion efficiency, and solubility in aqueous environments. In general, arylazopyrazoles exhibit π–π* and n–π* transitions in similar spectral regions compared to azobenzenes (**Table S1**). Depending on the substituents both transition maxima can be shifted more strongly between the *E* and *Z* isomer allowing for more quantitative photoconversion yields in both directions.[17] For example, methylated arylazopyrazole is known to exhibit improved separation of π–π*

and n–π* transitions, resulting in high photoconversion yields of >97% of the desired isomer in either switching direction.[17] However, we could not confirm this behavior in **MeAapF** or **HAapF**. Similar to **AzoF**, light of 365 nm obtained the highest photoconversion yields (**Figure S4**), which were only slightly improved for **MeAapF** (8*E*:92*Z*) but reduced for **HAapF** (24*E*:76*Z*) compared to **AzoF** (14*E*:86*Z*; **Table S5**). The changes in the PSS^{365} spectra, which are indicative of the isolated *Z* spectra, explain this (**Figure 3**, red). The π–π* transition maxima of **MeAapF** in its TEQ and PSS^{365} are slightly better separated ($\Delta\lambda_{TEQ-365}$ = 55 nm) than for **AzoF** ($\Delta\lambda_{TEQ-365}$ = 25 nm) in line with the higher photoconversion yields. However, the π–π* transition maxima of **HAapF** are similarly better separated ($\Delta\lambda_{TEQ-365}$ = 52), which does not explain the reduced *E*→*Z* photoconversion yields. Therefore, we assume that **HAapF** exhibits lower quantum yields at 365 nm, resulting in less efficient photoconversion. Remarkably, the n–π* transition maxima remained within the same wavelength range in both TEQ and PSS^{365} spectra for **MeAapF**, **HAapF** and **AzoF** without the expected red-shift as observed for other arylazopyrazoles. Accordingly, similar photoconversion yields in the backswitch were obtained for all three psUAAs (**Table S5**). Interestingly, despite a relatively strong n–π* signal at ~418 nm for **MeAapF** and **HAapF**, the apparently most effective wavelength for *Z*→*E* isomerization was 528 nm positioned at the tail of the signal (**Figure 3**, blue; **Figure S4**). Primarily, this result suggests that *Z*→*E* photoconversion yields are primarily driven by higher quantum yields of isomerization at this wavelength compared to more blue-shifted wavelengths. Additionally, it has to be noted that this effect might in part result from the Gaussian emission profile of LEDs, which in the case of the 528 nm LED includes wavelengths that overlap with the higher wavelengths of the n–π* signal.

Substitution of the heteroaryl ring was further shown to markedly influence the thermal stability of arylazopyrazoles.[17] The methyl group in **MeAapF** thereby enhances the steric and hydrophobic character, which increases the thermal half-life of the *Z* isomer to 45 d compared to **AzoF** ($t_{1/2}$ = 16 d; **Table 1**). In contrast, **HAapF** contains a secondary amine within the pyrazole ring, which moderately reduces the thermal stability of the *Z* isomer ($t_{1/2}$ = 10 d) while maintaining overall bistability.

Both **MeAapF** and **HAapF** retain the hydrophobic character of **AzoF**. However, the π-stacking capacity is reduced to only one phenyl ring in the phenylalanine moiety. Instead, both psUAAs offer hydrogen bond acceptors in their pyrazole ring, and **HAapF** extends

this potential for polar interactions with a hydrogen bond donor (**Figure 1**). Finally, the nitrogen atoms in the heteroaromatic ring within arylazopyrazoles are capable of coordinating metals.[18] These properties might allow both arylazopyrazole scaffolds to engage in a different interaction network than **AzoF**, particularly in a polar microenvironment within enzymes and within metalloenzymes.

Extended Text S4. Structure–activity relationship of **AatF**

Similar to Arylazopyrazoles, many arylazothiazole photoswitches show well-separated absorbance signals in their *E* and *Z* isomers allowing for more quantitative photoconversions, while retaining the overall structure-activity relationship as described in **Extended Text S1**. In our study, **AatF** demonstrated a significantly red-shifted π–π* absorbance maximum of 371 nm in its TEQ (**Table S1**), which facilitated efficient *E*→*Z* isomerization with visible light at 400 nm (**Figure S4**). Owing to the improved separation of the π–π* transition maximum in its PSS^{400} ($\lambda_{max,400}$ = 323 nm; $\Delta\lambda_{TEQ-400}$ = 48) compared to **AzoF** ($\Delta\lambda_{TEQ-365}$ = 25), **AatF** also obtained improved *E*→*Z* photoconversion yields of 9*E*:91*Z*. In contrast, absorbance values in the range of the n–π* transition maximum (~455 nm; **Table S1**) were similar in TEQ and PSS^{400}, which is indicative of the isolated *Z* spectrum. As a result, photoconversion yields of the backswitch were comparable to **AzoF** (81*E*:19*Z*) with 84*E*:16*Z* (**Table S5**).

The *Z* isomer thermal half-life is comparably low ($t_{1/2}$ = 107 min), making **AatF** suitable for transient switching applications (**Table 1**). Its robust switching performance and compatibility with aqueous systems further support its biological applicability.

Ultimately, the thiazole moiety offers similar properties than the pyrazole moiety including hydrogen bond acceptors and the potential for metal ion coordination via the nitrogen atoms. [19] However, this interaction profile also comes at the cost of a reduced π-stacking capacity, while tuning the potential to interfere with an enzyme in a different manner than **AzoF**.

Extended Text S5. Structure–activity relationship of **HtiF**

Hemithioindigos that undergo *Z*↔*E* isomerization around an olefinic bond instead of a diazo bond, have recently emerged as attractive alternatives to azobenzene derivatives due to their rapid thermal relaxation and exclusive responsiveness to visible light.[20,21]

Both isomers, the thermally more stable *Z* and the metastable *E*, are planar and exhibit C_S symmetry.[22] Only excitation of the π–π* transition above 350 nm contributes to the isomerization process, which leads to a reduction and red-shift of the transition maximum in the *E* isomer compared to the *Z* spectrum.[22] In line with this general observation, **HtiF** exhibited a pronounced π–π* signal at λ_{max} = 441 nm in its TEQ (**Figure 3**, black, **Table S1**). Upon 420 nm and 455 nm irradiation (**Figure S4**), the highest *E*-content was obtained (77*E*:23*Z* in DMSO; **Table S6**) decreasing the absorbance intensity and shifting the λ_{max} of the π–π* signal to 446 nm. In contrast to azo compounds, the backswitch (*E*→*Z*) of hemithioindigos is also induced by irradiation of the π–π* signal. However, individual excitation of the *E* isomer is achieved by irradiation with longer wavelengths owing to the red-shifted λ_{max} of the isolated *E* isomer. As a result, the most effective wavelength to induce *E*→*Z* switching of **HtiF** was 528 nm (**Figure S4**). With this, **HtiF** can be switched purely with visible light. Moreover, it shows a short thermal half-life of 2 min (**Table 1**), which is comparable to many other hemithioindigos.[20,21]

Finally, **HtiF** expands the chemical and mechanistic diversity of psUAAs and additionally enables hydrogen bonding interactions via its carbonyl carbon,[23] offering valuable functional diversity for transient and dynamic biological applications.

Extended Text S6. Structure–activity relationship of **SpiroY**

The sixth psUAA, **SpiroY**, equally expands the mechanistic diversity due to its spiropyran scaffold undergoing an electronical rearrangement between an open and a closed form. Thereby, the *O* isomer of spiropyrans is more flexible, charged and harbors a hydroxy group capable of hydrogen bonding interactions, whereas the closed form is more rigid and neutral.[24] At high concentrations in organic solvents, the *O* form of spiropyran dominates,[25] as observed for **SpiroY** right after dilution from the DMSO stock solution. This is evident from its characteristic absorbance maxima associated with the extended π-conjugation of the open form (**Figure 3**). In contrast, the closed form lacks absorbance due to the disrupted conjugation. Upon dilution in aqueous buffer, formation of a new equilibrium towards the *C* isomer was observed. This process is most likely attributed to the pH sensitivity of spiropyrans as dilution in 50 mM Tris pH 7.5 shifts the pH, resulting in the protonation of the *O* form, which, in turn drives isomerization towards the *C* form.[26] Spiropyrans typically undergo ring-closing upon irradiation with visible light and ring-

opening either thermally or with UV light. Accordingly, irradiation of **SpiroY** at 455 nm promoted ring closure (**Figure S4**), which then thermally reverted to the open form with a relatively short half-life of 1.9 min (**Table 1**). As the *O*–*C* transition is highly sensitive to environmental conditions, the photoconversion and the thermal half-life are strongly context-dependent. While ring closure was highly efficient with a PSS^{455} of 5*O*:95*C*, re-opening only proceeded up to the equilibrium established after dilution, limiting full reversibility (**Table S5**). Notably, the versatile character as well as the reversible switching between contrasting physicochemical states enables **SpiroY** to modulate its interaction potential with surrounding residues upon irradiation, which makes it particularly attractive for the use in responsive biomolecular environments.

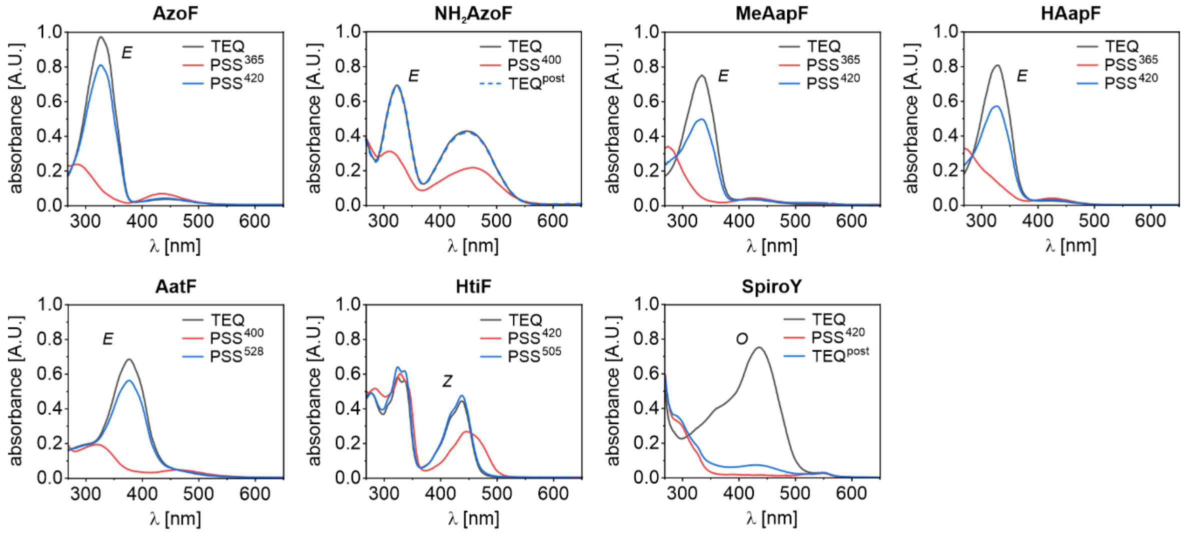

Figure S2. Absorbance spectra of the psUAAs in DMSO. Real-time tracking of the absorbance signal assured the complete establishment of the respective PSS$^{\lambda}$ or TEQ^{post} prior to spectrum acquisition (**Table S3** and **Figure S7**). The most abundant isomer in the TEQ is indicated next to the grey spectrum.

Table S1. Absorbance maxima λ_{max} of each psUAA in 50 mM Tris/HCl pH 7.5 and DMSO.

		buffer		DMSO	
psUAA	isomer	λ_{max}[a] [nm]	λ_{max}[b] [nm]	λ_{max}[a] [nm]	λ_{max}[b] [nm]
**AzoF**	*E*	324	423	329	446
	Z	299	427	287	437
**NH₂AzoF**	*E*	323	404	325	449
	Z	314	443	311	461
**MeAapF**	*E*	333	419	335	426
	Z	278	417	275	427
**HAapF**	*E*	326	412	329	424
	Z	274	419	271	425
**AatF**	*E*	371	—[c]	378	—[c]
	Z	323	455	325	467
**HtiF**	*E*	328	446	329	438
	Z	333	441	330	446
**SpiroY**	*O*	377	530	438	—[c]
	C	—[c]	—[c]	—[c]	—[c]

[a] π→π* transition in case of diazo compounds. [b] n→π* transition in case of diazo compounds. [c] no spectroscopic signal.

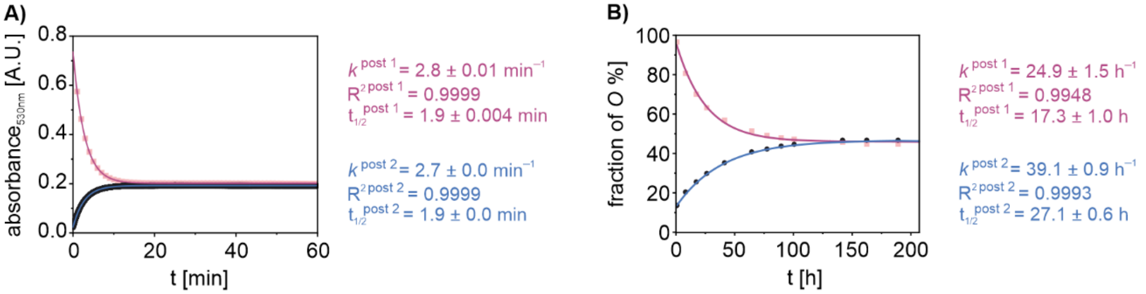

Figure S3. Establishment of TEQ^{post1} and TEQ^{post2} of **SpiroY** in 50 mM Tris/HCl pH 7.5 (A) or DMSO (B). TEQ^{post1} is established after dilution of the **SpiroY** stock solution ($k^{post\ 1}$) and TEQ^{post2} after thermal relaxation post irradiation with 455 nm ($k^{post\ 2}$). For the fast relaxation in buffer (A), the absorbance of *O* was followed over time. For the slow relaxation in DMSO (B), the isomer ratios at certain timepoints were determined via HPLC and the fraction of *O* was plotted against the time. Mono-exponential fitting yielded the rate constants *k* ± standard error of fit (S.E.). Corresponding thermal half-lives $t_{1/2}$ were deduced from the respective *k* values.

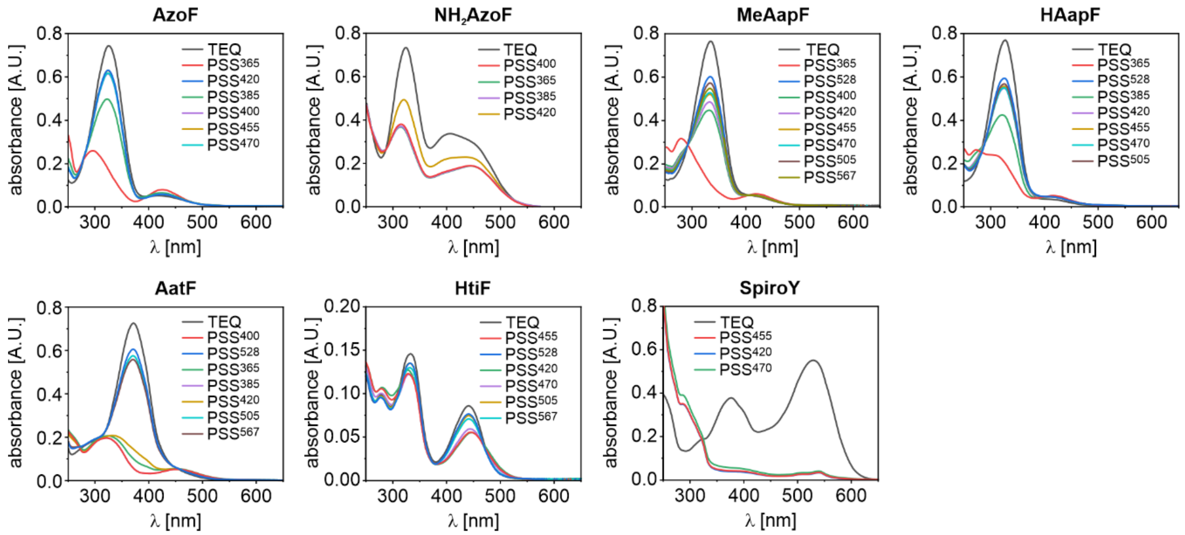

Figure S4. Absorbance spectra of each psUAA in 50 mM Tris/HCl pH 7.5 were acquired in the TEQ and in the PSS$^{\lambda}$ after irradiation with various wavelengths (*λ*). Real-time tracking of the absorbance signal assured the complete establishment of each PSS$^{\lambda}$ (**Table S2** and **Figure S6**). The most effective wavelengths, which induce the highest PSS for each isomer, were selected for further experiments. Note that for **NH$_2$AzoF** the *E*:*Z* distribution greatly depends on the intensity of irradiation owing to the fast thermal relaxation (cf. **Figure S6** and **Figure S8**). In this case, the most effective wavelength was 400 nm, because it induced the highest PSS with the lowest intensity of irradiation (**Table S14**).

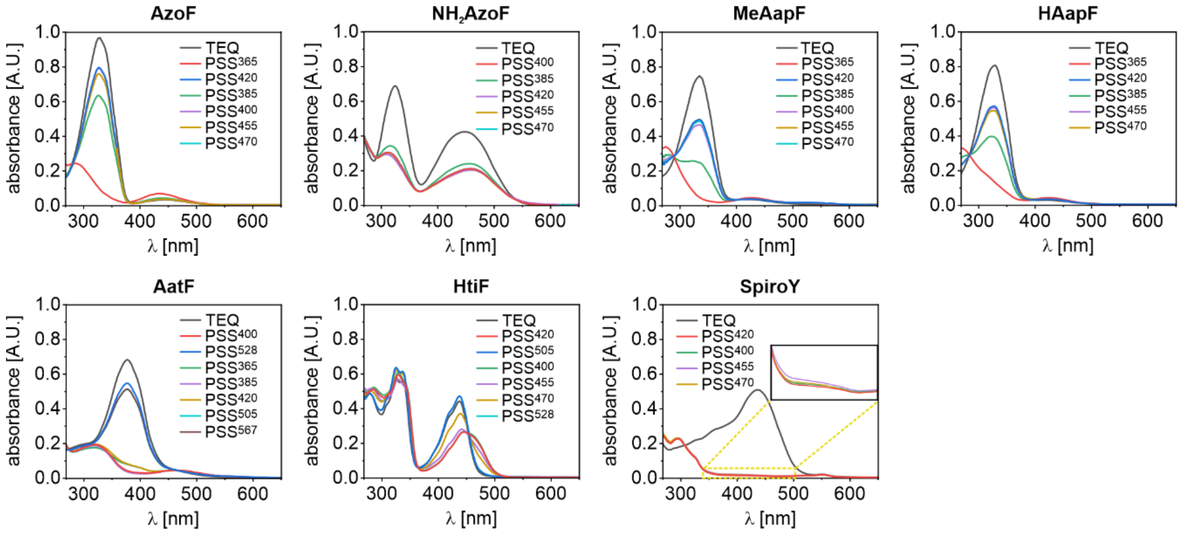

Figure S5. Absorbance spectra of each psUAA in DMSO were acquired in the TEQ and in the PSS$^{\lambda}$ after irradiation with various wavelengths (λ). Real-time tracking of the absorbance signal assured the complete establishment of each PSS$^{\lambda}$ (**Table S3** and **Figure S7**). The most effective wavelengths, which induce the highest PSS for each isomer, were selected for further experiments. Note that for **NH_2AzoF** the *E*:*Z* distribution greatly depends on the intensity of irradiation owing to the fast thermal relaxation (cf. **Figure S7**). In this case, the most effective wavelength was 400 nm, because it induced the highest PSS with the lowest intensity of irradiation (**Table S14**).

Table S2. Determination of isomerization half-lives ($t_{½}$) for each irradiation wavelength (λ) in 50 mM Tris/HCl pH 7.5.

psUAA	λ[a]	$t_{½}$[b] [s]
**AzoF**	365 nm	1.3 ± 0.006
	385 nm	3.0 ± 0.02
	400 nm	1.8 ± 0.002
	420 nm	0.9 ± 0.001
	455 nm	1.0 ± 0.001
	470 nm	1.8 ±0.001
**NH_2AzoF**	365 nm	0.7 ± 0.001
	385 nm	1.0 ± 0.001
	400 nm	0.6 ± 0.002
	470 nm	0.8 ± 0.0004
**MeAapF**	365 nm	0.7 ± 0.003
	400 nm	1.3 ± 0.001
	420 nm	1.1 ± 0.001
	455 nm	0.8 ± 0.003
	470 nm	1.8 ± 0.001
	505 nm	32.5 ± 0.05
	528 nm	129.1 ± 0.06
	567 nm	108.0 ± 0.03
**HAapF**	365 nm	1.2 ± 0.002
	385 nm	3.0 ± 0.004
	420 nm	1.03 ± 0.005
	455 nm	1.4 ± 0.005
	470 nm	2.5 ± 0.005
	505 nm	35.1 ± 0.03
	528 nm	164.7 ±0.04
**AatF**	365 nm	0.4 ± 0.007
	385 nm	0.6 ± 0.008
	400 nm	0.6 ± 0.004
	420 nm	0.6 ± 0.005
	505 nm	5.5 ± 0.004
	528 nm	13.8 ± 0.01
	567 nm	18.3 ± 0.01
**HtiF**	420 nm	0.7 ± 0.003
	455 nm	0.5 ± 0.002
	470 nm	0.8 ± 0.02
	505 nm	3.3 ± 0.02
	528 nm	13.2 ± 0.02
	567 nm	118.5 ± 0.02
**SpiroY**	420 nm	–[c]
	455 nm	–[c]
	470 nm	–[c]

[a] The most effective wavelengths according to **Figure S4** are highlighted in red (first switch) and blue (back switch). [b] $t_{½}$ ± S.E. values were derived from the respective isomerization rates k ± S.E. (**Figure S6**). [c] Isomerization rates of **SpiroY** could not be determined because the establishment of TEQ[post 1] prevented an unambiguous fit.

Table S3. Determination of isomerization half-lives ($t_{½}$) for each irradiation wavelength (λ) in DMSO.

psUAA	λ[a]	$t_{½}$[b] [s]	psUAA	λ[a]	$t_{½}$[b] [s]
**AzoF**	365 nm	2.0 ± 0.003	**AatF**	365 nm	0.4 ± 0.006
	385 nm	7.4 ± 0.0003		385 nm	0.5 ± 0.007
	400 nm	3.1 ± 0.0004		400 nm	0.5 ± 0.005
	420 nm	1.2 ± 0.0004		420 nm	0.4 ± 0.006
	455 nm	0.9 ± 0.0003		505 nm	3.5 ± 0.002
**NH_2AzoF**	385 nm	1.4 ± 0.002		528 nm	8.6 ± 0.004
	400 nm	0.9 ± 0.001		567 nm	11.6 ± 0.007
	420 nm	0.3 ± 0.001	**HtiF**	400 nm	0.02 ± 0.001
	455 nm	0.03 ± 0.001		420 nm[d,e]	0.35 ± 0.003 | 29.5 ± 1.47
	470 nm	0.4 ± 0.005		455 nm	0.2 ± 0.0001
**MeAapF**	365 nm	0.5 ± 0.002		470 nm	0.6 ± 0.001
	385 nm	2.0 ± 0.002		505 nm[d]	7.2 ± 0.007
	400 nm	2.9 ± 0.001		528 nm	42.1 ± 0.02
	420 nm	1.0 ± 0.002	**SpiroY**	420 nm[d]	–[c]
	455 nm	0.7 ± 0.001		400 nm	–[c]
	470 nm	2.4 ± 0.001		455 nm	–[c]
**HAapF**	365 nm	1.4 ± 0.003		470 nm	–[c]
	385 nm	4.4 ± 0.006			
	420 nm	1.2 ± 0.004			
	455 nm	0.8 ± 0.007			
	470 nm	1.7 ± 0.003			

[a] The most effective wavelengths according to **Figure S5** are highlighted in red (first switch) and blue (back switch). [b] $t_{½}$ ± S.E. values were derived from the respective isomerization rates k ± S.E. (**Figure S7**). [c] Isomerization rates of **SpiroY** could not be determined because the establishment of TEQ[post] [1] prevented an unambiguous fit. [d] Discrepancies were observed in the effective wavelengths in DMSO compared with the measurements in buffer. [e] Two $t_{½}$ values were obtained relating to the double-exponential fit for HtiF (**Figure S7**).

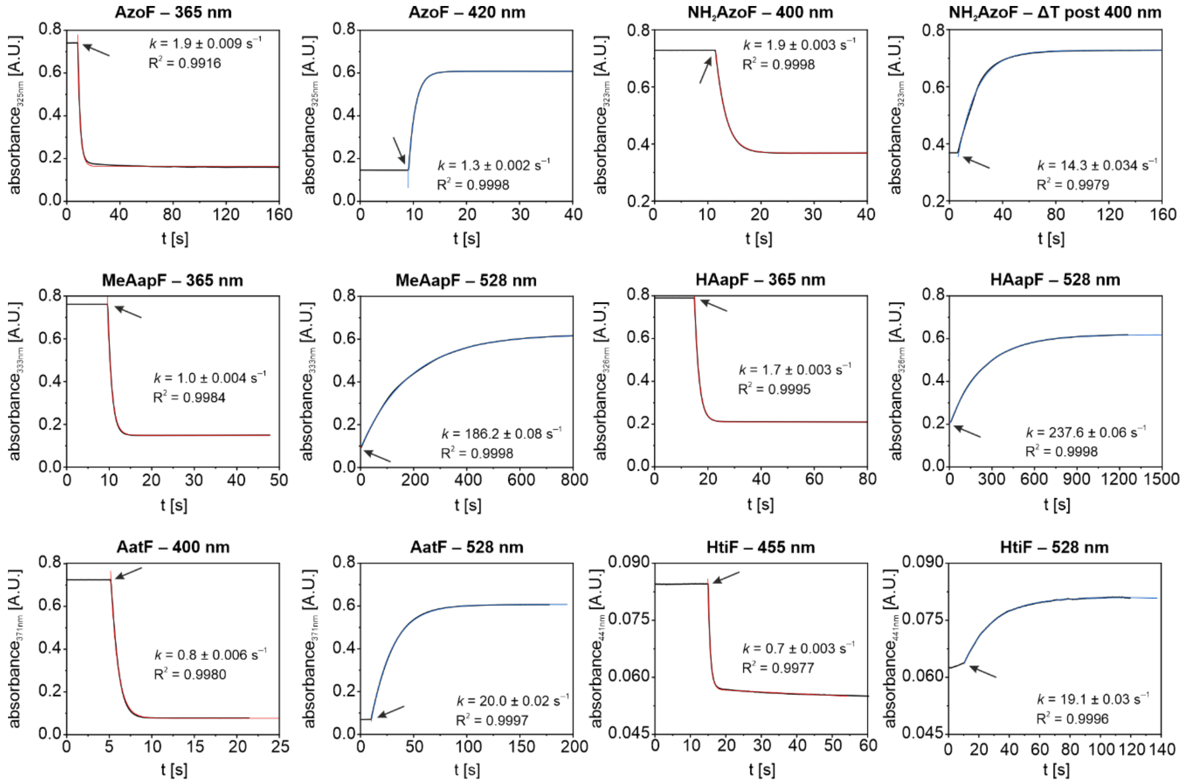

Figure S6. Exemplary isomerization rates of each psUAA in 50 mM Tris/HCl pH 7.5 upon irradiation with the most effective wavelengths (**Figure S4**) or through thermal relaxation (*ΔT*). The absorbance was followed at the π→π* or n→π* transition. Irradiation (arrow) was started and either maintained until a plateau was reached (for evaluation of isomerization upon irradiation) or stopped (for evaluation of thermal relaxation). The rates of isomerization *k* ± S.E. were determined by fitting the data with a mono-exponential function. Isomerization rates of **SpiroY** could not be determined because the establishment of $TEQ^{post\,1}$ prevented an unambiguous fit. Half-lives of isomerization $t_{½}$ ± S.E. were deduced from the respective *k* ± S.E. values and are listed in **Table S2**. The color code is according to **Figure 3** and **Table S2**.

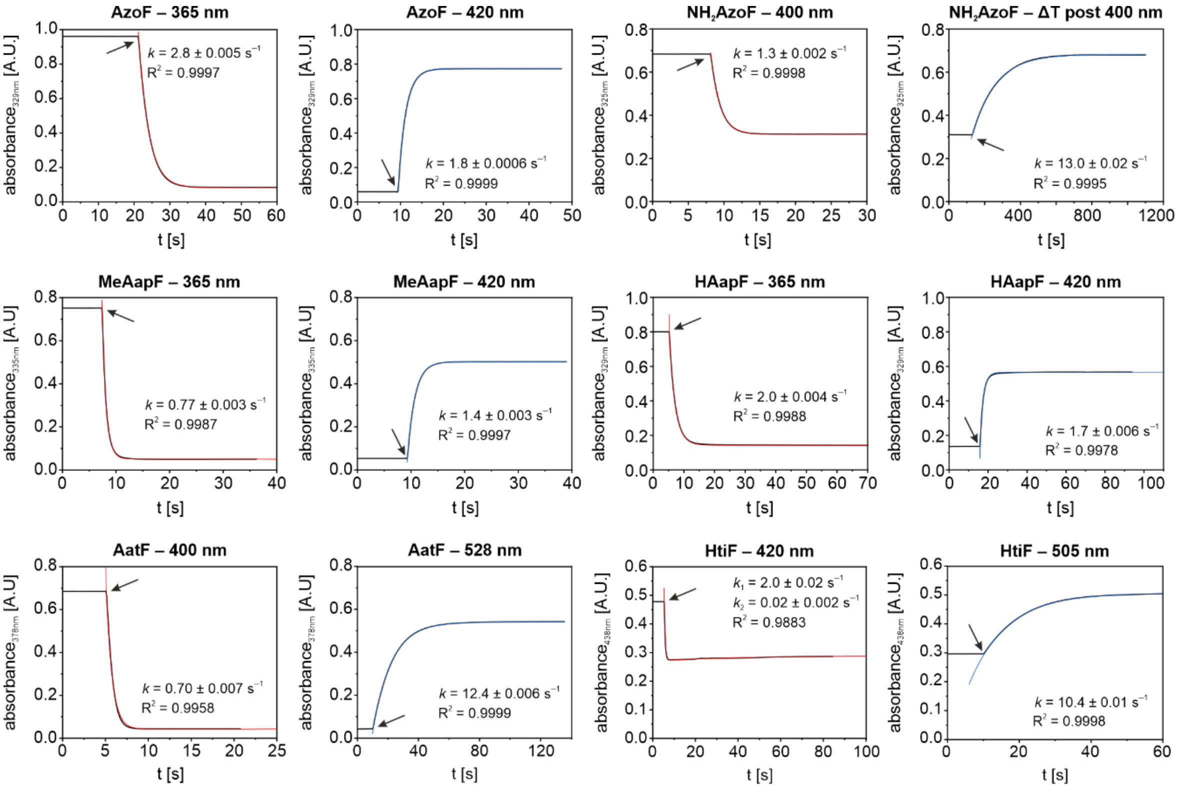

Figure S7. Exemplary isomerization rates of each psUAA in DMSO upon irradiation with the most effective wavelengths (**Figure S5**) or through thermal relaxation (*ΔT*). The absorbance was followed at the π→π* or n→π* transition. Irradiation (arrow) was started and either maintained until a plateau was reached (for evaluation of isomerization upon irradiation) or stopped (for evaluation of thermal relaxation). The rates of isomerization *k* ± S.E. were determined by fitting the data with a mono-exponential function. Isomerization rates of **SpiroY** could not be determined because the establishment of $TEQ^{post\ 1}$ prevented an unambiguous fit. In case of **HtiF**, a double-exponential behavior was observed, presumably originating from the underlying isomerization mechanism of hemithioindigos comprising various transient intermediates.[27] Half-lives of isomerization $t_{½}$ ± S.E. were deduced from the respective *k* ± S.E. values and are listed in **Table S3**. The color code is according to **Figure S2** and **Table S3**.

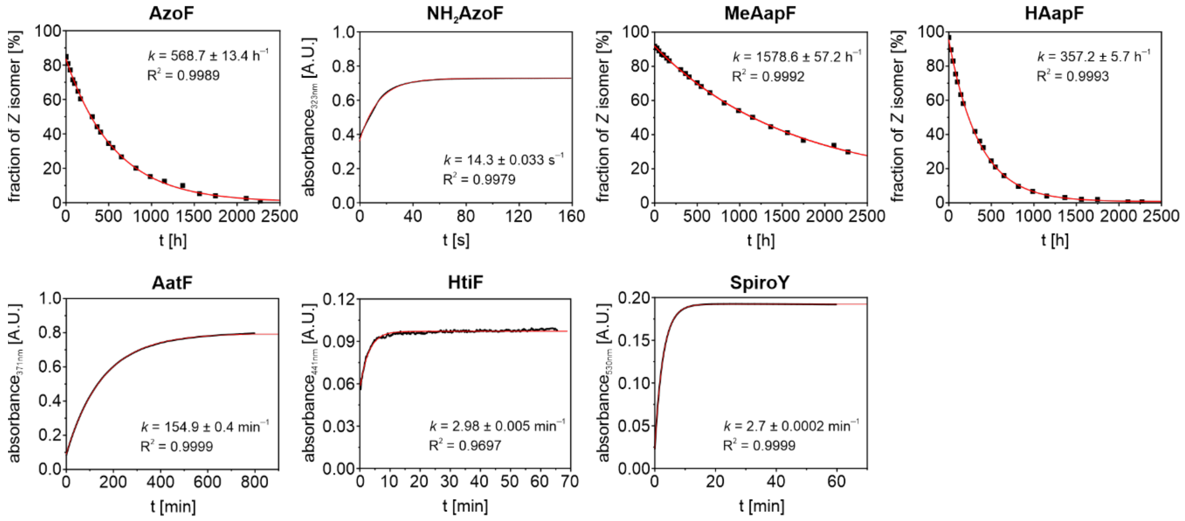

Figure S8. Thermal half-lives of each psUAA in 50 mM Tris/HCl pH 7.5 at 25 °C. Starting points of the measurements were the PSS^{λ} enriched in the thermally less stable isomer: PSS^{365} for **AzoF**, PSS^{385} for **NH₂AzoF**, PSS^{365} for **MeAapF**, PSS^{340} for **HAapF**, PSS^{400} for **AatF**, PSS^{455} for **HtiF**, and PSS^{455} for **SpiroY**. Note that we chose to irradiate **HAapF** with 340 nm, in contrast to all other experimental setups, since this wavelength achieved a remarkable PSS distribution of 9*E*:91*Z* (**Figure S9**). For psUAAs with a fast thermal relaxation (**NH₂AzoF**, **AatF**, **HtiF**, **SpiroY**), the absorbance at the π→π* or n→π* transition was followed over time. For psUAAs with longer rates of thermal isomerization (**AzoF**, **MeAapF**, **HAapF**), the isomer ratios at certain timepoints were determined via HPLC and the fraction of *Z* isomer was plotted against the time. Mono-exponential fitting yielded the rate constants of isomerization *k* ± S.E.. Corresponding thermal half-lives $t_{1/2}(\Delta T)$ ± S.E. were deduced from the respective *k* ± S.E. values.

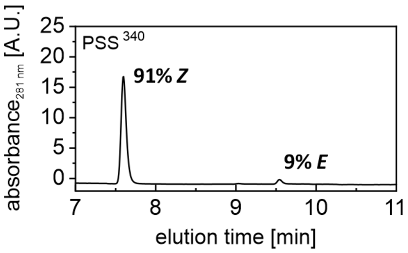

Figure S9. HPLC chromatogram of 20 µM **HAapF** in 50 mM Tris/HCl pH 7.5 to determine the isomer distribution in PSS^{340}. The absorbance was monitored at 281 nm and the isomer ratio was calculated from the peak areas. Irradiation at even shorter wavelengths (340 nm) induced a PSS with higher amount of *Z* isomer than observed for irradiation with 365 nm, however, it is not suited for most biological applications.

Table S4. Isosbestic points of psUAAs in 50 mM Tris/HCl pH 7.5 and DMSO.

psUAA	Tris/HCl	DMSO
**AzoF**	**278 nm**	**279 nm**
	391 nm	386 nm
**NH_2AzoF**	**285 nm**	**291 nm**
	525 nm	539 nm
**MeAapF**	**290 nm**	**291 nm**
	404 nm	402 nm
**HAapF**	**281 nm**	**284 nm**
	383 nm	393 nm
**AatF**	**272 nm**	**275 nm**
	456 nm	464 nm
**HtiF**	312 nm	**363 nm**
	373 nm	454 nm
	**471 nm**	
**SpiroY**	**306 nm**	**308 nm**

Isosbestic points were determined from the respective UV/Vis spectra in 50 mM Tris/HCl pH 7.5 or DMSO (**Figure S4** and **Figure S5**). Isosbestic points that were used for HPLC measurements are marked in bold.

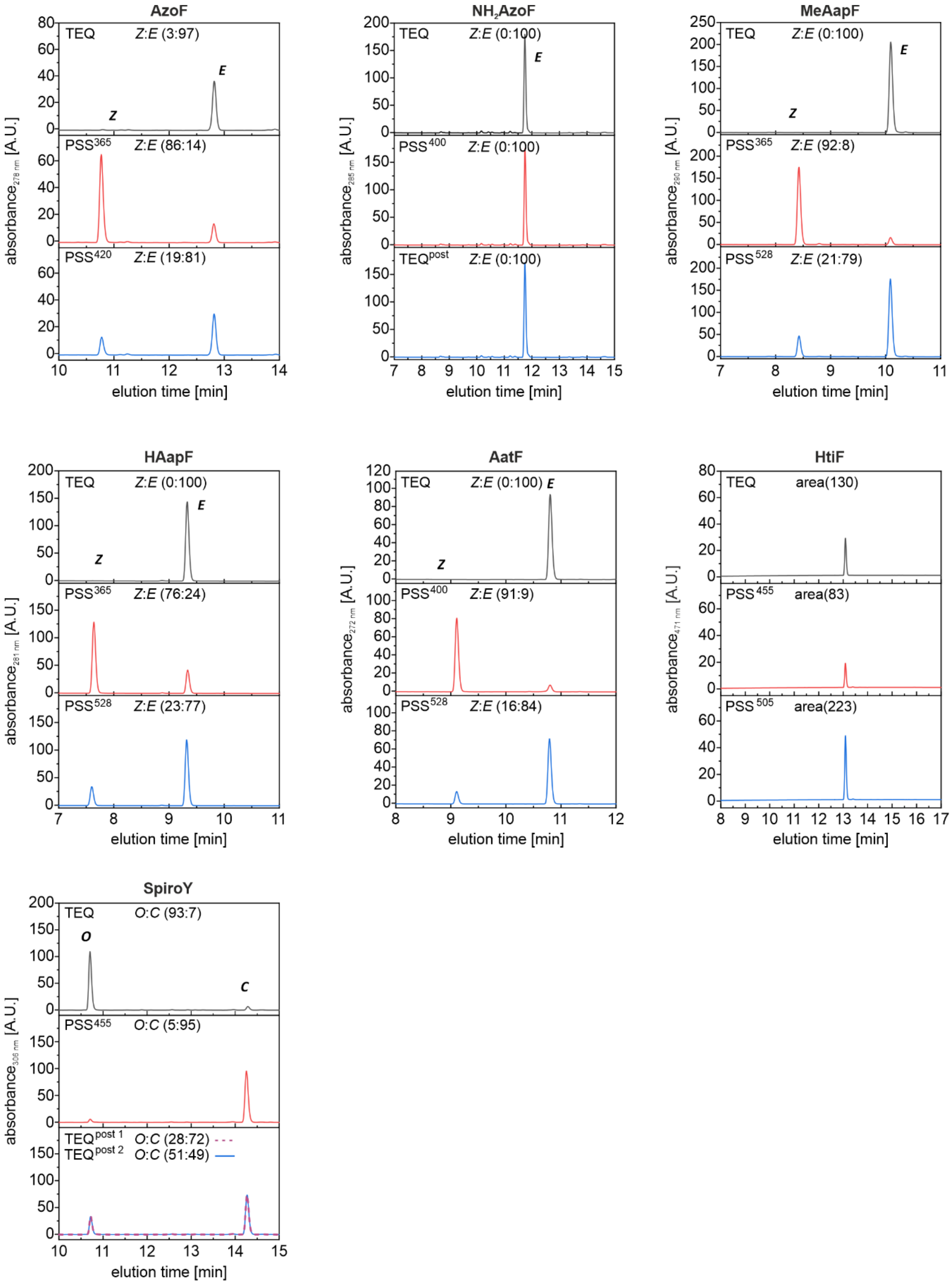

Figure S10. HPLC chromatograms of each psUAA in 50 mM Tris/HCl pH 7.5. The absorbance was monitored at certain isosbestic points (**Table S4**). Measurements of 400 µM **AzoF**, **NH_2AzoF**, **MeAapF**, **HAapF**, **AatF**, as well as 400 µM **HtiF**, and 400 µM **SpiroY** were performed in the thermal equilibrium (TEQ), after irradiation with the selected wavelengths (PSS^{λ}, **Table S2**), or after thermal relaxation (TEQ^{post}). The samples were irradiated right before the HPLC measurement and the column thermostat was set to 15 °C to prolong thermal relaxation of **NH_2AzoF**, **HtiF**, and **SpiroY**. Isomer ratios of each psUAA were determined by integration of the respective isomer peaks. For **NH_2AzoF**, thermal relaxation was still too fast and thus, 100% of the *E* isomer were regenerated before the measurement was finished. Only one peak was visible for **HtiF**, as well. However, we ruled out fast thermal relaxation, because the peak areas in TEQ, PSS^{455}, and PSS^{505} mismatched.

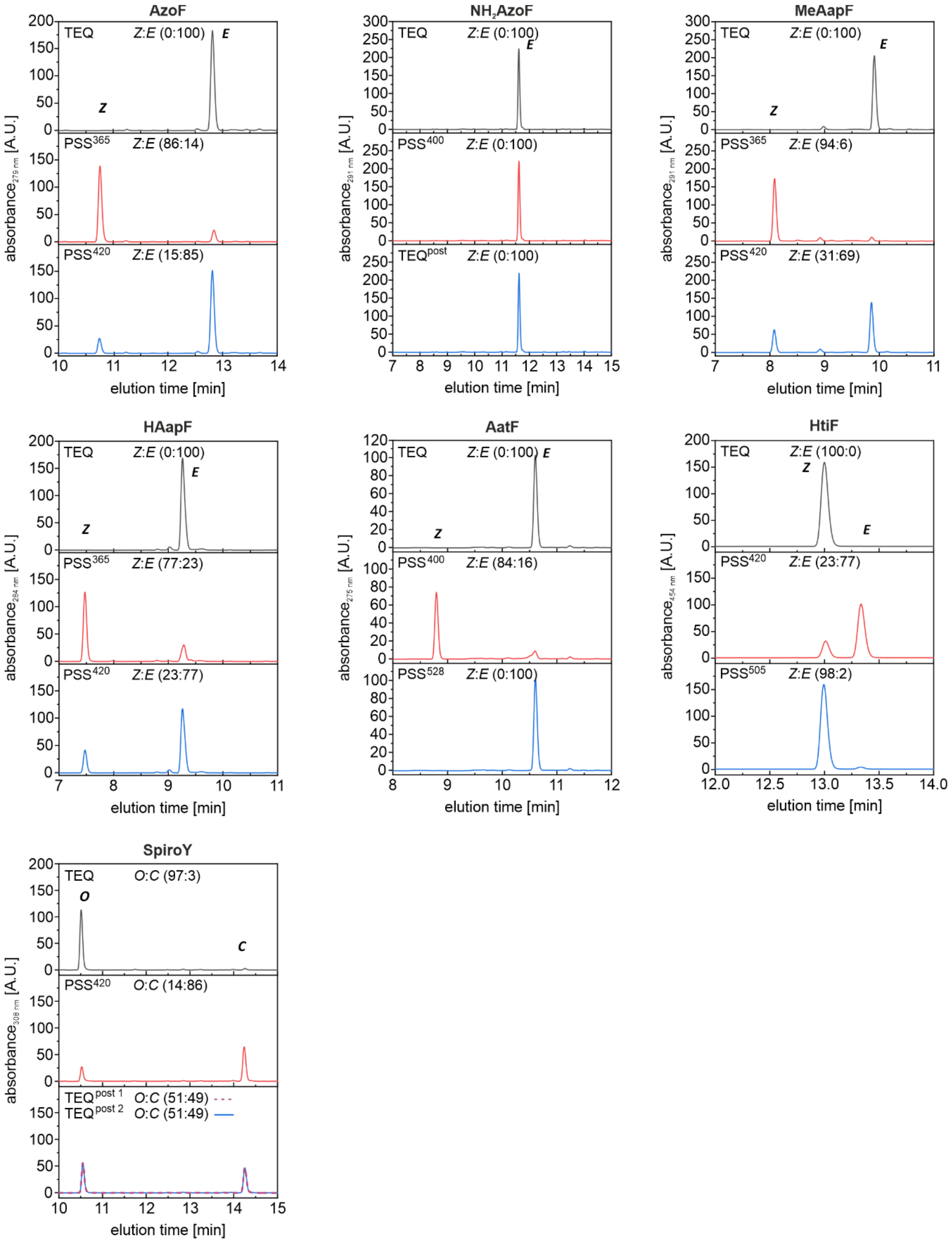

Figure S11. HPLC chromatograms of each psUAA in DMSO. The absorbance was monitored at certain isosbestic points (**Table S4**). Measurements of 400 µM AzoF, NH_2AzoF, MeAapF, HAapF, AatF, as well as 400 µM HtiF, and 400 µM SpiroY were performed in the thermal equilibrium (TEQ), after irradiation with the selected wavelengths (PSS^{λ}, **Table S3**) or after thermal relaxation (TEQ^{post}). The samples were irradiated right before HPLC measurement. Isomer ratios of each psUAA were determined by integration of the respective isomer peaks. For **NH_2AzoF**, thermal relaxation was really fast and thus, 100% of the *E* isomer were regenerated before the measurement was finished.

Table S5. Determination of isomer ratios at the established PSS$^{\lambda}$ after irradiation or thermal isomerization in 50 mM Tris/HCl pH 7.5 determined by HPLC and UV/Vis measurements.

UAA		HPLC		UV/Vis	
		E	*Z*	*E*	*Z*
**AzoF**	TEQ	96.6	3.40	100	0.00
	PSS^{365}	13.9	86.1	15.0	85.0
	PSS^{420}	80.7	19.3	79.0	21.0
**NH_2AzoF**	TEQ	100	0.00	100	0.00
	PSS^{400}	–[a]	–[a]	45.5	54.5
	TEQ^{post}	100	0.00	100	0.00
**MeAapF**	TEQ	100	0.00	100	0.00
	PSS^{365}	8.3	91.7	9.50	90.5
	PSS^{420}	63.0	37.0	70.8	29.2
	PSS^{528}	79.3	20.7	77.3	22.7
**HAapF**	TEQ	100	0.00	100	0.00
	PSS^{365}	24.2	75.8	24.0	76.0
	PSS^{505}	73.0	27.0	61.3	38.7
	PSS^{528}	77.3	22.7	77.5	22.5
**AatF**	TEQ	100	0.00	100	0.00
	PSS^{400}	9.10	90.9	8.90	91.1
	PSS^{528}	84.2	15.8	82.5	17.5
**HtiF**	TEQ	–[b]	–[b]	–[c]	–[c]
	PSS^{455}	–[b]	–[b]	–[c]	–[c]
	PSS^{528}	–[b]	–[b]	–[c]	–[c]
		O	*C*	*O*	*C*
**SpiroY**	TEQ	93.4	6.60	100	0.00
	PSS^{455}	5.40	94.6	3.50	96.5
	TEQ$^{post\ 1}$	28.4	71.6	33.8	66.2
	TEQ$^{post\ 2}$	28.4	71.6	33.8	66.2

The isomer ratios of each psUAA were determined in the thermal equilibrium (TEQ), as well as after irradiation with the selected wavelengths (PSS$^{\lambda}$, **Table S2**) or after thermal relaxation (TEQ^{post}). [a] *E*:*Z* ratio could not be determined since thermal isomerization was faster than the HPLC measurement. [b] isomer ratio could not be determined since only one isomer was visible in HPLC measurements. [c] *E*:*Z* ratio could not be determined since there is no method available.

Table S6. Determination of Isomer ratios at the established PSS$^{\lambda}$ after irradiation or thermal isomerization in DMSO determined by HPLC and UV/Vis measurements.

UAA		HPLC		UV/Vis	
		E	*Z*	*E*	*Z*
**AzoF**	TEQ	100	0	100	0.00
	PSS^{365}	13.7	86.3	5.40	94.6
	PSS^{420}	85.2	14.8	82.4	17.6
**NH_2AzoF**	TEQ	100	0.00	100	0.00
	PSS^{400}	–[a]	–[a]	37.2	62.8
	TEQ^{post}	100	0.00	99.3	0.70
**MeAapF**	TEQ	100	0.00	100	0.00
	PSS^{365}	6.00	94.0	4.30	95.7
	PSS^{420}	69.0	31.0	63.9	36.1
**HAapF**	TEQ	100	0.00	100	0.00
	PSS^{365}	23.1	76.9	13.3	86.7
	PSS^{420}	76.9	23.1	69.6	30.4
**AatF**	TEQ	100	0.00	100	0.00
	PSS^{400}	16.4	83.6	5.8	94.2
	PSS^{528}	100	0.00	81.8	18.2
**HtiF**	TEQ	0.00	100	–[b]	–[b]
	PSS^{420}	76.8	23.2	–[b]	–[b]
	PSS^{505}	2.40	97.6	–[b]	–[b]
		O	*C*	*O*	*C*
**SpiroY**	TEQ	97.2	2.80	100	0.00
	PSS^{420}	14.0	86.0	1.90	98.1
	TEQ$^{post\ 1}$	50.6	49.4	–[c]	–[c]
	TEQ$^{post\ 2}$	50.6	49.4	–[c]	–[c]

The isomer ratios of each psUAA were determined in the thermal equilibrium (TEQ), after irradiation with the selected wavelengths (PSS$^{\lambda}$, **Table S3**) or after thermal relaxation (TEQ^{post}). [a] *E*:*Z* ratio could not be determined since thermal isomerization was faster than the HPLC measurement. [b] *E*:*Z* ratio could not be determined since there is no method available. [c] *E*:*Z* ratio was not determined owing to the slow establishment of TEQ^{post1} and TEQ^{post2} (**Figure S3**).

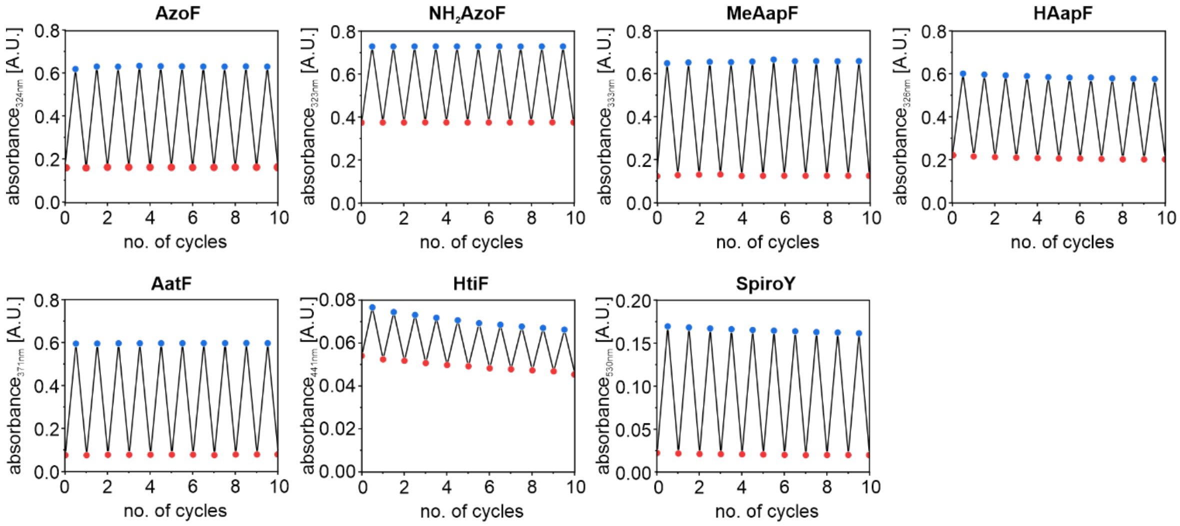

Figure S12. Cycle performance of the photoisomerization of each psUAA in 50 mM Tris/HCl pH 7.5. Alternating isomerization between PSS^{365} and PSS^{420} for **AzoF**, PSS^{385} and TEQ^{post} for **NH_2AzoF**, PSS^{365} and PSS^{528} for **MeAapF**, PSS^{365} and PSS^{528} for **HAapF**, PSS^{400} and PSS^{528} for **AatF**, PSS^{455} and PSS^{528} for **HtiF**, and PSS^{455} and $TEQ^{post\,2}$ for **SpiroY**. The absorbance at a characteristic maximum was plotted against the number of cycles (switching to *E* and subsequently to *Z* or inversely represents one cycle).

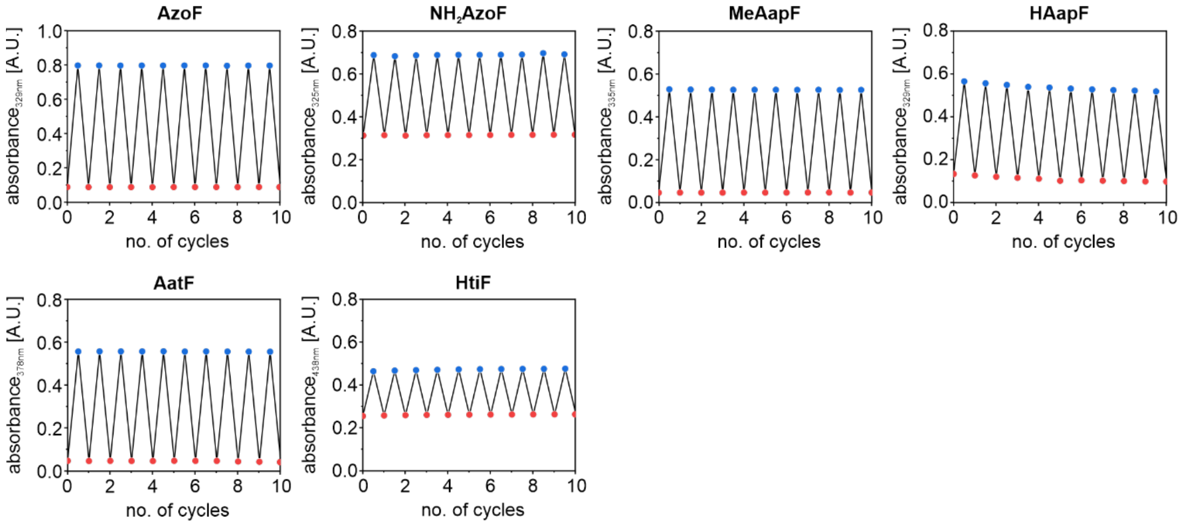

Figure S13. Cycle performance of the photoisomerization of each psUAA in DMSO. Alternating isomerization between PSS^{365} and PSS^{420} for **AzoF**, PSS^{420} and TEQ^{post} for **NH_2AzoF**, PSS^{365} and PSS^{420} for **MeAapF**, PSS^{365} and PSS^{420} for **HAapF**, PSS^{400} and PSS^{528} for **AatF**, and PSS^{420} and PSS^{505} for **HtiF**. The absorbance at a characteristic maximum was plotted against the number of cycles (switching to *E* and subsequently to *Z* or inversely represents one cycle). For **SpiroY**, evaluation of cycle performance was not feasible because there was no wavelength found that could induce ring-opening to switch back towards thermal equilibrium and the thermal half-life was rather long in DMSO.

Table S7. Identities and sequences of aaRSs tested for the incorporation of psUAAs.

aaRS	aaRS	sequence
a	AzoF-RS[1]	MDEFEMIKRNTSEIISEEELREVLKKDEKSAGIGFEPSGKIHLGHYLQIKKMIDLQNAGFDIIIELA<br>DLHAYLNQKGELDEIRKIGDYNKKVFEAMGLKAKYVYGSEAELDKDYTLNVYRLALKTTLKRA<br>RRSMELIAREDENPKVAEVIYPIMQVNGIHYHGVDVAVGGMEQRKIHMLARELLPKKVVCIHN<br>PVLTGLDGEGKMSSSKGNFIAVDDSPEEIRAKIKKAYCPAGVVEGNPIMEIAKYFLEYPLTIKRP<br>EKFGGDLTVNSYEELESLFKNKELHPMRLKNAVAEELIKILEPIRKRL
b	AzoF-RS$_{copt}$[a]	MDEFEMIKRNTSEIISEEELREVLKKDEKSAGIGFEPSGKIHLGHYLQIKKMIDLQNAGFDIIIELA<br>DLHAYLNQKGELDEIRKIGDYNKKVFEAMGLKAKYVYGSEAELDKDYTLNVYRLALKTTLKRA<br>RRSMELIAREDENPKVAEVIYPIMQVNGIHYHGVDVAVGGMEQRKIHMLARELLPKKVVCIHN<br>PVLTGLDGEGKMSSSKGNFIAVDDSPEEIRAKIKKAYCPAGVVEGNPIMEIAKYFLEYPLTIKGP<br>EKFGGDLTVNSYEELESLFKNKELHPMRLKNAVAEELIKILEPIRKRL
c	ONBY-RS$_i$[28]	MDEFEMIKRNTSEIISEEELREVLKKDEKSAAIGFEPSGKIHLGHYLQIKKMIDLQNAGFDIIIALA<br>DLNAYLNQKGELDEIRKIGDYNKKVFEAMGLKAKYVYQSEFALDKDYTLNVYRLALKTTLKRA<br>RRSMELIAREDENPKVAEVIYPIMQVNSAHYAGVDVSVGGMEQRKIHMLQRELLPKKVVCIHN<br>PVLTGLDGEGKMSSSKGNFIAVDDSPEEIRAKIKKAYCPAGVVEGNPIMEIAKYFLEYPLTIKRP<br>EKFGGDLTVNSYEELESLFKNKELHPMRLKNAVAEELIKILEPIRKRL
d	ONBY-RS$_i$ _R257G	MDEFEMIKRNTSEIISEEELREVLKKDEKSAAIGFEPSGKIHLGHYLQIKKMIDLQNAGFDIIIALA<br>DLNAYLNQKGELDEIRKIGDYNKKVFEAMGLKAKYVYQSEFALDKDYTLNVYRLALKTTLKRA<br>RRSMELIAREDENPKVAEVIYPIMQVNSAHYAGVDVSVGGMEQRKIHMLQRELLPKKVVCIHN<br>PVLTGLDGEGKMSSSKGNFIAVDDSPEEIRAKIKKAYCPAGVVEGNPIMEIAKYFLEYPLTIKGP<br>EKFGGDLTVNSYEELESLFKNKELHPMRLKNAVAEELIKILEPIRKRL
e	AzF-RS[29]	MDEFEMIKRNTSEIISEEELREVLKKDEKSATIGFEPSGKIHLGHYLQIKKMIDLQNAGFDIIILLA<br>DLHAYLNQKGELDEIRKIGDYNKKVFEAMGLKAKYVYGSNFQLDKDYTLNVYRLALKTTLKRA<br>RRSMELIAREDENPKVAEVIYPIMQVNPLHYQGVDVAVGGMEQRKIHMLARELLPKKVVCIHN<br>PVLTGLDGEGKMSSSKGNFIAVDDSPEEIRAKIKKAYCPAGVVEGNPIMEIAKYFLEYPLTIKRP<br>EKFGGDLTVNSYEELESLFKNKELHPMRLKNAVAEELIKILEPIRKRL
f	Azo-RS 1[30]	MDEFEMIKRNTSEIISEEELREVLKKDEKSAGIGFEPSGKIHLGHYLQIKKMIDLQNAGFDIIIVLA<br>DLHAYLNQKGELDEIRKIGDYNKKVFEAMGLKAKYVYGSEFQLDKDYTLNVYRLALKTTLKRA<br>RRSMELIAREDENPKVAEVIYPIMQVNGYHYSGVDVFVGGMEQRKIHMLARELLPKKVVCIHN<br>PVLTGLDGEGKMSSSKGNFIAVDDSPEEIRAKIKKAYCPAGVVEGNPIMEIAKYFLEYPLTIKGP<br>EKFGGDLTVNSYEELESLFKNKELHPMRLKNAVAEELIKILEPIRKRL
g	Azo-RS 4[30]	MDEFEMIKRNTSEIISEEELREVLKKDEKSAGIGFEPSGKIHLGHYLQIKKMIDLQNAGFDIIIVLA<br>DLHAYLNQKGELDEIRKIGDYNKKVFEAMGLKAKYVYGSEFQLDKDYTLNVYRLALKTTLKRA<br>RRSMELIAREDENPKVAEVIYPIMQVNGYHYRGVDVAVGGMEQRKIHMLARELLPKKVVCIHN<br>PVLTGLDGEGKMSSSKGNFIAVDDSPEEIRAKIKKAYCPAGVVEGNPIMEIAKYFLEYPLTIKGP<br>EKFGGDLTVNSYEELESLFKNKELHPMRLKNAVAEELIKILEPIRKRL
h	AapF-RS	MDEFEMIKRNTSEIISEEELREVLKKDEKSAAIGFEPSGKIHLGHYLQIKKMIDLQNAGFDIIILLA<br>DLHAYLNQKGELDEIRKIGDYNKKVFEAMGLKAKYVYGSEFMLDKDYTLNVYRLALKTTLKRA<br>RRSMELIAREDENPKVAEVIYPIMQVNAIHYFGVDVAVGGMEQRKIHMLARELLPKKVVCIHNP<br>VLTGLDGEGKMSSSKGNFIAVDDSPEEIRAKIKKAYCPAGVVEGNPIMEIAKYFLEYPLTIKGPE<br>KFGGDLTVNSYEELESLFKNKELHPMRLKNAVAEELIKILEPIRKRL
i	AzoF-RS$_{copt}$_Q155V	MDEFEMIKRNTSEIISEEELREVLKKDEKSAGIGFEPSGKIHLGHYLQIKKMIDLQNAGFDIIIELA<br>DLHAYLNQKGELDEIRKIGDYNKKVFEAMGLKAKYVYGSEAELDKDYTLNVYRLALKTTLKRA<br>RRSMELIAREDENPKVAEVIYPIMVVNGIHYHGVDVAVGGMEQRKIHMLARELLPKKVVCIHN<br>PVLTGLDGEGKMSSSKGNFIAVDDSPEEIRAKIKKAYCPAGVVEGNPIMEIAKYFLEYPLTIKGP<br>EKFGGDLTVNSYEELESLFKNKELHPMRLKNAVAEELIKILEPIRKRL
j	AzoF-RS$_{copt}$_A167V	MDEFEMIKRNTSEIISEEELREVLKKDEKSAGIGFEPSGKIHLGHYLQIKKMIDLQNAGFDIIIELA<br>DLHAYLNQKGELDEIRKIGDYNKKVFEAMGLKAKYVYGSEAELDKDYTLNVYRLALKTTLKRA<br>RRSMELIAREDENPKVAEVIYPIMQVNGIHYHGVDVVVGGMEQRKIHMLARELLPKKVVCIHN<br>PVLTGLDGEGKMSSSKGNFIAVDDSPEEIRAKIKKAYCPAGVVEGNPIMEIAKYFLEYPLTIKGP<br>EKFGGDLTVNSYEELESLFKNKELHPMRLKNAVAEELIKILEPIRKRL
k	AzoF-RS$_{copt}$_A167C	MDEFEMIKRNTSEIISEEELREVLKKDEKSAGIGFEPSGKIHLGHYLQIKKMIDLQNAGFDIIIELA<br>DLHAYLNQKGELDEIRKIGDYNKKVFEAMGLKAKYVYGSEAELDKDYTLNVYRLALKTTLKRA<br>RRSMELIAREDENPKVAEVIYPIMQVNGIHYHGVDVCVGGMEQRKIHMLARELLPKKVVCIHN<br>PVLTGLDGEGKMSSSKGNFIAVDDSPEEIRAKIKKAYCPAGVVEGNPIMEIAKYFLEYPLTIKGP<br>EKFGGDLTVNSYEELESLFKNKELHPMRLKNAVAEELIKILEPIRKRL
l	AzoF-RS$_{copt}$_G32A	MDEFEMIKRNTSEIISEEELREVLKKDEKSAAIGFEPSGKIHLGHYLQIKKMIDLQNAGFDIIIELA<br>DLHAYLNQKGELDEIRKIGDYNKKVFEAMGLKAKYVYGSEAELDKDYTLNVYRLALKTTLKRA<br>RRSMELIAREDENPKVAEVIYPIMQVNGIHYHGVDVAVGGMEQRKIHMLARELLPKKVVCIHN<br>PVLTGLDGEGKMSSSKGNFIAVDDSPEEIRAKIKKAYCPAGVVEGNPIMEIAKYFLEYPLTIKGP<br>EKFGGDLTVNSYEELESLFKNKELHPMRLKNAVAEELIKILEPIRKRL
m	AzoF-RS$_{copt}$_E65L	MDEFEMIKRNTSEIISEEELREVLKKDEKSAGIGFEPSGKIHLGHYLQIKKMIDLQNAGFDIIILLA<br>DLHAYLNQKGELDEIRKIGDYNKKVFEAMGLKAKYVYGSEAELDKDYTLNVYRLALKTTLKRA<br>RRSMELIAREDENPKVAEVIYPIMQVNGIHYHGVDVAVGGMEQRKIHMLARELLPKKVVCIHN<br>PVLTGLDGEGKMSSSKGNFIAVDDSPEEIRAKIKKAYCPAGVVEGNPIMEIAKYFLEYPLTIKGP<br>EKFGGDLTVNSYEELESLFKNKELHPMRLKNAVAEELIKILEPIRKRL

n	AzoF-RS$_{copt}$_V188P	MDEFEMIKRNTSEIISEEELREVLKKDEKSAGIGFEPSGKIHLGHYLQIKKMIDLQNAGFDIIELA<br>DLHAYLNQKGELDEIRKIGDYNKKVFEAMGLKAKYVYGSEAELDKDYTLNVYRLALKTTLKRA<br>RRSMELIAREDENPKVAEVIYPIMQVNGIHYHGVDVAVGGMEQRKIHMLARELLPKKPVCIHN<br>PVLTGLDGEGKMSSSKGNFIAVDDSPEEIRAKIKKAYCPAGVVEGNPIMEIAKYFLEYPLTIKGP<br>EKFGGDLTVNSYEELESLFKNKELHPMRLKNAVAEELIKILEPIRKRL
o	AzoF-RS$_{copt}$_A108F	MDEFEMIKRNTSEIISEEELREVLKKDEKSAGIGFEPSGKIHLGHYLQIKKMIDLQNAGFDIIELA<br>DLHAYLNQKGELDEIRKIGDYNKKVFEAMGLKAKYVYGSEFELDKDYTLNVYRLALKTTLKRA<br>RRSMELIAREDENPKVAEVIYPIMQVNGIHYHGVDVAVGGMEQRKIHMLARELLPKKVVCIHN<br>PVLTGLDGEGKMSSSKGNFIAVDDSPEEIRAKIKKAYCPAGVVEGNPIMEIAKYFLEYPLTIKGP<br>EKFGGDLTVNSYEELESLFKNKELHPMRLKNAVAEELIKILEPIRKRL
p	AzoF-<br>RS$_{copt}$_E109M	MDEFEMIKRNTSEIISEEELREVLKKDEKSAGIGFEPSGKIHLGHYLQIKKMIDLQNAGFDIIELA<br>DLHAYLNQKGELDEIRKIGDYNKKVFEAMGLKAKYVYGSEAMLDKDYTLNVYRLALKTTLKRA<br>RRSMELIAREDENPKVAEVIYPIMQVNGIHYHGVDVAVGGMEQRKIHMLARELLPKKVVCIHN<br>PVLTGLDGEGKMSSSKGNFIAVDDSPEEIRAKIKKAYCPAGVVEGNPIMEIAKYFLEYPLTIKGP<br>EKFGGDLTVNSYEELESLFKNKELHPMRLKNAVAEELIKILEPIRKRL
q	AzoF-RS$_{copt}$_G158A	MDEFEMIKRNTSEIISEEELREVLKKDEKSAGIGFEPSGKIHLGHYLQIKKMIDLQNAGFDIIELA<br>DLHAYLNQKGELDEIRKIGDYNKKVFEAMGLKAKYVYGSEAELDKDYTLNVYRLALKTTLKRA<br>RRSMELIAREDENPKVAEVIYPIMQVNAIHYHGVDVAVGGMEQRKIHMLARELLPKKVVCIHN<br>PVLTGLDGEGKMSSSKGNFIAVDDSPEEIRAKIKKAYCPAGVVEGNPIMEIAKYFLEYPLTIKGP<br>EKFGGDLTVNSYEELESLFKNKELHPMRLKNAVAEELIKILEPIRKRL
r	AzoF-RS$_{copt}$_H162F	MDEFEMIKRNTSEIISEEELREVLKKDEKSAGIGFEPSGKIHLGHYLQIKKMIDLQNAGFDIIELA<br>DLHAYLNQKGELDEIRKIGDYNKKVFEAMGLKAKYVYGSEAELDKDYTLNVYRLALKTTLKRA<br>RRSMELIAREDENPKVAEVIYPIMQVNGIHYFGVDVAVGGMEQRKIHMLARELLPKKVVCIHN<br>PVLTGLDGEGKMSSSKGNFIAVDDSPEEIRAKIKKAYCPAGVVEGNPIMEIAKYFLEYPLTIKGP<br>EKFGGDLTVNSYEELESLFKNKELHPMRLKNAVAEELIKILEPIRKRL
s	AzoF-RS$_{copt}$_I159Y	MDEFEMIKRNTSEIISEEELREVLKKDEKSAGIGFEPSGKIHLGHYLQIKKMIDLQNAGFDIIELA<br>DLHAYLNQKGELDEIRKIGDYNKKVFEAMGLKAKYVYGSEAELDKDYTLNVYRLALKTTLKRA<br>RRSMELIAREDENPKVAEVIYPIMQVNGYHYHGVDVAVGGMEQRKIHMLARELLPKKVVCIHN<br>PVLTGLDGEGKMSSSKGNFIAVDDSPEEIRAKIKKAYCPAGVVEGNPIMEIAKYFLEYPLTIKGP<br>EKFGGDLTVNSYEELESLFKNKELHPMRLKNAVAEELIKILEPIRKRL
t	AzoF-RS$_{copt}$_I159K	MDEFEMIKRNTSEIISEEELREVLKKDEKSAGIGFEPSGKIHLGHYLQIKKMIDLQNAGFDIIELA<br>DLHAYLNQKGELDEIRKIGDYNKKVFEAMGLKAKYVYGSEAELDKDYTLNVYRLALKTTLKRA<br>RRSMELIAREDENPKVAEVIYPIMQVNGKHYHGVDVAVGGMEQRKIHMLARELLPKKVVCIHN<br>PVLTGLDGEGKMSSSKGNFIAVDDSPEEIRAKIKKAYCPAGVVEGNPIMEIAKYFLEYPLTIKGP<br>EKFGGDLTVNSYEELESLFKNKELHPMRLKNAVAEELIKILEPIRKRL
u	AzoF-RS$_{copt}$_I159F	MDEFEMIKRNTSEIISEEELREVLKKDEKSAGIGFEPSGKIHLGHYLQIKKMIDLQNAGFDIIELA<br>DLHAYLNQKGELDEIRKIGDYNKKVFEAMGLKAKYVYGSEAELDKDYTLNVYRLALKTTLKRA<br>RRSMELIAREDENPKVAEVIYPIMQVNGFHYHGVDVAVGGMEQRKIHMLARELLPKKVVCIHN<br>PVLTGLDGEGKMSSSKGNFIAVDDSPEEIRAKIKKAYCPAGVVEGNPIMEIAKYFLEYPLTIKGP<br>EKFGGDLTVNSYEELESLFKNKELHPMRLKNAVAEELIKILEPIRKRL
v	AzoF-RS$_{copt}$_I159W	MDEFEMIKRNTSEIISEEELREVLKKDEKSAGIGFEPSGKIHLGHYLQIKKMIDLQNAGFDIIELA<br>DLHAYLNQKGELDEIRKIGDYNKKVFEAMGLKAKYVYGSEAELDKDYTLNVYRLALKTTLKRA<br>RRSMELIAREDENPKVAEVIYPIMQVNGWHYHGVDVAVGGMEQRKIHMLARELLPKKVVCIH<br>NPVLTGLDGEGKMSSSKGNFIAVDDSPEEIRAKIKKAYCPAGVVEGNPIMEIAKYFLEYPLTIKG<br>PEKFGGDLTVNSYEELESLFKNKELHPMRLKNAVAEELIKILEPIRKRL

[a] aaRS-b is a codon-optimized version (see **Table S8**) of aaRS-a harboring the R257G mutation that improves tRNA binding.[30]

Table S8. DNA sequence of the codon-optimized AzoF-RS$_{copt}$ (aaRS-b).

AzoF-RS$_{copt}$	AAAAAGGTCTCACATGGATGAGTTCGAAATGATTAAACGTAACACCAGCGAAATCATCAGCGAAGAAGAACTGCGCGAAGT<br>ACTGAAAAAGGATGAAAAATCAGCCGGTATTGGTTTTGAACCGAGCGGTAAAATTCATCTGGGTCATTATCTGCAAATCAAA<br>AAGATGATCGATCTGCAGAATGCCGGTTTCGATATTATCATTGAACTGGCCGATCTGCATGCATATCTGAATCAGAAAGGTG<br>AACTGGATGAAATTCGCAAAATCGGCGATTACAACAAAAAGGTGTTTGAAGCAATGGGCCTGAAAGCCAAATATGTTTATGG<br>TAGCGAAGCCGAGCTGGATAAAGATTATACCCTGAATGTTTATCGTCTGGCCCTGAAAACCACACTGAAACGTGCCCGTCG<br>TAGCATGGAACTGATTGCACGTGAAGATGAAAATCCGAAAGTTGCCGAAGTGATTTATCCGATTATGCAGGTTAACGGCATT<br>CATTATCATGGTGTTGATGTTGCAGTTGGTGGTATGGAACAGCGCAAAATTCATATGCTGGCACGTGAACTGCTGCCGAAG<br>AAAGTTGTTTGTATTCATAATCCGGTTCTGACCGGTCTGGATGGTGAAGGCAAAATGAGCAGCAGCAAAGGTAACTTTATTG<br>CCGTTGATGATAGTCCGGAAGAAATTCGTGCCAAAATCAAGAAAGCATATTGTCCGGCAGGCGTTGTTGAAGGTAACCCGA<br>TTATGGAAATTGCCAAATACTTTCTGGAATACCCGCTGACCATTAAAGGACCGGAAAAATTTGGTGGTGATCTGACCGTTAA<br>TAGCTATGAAGAACTGGAAAGCCTGTTTAAAAACAAAGAACTGCATCCGATGCGTCTGAAAAATGCAGTTGCGGAAGAACT<br>GATTAAAATCCTGGAACCGATTCGTAAACGTCTGCTCGAGAGACCAAAAA

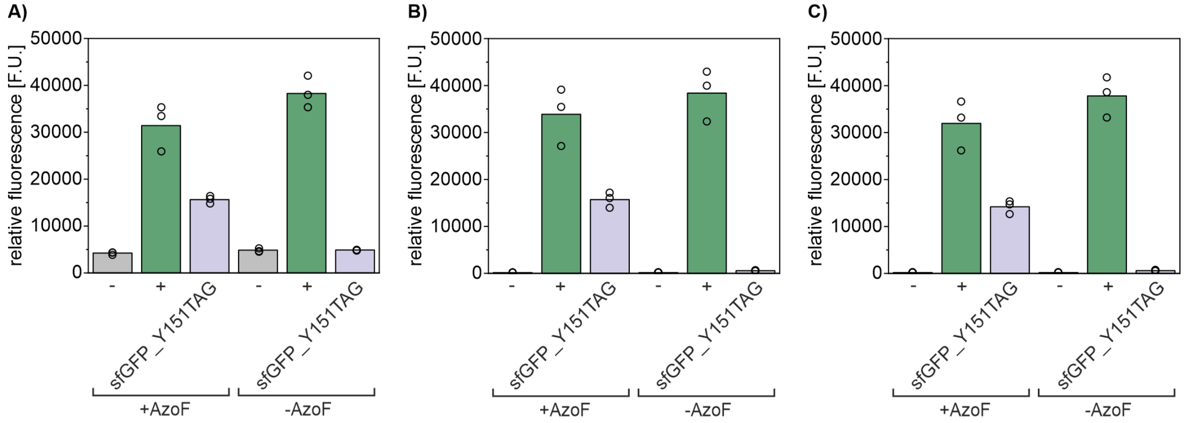

Figure S14. Optimization of the aaRS screening procedure. Different conditions for the treatment of the cells before sfGFP measurement were investigated. Relative fluorescence was measured right after expression in the presence and absence of **AzoF** overnight (A), after subsequent centrifugation and resuspension in 50 mM Tris/HCl pH 7.5 (B), and after additional cell lysis with 1x FastBreak^{TM} Cell Lysis Reagent (C). The empty vector pETBAD (corresponding to a former pET24a vector where the arabinose inducible pBAD promoter was inserted) in combination with pGLNS_AzoF-RS$_{copt}$ served as negative control (−), pETBAD_sfGFP_wt in combination with pGLNS_AzoF-RS$_{copt}$ as positive control (+). The sfGFP_Y151TAG gene was co-expressed with pGLNS_AzoF-RS$_{copt}$. Bars represent the mean of biological triplicates with the single values depicted as circles. Depending on the treatment, significant differences in the background fluorescence were observed. At least cell lysis (B) was necessary to sufficiently reduce background fluorescence in the absence of **AzoF**.

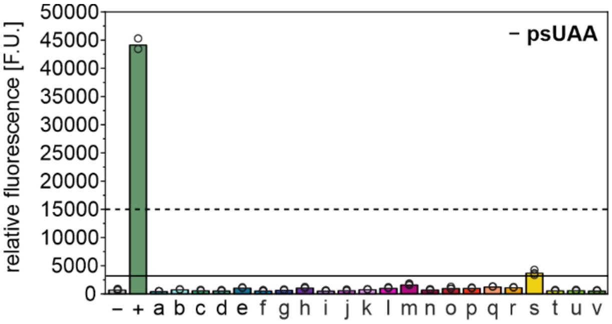

Figure S15. Evaluation of misincorporation of natural amino acids in the absence of a psUAA with *Mj*Tyr-RS variants a–v. Orthogonality could be confirmed for each aaRS except aaRS-s. Empty circles: fluorescence intensities of three biological replicates; columns: mean fluorescence intensity; solid line: lower threshold corresponding to the fluorescence intensity with the original AzoF-RS (aaRS-a); dashed line: upper threshold corresponding to ~30% of the wild type sfGFP fluorescence signal (**Figure 4A**).

Table S9. Successful psUAA-aaRS combinations found in the synthetase screening.

psUAA to be incorporated	suitable synthetase as identified from screening
**AzoF**	aaRS-g in pGLNS
**NH_2AzoF**	aaRS-g in pGLNS
**MeAapF**	aaRS-p in pGLNS
**HAapF**	aaRS-g in pEVOL
**AatF**	aaRS-q in pGLNS
**HtiF**	aaRS-g in pGLNS

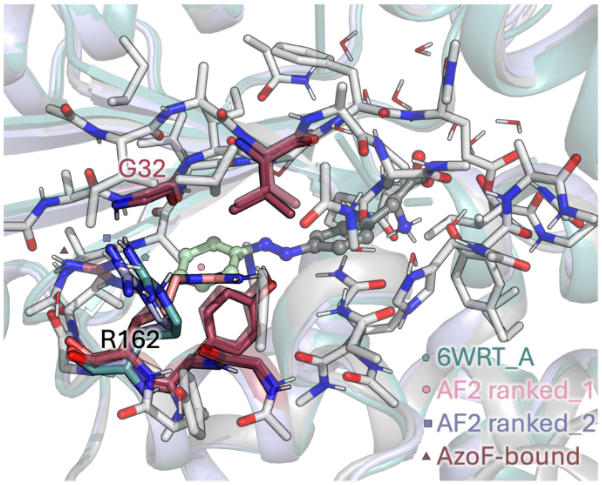

Figure S16. Optimized cluster model of aaRS-g with **AzoF** bound in the active site (**AzoF** is shown in gray and green spheres and sticks, whereas the protein residues in gray and raspberry sticks) showing R162 conformation pointing outside the pocket to allow **AzoF** binding. This conformation of R162 has also been reported crystallographically (shown in teal sticks, PDB: 6WRT). AF2 predicts the two possible conformations of R162: model ranked 1 positions R162 in the space left by the Y32G mutation, whereas model ranked 2 places R162 outside the pocket similarly to the conformation adopted in 6WRT crystal structure.

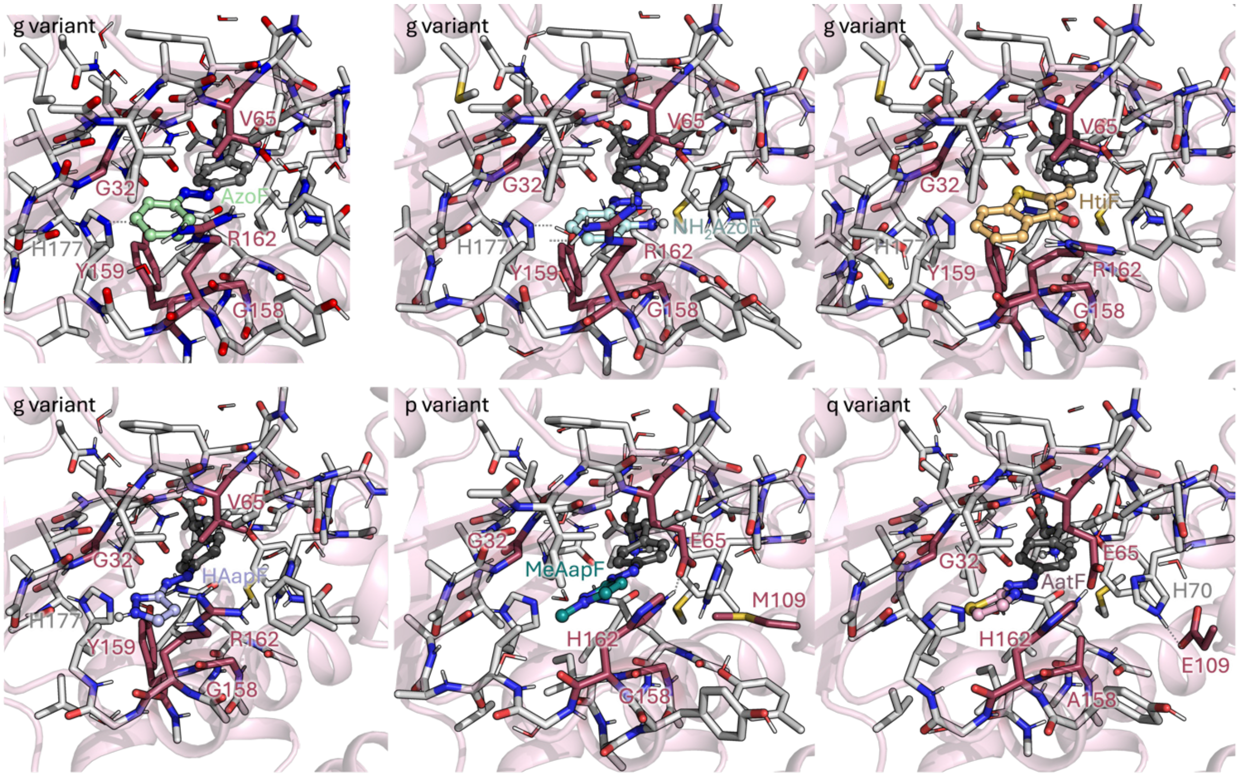

Figure S17. Optimized cluster models of aaRS-g, -p, and -q. The optimized geometry of aaRS-g containing **AzoF** (top left, shown in gray and green spheres), **NH$_2$AzoF** (top middle, in gray and cyan), **HtiF** (top right, in gray and gold), and **HAapF** (bottom left, in gray and light blue). The optimized structure of aaRS-p containing **MeAapF** (in gray and teal) bound in the pocket is shown in the bottom middle, and the aaRS-q containing **AatF** (in gray and pink) in the bottom right. All mutations are colored in raspberry and labeled.

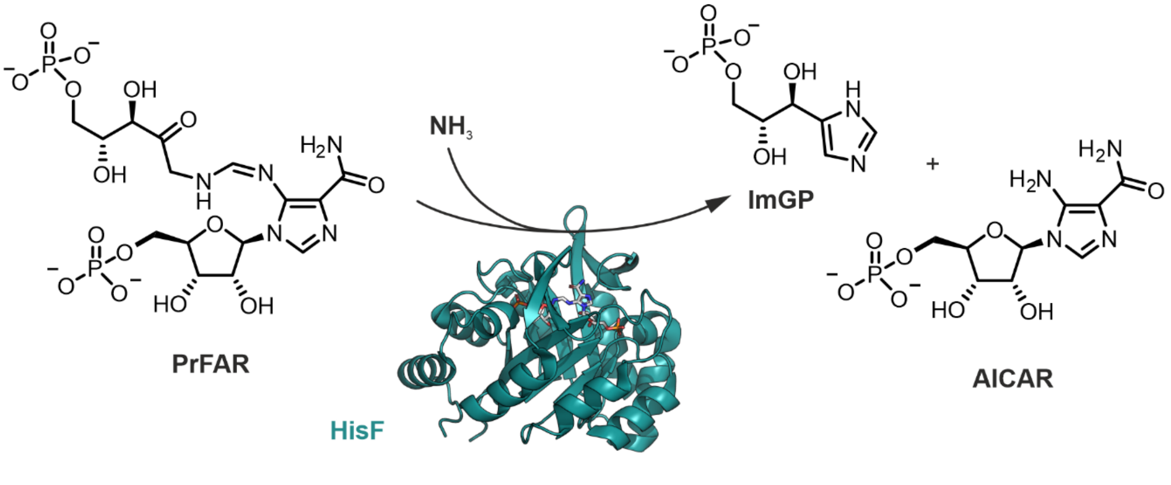

Figure S18. Reaction catalyzed by HisF (PDB: 7ac8). Turnover of *N'*-[(5'-phosphoribulosyl)formimino]-5-amino-imidazole-4-carboxamide ribonucleotide (PrFAR) and NH_3 to yield imidazole glycerol phosphate (ImGP) and 5-aminoimidazol-4-carboxamidribotide (AICAR).

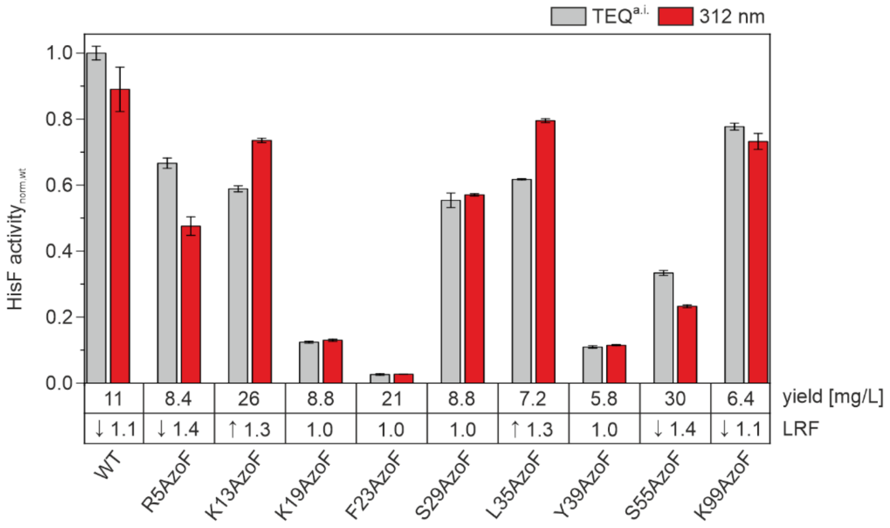

Figure S19. Screening of HisF-AzoF variants comparing their activities in the as-isolated, thermally equilibrated state (TEQ$^{a.i.}$, grey) and after irradiation with 312 nm in their PSS^{312}. Expression yields in mg per liter expression medium and the LRF values upon irradiation are given for each variant below the bar graph. Reactions were prepared in a 96-well plate at 25 °C and measured in a Tecan Infinite M200 Pro. Reaction conditions: 50 mM Tris acetate pH 8.5, 100 mM ammonium acetate, 1 µM HisA (to convert ProFAR to PrFAR), 70 µM ProFAR (saturation) and 0.2 µM HisF (reaction start). Activities of technical triplicates were determined in µM min^{-1} as mean ± standard error of mean and normalized to the activity of wildtype HisF. WT: wildtype HisF.

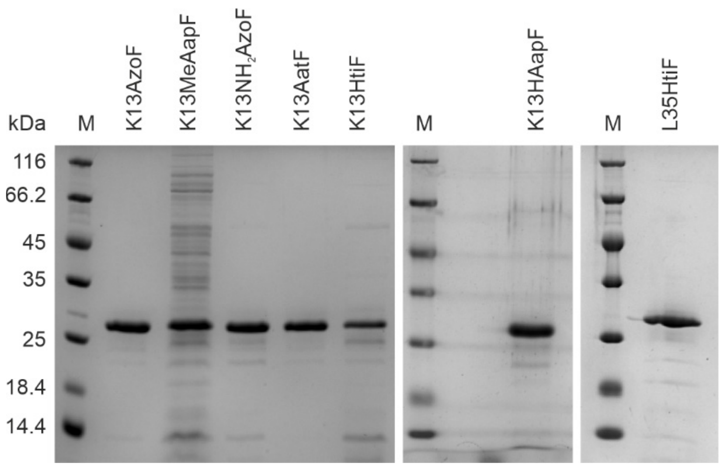

Figure S20. SDS-PAGE of the HisF-psUAA variants. 7 µL low-molecular weight marker (M) and 3 µg of each protein were applied to the gel. The variants showed purities of >95% (K13AzoF), >70% (K13MeAapF), >90% (K13NH$_2$AzoF), >95% (K13AatF), >50% (K13HtiF), >95% (K13HAapF), and >95% (L35HtiF, 13 mg/L expression culture).

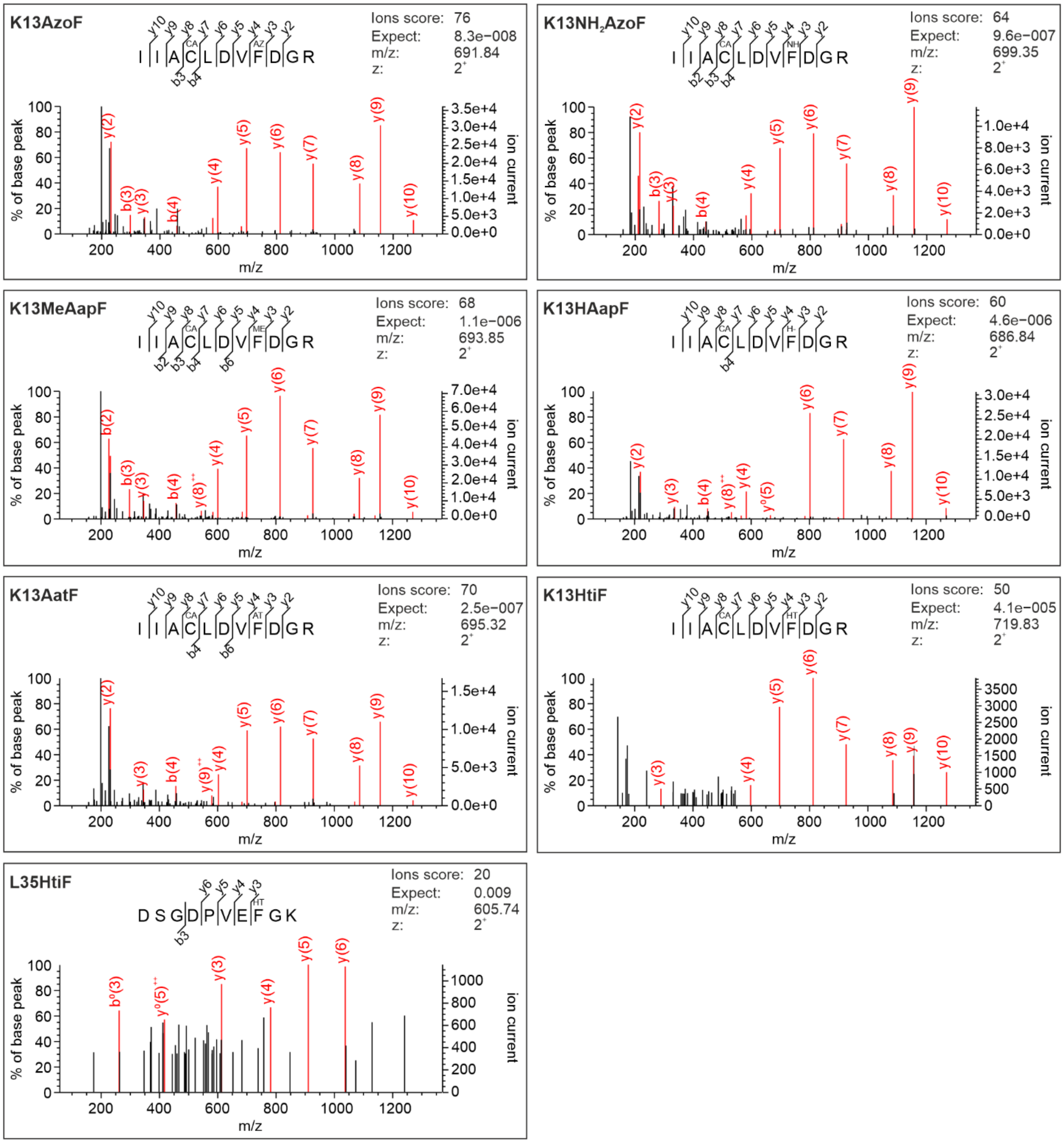

Figure S21. Confirmation of correct incorporation of psUAAs in HisF from *Thermotoga maritima* by LC-ESI-MS/MS. Shown are the fragment spectra of tryptic peptides containing **AzoF** (AZ), **NH2AzoF** (NH), **MeAapF** (ME), **HAapF** (H-), **AatF** (AT), and **HtiF** (HT). CA: carbamidomethyl.

Table S10. Absorbance maxima λ_{max} of each psUAA in 50 mM Tris/HCl pH 7.5 and DMSO.

psUAA	isomer	λ_{max}[a] [nm]	λ_{max}[b] [nm]
**K13AzoF**	*E*	333	425
	Z	—[c]	426
**K13NH$_2$AzoF**	*E*	~333	~420
	Z	~333	~420
**K13MeAapF**	*E*	333	~420
	Z	—[c]	~420
**K13HAapF**	*E*	331	419
	Z	—[c]	420
**K13AatF**	*E*	375	—[d]
	Z	326	463
**K13HtiF**	*E*	~335	~448
	Z	~335	~448

[a] $\pi\rightarrow\pi^*$ transition in case of diazo compounds. [b] $n\rightarrow\pi^*$ transition in case of diazo compounds. [c] cannot be determined due to overlap with protein signal [d] no spectroscopic signal.

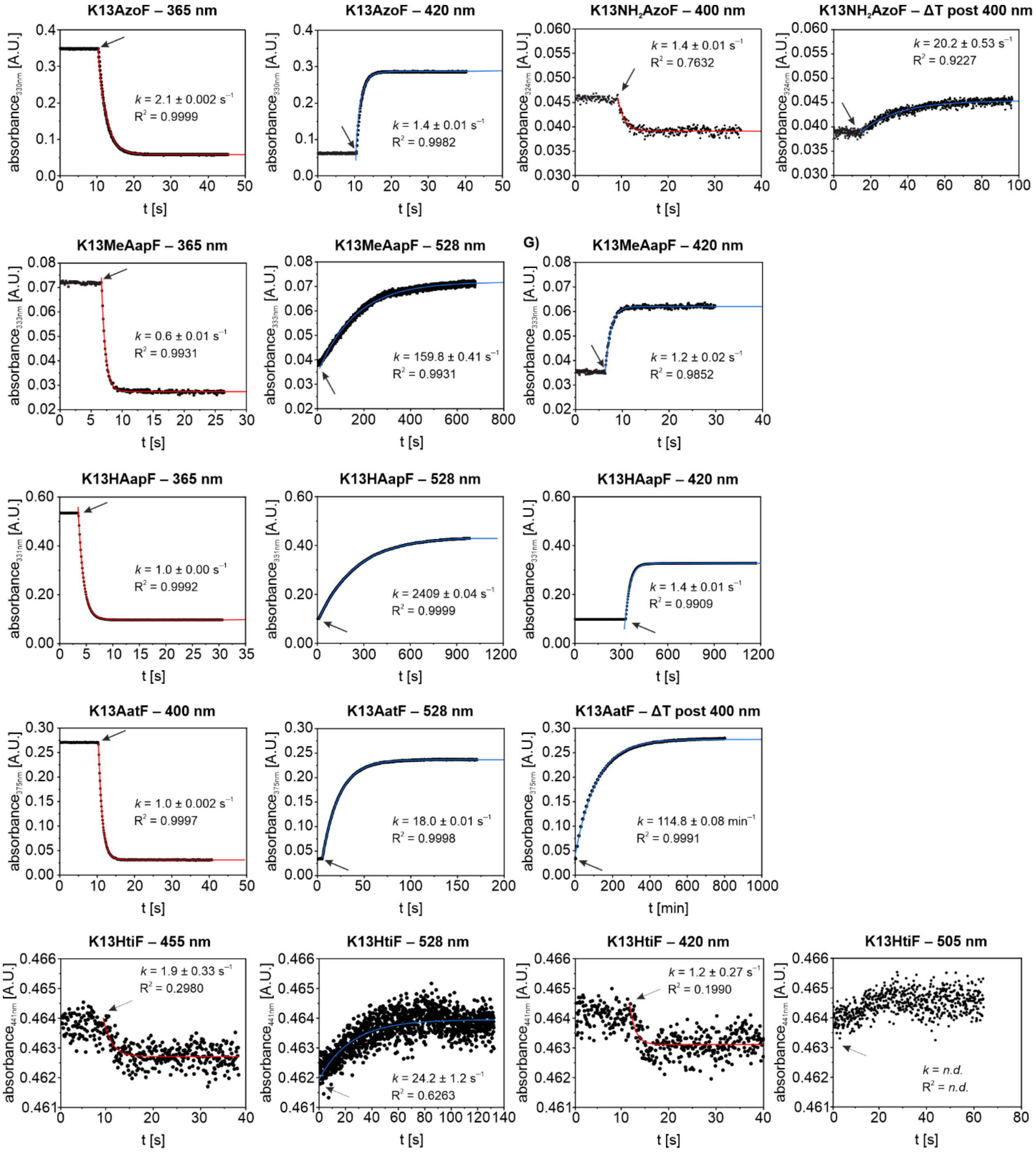

Figure S22. Exemplary isomerization rates of each HisF-psUAA in 50 mM Tris/HCl pH 7.5, 100 mM NaCl upon irradiation with the selected wavelengths or through thermal relaxation (*ΔT*). The absorbance was followed at the π→π* or n→π* transition. Irradiation (arrow) was started and either maintained until a plateau was reached (for evaluation of isomerization upon irradiation) or stopped (for evaluation of thermal relaxation). The rates of isomerization *k* ± S.E. were determined by fitting the data with a mono-exponential function (red). For K13HtiF irradiation with 505 nm, the difference in the absorbance signal was insufficient to determine *k*. Half-lives of isomerization $t_{½}$ ± S.E. were deduced from the respective *k* ± S.E. values and are listed in **Table S11**.

Table S11. Determination of isomerization half-lives ($t_{½}$) for K13psUAA variants in 50 mM Tris/HCl pH 7.5, 100 mM NaCl at 25 °C.

Protein	λ	selected as best wavelength in …[a]	$t_{½}$ [b]
K13AzoF	365 nm	Tris/HCl, DMSO	1.40 ± 0.001 s
	420 nm	Tris/HCl, DMSO	0.90 ± 0.01 s
K13NH_2AzoF	400 nm	Tris/HCl, DMSO	0.90 ± 0.07 s
	ΔT		14.0 ± 0.37 s
K13MeAapF	365 nm	Tris/HCl, DMSO	0.40 ± 0.01 s
	420 nm	DMSO	0.80 ± 0.01 s
	528 nm	Tris/HCl	111 ± 0.28 s
K13HAapF	365 nm	Tris/HCl, DMSO	0.70 ± 0.00 s
	420 nm	DMSO	0.95 ± 0.01 s
	528 nm	Tris/HCl	167.0 ± 0.03 s
K13AatF	400 nm	Tris/HCl, DMSO	0.70 ± 0.001 s
	528 nm	Tris/HCl, DMSO	12.5 ± 0.01 s
	ΔT		79.6 ± 0.06 min
K13HtiF	455 nm	Tris/HCl	1.29 ± 0.23 s
	528 nm	Tris/HCl	16.9 ± 0.83 s
	420 nm	DMSO	0.83 ± 0.19
	505 nm	DMSO	–[c]

[a] cf. **Figure 3** and **Figure S2**. [b] Isomerization half-lives $t_{½}$ ± S.E. induced by irradiation or thermally were derived from the respective isomerization rates k ± S.E. (see **Figure S22**). [c] could not be determined because of insufficient absorbance signal.

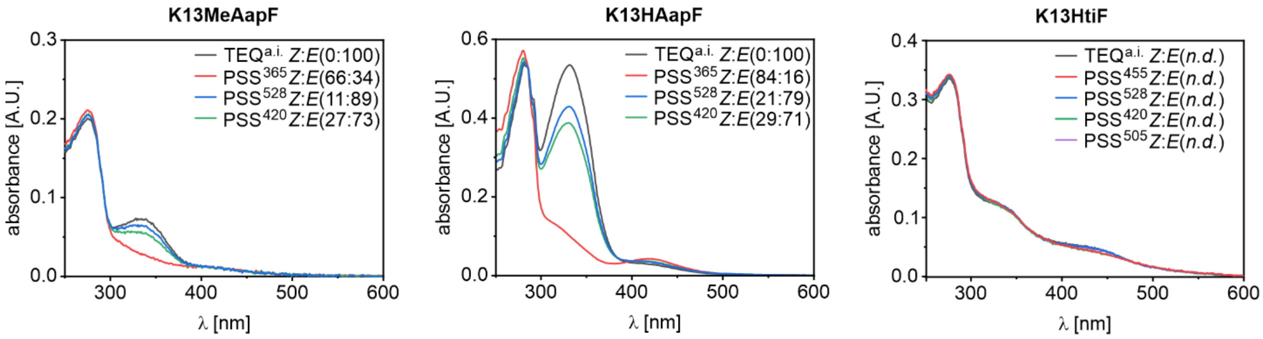

Figure S23. Comparison of the most effective wavelengths for the isomerization of K13MeAapF, K13HAapF, and K13HtiF in 50 mM Tris/HCl pH 7.5, 100 mM NaCl. The isolated psUAAs **MeAapF**, **HAapF**, and **HtiF** showed diverging effective wavelengths in buffer and DMSO (cf. **Figures S4–S5**) and thus, these wavelengths had to be re-evaluated in the protein context.

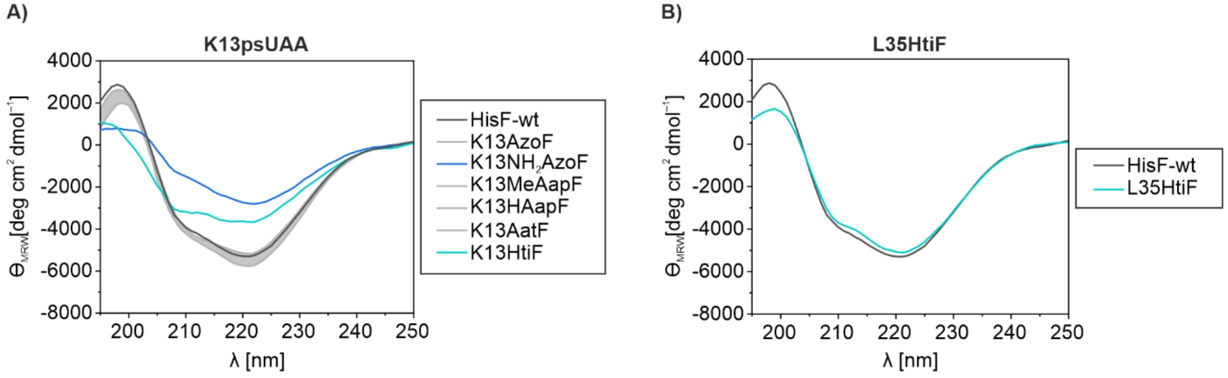

Figure S24. Circular dichroism spectra of K13psUAA variants (A) and L35HtiF (B) in 50 mM Tris/HCl, 100 mM NCl, pH 7.5. to confirm the structural integrity of the enzymes. For the sake of simplicity, essentially similar spectra of K13AzoF, K13MeAapF, K13HAapF, and K13AatF were combined in the grey-shaded area.

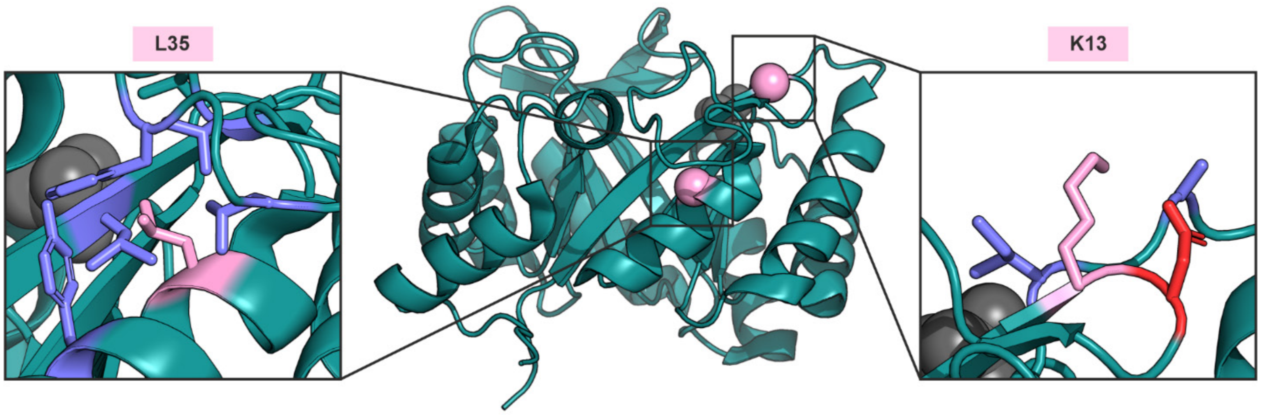

Figure S25. Localization of positions K13 and L35 in HisF (PDB: 7ac8). While position K13 points into the solvent and is close to a charged residue (red), L35 is part of a hydrophobic pocket (purple).

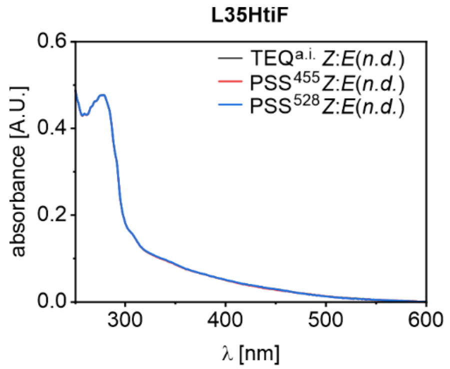

Figure S26. Absorbance spectra of 23 µM L35HtiF in 50 mM Tris/HCl pH 7.5, 100 mM NaCl. L35HtiF exhibits light scattering and a less pronounced absorbance signal, with no change upon irradiation.

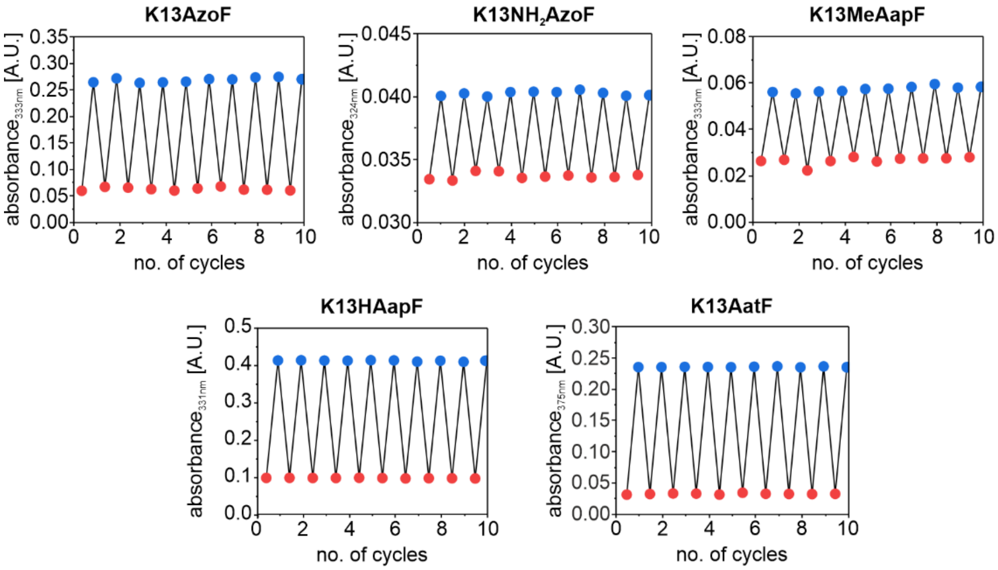

Figure S27. Cycle performance of the photoisomerization of HisF-psUAA variants in 50 mM Tris/HCl pH 7.5, 100 mM NaCl. Alternating isomerization was induced between PSS^{365} and PSS^{420} for K13AzoF, PSS^{385} and TEQ^{post} for NH_2AzoF, PSS^{365} and PSS^{420} for K13MeAapF, PSS^{365} and PSS^{505} for K13HAapF, and PSS^{400} and PSS^{528} for K13AatF. The absorbance at the characteristic maxima was plotted against the number of cycles (switching to *E* and subsequently to *Z* represents one cycle). All HisF-psUAA variants tolerated repeated photoswitching for ten cycles without photobleaching effects (decrease in absorbance) or light scattering effects (increase in absorbance). For K13MeAapF and K13HAapF, PSS^{420} and PSS^{505} instead of PSS^{528} were used for the sake of time, because considerably less time was required for switching over ten cycles, while simultaneously yielding comparable photoconversion yields (cf. **Figure S23**). For **HtiF**, minor fluctuations in overall absorbance combined with insufficient absorption changes prevented the acquisition of reliable data.

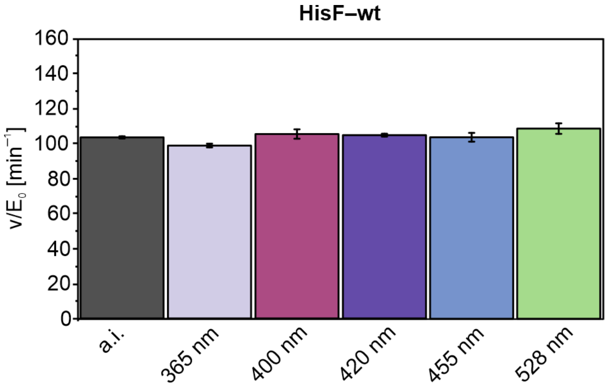

Figure S28. HisF-wt activity before and after irradiation with the most effective wavelengths for psUAA isomerization in the presence of 7 µM ProFAR (close to K_m of HisF-wt). To this end, HisF-wt was subjected to the same irradiation conditions verifying that the enzyme system is not impaired by light exposure. Reaction conditions included 50 mM Tris acetate pH 8.5, 100 mM ammonium acetate, 1 µM HisA (to convert ProFAR to PrFAR) and 7 µM ProFAR at 25 °C. Measurements were performed in technical triplicates, which were fitted simultaneously to obtain a mean fit value (column) ± S.E. (error bars). The LRF values comparing $TEQ^{a.i.}$ with either PSS^{λ} were obtained by selecting the respective data sets of triplicates and fitting them with a global fit analysis using a linear regression interaction model. Obtained v/E_0 ± S.E. and LRF ± S.E. values are listed in **Table S12**.

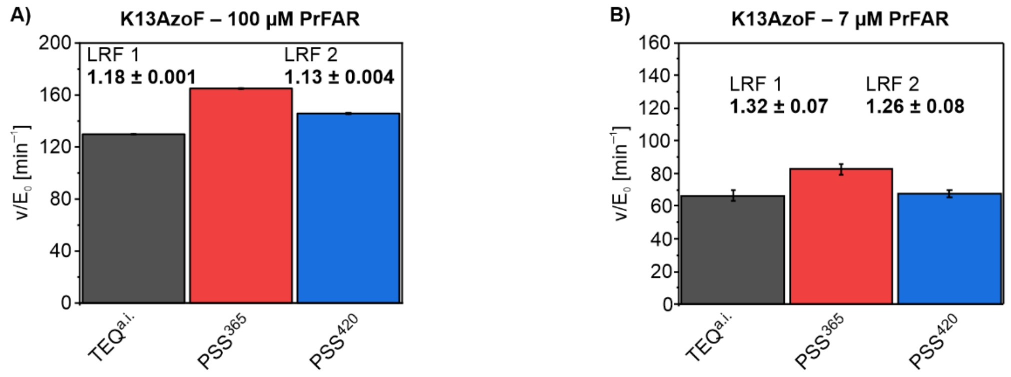

Figure S29. Photocontrol of K13AzoF activity in the presence of 100 µM PrFAR (saturation; A) or 7 µM PrFAR (close to K_m of HisF-wt, B). Reaction conditions included 50 mM Tris acetate pH 8.5, 100 mM ammonium acetate, 1 µM HisA (to convert ProFAR to PrFAR) and 7/100 µM ProFAR at 25 °C. The reaction was started with 33 nM K13AzoF either in its $TEQ^{a.i.}$, PSS^{365} or PSS^{420}. Measurements were performed in technical triplicates, which were fitted simultaneously to obtain a mean fit value (column) ± S.E. (error bars). The LRF values comparing either $TEQ^{a.i.}$ with PSS^{365} or PSS^{365} with PSS^{420} were obtained by selecting the respective data sets of triplicates and fitting them with a global fit analysis using a linear regression interaction model.

Table S12. Photocontrol of HisF-wt and HisF-psUAA activity and deduced LRF values.

Protein		v/E_0 [min^{-1}]	LRF[c]	p-value of LRF[d]
HisF-wt[a]	TEQ$^{a.i.}$	102.8 ± 0.5		
	PSS^{365}	98.2 ± 1.1	↓ 1.05 ± 0.01[e]	2.20E-294
	PSS^{400}	104.7 ± 2.7	↑ 1.02 ± 0.03[e]	5.93E-147
	PSS^{420}	104.1 ± 0.8	↑ 1.01 ± 0.01[e]	0
	PSS^{455}	103.1 ± 2.5	↑ 1.00 ± 0.02[e]	6.41E-155
	PSS^{528}	108.0 ± 3.0	↑ 1.05 ± 0.03[e]	4.13E-135
K13AzoF (100 µM PrFAR) [b]	TEQ$^{a.i.}$	129.9 ± 0.06		
	PSS^{365}	165.0 ± 0.31	↑ 1.18 ± 0.001[f]	0
	PSS^{420}	145.8 ± 0.44	↓ 1.13 ± 0.004[f]	0
K13AzoF[a]	TEQ$^{a.i.}$	65.0 ± 2.7		
	PSS^{365}	85.5 ± 3.3	↑ 1.32 ± 0.07[f]	6.80E-60
	PSS^{420}	67.6 ± 2.4	↓ 1.26 ± 0.08[f]	1.62E-52
K13NH_2AzoF[a]	TEQ$^{a.i.}$	84.4 ± 0.5		
	PSS^{400}	100.6 ± 0.8	↑ 1.19 ± 0.01[f]	0
	TEQ^{post}	87.0 ± 0.8	↓ 1.15 ± 0.01[f]	0
K13MeAapF[a]	TEQ$^{a.i.}$	24.6 ± 0.3		
	PSS^{365}	33.5 ± 0.5	↑ 1.37 ± 0.02[f]	0
	PSS^{528}	24.4 ± 0.4	↓ 1.37 ± 0.03[f]	0
K13HAapF[a]	TEQ$^{a.i.}$	55.9 ± 0.6		
	PSS^{365}	82.5 ± 0.5	↑ 1.48 ± 0.03[f]	0
	PSS^{528}	59.9 ± 0.6	↓ 1.38 ± 0.03[f]	0
K13AatF[a]	TEQ$^{a.i.}$	56.3 ± 0.5		
	PSS^{400}	92.9 ± 0.6	↑ 1.64 ± 0.02[f]	0
	PSS^{528}	67.6 ± 0.8	↓ 1.37 ± 0.02[f]	0
K13HtiF[a]	TEQ$^{a.i.}$	23.1 ± 0.6		
	PSS^{455}	22.0 ± 0.8	↑ 1.05 ± 0.04[f]	8.75E-95
	PSS^{528}	22.4 ± 0.6	↓ 1.02 ± 0.05[f]	5.43E-74

[a] Reactions were conducted with PrFAR close to K_m as described in **Figure S29**. [b] Reactions were conducted with PrFAR in saturation. [c] generally delineates the activity ratio $\frac{v1}{v2}$ with $v_1>v_2$ with arrows indicating an activity increase (↑) or decrease (↓). [d] p-values <0.05 indicate a significantly different activity in the two compared data sets. [e] LRF values compare TEQ$^{a.i.}$ with either PSS$^{\lambda}$. [f] LRF values compare either TEQ$^{a.i.}$ with PSS^{365} / PSS^{400} / PSS^{455} or PSS^{365} / PSS^{400} / PSS^{455} with PSS^{420} / TEQ^{post} / PSS^{528}. Note: data for v/E_0 and LRF are provided as mean fit value ± S.E. of three technical replicates.

Table S13. Primers used in this work.

purpose	primers used[a]
generation of pGLNS from pEVOL (deletion of pBAD cassette)	GAGCTCCCGGTCATCAATCATCC
	CAACTTATATCGTATGGGGCTGAC
introduction of restriction sites in pGLNS for subcloning of aaRS	AAAAAA**GGTCTCA**CCATATGGGATTCCTCAAAGCG
	AAAAAA**GGTCTCT**GTAACTGCAGTTTCAAACGCTAAATTGC
introduction of restriction sites in pBAD for subcloning of sfGFP	AAAAAA**GAAGACAT**CCATAGATCTAATTCCTCCTGTTAGC
	AAAAAA**GAAGACAT**GTAATTAAACGGTCTCCAGCTTGGCTG
preparation of AzoF-RS_{copt} for pGLNS	AAAAAA**GGTCTCA**ATGGATGAGTTCGAAATGATTAAAC
	AAAAAA**GGTCTCT**TTACAGACGTTTACGAATCGGTTCCAGG
introduction of Q155V in AzoF-RS_{copt}	GATTATGGTGGTTAACGGCATTCATTATC
	GGATAAATCACTTCGGCAACTTTCG
introduction of A167V in AzoF-RS_{copt}	GTGTTGATGTTGTGGTTGGTGGTATGG
	CATGATAATGAATGCCGTTAACCTG
introduction of A167C in AzoF-RS_{copt}	GTGTTGATGTTTGCGTTGGTGGTATGG
	CATGATAATGAATGCCGTTAACCTG
introduction of V188P in AzoF-RS_{copt}	CGAAGAAACCGGTTTGTATTCATAATCC
	GCAGCAGTTCACGTGCCAGC
introduction of G32A in AzoF-RS_{copt}	CGCGATTGGTTTTGAACCGAGC
	GCTGATTTTTCATCCTTTTTCAGTACTTCG
introduction of E65L in AzoF-RS_{copt}	CGATCTGCATGCATATCTGAATCAGAAAGG
	GCCAGCAGAATGATAATATCGAAACCG
introduction of A108F in AzoF-RS_{copt}	GAATTTGAGCTGGATAAAGATTATACC
	GCTACCATAAACATATTTGGCTTTCAGGC
introduction of E109M in AzoF-RS_{copt}	GAAGCCATGCTGGATAAAGATTATACC
	GCTACCATAAACATATTTGGCTTTCAGGC
introduction of G158A in AzoF-RS_{copt}	GCGATTCATTATCATGGTGTTGATGTTGCAG
	GTTAACCTGCATAATCGGATAAATCACTTCGG
introduction of H162F in AzoF-RS_{copt}	GGCATTCATTATTTTGGTGTTGATGTTGCAG
	GTTAACCTGCATAATCGGATAAATCACTTCGG
introduction of I159Y in AzoF-RS_{copt}	GCTATCATTATCATGGTGTTGATGTTGC
	CGTTAACCTGCATAATCGGATAAATCACTTCG
introduction of I159W in AzoF-RS_{copt}	GCTGGCATTATCATGGTGTTGATGTTGC
	CGTTAACCTGCATAATCGGATAAATCACTTCG
introduction of I159F in AzoF-RS_{copt}	GCTTTCATTATCATGGTGTTGATGTTGC
	CGTTAACCTGCATAATCGGATAAATCACTTCG
introduction of I159K in AzoF-RS_{copt}	GCAAACATTATCATGGTGTTGATGTTGC
	CGTTAACCTGCATAATCGGATAAATCACTTCG
introduction of R257G in ONBY-RS_{i}	CATTAAAGGCCCAGAAAAATTTGGTGGTGATCTGAC
	GTCAGCGGGTATTCCAGAAAGTATTTGGC
generation of Azo-RS 4 from Azo-RS 1[30]	GTGTTGATGTTGCGGTTGGTGGTATGG
	CACGATAATGATAGCCGTTAACCTGC
introduction of restriction sites in pBAD for subcloning of sfGPF_Y151TAG	AAAAAA**GAAGACAT**CATCGATCTAATTCCTCCTGTTAGC
	AAAAAA**GAAGACAT**GTAATTAAACGGTCTCCAGCTTGGCTG

preparation of ONBY-RS$_i$ for subcloning in pGLNS	AAAAAA**GGTCTC**AATGGATGAGTTCGAGATGATTAAACG
	AAAAAA**GGTCTCT**TTACAGGCGTTTACGAATCGGTTCC
preparation of AzF-RS for subcloning in pGLNS	AAAAAA**GGTCTC**AATGGACGAGTTCGAAATGATTAAAC
	AAAAAA**GGTCTCT**TTACAGACGTTTGCGAATTGGTTCCAGAATC
preparation of pET24a for subcloning of pBAD_sfGFP_Y151TAG_rrnB	AAAAAA**GAAGACAT**AAGGAGGAACTATATCCGG
	AAAAAA**GAAGACAT**CGCAACGCAATTAATGTAAGTTAG
preparation of araBAD_sfGFP_Y151TAG_rrnB for subcloning in pET24a	AAAAAAA**GAAGACAT**TGCGCTTATTAATCAGATAAAATATTTC
	AAAAAAA**GAAGACAT**CCTTGCACACGGTCACACTG
introduction of TAG151Y in sfGFP_Y151TAG	GTCTATATCACCGCAGATAAACAGAAAAACG
	GTTGTGGCTGTTGAAATTATATTCCAGTTTGTGG

[a] recognition sites for BsaI or BbsI are shown in bold and the resulting four base pair overhangs are highlighted in red. Point mutations are underlined.

2. Experimental section

2.1 Synthesis of photoswitchable UAAs

AzoF was synthesized according to a previously published protocol.[31] Detailed synthesis schemes of psUAAs and corresponding procedures are given below. Starting materials and reagents were purchased from commercial suppliers (Sigma Aldrich, Alfa Aesar, Acros, Fluka, TCI or VWR) and used without further purification. Solvents were used as p.a. grade or distilled. Dry solvents were dried over molecular sieves. For automated flash column chromatography industrial grade of solvents was used. All NMR spectra were measured at room temperature using a Bruker Avance 300 (300 MHz for ^{1}H, 75 MHz for ^{13}C, 282 MHz for ^{19}F) or a Bruker Avance 400 (400 MHz for ^{1}H, 101 MHz for ^{13}C, 376 MHz for ^{19}F) NMR spectrometer. All chemical shifts are reported in *δ*-scale as parts per million [ppm] (multiplicity, coupling constant J, number of protons) relative to the solvent residual peaks as the internal standard. Coupling constants J are given in Hertz [Hz]. Abbreviations used for signal multiplicity: ^{1}H NMR: b = broad, s = singlet, d = doublet, t = triplet, q = quartet, hept = heptet dd = doublet of doublets, dt = doublet of triplets, dq = doublet of quartets, and m = multiplet; ^{13}C NMR: (+) = primary/tertiary, (-) = secondary, (Cq) = quaternary carbon. HRMS (high resolution mass spectra) and LRMS (low resolution mass spectra) were measured at the Central Analytical Laboratory of the University of Regensburg. These mass spectra were recorded on a Finnigan MAT 95, ThermoQuest Finnigan TSQ 7000, Finnigan MAT SSQ 710 A or an Agilent Q-TOF 6540 UHD instrument. Analytical TLC was performed on silica gel coated alumina plates (MN TLC sheets ALUGRAM® Xtra SIL G/UV254). Visualization was done by UV light (254 or 366 nm). If necessary, ninhydrin, vanillin or permanganate were used for chemical staining. Purification by column chromatography was performed with silica gel 60 M (40-63 µm, 230-440 mesh, Merck) or with a pre-packed Biotage® Sfär C18 D Duo 100 Å 30 µm column on a Biotage® IsoleraTM Spektra One device.

2.1.1 Schemes

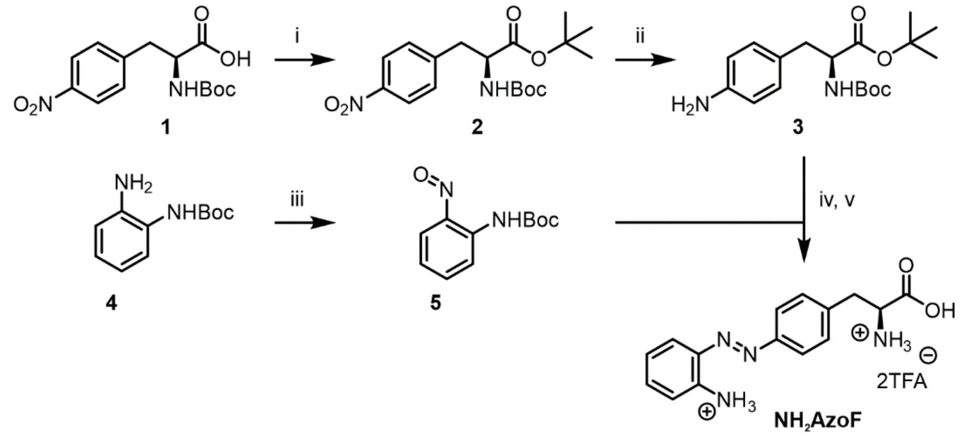

Scheme S1. Synthesis of **NH_2AzoF**. Reaction conditions: (i) Boc_2O, DMAP, tBuOH; (ii) H_2, Pd/C; (iii) oxone; iv) AcOH; v) TFA. Overall yield 10%.

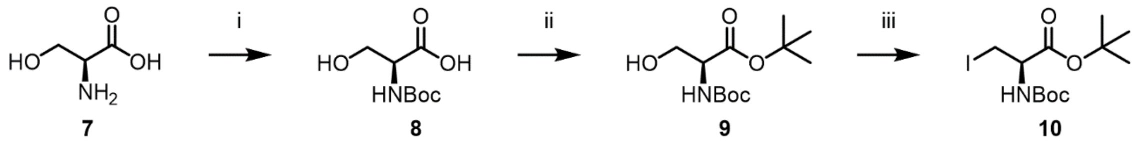

Scheme S2. Synthesis of *tert*-butyl (*R*)-2-((*tert*-butoxycarbonyl)amino)-3-iodopropanoate as precursor for the syntheses of **MeAapF**, **HtiF**, and **SpiroY**. Reaction condtions: (i) Boc_2O, NaOH; (ii) tert-butyl N,N'-diisopropylcarbamimidate; (iii) I_2, PPh_3, imidazole.

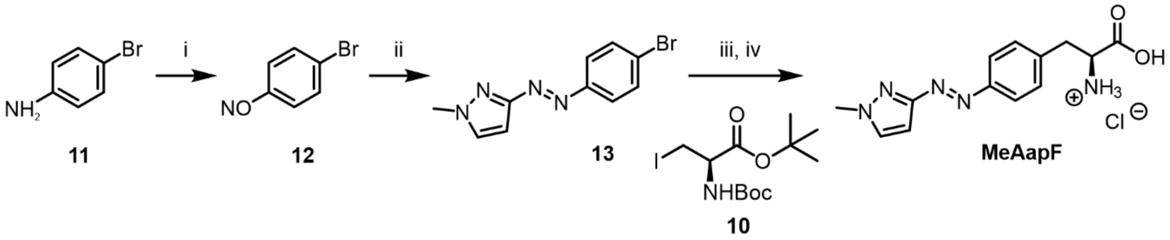

Scheme S3. Synthesis of **MeAapF**. Reaction conditions: (i) Oxone; (ii) 1-methyl-3-aminopyrazole, AcOH; (iii) Zn, 1,2.dibromoethane, TMSCl, $Pd_2(dba)_3$, SPhos; (iv) TFA. Synthesis of the precursor **10** is shown in Scheme 2. Overall yield 16%.

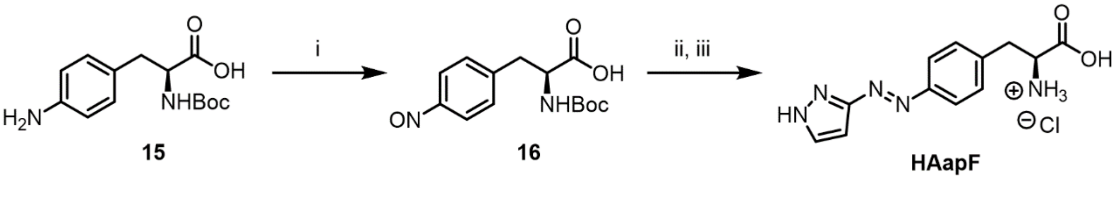

Scheme S4. Synthesis of **HAapF**. Reaction conditions: (i) Oxone; (ii) 3-aminopyrazole, AcOH; (iii) HCl. Overall yield 3%.

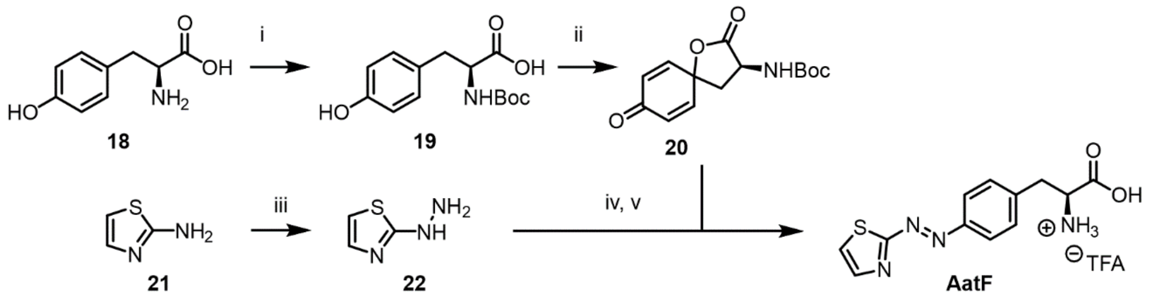

Scheme S5. Synthesis of **AatF**. Reaction conditions: (i) Boc_2O, Et_3N; (ii) $PhI(OAc)_2$; (iii) HCl, $NaNO_2$, $SnCl_2$; (iv) $Ce(NO_3)_6(NH_4)_2$; (v) TFA. Overall yield 6%.

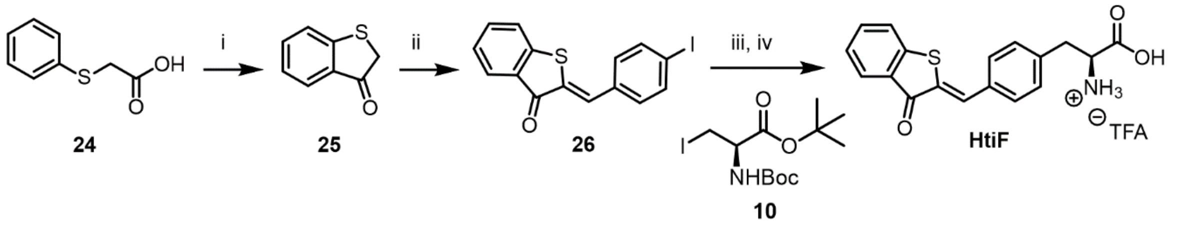

Scheme S6. Synthesis of **HtiF**. Reaction conditions: (i)TfOH; (ii) *p*-iodobenzaldehyde, piperidine; (iii) Zn, 1,2.dibromoethane, TMSCl, $Pd_2(dba)_3$, SPhos; (iv) TFA. Synthesis of the precursor **10** is shown in Scheme 2. Overall yield 4%.

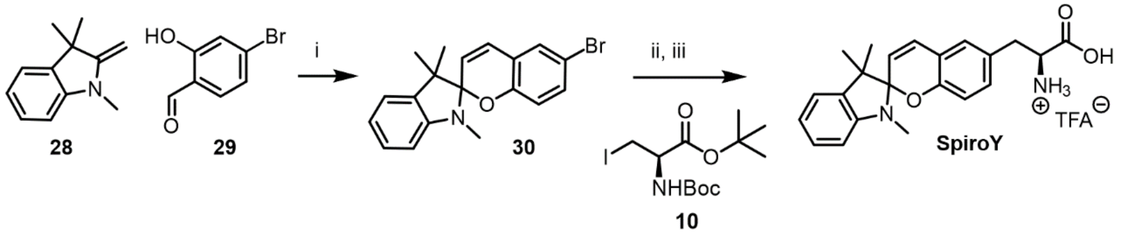

Scheme S7. Synthesis of **SpiroY**. Reaction conditions: (i) piperidine; (ii) Zn, 1,2.dibromoethane, TMSCl, $Pd_2(dba)_3$, SPhos; (iii) TFA. Synthesis of the precursor **10** is shown in Scheme 2. Overall yield 71%.

2.1.2 Procedures

^1H NMR spectra of all synthesized compounds and ^{13}C spectra of compounds that have not yet been reported are given in this document. Corresponding raw data as well as reports on MS spectra can be found in: DOI: 10.22000/41f30q9xz9mmznqp

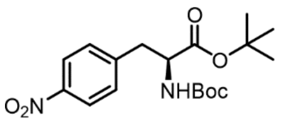

***tert*-butyl (*S*)-2-((*tert*-butoxycarbonyl)amino)-3-(4-nitrophenyl)propanoate 2:** Synthesized according to literature reports.[32] To Boc-4-nitrophenylalanine **1** (6.45 mmol, 1 eq) in 10 mL *t*-BuOH:DCM 1:2, DMAP (0.3 eq) Boc anhydride (1.1 eq) was added. Reaction mixture was stirred at room temperature (rt) overnight. All solvents were evaporated *in vacuo* and the product was purified by silica gel flash column chromatography (PE:EtOAc 9:1). Colorless oil turned to sticky white solid overnight, 87% yield. ^1H-NMR (300 MHz, Chloroform-*d*): *δ* 8.16 (d, J = 8.7 Hz, 2H), 7.35 (d, J = 8.7 Hz, 2H), 5.07 (d, J = 7.7 Hz, 1H), 4.49 (q, J = 6.7 Hz, 1H), 3.36 - 2.93 (m, 2H), 1.41 (d, J = 1.2 Hz, 18H). Data matches with existing literature.

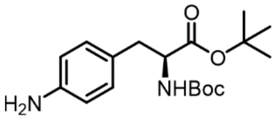

***tert*-butyl (*S*)-3-(4-aminophenyl)-2-((*tert*-butoxycarbonyl)amino)propanoate 3:** Synthesized according to literature reports.[32] Compound **2** (1.4 mmol, 1 eq) was dissolved in 10 mL EtOH and Pd/C (0.05 eq) was added. After 3 vacuum/H_2 cycles, the reaction was left at room temperature overnight with a H_2 balloon. Filtered through Celite® and dried. Pinkish solid, 100% yield. ^1H-NMR (300 MHz, Chloroform-*d*): *δ* 6.94 (d, J = 8.4 Hz, 2H), 6.61 (d, J = 8.4 Hz, 2H), 4.94 (d, J = 8.4 Hz, 1H), 4.48 - 4.29 (m, 1H), 3.61 (s, 2H), 2.94 (d, J = 5.9 Hz, 2H), 1.41 (d, J = 1.9 Hz, 18H). Data matches with existing literature.

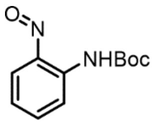

***tert*-butyl (2-nitrosophenyl)carbamate 5**: Synthesized according to literature reports.[33] To a solution of *tert*-butyl-N-(2-aminophenyl)carbamate **4** (2.4 mmol, 1 eq) in DCM (5 mL) a solution of Oxone® (2 eq) in water (20 mL) was added and stirred vigorously at ambient temperature for 1 h. The organic phase was extracted with DCM and the organic layers were combined, washed with 1 M HCl and saturated $NaHCO_3$, dried over $MgSO_4$, filtered and concentrated under reduced pressure. The resulting brown solid was obtained in 43% yield. ^1H-NMR (300 MHz, Chloroform-*d*): *δ* 10.20 (s, 1H), 8.58 (dd, J = 8.6, 1.2 Hz, 1H), 7.65 (dddd, J = 8.8, 7.1, 1.7, 0.6 Hz, 1H), 7.49 - 7.30 (m, 1H), 7.06 (ddd, J = 8.2, 7.1, 1.2 Hz, 1H), 1.56 (s, 9H). Data matches with existing literature.

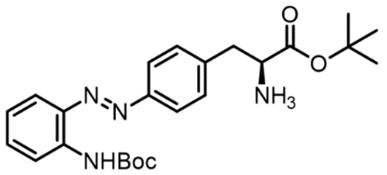

***tert*-butyl (*S*,*E*)-2-((*tert*-butoxycarbonyl)amino)-3-(4-((2-((*tert*-butoxy-carbonyl)amino)phenyl)diazenyl)phenyl)propanoate 6:** Compounds **3** (1.5 mmol, 1 eq) and **5** (1 eq) were mixed in 15 mL AcOH and stirred at rt overnight. The reaction was quenched with saturated $NaHCO_3$ and the

organic phase was extracted with EtOAc, washed with brine and dried over $MgSO_4$. Separation by column chromatography (PE:EtOAc 95:5) obtained an orange oil in 25% yield. ^{1}H-NMR (300 MHz, Chloroform-*d*): *δ* 9.21 (s, 1H), 8.38 (dd, J = 8.5, 1.4 Hz, 1H), 7.88 - 7.71 (m, 3H), 7.43 (ddd, J = 8.6, 7.3, 1.6 Hz, 1H), 7.39 - 7.31 (m, 2H), 7.08 (ddd, J = 8.2, 7.1, 1.2 Hz, 1H), 5.06 (d, J = 7.8 Hz, 1H), 4.51 (q, J = 6.2 Hz, 1H), 3.29 - 3.00 (m, 2H), 1.57 (s, 9H), 1.43 (d, J = 1.4 Hz, 18H). ^{13}C NMR (101 MHz, CDCl3) δ 170.9, 155.2, 152.7, 151.6, 140.3, 138.9, 137.0, 132.8, 130.6, 122.8, 122.2, 120.3, 118.8, 82.5, 80.9, 80.0, 54.9, 38.7, 28.5, 28.1. HRMS (ESI-TOF) calculated for $[C_{29}H_{41}N_4O_6]^+$ $[MH]^+$ 541.3021, found 541.2930.

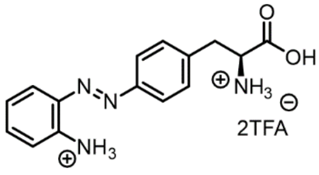

(S,E)-2-amino-3-(4-((2-aminophenyl)diazenyl)phenyl)propanoic acid NH_2AzoF: 0.37 mmol (1 eq) of **6** was dissolved in 5 mL DCM and excess TFA was added before the reaction was allowed to stir at rt for 2 days. The solvent was removed by rotatory evaporation and the residue was dissolved in a minimum amount of water and lyophilized. The compound was then triturated with Et_2O and dried in vacuum to obtain the final product as a double TFA salt. Red solid in 97% yield. ^{1}H-NMR (400 MHz, DMSO-*d$_6$*): *δ* 10.01 (s, 3H), 8.38 (s, 3H), 7.87 (d, J = 8.1 Hz, 2H), 7.64 (dd, J = 8.1, 1.6 Hz, 1H), 7.43 (d, J = 8.1 Hz, 2H), 7.28 - 7.09 (m, 1H), 6.88 (d, J = 8.3 Hz, 1H), 6.65 (t, J = 7.5 Hz, 1H), 4.26 (s, 1H), 3.19 (dd, J = 6.4, 2.7 Hz, 2H). ^{13}C-NMR (101 MHz, DMSO-*d$_6$*): *δ* 170.4, 151.8, 145.1, 137.0, 135.9, 132.7, 130.5, 124.6, 122.3, 117.3, 115.8, 53.1, 35.7. HRMS (ESI-TOF) m/z calculated for $[C_{15}H_{17}N_4O_2]^+$ $[MH]^+$ 285.1347, found 285.1363.

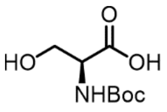

(*tert*-butoxycarbonyl)-*L*-serine 8: Synthesized according to a reported procedure.[34] A solution of *L*-serine **7** (47.5 mmol, 1 eq) in 1 M NaOH (50 mL) and dioxane (25 mL) at 0 °C was treated with Boc_2O (1.2 eq) and the mixture was allowed to warm to rt and stirred for 24 h. The dioxane was then evaporated and the aqueous layer was washed with Et_2O. EtOAc was then added to the aqueous layer and the pH was adjusted with 1 M HCl to pH 2-3. The organic layer was extracted, and the aqueous layer was saturated with NaCl and extracted with EtOAc. The combined organic layers were dried and filtered, and the solvent was removed in vacuum to obtain a thick colorless oil in 100% yield. ^{1}H-NMR (400 MHz, Methanol-*d$_4$*): *δ* 4.19 (t, J = 4.3 Hz, 1H), 3.83 (qd, J = 11.3, 4.3 Hz, 2H), 1.45 (s, 9H). Data matches with existing literature.

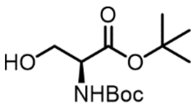

***tert*-butyl (*tert*-butoxycarbonyl)-*L*-serinate 9**: Synthesized according to a reported procedure.[34] **8** (4.9 mmol, 1 eq) was dissolved in DCM and cooled to 0 °C under N_2. *tert*-butyl N,N'-diisopropylcarbamimidate (3 eq) was added in three portions over 5 min. The reaction was stirred overnight at rt before it was stirred with hexane for 30 min, then filtered and concentrated under reduced pressure. The crude was purified by column chromatography (PE:EtOAc 3:1 to 1:1) to yield a colorless oil/sticky solid, with 57% yield. ^{1}H-NMR (300 MHz, Chloroform-*d*): *δ* 5.42 (s, 1H), 4.25 (s, 1H), 3.89 (d, J = 3.8 Hz, 2H), 1.48 (s, 9H), 1.45 (s, 9H). Data matches with existing literature.

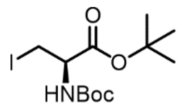

***tert*-butyl (*R*)-2-((*tert*-butoxycarbonyl)amino)-3-iodopropanoate 10**: Synthesized according to a reported procedure.[35] To a solution of triphenylphosphine (1.3 eq) and imidazole (1.3 eq) in DCM (20 mL) at 0°C was added iodine (1.3 eq) in 3 portions over 30 min. A solution of **9** (2.8 mmol, 1 eq) in DCM (20 mL) was added dropwise. The resultant solution was stirred at 0°C for 1 h before being warmed to room temperature for 2 h. Methanol (10 mL) was added, and the solvent was removed *in vacuo*. Purification by flash column chromatography (PE:EtOAc, 9:1). Colorless oil, 49% yield. ^{1}H-NMR (300 MHz, Chloroform-*d*): δ 5.34 (d, J = 6.6 Hz, 1H), 4.34 (dt, J = 7.4, 3.5 Hz, 1H), 3.56 (d, J = 3.8 Hz, 2H), 1.50 (s, 9H), 1.45 (s, 9H). Data matches with existing literature.

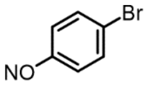

1-bromo-4-nitrosobenzene 12: Synthesized according to literature report.[36] A solution of Oxone® (2 eq) in water was added to a solution of 4-bromoaniline **11** (4.6 mmol, 1 eq) in DCM with vigorous stirring. The reaction mixture was stirred at room temperature for 3h. After disappearance of starting materials (indicated by TLC), the reaction was extracted with DCM. The combined organic layers were dried over $MgSO_4$ and concentrated under reduced pressure to give a light brown solid in quantitative yield. ^{1}H-NMR (400 MHz, Chloroform-*d*): δ 7.78 (s, 4H) ppm. Data matches with existing literature.

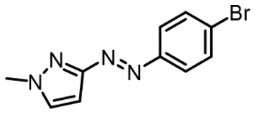

(*E*)-3-((4-bromophenyl)diazenyl)-1-methyl-1*H*-pyrazole 13: A solution of **12** and N-methyl-3-aminopyrazole in a mixture of DCM:AcOH 1:1 was stirred at rt overnight. The reaction was quenched with $NaHCO_3$, diluted with water and extracted with EtOAc, separated by column chromatography and dried in high vacuum. Yellow solid, 28% yield. ^{1}H-NMR (400 MHz, Chloroform-*d*): δ 7.91 - 7.73 (m, 2H), 7.70 - 7.54 (m, 2H), 7.40 (d, J = 2.4 Hz, 1H), 6.64 (d, J = 2.5 Hz, 1H), 4.02 (s, 3H). ^{13}C NMR (101 MHz, Chloroform-*d*) δ 163.7, 151.6, 132.4, 132.1, 125.6, 124.5, 96.5, 39.9. HRMS (ESI-TOF) calculated for $[C_{10}H_{10}BrN_4]^+$ $[MH]^+$ 265.0084, found 265.0084.

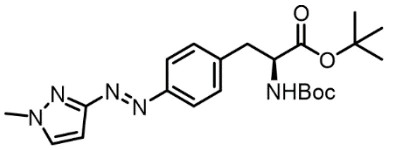

***tert*-butyl (*S*,*E*)-2-((*tert*-butoxycarbonyl)amino)-3-(4-((1-methyl-1*H*-pyrazol-3-yl)diazenyl)phenyl)propanoate 14**: Procedure adapted from literature.[37] Zinc (3 eq) was added to a dry crimp capped vial and purged with three cycles of vacuum/N_2. Dry DMF (1 mL), 1,2-dibromoethane (0.08 eq), and TMSCl (0.04 eq) were added to the flask. Stirred reaction was heated to 60°C under N_2 for 5 min, and then it was allowed to cool to rt before the addition of **10** (1.2 mmol, 1.5 eq). The sealed reaction was again purged with three cycles of vacuum/N_2 and sonicated without stirring for 30 min at 35°C. **13** (0.80 mmol, 1 eq), Pd_2dba_3 (0.025 eq.) and SPhos (0.05 eq.) were added in another crimp capped vial and purged with three cycles of vacuum/N_2 before 1 mL DMF was added. The organozinc mixture was carefully syringed, with minimal transfer of unreacted zinc, in the second vial and stirred overnight at 70°C under N_2. Reaction mixture was quenched with saturated NH_4Cl and extracted with EtOAc. Organic phase was washed with brine, dried over $MgSO_4$, filtered, concentrated and dried under high vacuum. Crude was purified by flash chromatography (PE:EtOAc 95:5 to 90:10) to obtain a yellow oil in 63% yield. ^{1}H-NMR (400

MHz, Chloroform-*d*): *δ* 7.88 (d, J = 8.5 Hz, 2H), 7.39 (d, J = 2.5 Hz, 1H), 7.31 (d, J = 8.5 Hz, 2H), 6.64 (d, J = 2.5 Hz, 1H), 5.05 (d, J = 7.5 Hz, 1H), 4.49 (d, J = 6.8 Hz, 1H), 4.03 (s, 3H), 3.13 (d, J = 5.7 Hz, 2H), 1.43 (s, 9H), 1.39 (s, 9H). ^{13}C NMR (101 MHz, CDCl3) δ 215.1, 179.4, 163.5, 151.5, 139.8, 131.6, 130.1, 122.8, 96.0, 82.2, 77.0, 60.2, 39.5, 28.2, 27.8, 20.9, 14.0. HRMS (ESI-TOF) m/z calculated for $[C_{22}H_{32}N_5O_4]^+$ [MH]$^+$ 430.2449, found 430.2455.

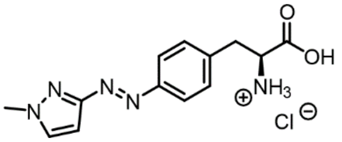

(*S*,*E*)-2-amino-3-(4-((1-methyl-1*H*-pyrazol-3-yl)diazenyl)phenyl) propanoic acid MeAapF: Compound **14** (0.57 mmol, 1 eq) was dissolved in dioxane:HCl 4 M 1:1 and stirred for 2 days at rt. The solvent was evaporated, and the residue was lyophilized, then triturated with Et_2O and dried in *vacuo* to obtain the final product as a HCl salt. Yellow solid, 91% yield. ^{1}H-NMR (400 MHz, DMSO-*d*$_6$): *δ* 8.53 (dd, J = 4.8, 1.6 Hz, 3H), 7.84 (d, J = 2.4 Hz, 1H), 7.82 - 7.75 (m, 2H), 7.56 - 7.44 (m, 2H), 6.53 (d, J = 2.5 Hz, 1H), 4.23 (d, J = 5.5 Hz, 1H), 3.97 (s, 3H), 3.24 (d, J = 6.4 Hz, 2H). ^{13}C-NMR (101 MHz, DMSO-*d*$_6$): *δ* 170.3, 163.0, 151.4, 138.5, 133.2, 130.7, 122.4, 94.5, 53.0, 35.5. HRMS (ESI-TOF) m/z calculated for $[C_{13}H_{16}N_5O_2]^+$ [MH]$^+$ 274.1299, found 274.1302.

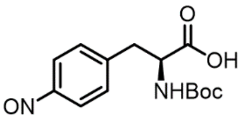

(*S*)-2-((tert-butoxycarbonyl)amino)-3-(4-nitrosophenyl)propanoic acid 16: To a solution of amino acid (7.13 mmol, 1 eq.) in DCM (15.0 mL/mmol) at 0 °C, Oxone® (7.13 mmol, 1.0 eq) in water (15.0 mL/mmol) was added. The orange, biphasic was allowed to stir for 1 h at 0°C, before the ice bath was removed and the mixture was stirred for another 1 h. Afterwards, the aqueous phase was decanted. To the remaining organic layer in the round bottom flask, water (2x 20 mL) was added and once again decanted off. The resulting organic DCM mixture in the round bottom flask was used directly in the next step. The water layer was further extracted with 1% AcOH (10 ml) and DCM (2x 10 ml). Without further drying, the organic phase was used directly in the next step because of instability of the reaction product.

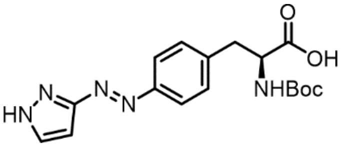

(*S*,*E*)-3-(4-((1*H*-pyrazol-3-yl)diazenyl)phenyl)-2-((*tert*-butoxycarbonyl) amino)propanoic acid 17: Following nitroso formation, 3-aminopyrazole (7.48 mmol, 1.10 eq.) and DCM/ glacial AcOH (15 mL/mmol) were added to the nitroso intermediate **16**. The orange solution was allowed to stir for 15 hours under N_2 atmosphere in the dark. The solvent was removed *in vacuo* and the crude product was subjected to reverse phase chromatography (solvent A: H_2O + 0.05% TFA, solvent B: MeCN, gradient: 0–100% B) The product was obtained as a yellow solid (4%). ^{1}H NMR (400 MHz, MeOD) δ 7.72 (d, J = 8.2 Hz, 2H), 7.59 (t, J = 4.9 Hz, 1H), 7.31 (d, J = 8.2 Hz, 2H), 6.54 (d, J = 2.5 Hz, 1H), 4.31 (dd, J = 9.1, 5.0 Hz, 1H), 3.23-3.13 (m, 3.2 Hz, 2H), 2.92-2.86 (m, 1H), 1.25 (s, 9H). ^{13}C-NMR (151 MHz, MeOD) δ 175.1, 152.9,

142.8, 131.3, 123.7, 80.6, 56.1, 38.6, 28.7. HRMS (ESI-TOF) m/z calculated for $[C_{17}H_{21}N_5O_4]^+$ $[MH]^+$ 360.1667, found 360.1672.

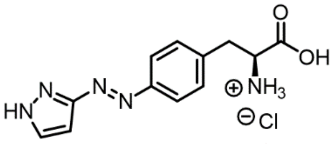

(*S*,*E*)-3-(4-((1*H*-pyrazol-3-yl)diazenyl)phenyl)-2-aminopropanoic acid HAapF: The Boc protected amine **17** (0.139 mmol, 1.0 eq.) was dissolved in dioxane (4.0 mL/mmol) at 0 °C and 4 N HCl (1.33 mL/mmol) was added dropwise. The reaction mixture was allowed to stir at room temperature for 5 h. Toluene (2.00 mL/mmol) was added, and the solvents were removed in *vacuo*. Subsequently, the product was triturated with diethyl ether (3x 1.0 mL/mmol) to obtain the target compound as a yellow solid (83%). ^{1}H-NMR (400 MHz, MeOD): δ (ppm) = 7.91 (d, J = 8.40 Hz, 2H), 7.73 (d, J = 2.50 Hz, 1H), 7.49 (d, J = 8.40 Hz, 2H), 6.67 (d, J = 2.52 Hz, 1H), 4.30 (dd, J = 7.7, 5.6 Hz, 1H), 3.40 (dd, J = 14.5, 5.6 Hz, 1H), 3.25 (dd, J = 14.5, 7.7 Hz, 1H). ^{13}C-NMR (101 MHz, MeOD): δ = 171.27, 164.71, 153.57, 139.38, 132.47, 131.48, 124.31, 95.41, 55.06, 37.20. ESI-MS (m/z): $[M^+H]^+$ calculated: 260.1142; found: 260.1143.

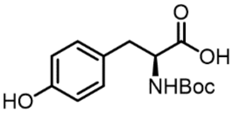

(*tert*-butoxycarbonyl)-*L*-tyrosine 19: Synthesized according to literature report.[38] To a solution of *L*-tyrosine (11 mmol, 1 eq) in 12 mL 1:1 dioxane/water was added Et_3N (1.5 eq). The reaction flask was cooled to 0°C with an ice bath and Boc anhydride (1.1 eq) was added. After 1 h, the cold bath was removed, and the reaction mixture was stirred at ambient temperature for 20 h. The reaction mixture was then concentrated on a rotary evaporator and the residue diluted with water and ethyl acetate. The aqueous layer was acidified to pH 1 with 1 M HCl and extracted with ethyl acetate. The organic layer was washed with brine, dried over $MgSO_4$ and evaporated and a white solid was obtained with 74% yield. ^{1}H-NMR (300 MHz, Chloroform-*d*): *δ* 7.01 (d, J = 8.0 Hz, 2H), 6.74 (d, J = 8.1 Hz, 2H), 4.99 (d, J = 7.8 Hz, 1H), 4.55 (d, J = 7.4 Hz, 1H), 3.05 (s, 2H), 1.43 (s, 9H). Data matches with existing literature.

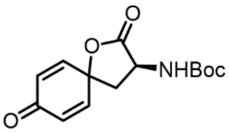

***tert*-butyl (*S*)-(2,8-dioxo-1-oxaspiro[4.5]deca-6,9-dien-3-yl)carbamate 20**: Synthesized according to literature report.[39] Phenyliodonium diacetate (1.1 eq) was added to a round-bottom flask containing 10 mL acetonitrile, and the mixture was stirred at room temperature. A solution of **19** (5 mmol, 1 eq) in 10 mL acetonitrile was added to the above round-bottom flask dropwise over 20 min. The mixture was allowed to stir for overnight before diluting with 20 mL ethyl acetate and 20 mL brine. The reaction mixture was extracted with ethyl acetate, and the organic layers were combined, dried over $MgSO_4$ and concentrated under reduced pressure. The crude mixture was purified by column PE:EtOAc 3:1 to 1:1 and a yellowish solid was obtained with 20% yield. ^{1}H-NMR (300 MHz, Chloroform-*d*): *δ* 6.96 - 6.75 (m, 2H), 6.30 (td, J = 10.4, 2.1 Hz, 2H), 5.15 (s, 1H), 4.53 (s, 1H), 2.76 (dd, J = 12.9, 9.1 Hz, 1H), 2.53 - 2.34 (m, 1H), 1.46 (s, 9H). Data matches with existing literature.

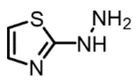

2-hydrazineylthiazole 22: A solution of sodium nitrite (1 eq) in water was added dropwise to a suspension of 2-aminothiazole (20 mmol 1 eq) in HCl (16mL) at –10°C. The cold diazonium salt solution was added dropwise to a solution of stannous chloride (2 eq) in HCl (20mL) at –10°C. The reaction mixture was stirred for 1 h, neutralized with NaOH 4 M, and filtered. The filtrate was brought to pH>9 extracted with EtOAc, dried over $MgSO_4$ and dried in vacuum. Grey solid was obtained with 11% yield. ^{1}H-NMR (400 MHz, DMSO-*d$_6$*): *δ* 8.34 (s, 1H), 7.02 (d, J = 3.6 Hz, 1H), 6.63 (d, J = 3.6 Hz, 1H), 4.76 (s, 2H). ^{13}C NMR (101 MHz, DMSO) δ 176.6, 139.4, 106.9. HRMS (ESI-TOF) m/z calculated for $[C_3H_6N_3S]^+$ $[MH]^+$ 116.0277, found 116.0280.

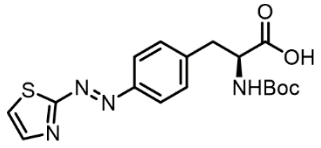

(*S*,*E*)-2-((*tert*-butoxycarbonyl)amino)-3-(4-(thiazol-2-yldiazenyl)phenyl) propanoic acid 23: Adapted from reported procedure.[3] **20** (1.1 mmol, 1 eq) and **22** (0.9 eq) were mixed in 10 mL acetonitrile in the presence of 3 mol% cerium(IV) ammonium nitrate at room temperature overnight. The solution was concentrated under reduced pressure. The crude product was purified by flash chromatography DCM:MeOH 100:0 to 90:10. Part of nonreacted spirolactone was also recovered. Orange solid was obtained with 55% yield. ^{1}H-NMR (400 MHz, Chloroform-*d*): *δ* 8.02 (d, J = 3.3 Hz, 1H), 7.92 (d, J = 8.3 Hz, 2H), 7.41 (d, J = 3.4 Hz, 1H), 7.38 (d, J = 8.4 Hz, 2H), 5.21 (d, J = 8.1 Hz, 1H), 4.76 - 4.53 (m, 1H), 3.38 - 3.09 (m, 2H), 1.42 (s, 9H). ^{13}C-NMR (101 MHz, Chloroform-*d*): *δ* 177.4, 174.6, 155.4, 150.7, 143.6, 142.4, 130.7, 124.2, 121.6, 38.2, 31.1, 28.4. HRMS (ESI-TOF) m/z calculated for $[C_{17}H_{21}N_4O_4S]^+$ $[MH]^+$ 377.1279, found 377.1286.

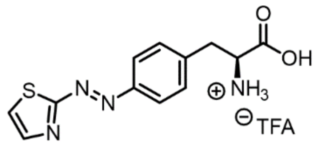

(*S*,*E*)-2-amino-3-(4-(thiazol-2-yldiazenyl)phenyl)propanoic acid AatF: Compound **23** (0.23 mmol, 1 eq) was dissolved in 5 mL DCM and excess TFA is added before the solution was stirred at rt for 2 days. The solvent was removed by rotatory evaporation and the residue was dissolved in a minimum amount of water and lyophilized. The compound was then triturated with Et_2O and dried in vacuum to obtain the final product as a TFA salt. A red solid was obtained with 88% yield. ^{1}H-NMR (400 MHz, DMSO-*d$_6$*): *δ* 8.15 (d, J = 3.3 Hz, 1H), 7.96 (d, J = 3.3 Hz, 1H), 7.93 (d, J = 8.4 Hz, 2H), 7.54 (d, J = 8.5 Hz, 2H), 4.16 (t, J = 6.6 Hz, 1H), 3.21 (qd, J = 14.4, 6.7 Hz, 2H). ^{13}C-NMR (101 MHz, DMSO-*d$_6$*): *δ* 176.1, 170.2, 150.2, 144.4, 141.5, 131.0, 123.5, 123.4, 53.3, 36.0. HRMS (ESI-TOF) m/z calculated for $[C_{12}H_{13}N_4O_2S]^+$ $[MH]^+$ 277.0754, found 277.0760.

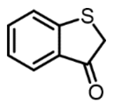

benzo[*b*]thiophen-3(2*H*)-one 25: Synthesized according to reported procedure.[40] To a flame-dried Schlenk flask equipped with a magnetic stir bar thiophenoxy acetic acid (3 mmol, 1 eq) and 10 mL DCM were added. To this solution, TfOH (5eq) was carefully added. Subsequently, the

flask was sealed, placed into a prewarmed oil bath set to 40°C and stirred for 7 hours. After 7 hours, the reaction mixture was cooled to room temperature and carefully poured into ice/water. The organic layer was separated, and the aqueous layer was further extracted with DCM. The combined organic layers were washed with saturated $NaHCO_3$ and brine, before they were dried over anhydrous $MgSO_4$, filtered and concentrated under reduced pressure by rotary evaporation. The the crude product was obtained as a yellow solid in 52% yield and was immediately used without further purification. ^{1}H-NMR (300 MHz, Chloroform-*d*): *δ* 7.78 (dd, J = 7.8, 1.2 Hz, 1H), 7.55 (ddd, J = 8.2, 7.2, 1.3 Hz, 1H), 7.43 (d, J = 8.0 Hz, 1H), 7.25 - 7.17 (m, 1H), 3.79 (s, 2H). Data matches with existing literature.

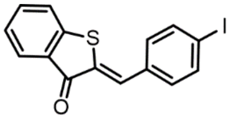

(*Z*)-2-(4-iodobenzylidene)benzo[*b*]thiophen-3(2*H*)-one 26: To a flame-dried 5 mL microwave vial **25** (1.25 eq), *p*-iodobenzaldehyde (1.1 mmol, 1 eq) and 1mL DMF were added. Then, piperidine (0.2 eq) was added to the solution. The reaction was stirred for five hours and then diluted with chloroform (20 mL) and washed with saturated aqueous NH_4Cl (50 mL) and H_2O (100 mL). The aqueous layer was then extracted with chloroform. The combined organic layers were then washed with brine (60 mL) and dried over anhydrous Na_2SO_4, filtered and concentrated. Purification by flash column chromatography. A yellow solid was obtained in 33% yield. ^{1}H-NMR (300 MHz, Chloroform-*d*): *δ* 7.95 (ddd, J = 7.8, 1.4, 0.7 Hz, 1H), 7.89 - 7.78 (m, 3H), 7.60 (ddd, J = 8.5, 7.1, 1.4 Hz, 1H), 7.51 (dt, J = 8.0, 1.0 Hz, 1H), 7.47 - 7.37 (m, 2H), 7.32 (ddd, J = 8.1, 7.1, 1.0 Hz, 1H). ^{13}C NMR (101 MHz, $CDCl_3$) δ 138.5, 135.7, 132.4, 132.3, 127.3, 126.0, 124.1. HRMS (EI) m/z calculated for $[C_{15}H_9IOS]^{\bullet+}$ $[M]^{\bullet+}$ 363.9414, found 363.9409.

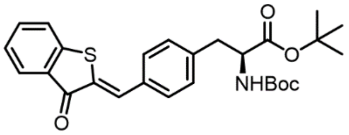

***tert*-butyl (*S*,*Z*)-2-((*tert*-butoxycarbonyl)amino)-3-(4-((3-oxobenzo[*b*]-thiophen-2(3*H*)-ylidene)methyl)phenyl)propanoate 27**: Same procedure as **14** but with **26** (0.33 mmol, 1 eq) instead of **13**. Yellow oil was obtained in 76% yield. ^{1}H-NMR (400 MHz, Chloroform-*d*): *δ* 7.97 - 7.90 (m, 2H), 7.68 - 7.61 (m, 2H), 7.58 (ddd, J = 8.3, 7.1, 1.4 Hz, 1H), 7.53 - 7.48 (m, 1H), 7.34 - 7.27 (m, 3H), 5.05 (d, J = 8.1 Hz, 1H), 4.55 - 4.42 (m, 1H), 3.12 (h, J = 7.9, 7.2 Hz, 2H), 1.42 (d, J = 6.4 Hz, 18H). ^{13}C NMR (101 MHz, $CDCl_3$) δ 189.1, 146.5, 135.7, 133.8, 131.5, 131.0, 130.8, 127.5, 126.1, 124.4, 82.8, 80.3, 55.1, 39.1, 28.8, 28.4. HRMS (ESI-TOF) m/z calculated for $[C_{27}H_{31}NO_6SNa]^+$ $[MNa]^+$ 504.1816, found 504.1821.

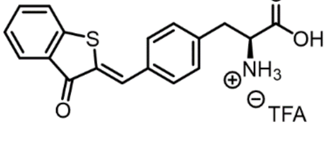

(*S*,*Z*)-2-amino-3-(4-((3-oxobenzo[*b*]thiophen-2(3*H*)-ylidene)methyl) phenyl)propanoic acid HtiF: **27** (0.25 mmol, 1 eq) was dissolved in 5 mL DCM and excess TFA was added before the solution was stirred at rt for 2 days. The solvent was removed *in vacuo* and the residue was dissolved in a minimum amount of water, followed by lyophilization. The compound was then triturated with Et_2O and dried *in vacuo* to obtain the final product as a TFA salt. Orange solid in 73% yield. ^{1}H-NMR (400 MHz, DMSO-*d*$_6$): *δ* 7.95 (s, 1H), 7.92 - 7.86 (m, 1H), 7.83 - 7.79 (m, 1H), 7.79 - 7.72 (m, 3H), 7.50 - 7.45 (m, 2H),

7.43 (td, J = 7.2, 1.2 Hz, 1H), 4.10 (t, J = 6.6 Hz, 1H), 3.23 - 3.07 (m, 2H). ^{13}C-NMR (101 MHz, DMSO-*d₆*): *δ* 136.1, 131.1, 130.5, 129.6, 129.5, 126.6, 124.6. HRMS (ESI-TOF) m/z calculated for $[C_{18}H_{16}NO_3S]^+$ $[MH]^+$ 326.0846, found 326.0847.

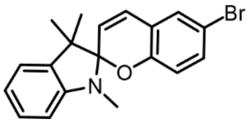

6-bromo-1',3',3'-trimethylspiro[chromene-2,2'-indoline] 30: To a solution of 1,3,3-trimethyl-2-methyleneindoline (1 eq) in EtOH, was added 5-bromosalicylaldehyde. The reaction mixture was refluxed overnight before being concentrated *in vacuo*. The crude product was purified by flash column chromatography to obtain a red oil in quantitative yield. ^{1}H-NMR (300 MHz, Chloroform-*d*): *δ* 7.22 - 7.13 (m, 3H), 7.07 (ddd, J = 7.3, 1.4, 0.5 Hz, 1H), 6.85 (td, J = 7.4, 1.0 Hz, 1H), 6.78 (dd, J = 10.3, 0.8 Hz, 1H), 6.60 (dd, J = 9.1, 0.8 Hz, 1H), 6.56 - 6.48 (m, 1H), 5.73 (d, J = 10.3 Hz, 1H), 2.72 (s, 3H), 1.29 (s, 3H), 1.16 (s, 3H). Data matches with existing literature.[41]

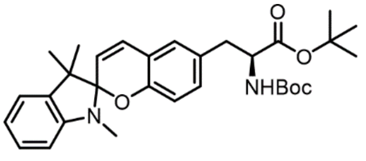

***tert*-butyl (2*S*)-2-((*tert*-butoxycarbonyl)amino)-3-(1',3',3'-trimethylspiro[chromene-2,2'-indolin]-6-yl)propanoate 31**: Same procedure as **14** but with **30** (1.3 mmol, 1 eq) instead of **13**. Red oil was obtaned in 71% yield. ^{1}H-NMR (400 MHz, Chloroform-*d*): *δ* 7.18 (td, J = 7.5, 1.2 Hz, 1H), 7.07 (dd, J = 7.2, 1.4 Hz, 1H), 6.93 - 6.82 (m, 3H), 6.80 (d, J = 10.4 Hz, 1H), 6.63 (d, J = 8.0 Hz, 1H), 6.52 (d, J = 7.0 Hz, 1H), 5.67 (d, J = 10.2 Hz, 1H), 5.01 (d, J = 8.1 Hz, 1H), 4.39 (q, J = 6.5 Hz, 1H), 2.94 (d, J = 6.2 Hz, 2H), 2.72 (d, J = 2.1 Hz, 3H), 1.43 (s, 9H), 1.40 (s, 9H), 1.29 (s, 3H), 1.16 (s, 3H). ^{13}C NMR (101 MHz, $CDCl_3$) δ 155.2, 153.6, 148.3, 136.9, 130.9, 130.9, 129.4, 127.9, 127.8, 127.7, 127.7, 121.6, 119.7, 119.2, 118.7, 115.0, 106.9, 104.3, 82.1, 79.8, 55.1, 51.8, 38.0, 29.0, 29.0, 28.5, 28.1, 26.0, 20.3. HRMS (ESI-TOF) m/z calculated for $[C_{31}H_{41}N_2O_5]^+$ $[MH]^+$ 521.3010, found 521.3037.

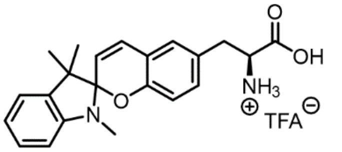

(2*S*)-2-amino-3-(1',3',3'-trimethylspiro[chromene-2,2'-indolin]-6-yl) propanoic acid SpiroY: 31 (0.1 mmol, 1 eq) was dissolved in 5 mL DCM and excess TFA is added before the solution was stirred at rt for 2 days. The solvent was removed *in vacuo* and the residue was dissolved in a minimum amount of water followed by lyophilization. The compound was then triturated with Et_2O and dried *in vacuo* to obtain the final product. The product was a mixture of spyropyran TFA salt and merocyanine double TFA salt. Red solid was obtained in quantitative yield. ^{1}H-NMR (400 MHz, DMSO-*d₆*): *δ*. (spiropyran) 8.38 - 8.12 (m, 3H), 7.13 - 7.02 (m, 3H), 6.97 (td, J = 7.0, 6.0, 3.4 Hz, 2H), 6.77 (t, J = 7.3 Hz, 1H), 6.65 (d, J = 8.3 Hz, 1H), 6.55 (d, J = 7.7 Hz, 1H), 5.77 (d, J = 10.3 Hz, 1H), 4.17 - 4.11 (m, 1H), 3.09 - 2.88 (m, 2H), 2.64 (d, J = 3.4 Hz, 3H), 1.21 (d, J = 2.7 Hz, 3H), 1.09 (s, 3H). (merocyanine) 8.47 (d, J = 16.4 Hz, 1H), 8.42 (s, 3H), 8.00 (d, J = 2.3 Hz, 1H), 7.95 - 7.82 (m, 2H), 7.73 - 7.56 (m, 3H), 7.35 (dd, J = 8.5, 2.2 Hz, 1H), 7.04 (d, J = 8.5 Hz, 1H), 4.20 (s, 1H), 4.09 (s, 3H), 3.19 - 3.07 (m, 2H), 1.76 (s, 6H). ^{13}C-NMR (101 MHz, DMSO-*d₆*): *δ*

181.7, 170.5, 170.4, 158.5, 158.4, 158.1, 153.2, 148.4, 147.8, 143.2, 141.9, 136.5, 136.3, 130.9, 129.3, 129.2, 129.0, 128.0, 127.5, 126.4, 122.9, 121.5, 121.2, 119.6, 119.0, 118.6, 118.4, 117.1, 115.1, 114.6, 112.0, 106.8, 104.0, 88.7, 53.2, 51.9, 51.3, 35.0, 34.2, 28.6, 26.0, 25.7, 20.0. HRMS (ESI-TOF) m/z calculated for $[C_{22}H_{25}N_2O_3]^+$ $[MH]^+$ 365.1860, found 365.1862 (z=1), 183.0973 (z=2).

2.1.3 ^{1}H-NMR Spectra

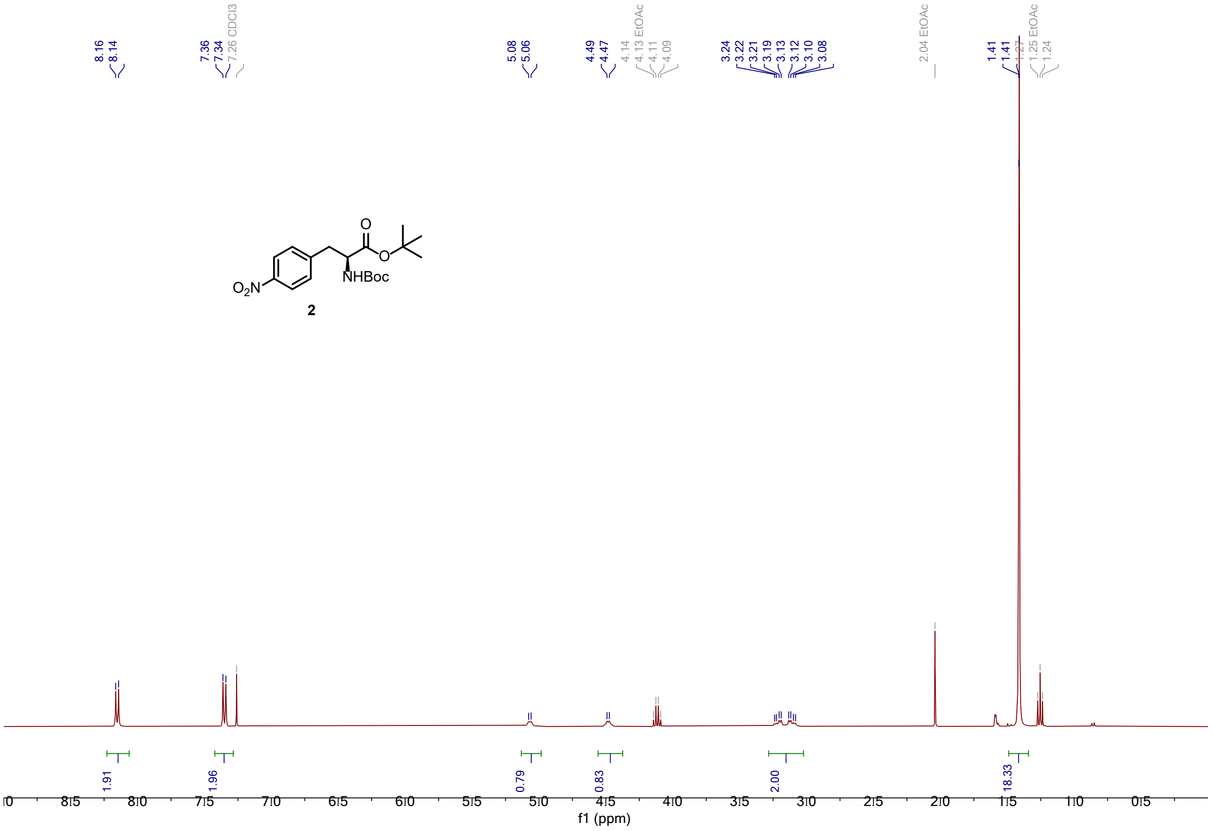

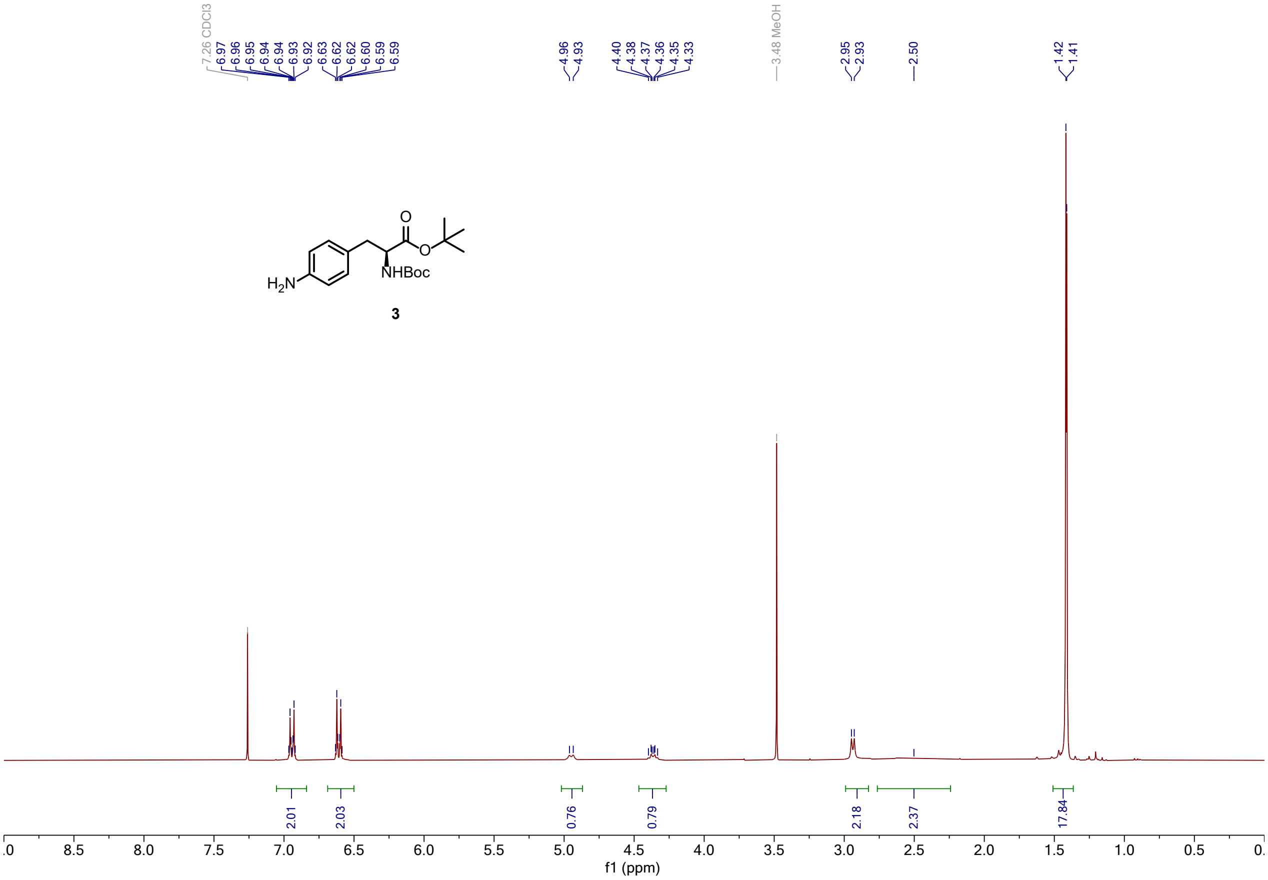

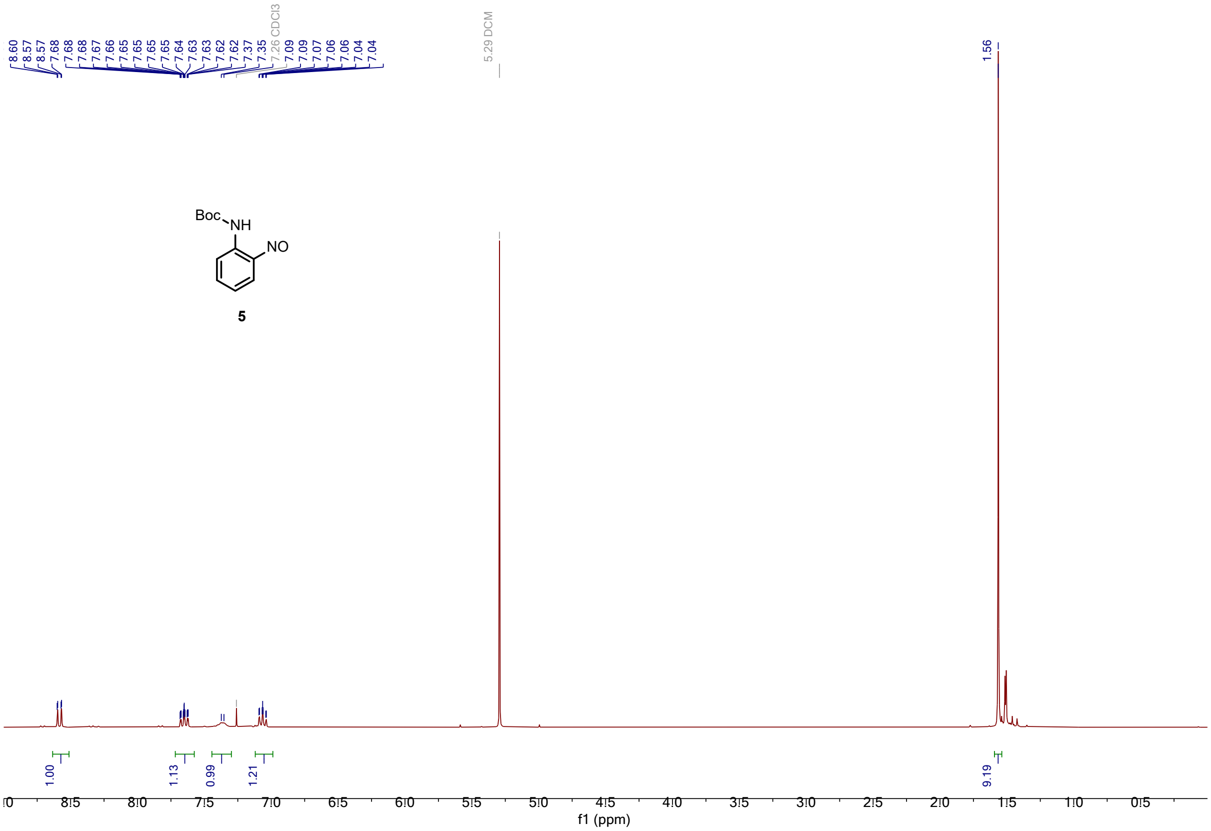

5.29 DCM
7.26 CDCl3
Boc
NH
NO
5
f1 (ppm)

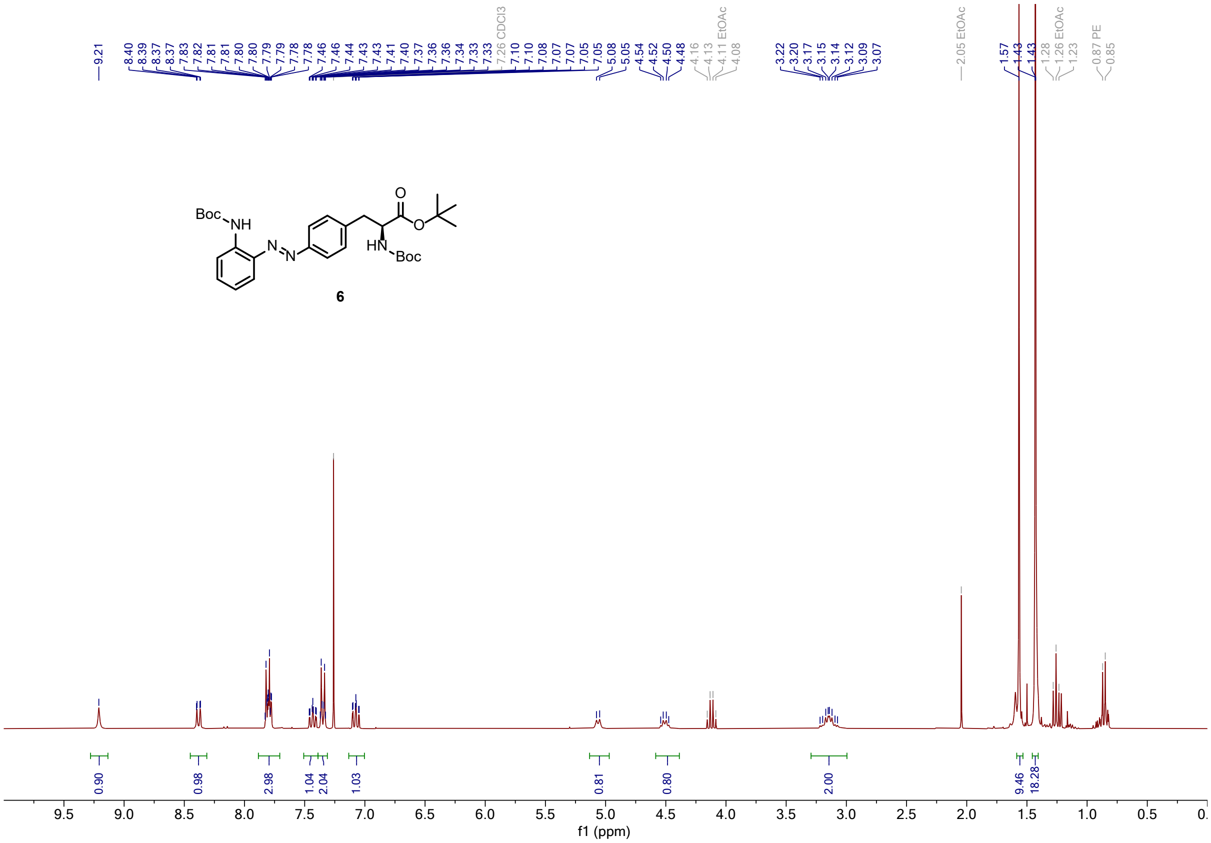

7.26 CDCl3
4.11 EtOAc
2.05 EtOAc
1.26 EtOAc
0.87 PE
Boc
NH
HN
Boc
O
6
f1 (ppm)

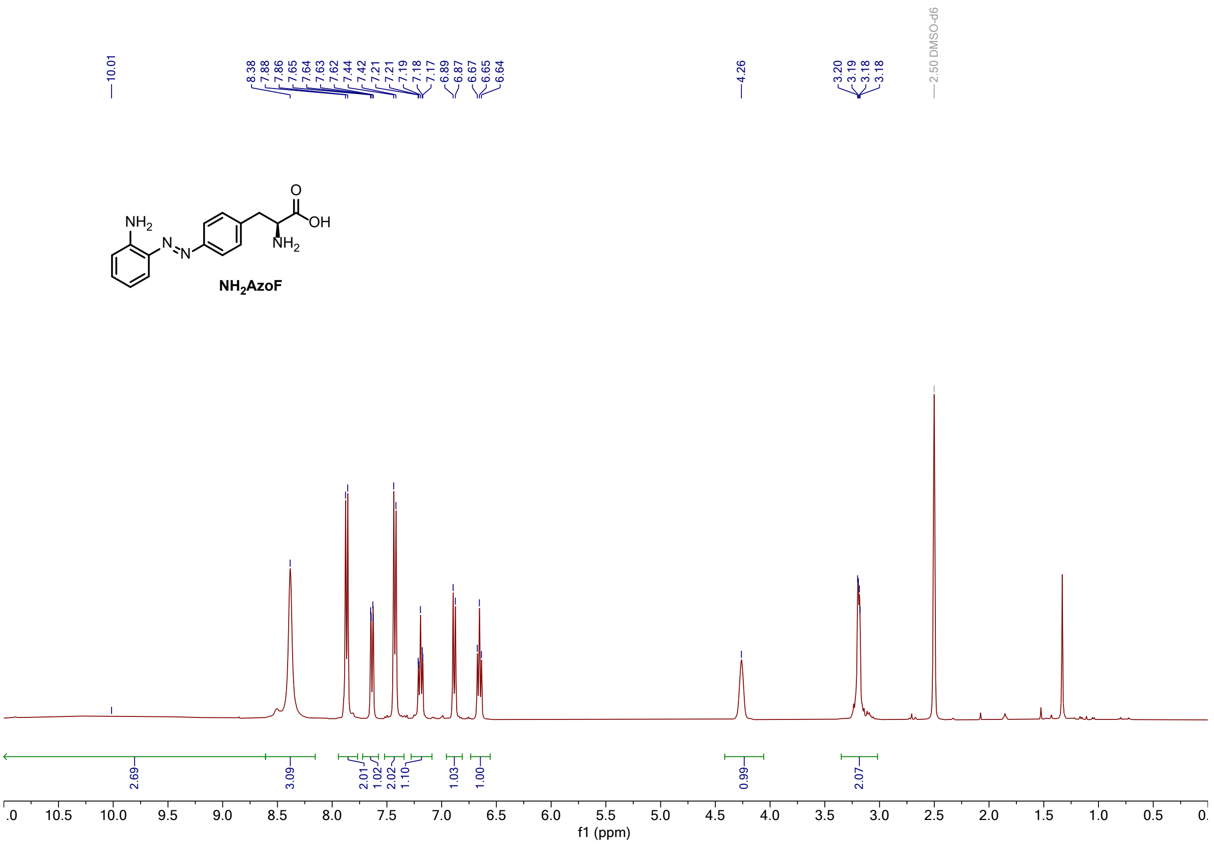
10.01
8.38
7.88
7.86
7.65
7.64
7.63
7.62
7.44
7.42
7.21
7.21
7.19
7.18
7.17
6.89
6.87
6.67
6.65
6.64
4.26
3.20
3.19
3.18
3.18
2.50 DMSO-d6
NH2
N
N
O
OH
NH2
NH2AzoF
2.69
3.09
2.01
1.02
2.02
1.10
1.03
1.00
0.99
2.07
f1 (ppm)

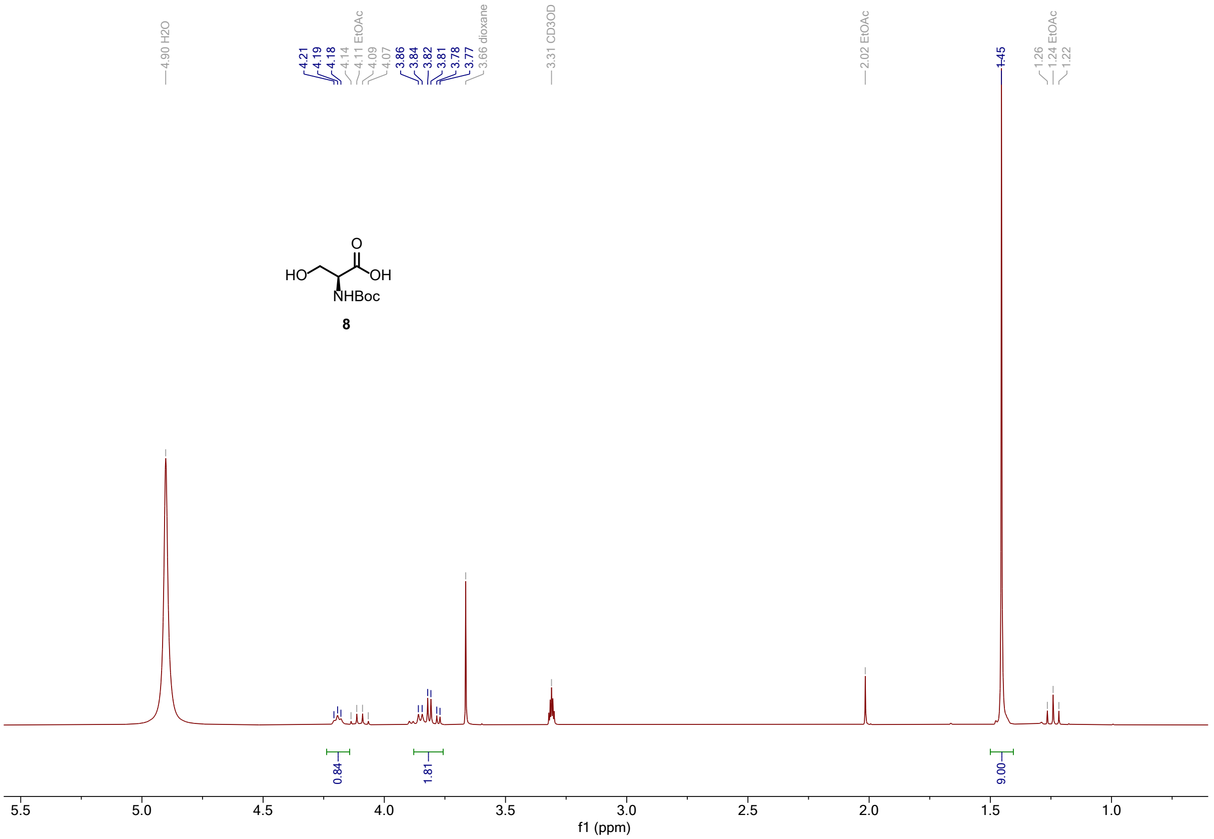
4.90 H2O
4.21
4.19
4.18
4.14
4.11 EtOAc
4.09
4.07
3.86
3.84
3.82
3.81
3.78
3.77
3.66 dioxane
3.31 CD3OD
2.02 EtOAc
1.45
1.26
1.24 EtOAc
1.22
HO
O
OH
NHBoc
8
0.84
1.81
9.00
f1 (ppm)

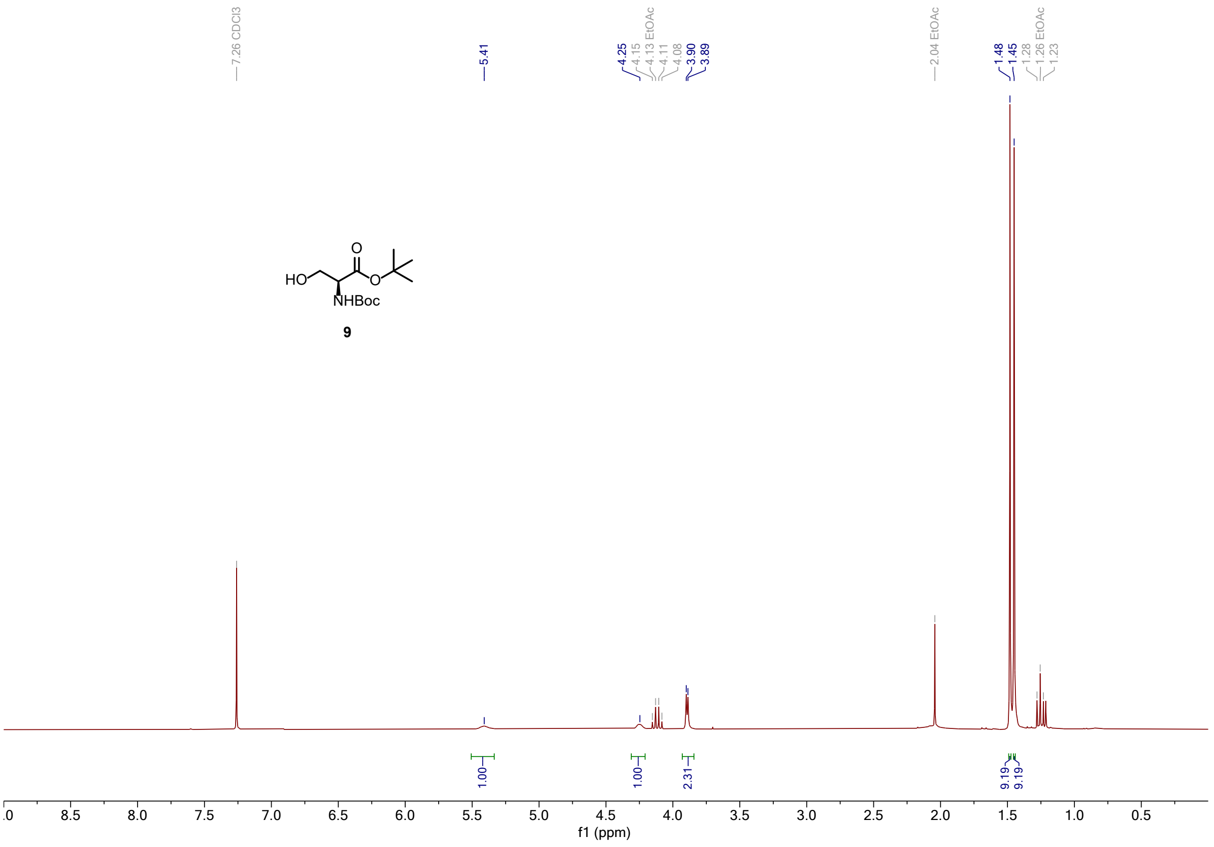
7.26 CDCl3
5.41
4.25
4.15
4.13 EtOAc
4.11
4.08
3.90
3.89
2.04 EtOAc
1.48
1.45
1.28
1.26 EtOAc
1.23
HO
O
O
NHBoc
9
1.00
1.00
2.31
9.19
9.19
.0
8.5
8.0
7.5
7.0
6.5
6.0
5.5
5.0
4.5
4.0
3.5
3.0
2.5
2.0
1.5
1.0
0.5
f1 (ppm)

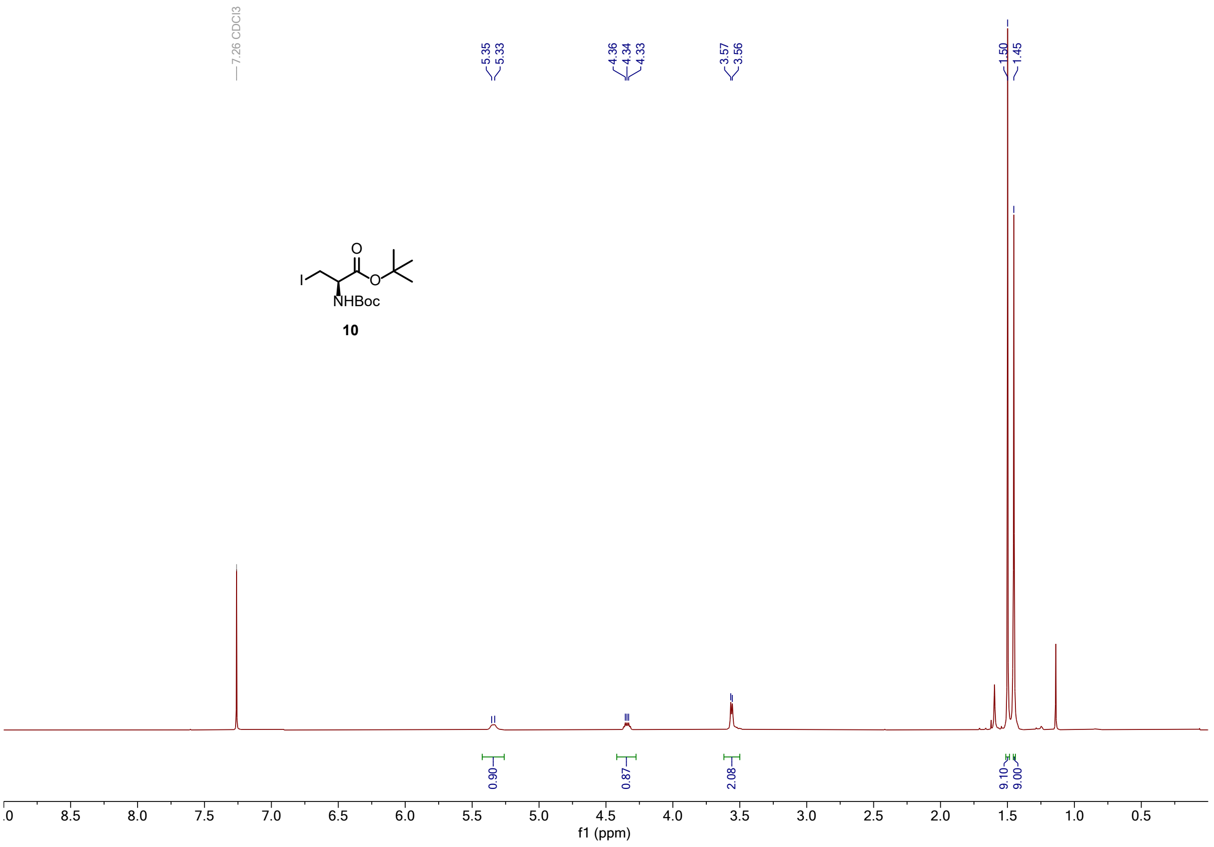
7.26 CDCl3
5.35
5.33
4.36
4.34
4.33
3.57
3.56
1.50
1.45
I
O
O
NHBoc
10
0.90
0.87
2.08
9.10
9.00
.0
8.5
8.0
7.5
7.0
6.5
6.0
5.5
5.0
4.5
4.0
3.5
3.0
2.5
2.0
1.5
1.0
0.5
f1 (ppm)

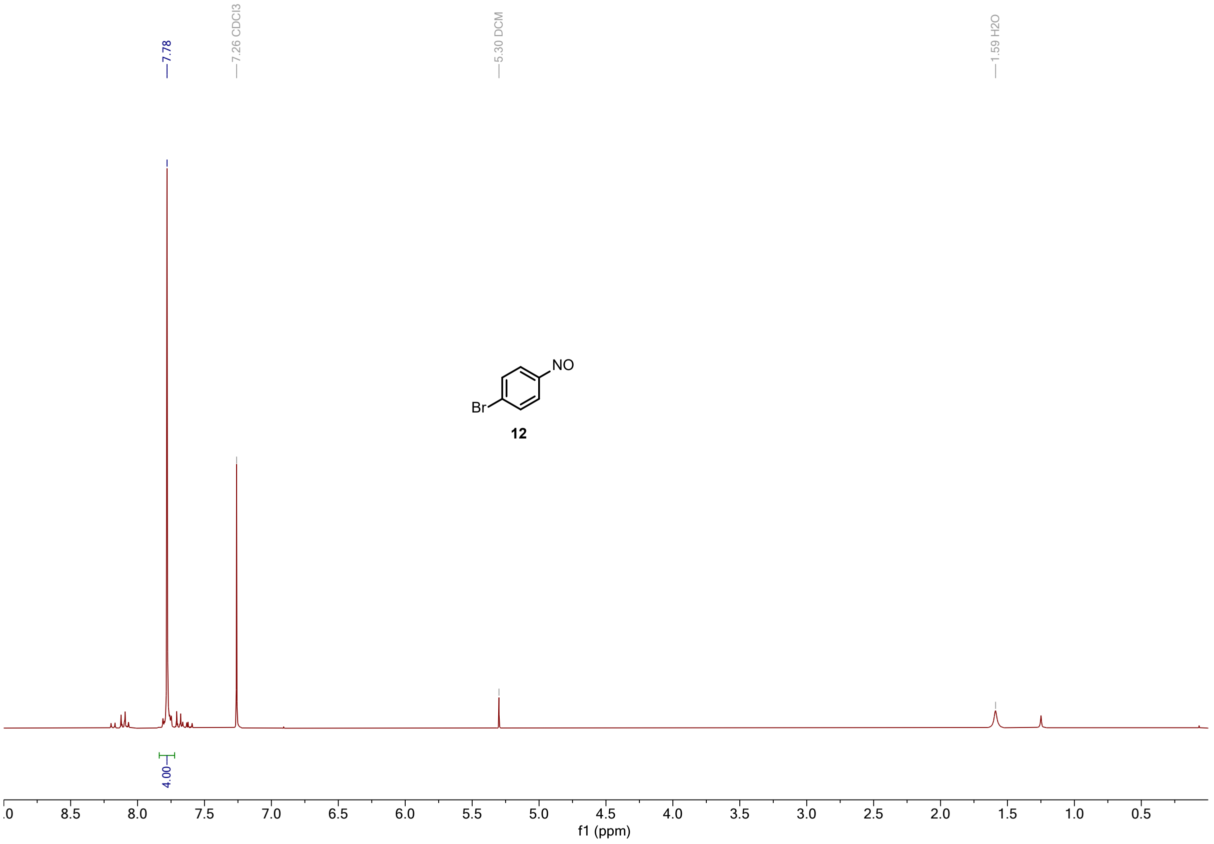

7.78
7.26 CDCl3
5.30 DCM
1.59 H2O
NO
Br
4.00
.0 8.5 8.0 7.5 7.0 6.5 6.0 5.5 5.0 4.5 4.0 3.5 3.0 2.5 2.0 1.5 1.0 0.5
f1 (ppm)

12

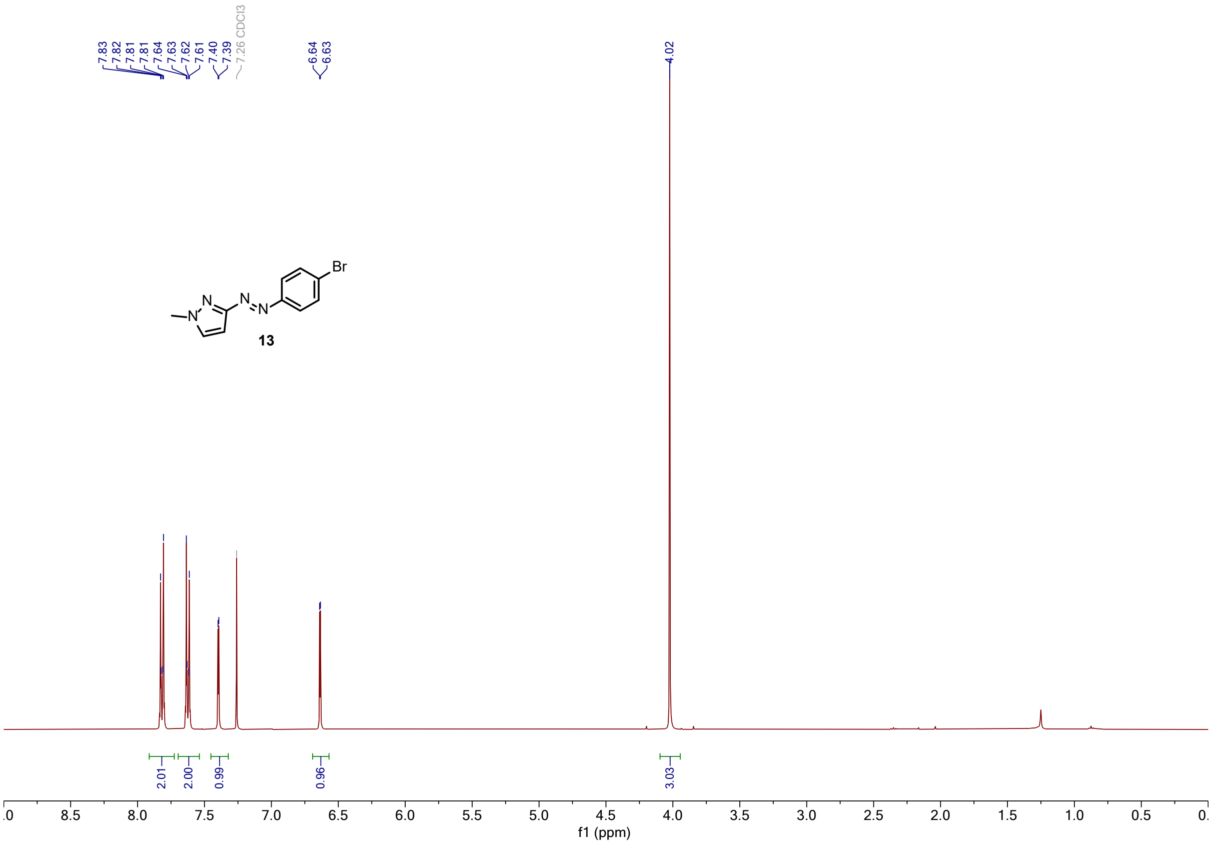

7.83 7.82 7.81 7.81 7.64 7.63 7.62 7.61 7.40 7.39
7.26 CDCl3
6.64 6.63
4.02
N N N N Br
2.01 2.00 0.99 0.96 3.03
.0 8.5 8.0 7.5 7.0 6.5 6.0 5.5 5.0 4.5 4.0 3.5 3.0 2.5 2.0 1.5 1.0 0.5 0.
f1 (ppm)

13

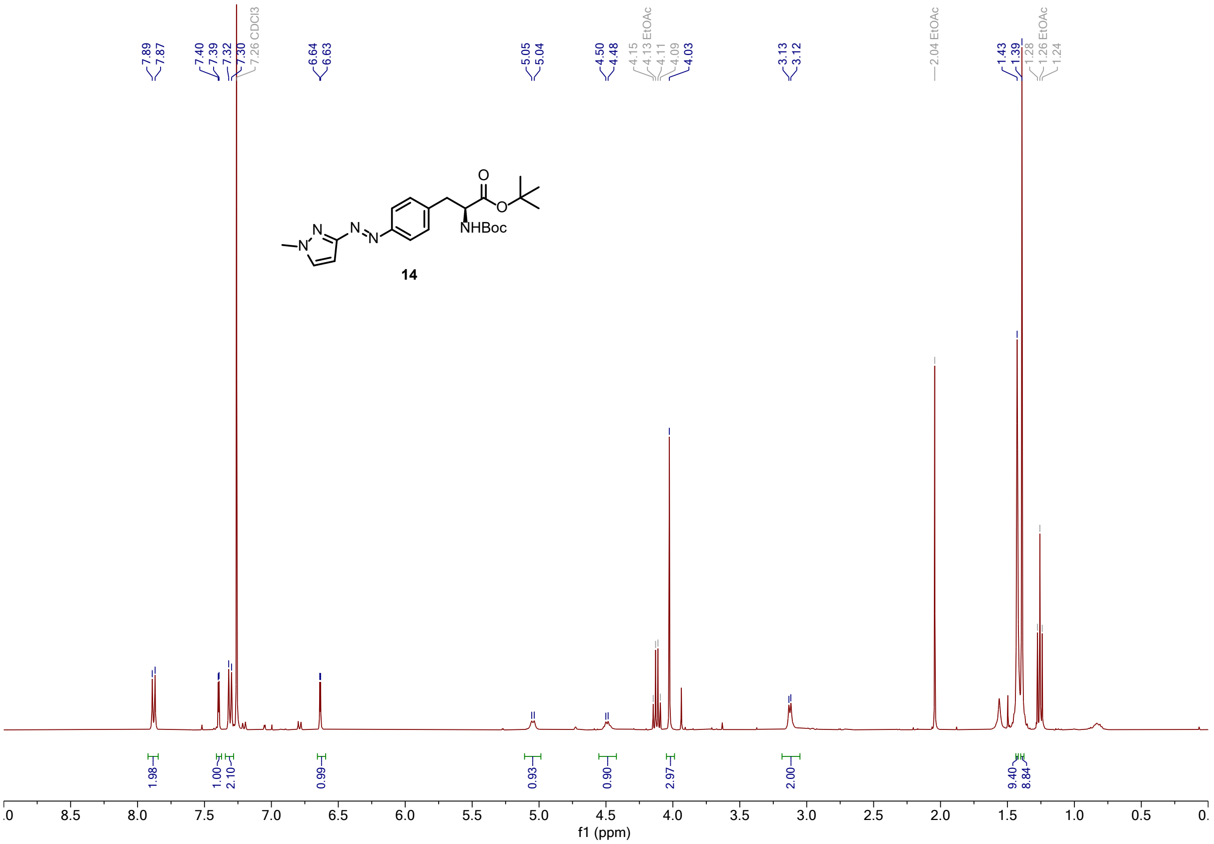
NHBoc
14
f1 (ppm)

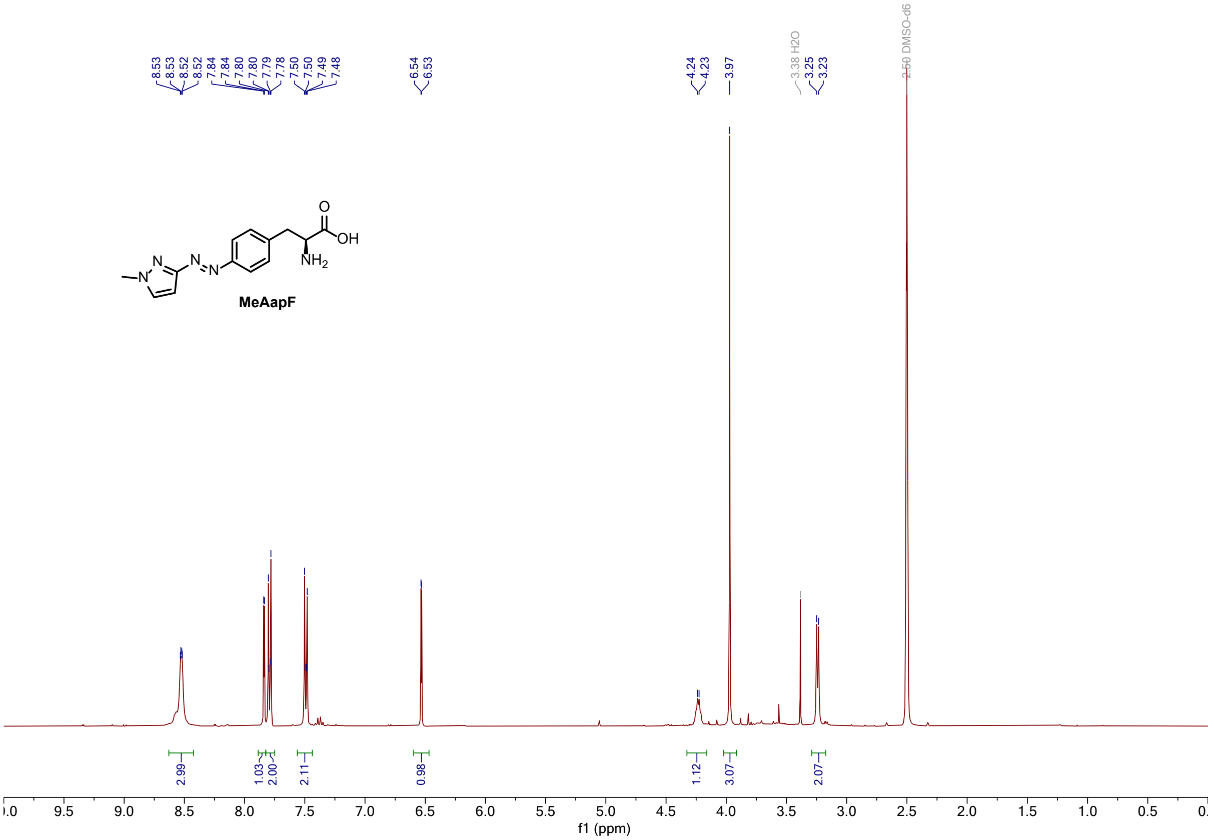
OH
NH2
MeAapF
f1 (ppm)

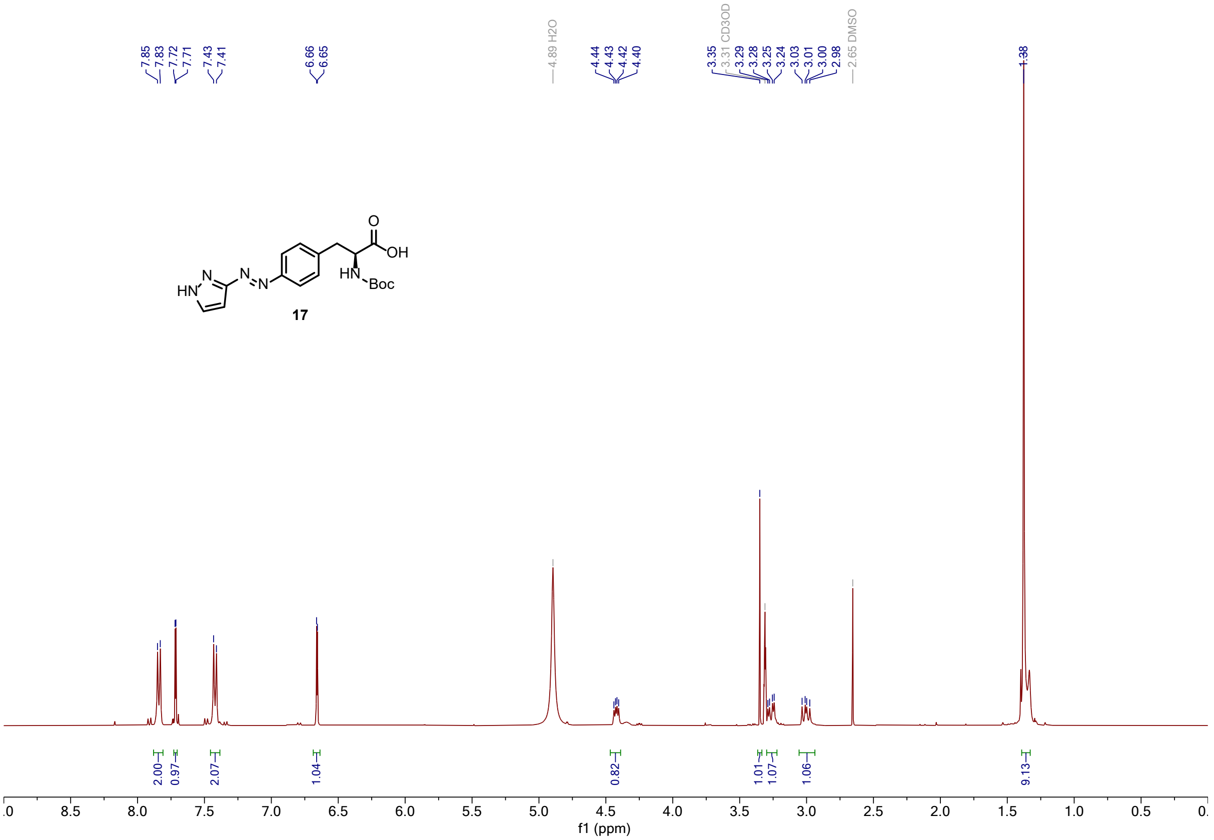
7.85 7.83 7.72 7.71 7.43 7.41
6.66 6.65
4.89 H2O
4.44 4.43 4.42 4.40
3.35 3.31 CD3OD 3.29 3.28 3.25 3.24 3.03 3.01 3.00 2.98
2.65 DMSO
1.38
17
2.00 0.97 2.07 1.04 0.82 1.01 1.07 1.06 9.13
f1 (ppm)

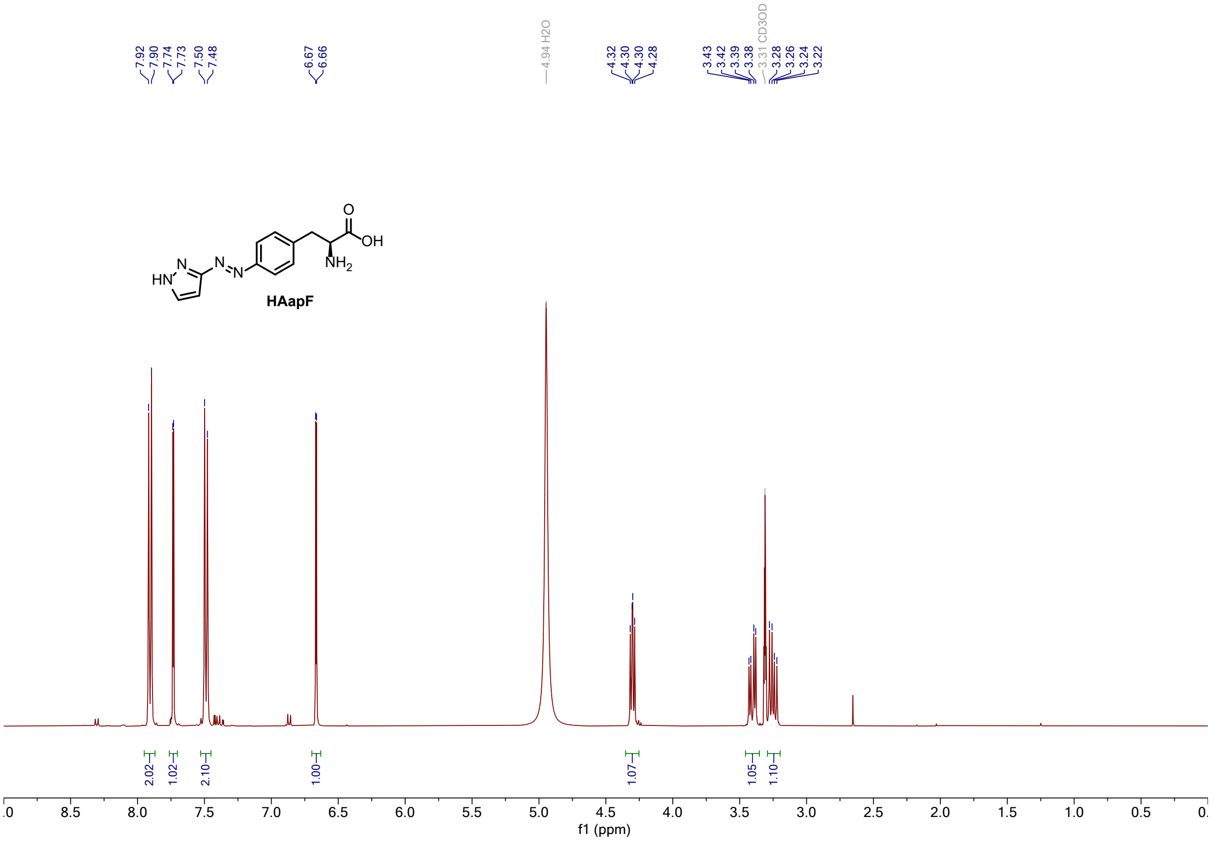
7.92 7.90 7.74 7.73 7.50 7.48
6.67 6.66
4.94 H2O
4.32 4.30 4.30 4.28
3.43 3.42 3.39 3.38 3.31 CD3OD 3.28 3.26 3.24 3.22
HAapF
2.02 1.02 2.10 1.00 1.07 1.05 1.10
f1 (ppm)

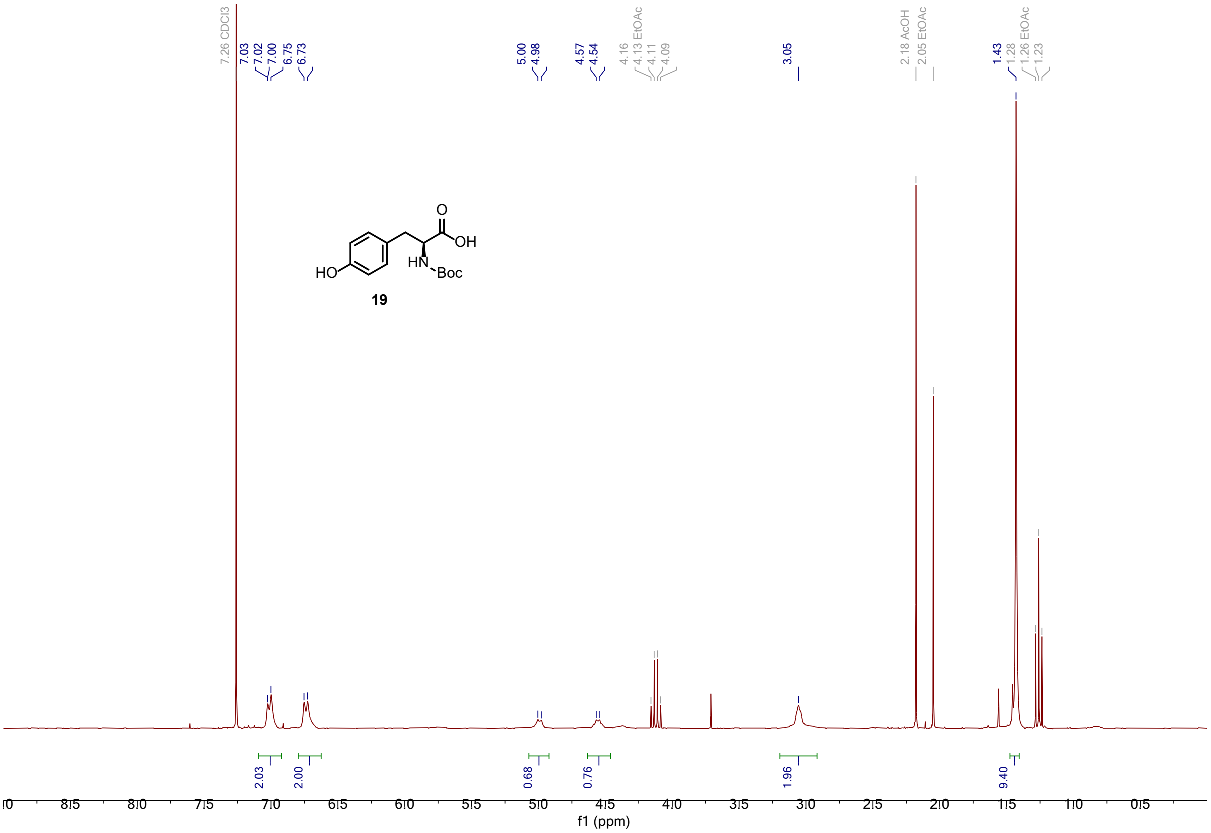
7.26 CDCl3
7.03
7.02
7.00
6.75
6.73
5.00
4.98
4.57
4.54
4.16
4.13 EtOAc
4.11
4.09
3.05
2.18 AcOH
2.05 EtOAc
1.43
1.28
1.26 EtOAc
1.23
OH
O
HO
HN
Boc
19
2.03
2.00
0.68
0.76
1.96
9.40
f1 (ppm)

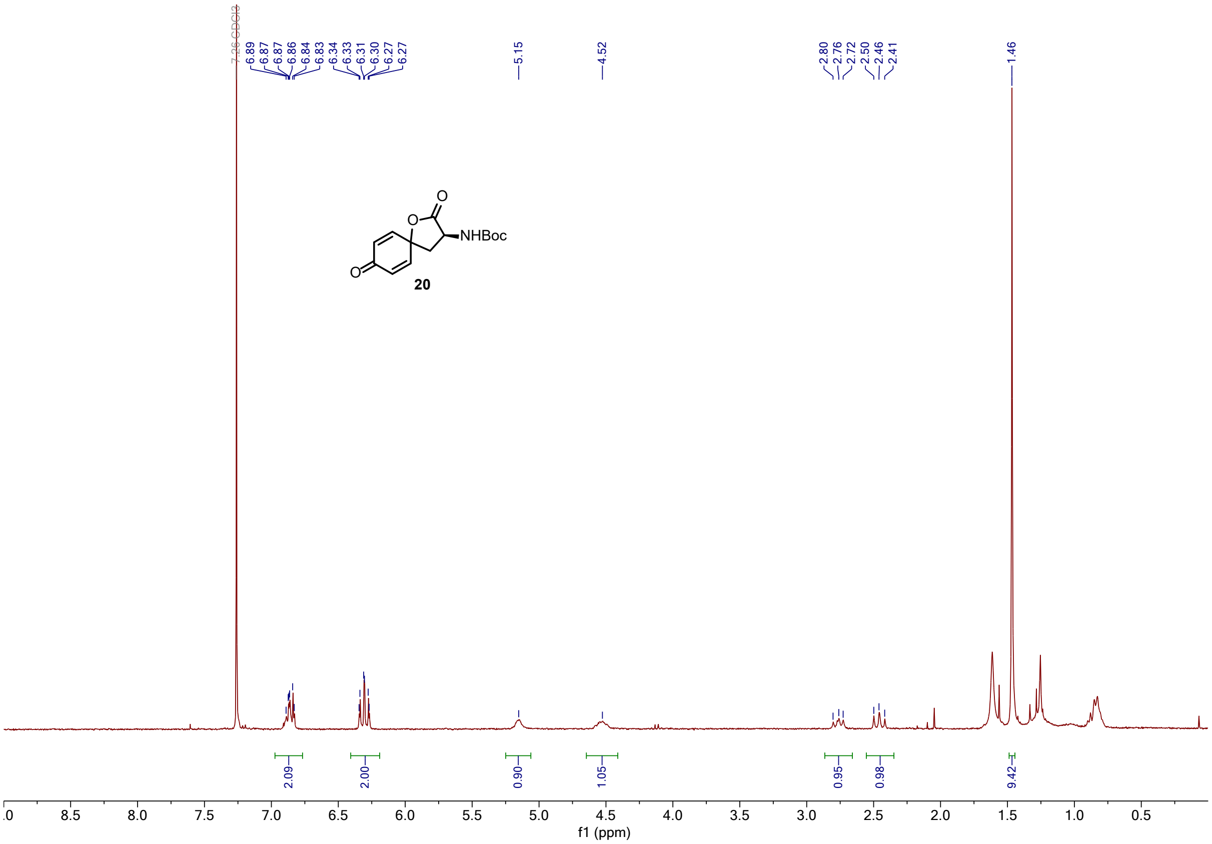
7.26 CDCl3
6.89
6.87
6.87
6.86
6.84
6.83
6.34
6.33
6.31
6.30
6.27
6.27
5.15
4.52
2.80
2.76
2.72
2.50
2.46
2.41
1.46
O
O
O
NHBoc
20
2.09
2.00
0.90
1.05
0.95
0.98
9.42
f1 (ppm)

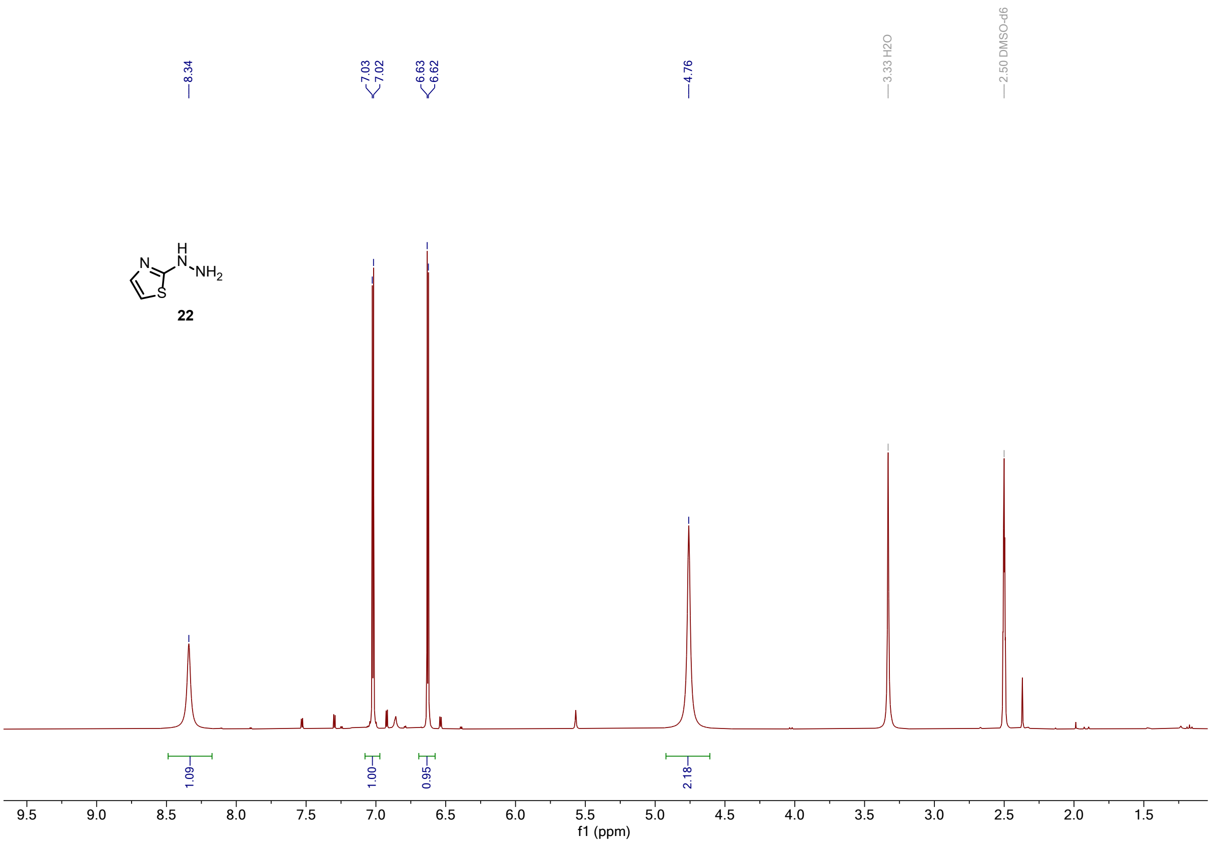
8.34
7.03
7.02
6.63
6.62
4.76
3.33 H2O
2.50 DMSO-d6
H
N
NH2
N
S
22
1.09
1.00
0.95
2.18
9.5 9.0 8.5 8.0 7.5 7.0 6.5 6.0 5.5 5.0 4.5 4.0 3.5 3.0 2.5 2.0 1.5
f1 (ppm)

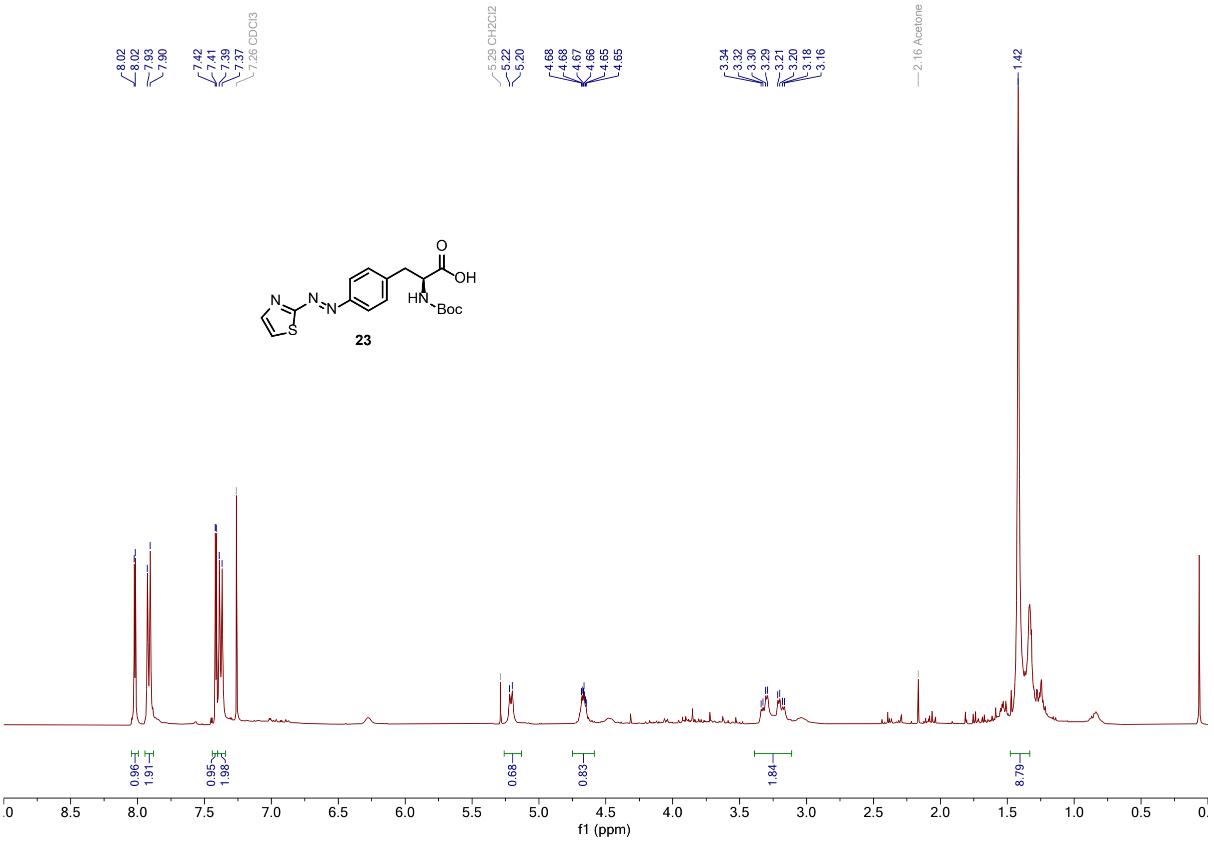
8.02
8.02
7.93
7.90
7.42
7.41
7.39
7.37
7.26 CDCl3
5.29 CH2Cl2
5.22
5.20
4.68
4.68
4.67
4.66
4.65
4.65
3.34
3.32
3.30
3.29
3.21
3.20
3.18
3.16
2.16 Acetone
1.42
O
OH
N
N
N
S
HN
Boc
23
0.96
1.91
0.95
1.98
0.68
0.83
1.84
8.79
8.5 8.0 7.5 7.0 6.5 6.0 5.5 5.0 4.5 4.0 3.5 3.0 2.5 2.0 1.5 1.0 0.5
f1 (ppm)

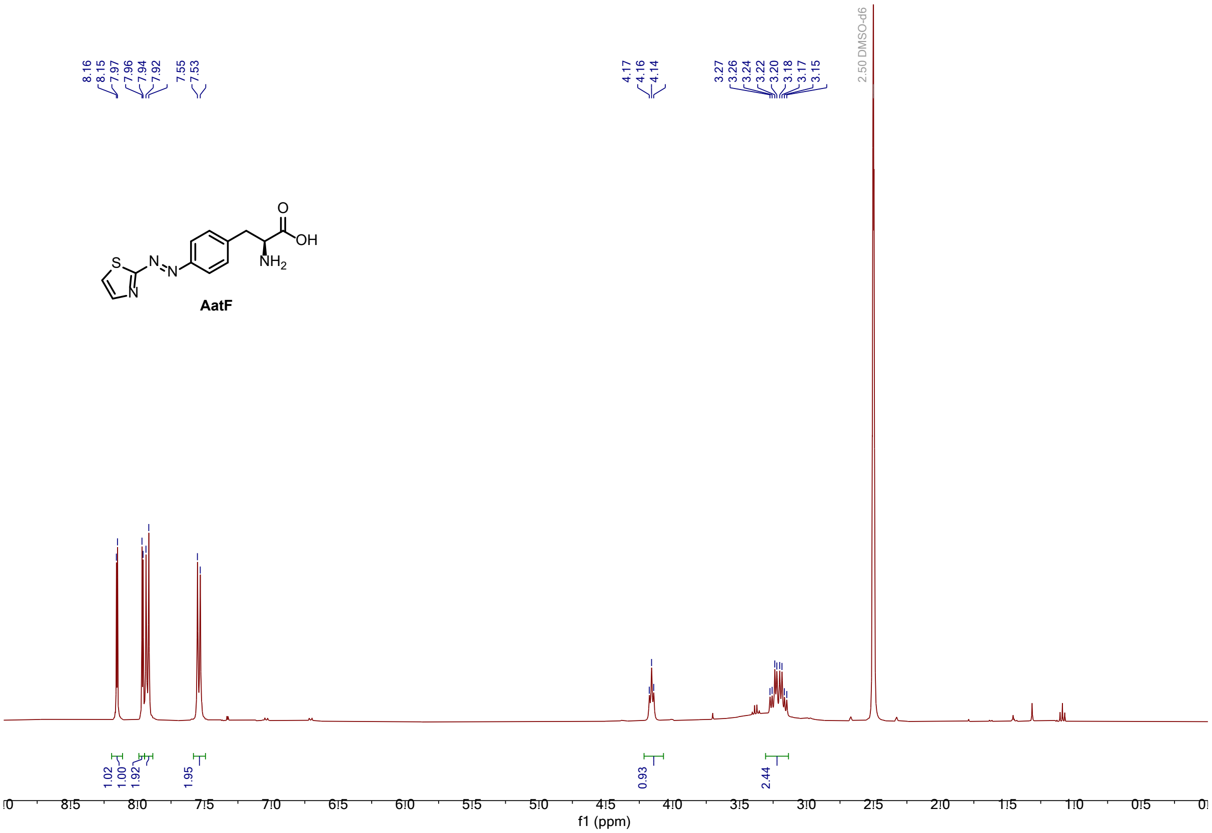
8.16
8.15
7.97
7.96
7.94
7.92
7.55
7.53
4.17
4.16
4.14
3.27
3.26
3.24
3.22
3.20
3.18
3.17
3.15
2.50 DMSO-d6
AatF
1.02
1.00
1.92
1.95
0.93
2.44
f1 (ppm)

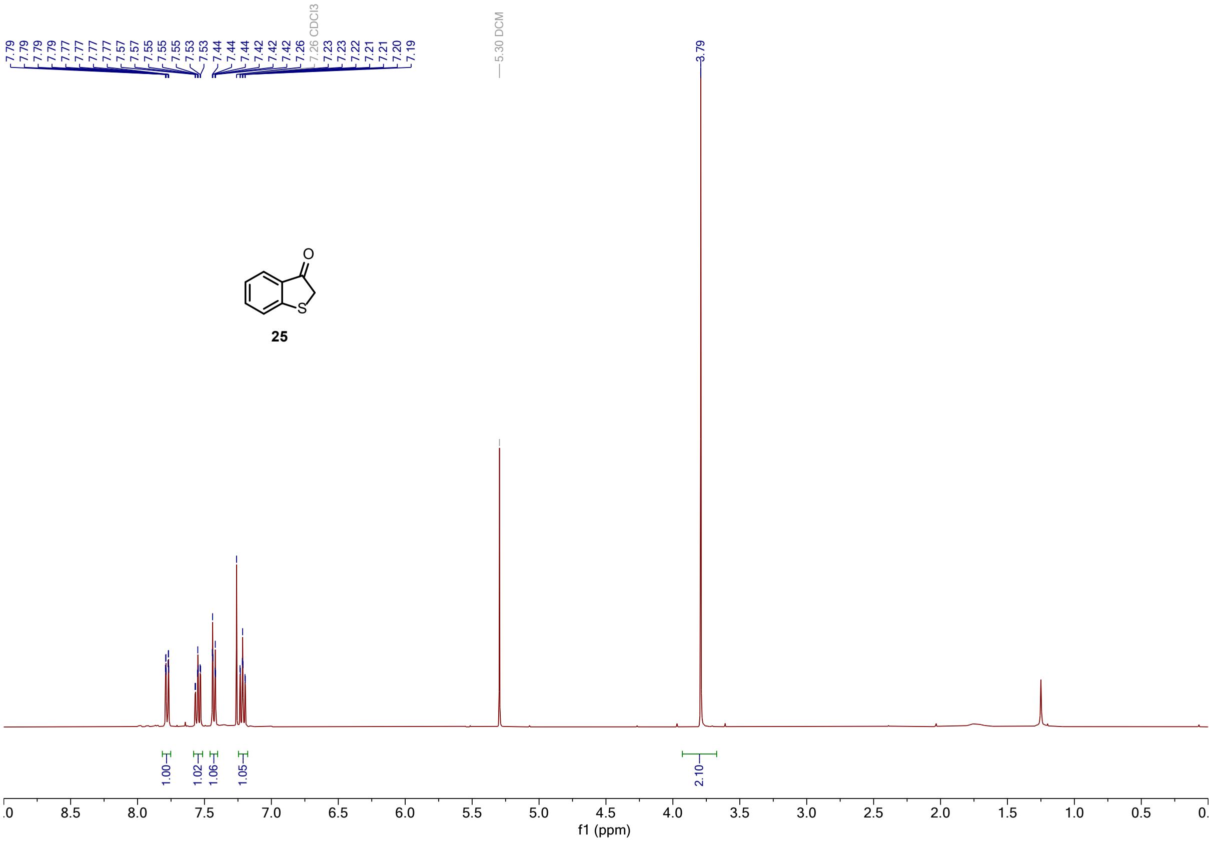
7.79
7.79
7.79
7.79
7.77
7.77
7.77
7.77
7.57
7.57
7.55
7.55
7.53
7.53
7.44
7.44
7.44
7.42
7.42
7.42
7.26 CDCl3
7.23
7.23
7.22
7.21
7.21
7.20
7.19
5.30 DCM
3.79
25
1.00
1.02
1.06
1.05
2.10
f1 (ppm)

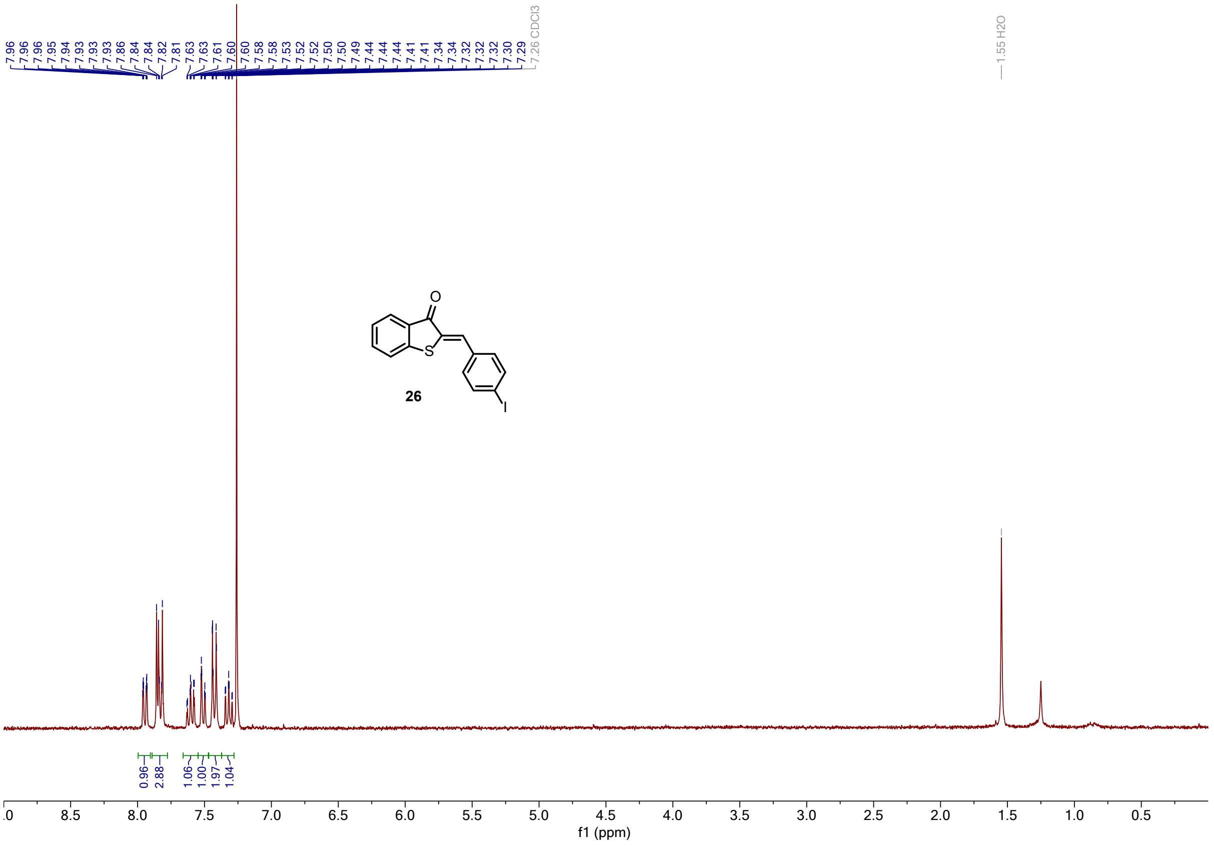

7.26 CDCl3
1.55 H2O
26
0.96
2.88
1.06
1.00
1.97
1.04
f1 (ppm)

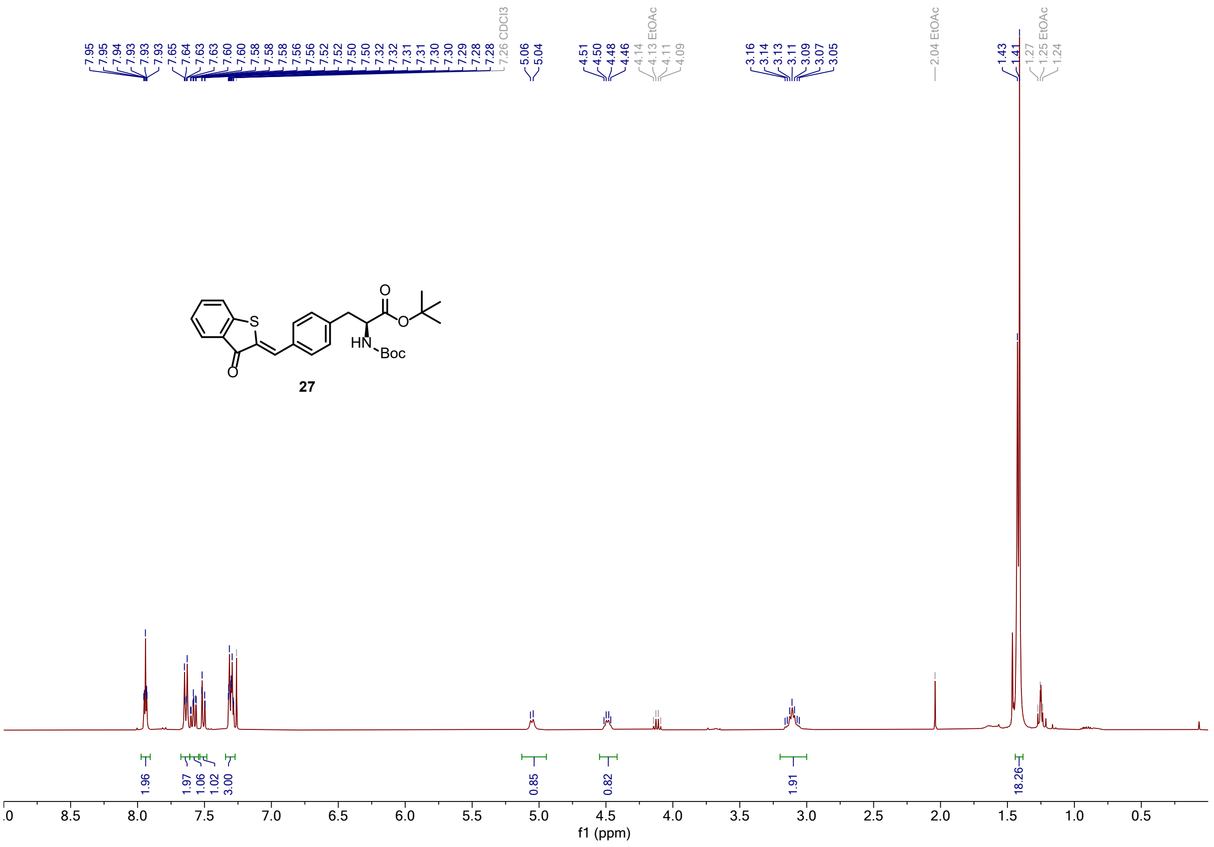

7.26 CDCl3
4.13 EtOAc
2.04 EtOAc
1.25 EtOAc
HN
Boc
27
1.96
1.97
1.06
1.02
3.00
0.85
0.82
1.91
18.26
f1 (ppm)

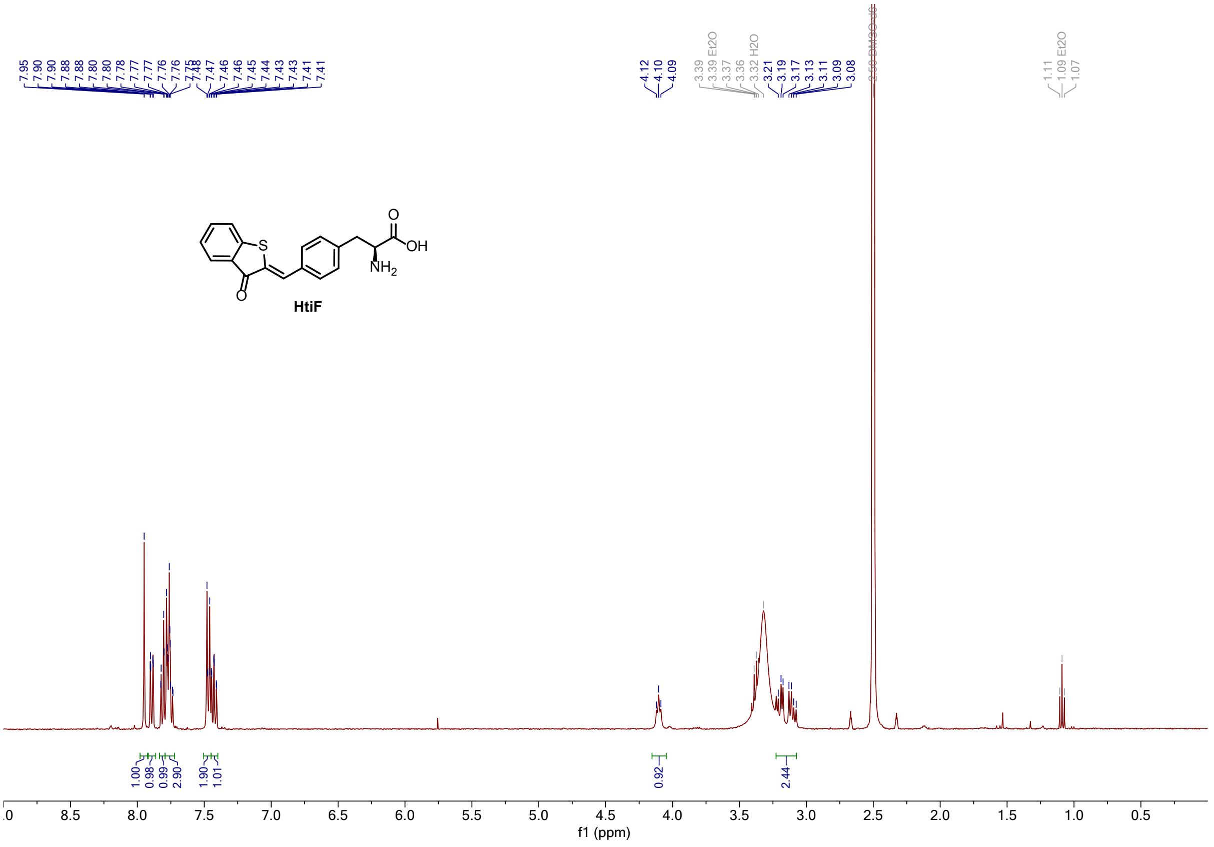
HtiF
f1 (ppm)

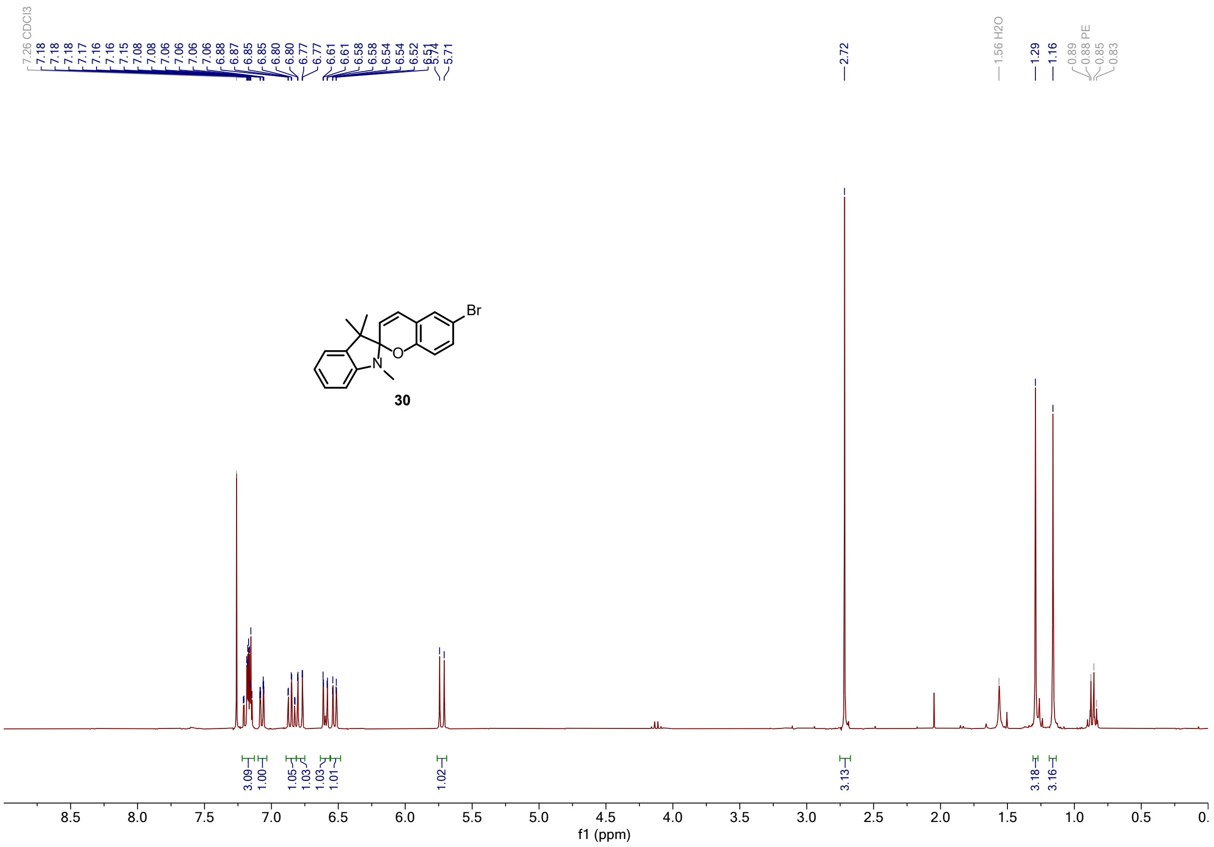
30
Br
f1 (ppm)

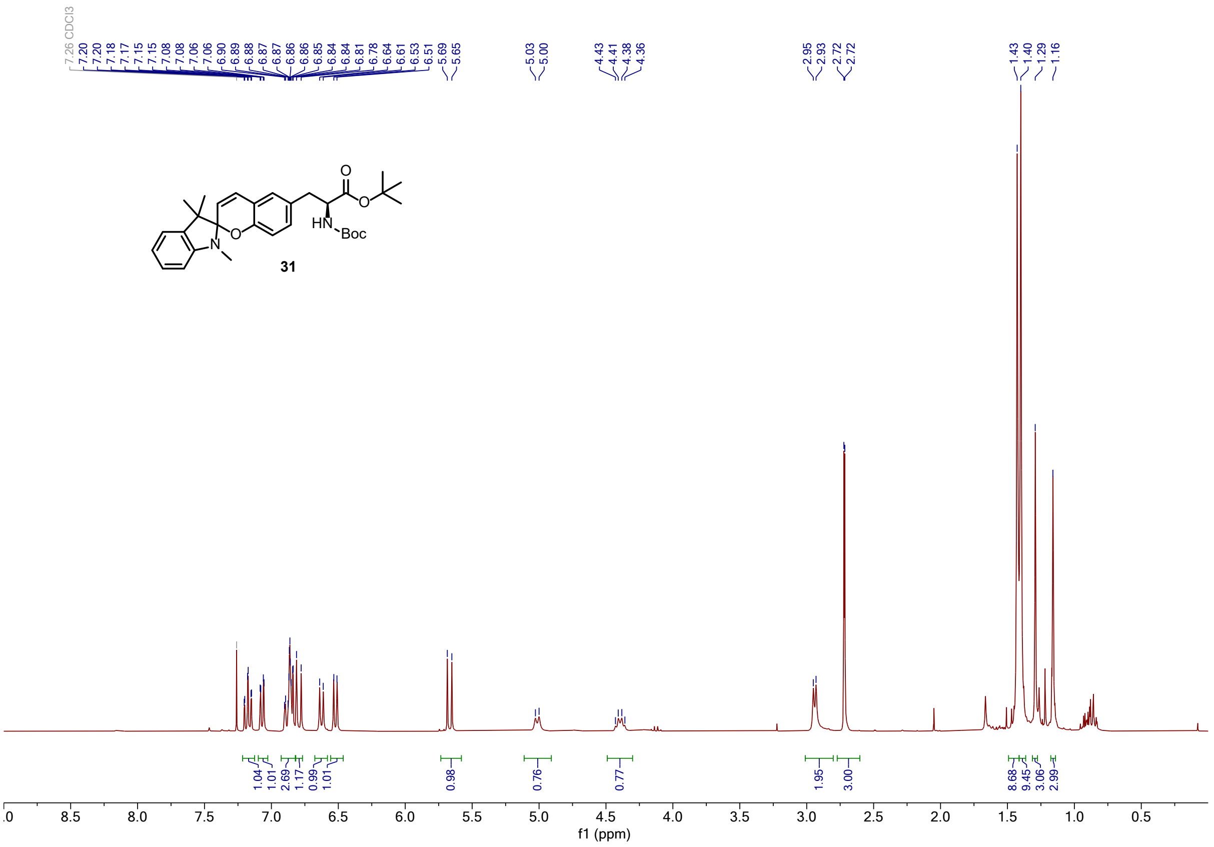
7.26 CDCl3
31
f1 (ppm)

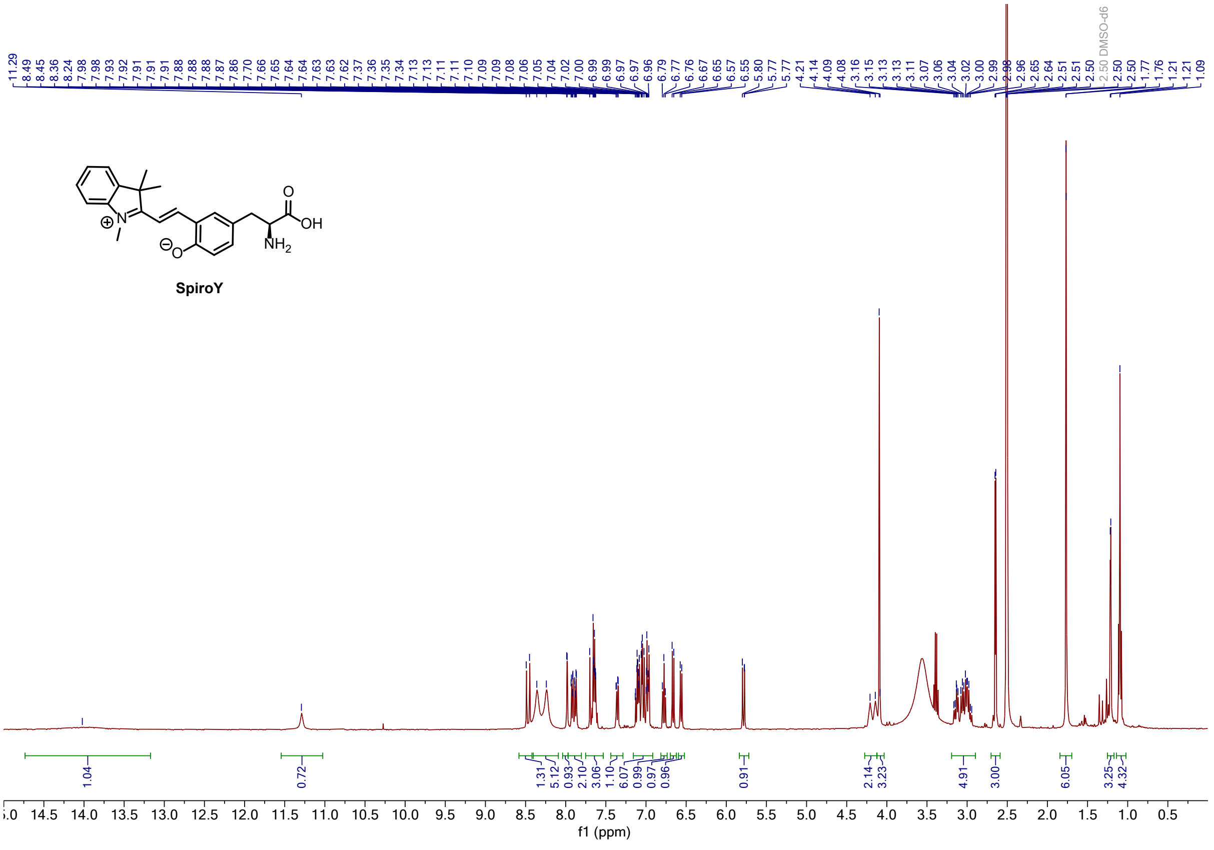
2.50 DMSO-d6
SpiroY
f1 (ppm)

2.1.4 13C-NMR Spectra

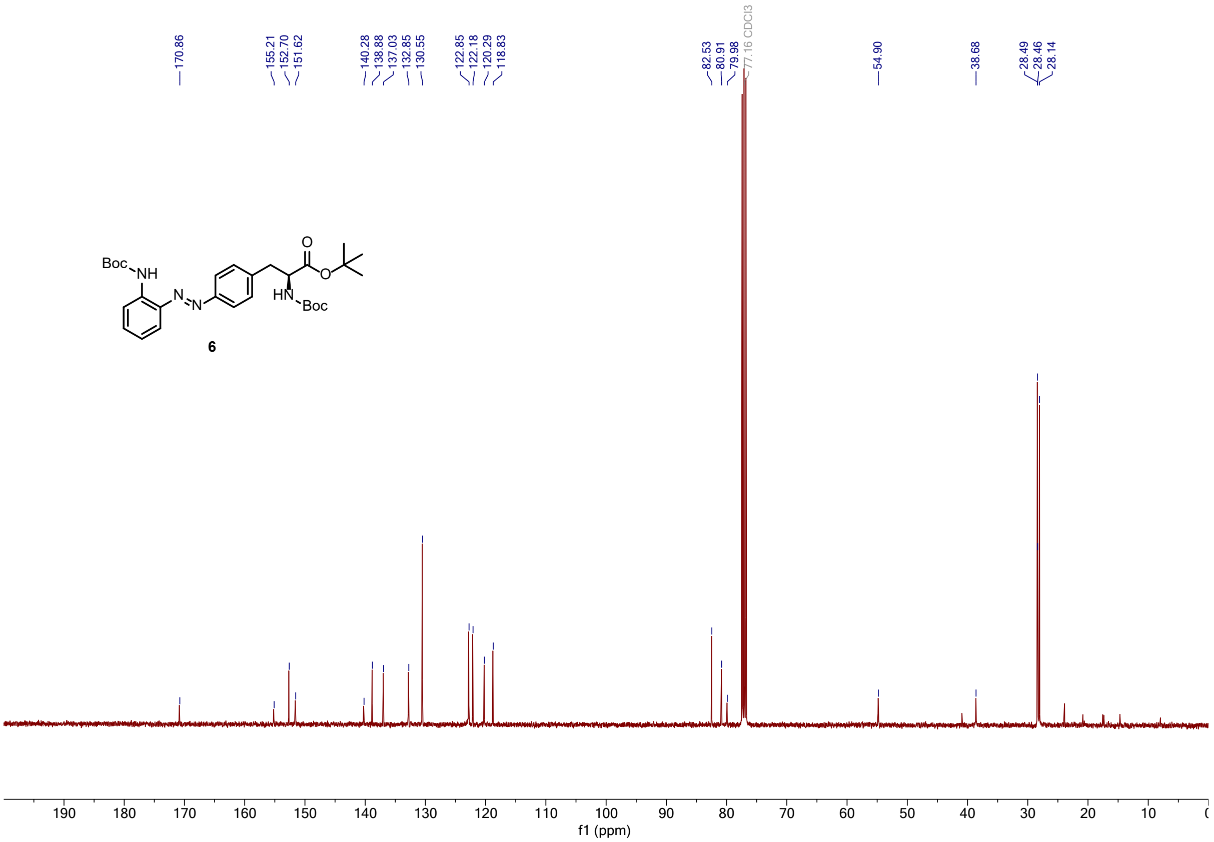

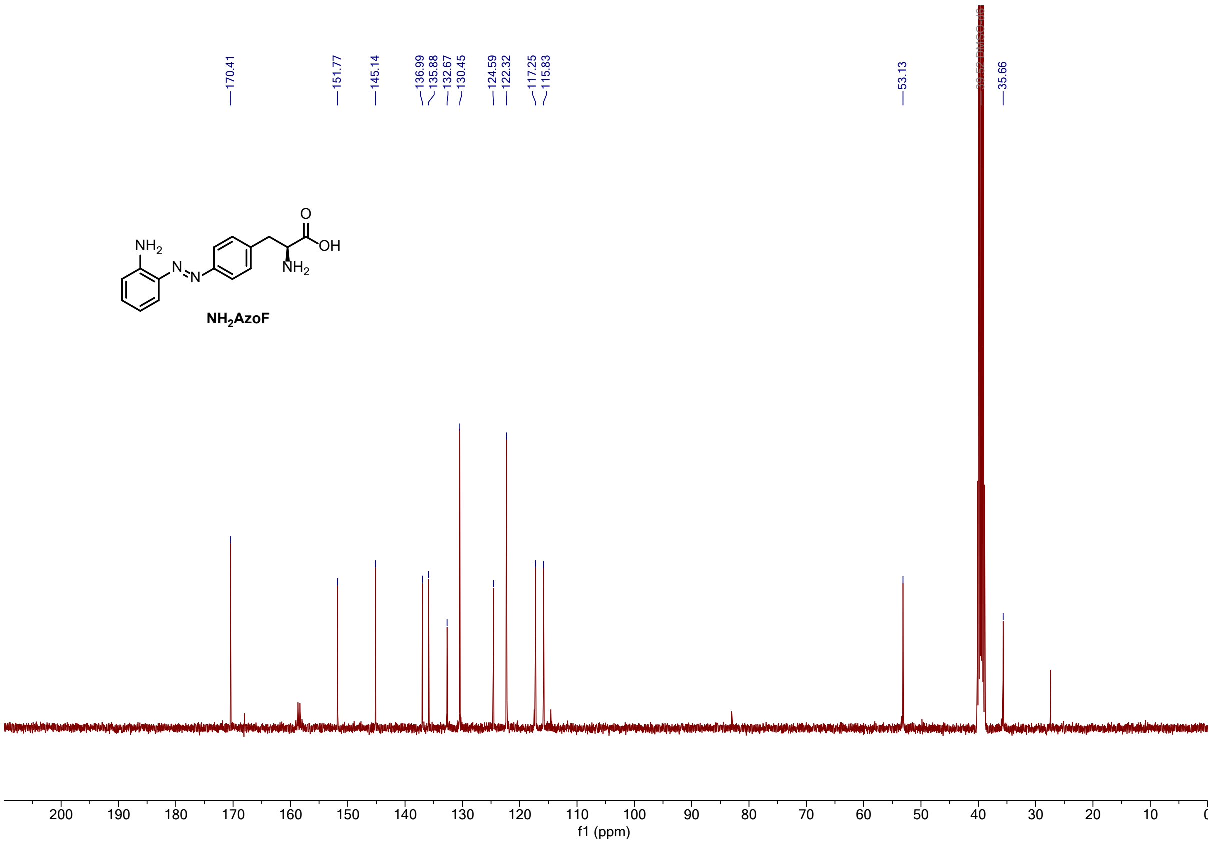

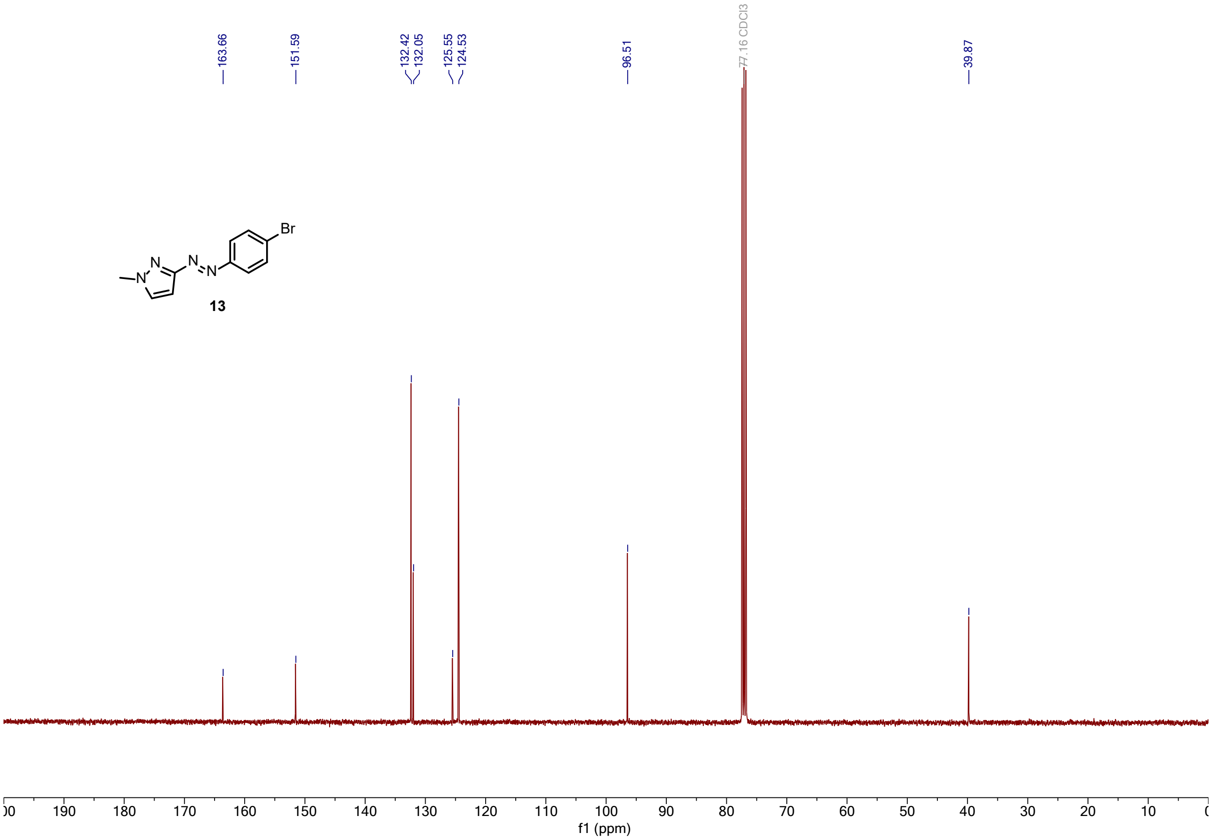
163.66
151.59
132.42
132.05
125.55
124.53
96.51
77.16 CDCl3
39.87
Br
N
N
N
N
13
f1 (ppm)

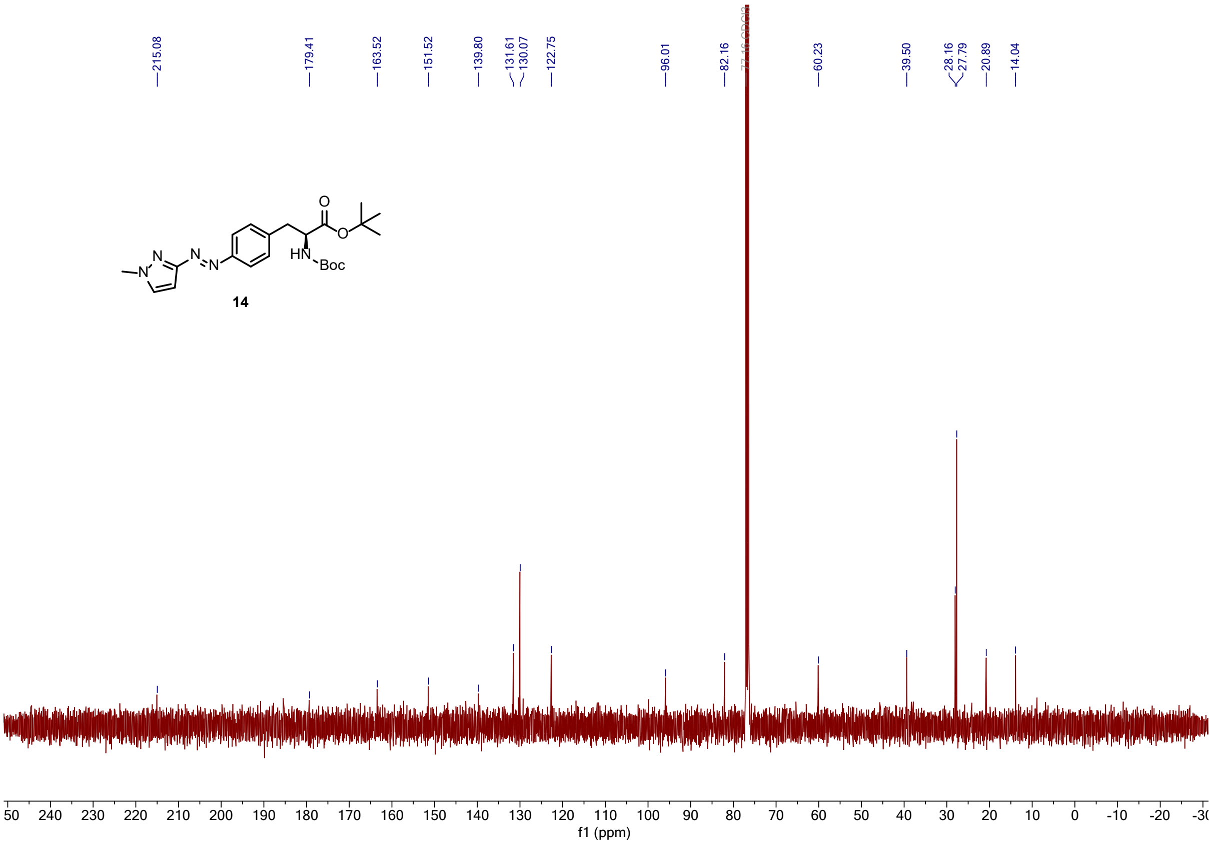
215.08
179.41
163.52
151.52
139.80
131.61
130.07
122.75
96.01
82.16
60.23
39.50
28.16
27.79
20.89
14.04
O
O
HN
Boc
N
N
N
N
14
f1 (ppm)

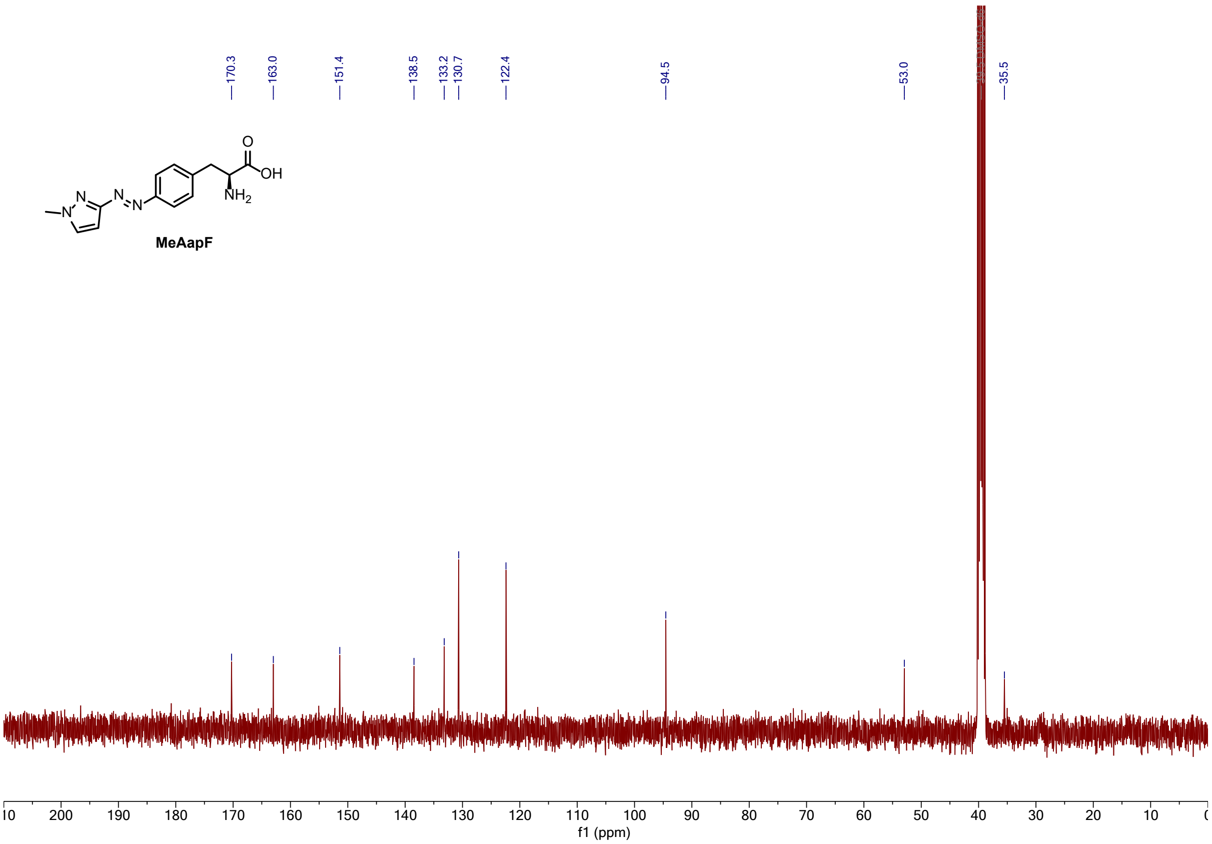
170.3
163.0
151.4
138.5
133.2
130.7
122.4
94.5
53.0
35.5
O
OH
N
N
N
N
NH2
MeAapF
f1 (ppm)

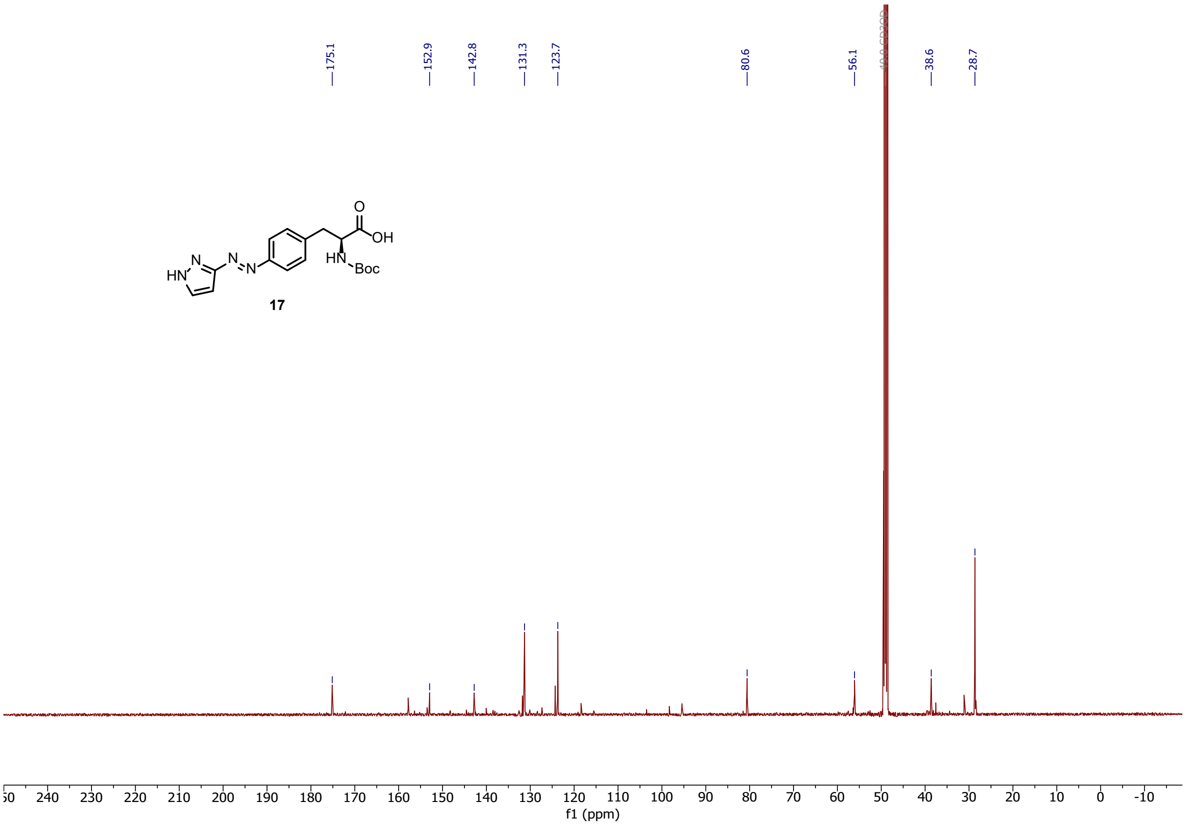
175.1
152.9
142.8
131.3
123.7
80.6
56.1
38.6
28.7
O
OH
HN
N
N
N
HN
Boc
17
f1 (ppm)

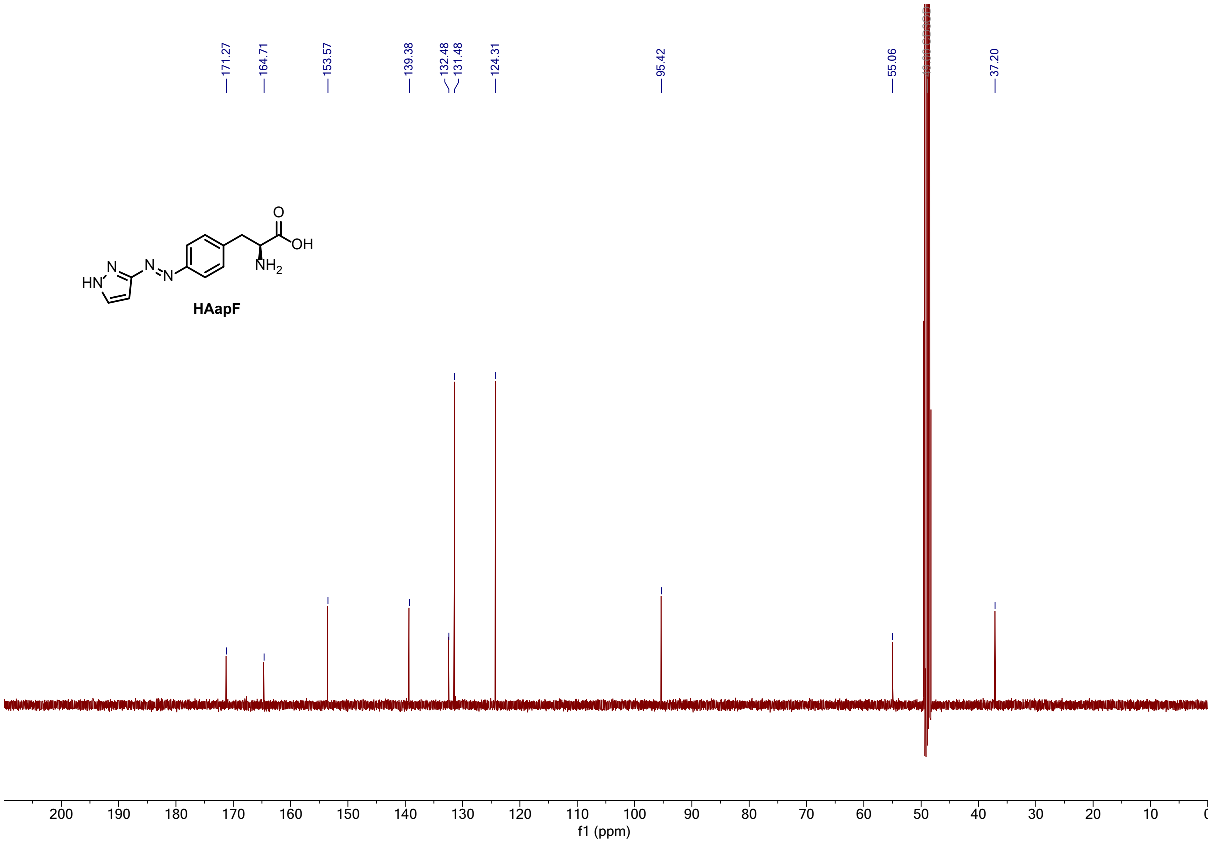
171.27
164.71
153.57
139.38
132.48
131.48
124.31
95.42
55.06
37.20
HAapF
f1 (ppm)

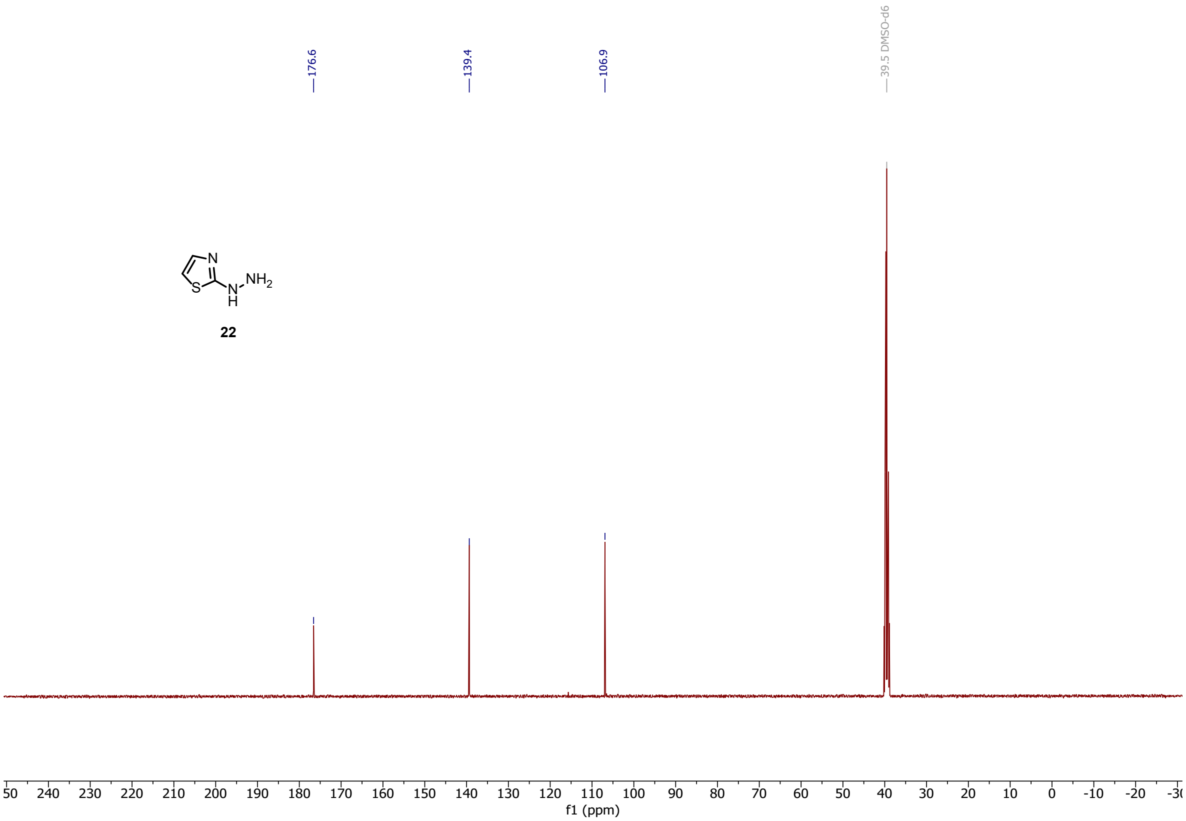
176.6
139.4
106.9
39.5 DMSO-d6
22
f1 (ppm)

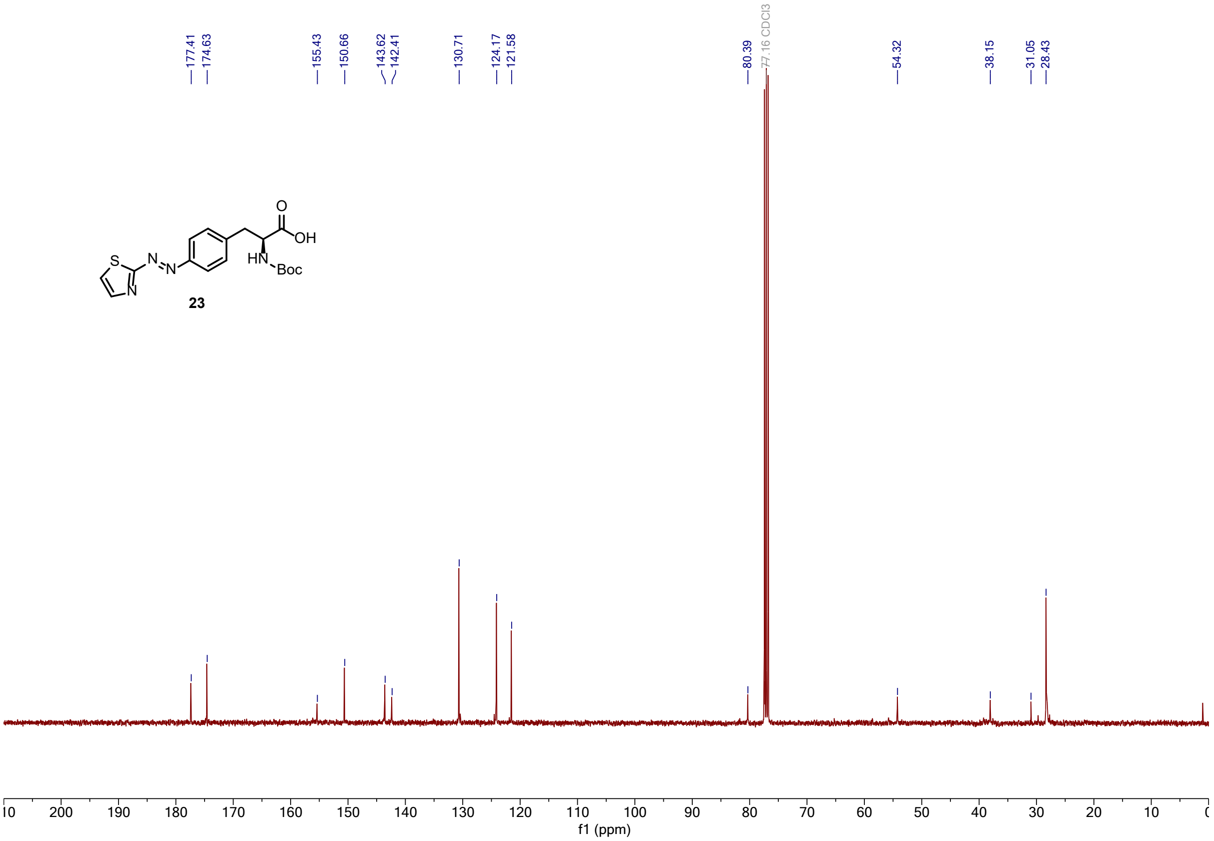

177.41
174.63
155.43
150.66
143.62
142.41
130.71
124.17
121.58
80.39
77.16 CDCl3
54.32
38.15
31.05
28.43
f1 (ppm)

23

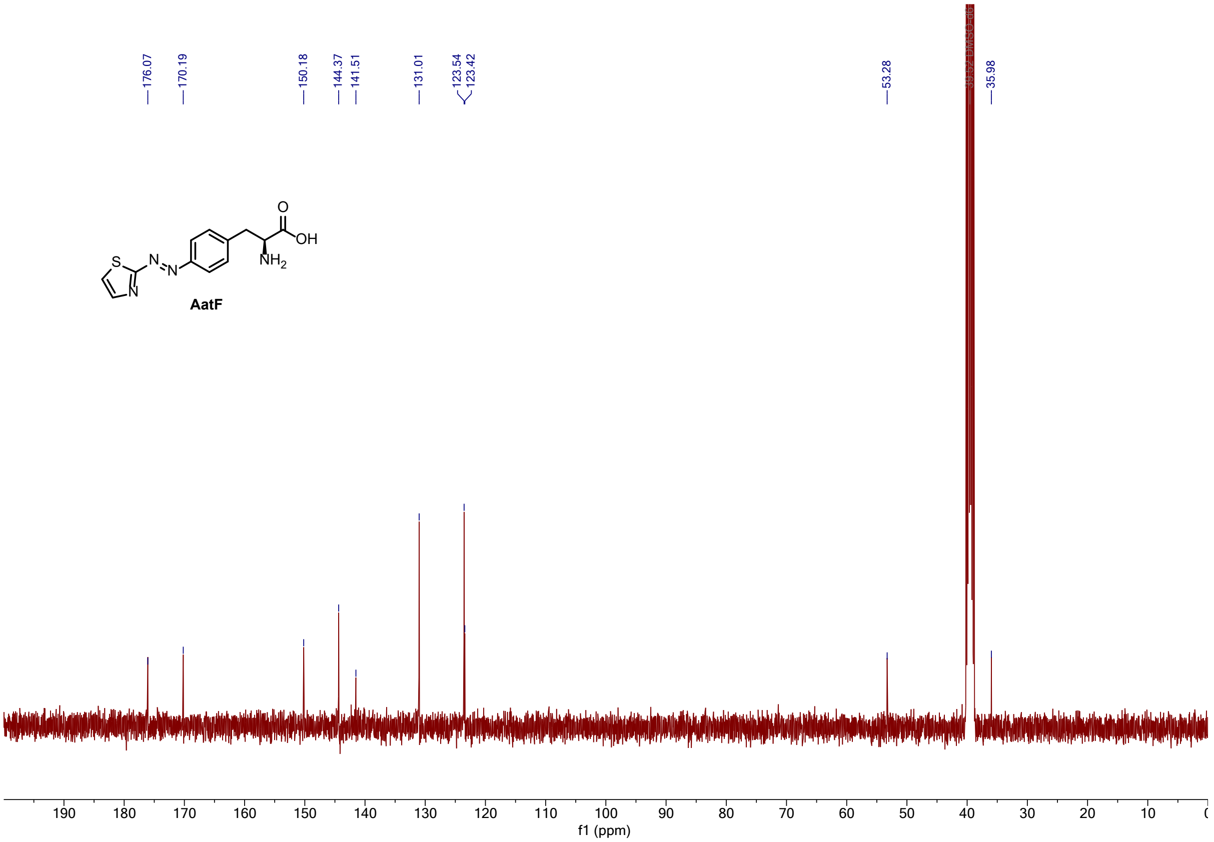

176.07
170.19
150.18
144.37
141.51
131.01
123.54
123.42
53.28
35.98
f1 (ppm)

AatF

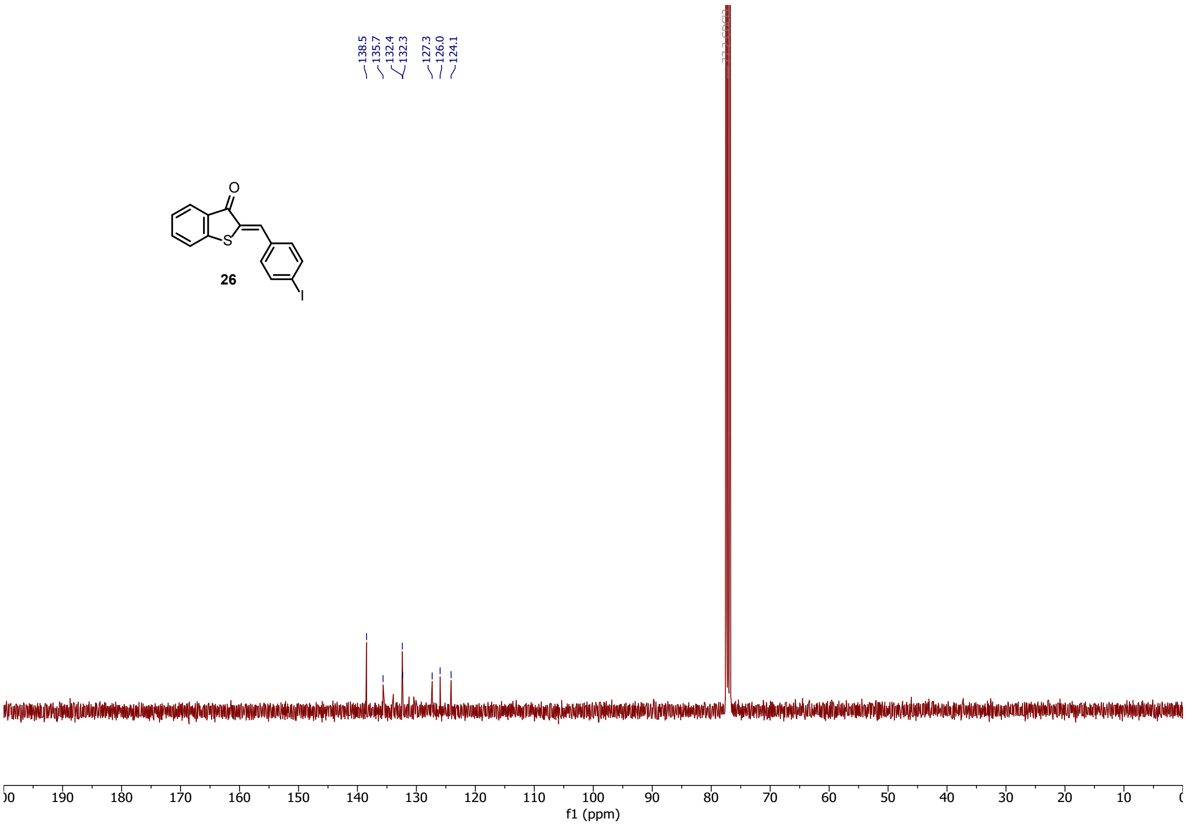
138.5
135.7
132.4
132.3
127.3
126.0
124.1
O
S
I
26
f1 (ppm)

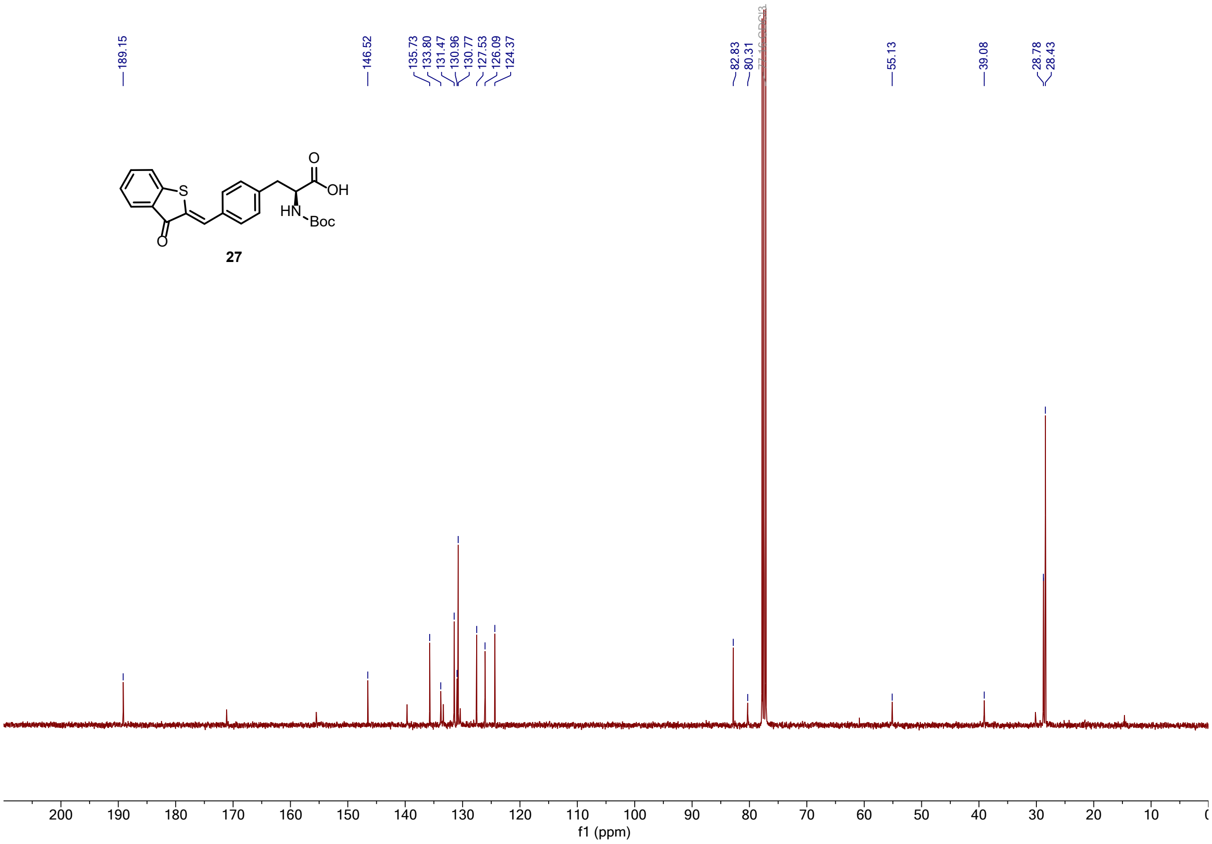
189.15
146.52
135.73
133.80
131.47
130.96
130.77
127.53
126.09
124.37
82.83
80.31
55.13
39.08
28.78
28.43
S
O
OH
HN
Boc
27
f1 (ppm)

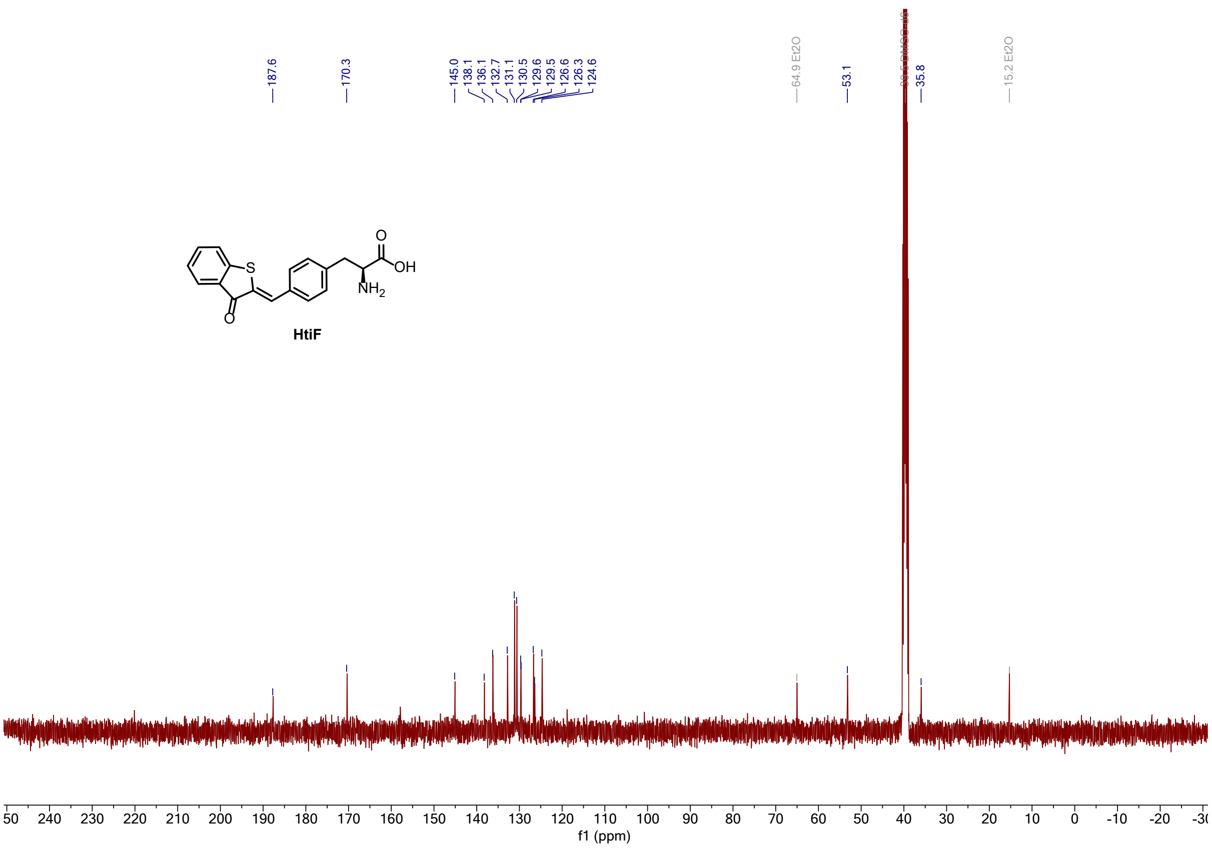
HtiF
f1 (ppm)

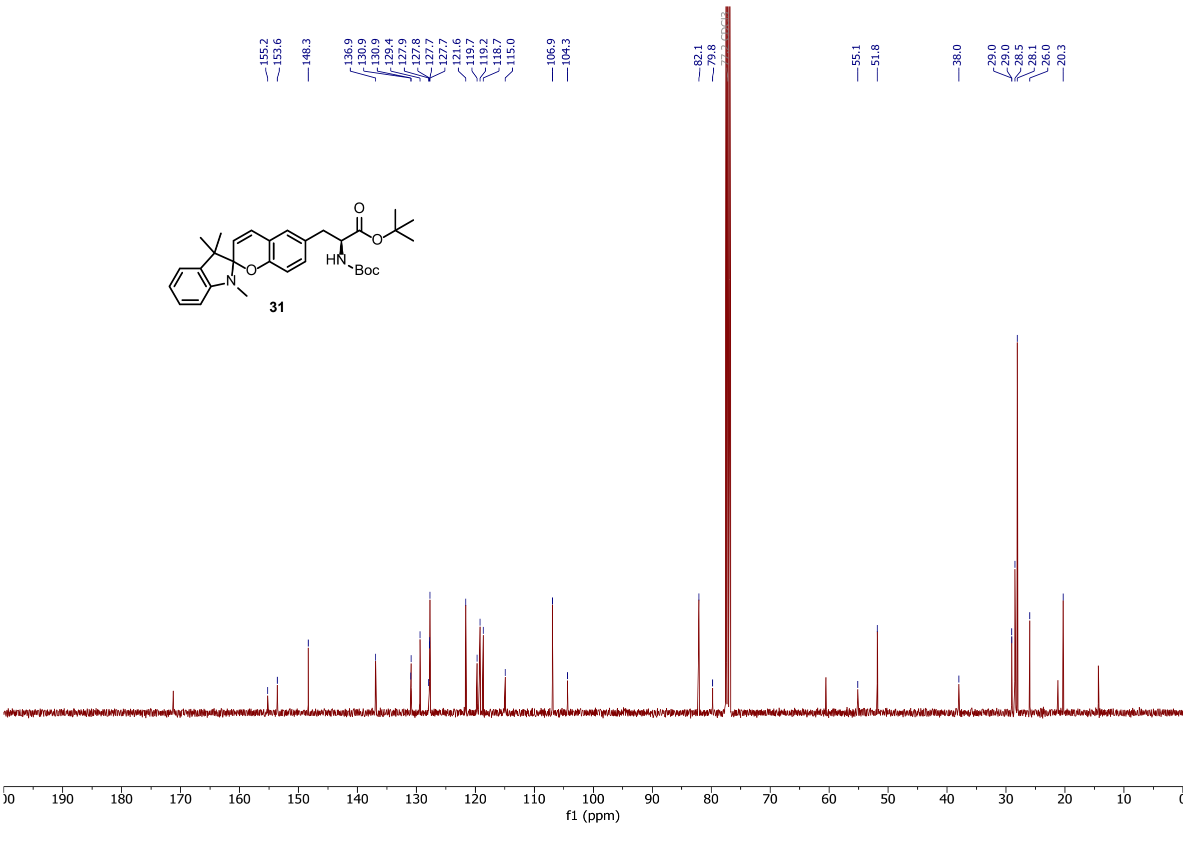
31
f1 (ppm)

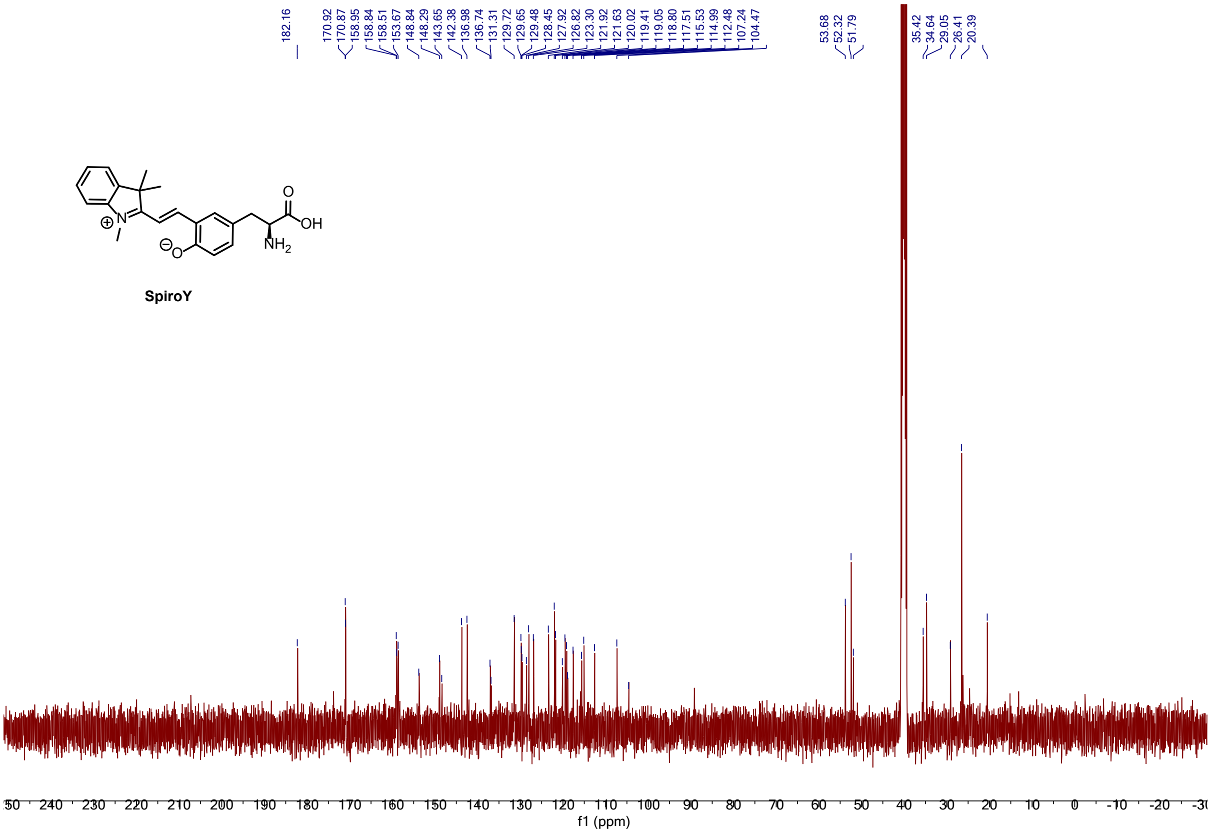
O
OH
NH2
N
O
SpiroY
182.16
170.92
170.87
158.95
158.84
158.51
153.67
148.84
148.29
143.65
142.38
136.98
136.74
131.31
129.72
129.65
129.48
128.45
127.92
126.82
123.30
121.92
121.63
120.02
119.41
119.05
118.80
117.51
115.53
114.99
112.48
107.24
104.47
53.68
52.32
51.79
35.42
34.64
29.05
26.41
20.39
f1 (ppm)

2.2 Irradiation Devices

Irradiation of psUAAs and HisF-psUAA variants was conducted using a programmable power supply (Korad, KA3005P, 30 V, 5 A). The applied settings include a voltage of 3.9 V and a maximum current of 0.5 mA for each LED (**Table S14**), unless otherwise stated. To realize simultaneous irradiation and UV/Vis measurement, the LEDs were installed perpendicular to the measurement beam directly in front of the cuvette.

Table S14. Specifications of LEDs used for irradiation.

LEDs	λ_{max} [nm]	I_{max} [mA][a]	P_{max} [mW][b]
Seoul Viosys CUD4AF1B	340	500	12
Nichia NCSU033B	365	700	240
Nichia NCSU034B	385	700	180
Luxeon SZ-01-S2 LHUV-0395-A065	400	1000	155
ILH-XC01-S410-SC211-WIR200	420	800	290
Oslon SSL 80 LDCQ7P-1U3U	455	1000	330
Oslon SSL 80 LBCP7P-GYHY	470	1000	260
Winger WEPCN3-S1	505	700	155
Oslon SSL 80 LTCP7P-KXKZ	528	1000	120
Luxeon Rebel ES Lime	567	1000	130

[a]The maximum forward current was taken from the manufacturer specifications. [b]Intensity was determined with a Field Max_{II}-TO high sensitivity power sensor (Coherent, #1098579, Range 0–10 W) directly in front of the LED.

2.3 UV/Vis absorbance spectra

UV/Vis absorbance spectra of the psUAAs and the HisF-psUAA variants were recorded in a Varian Cary 4000 Spectrometer in the range of 250–700 nm in a 1 cm quartz cuvette at 25 °C. Measurements of each isolated psUAA were performed in 50 mM Tris/HCl pH 7.5 (40 µM **AzoF**, **NH_2AzoF**, **MeAapF**, **HAapF**, and **AatF**, as well as 20 µM **HtiF**, and 40 µM **SpiroY**) or in DMSO (40 µM **AzoF**, **NH_2AzoF**, **MeAapF**, **HAapF**, and **AatF**, as well as 40 µM **HtiF**, and 40 µM **SpiroY**) and UV/Vis spectra of the HisF-psUAA variants were acquired in 50 mM Tris/HCl pH 7.5, 100 mM NaCl (28 µM K13AzoF, 13 µM K13NH_2AzoF, 9 µM K13MeAapF, 30 µM K13HAapF, 21 µM K13AatF, 9 µM K13HtiF). Samples were measured in the thermal equilibrium (TEQ), after establishment of a certain PSS (PSS^{λ}) or after back-isomerization to thermal equilibrium post irradiation (TEQ^{post}). Irradiation was maintained during the measurement to avoid back-isomerization of isomers with low thermodynamic stability. All spectra were baseline corrected at 700 nm.

2.4 Wavelength screen and determination of isomerization rates

To identify the most effective wavelengths in terms of a *Z*-enriched or *E*-enriched PSS in 50 mM Tris/HCl pH 7.5 and DMSO as well as of the corresponding HisF-psUAA variants in 50 mM Tris/HCl pH 7.5, 100 mM NaCl, a wavelength screen was performed. At first, UV/Vis spectra of the thermally equilibrated state ($TEQ^{a.s.}$ or $TEQ^{a.i.}$) were recorded in the dark. Starting from this state, the absorbance at the maximum of either the π→π* or the n→π* transition was followed spectrophotometrically. At a certain timepoint, the sample was irradiated at a specific wavelength *λ* and the absorbance was followed until a plateau was reached, indicating the complete establishment of PSS^{λ}. The measurement was stopped, and a spectrum was recorded while irradiation was maintained to avoid thermal back-isomerization. Isomerization rates for *E*→*Z* isomerization were recorded starting from TEQ (isolated psUAAs) or $TEQ^{a.i.}$ (HisF-psUAA variants), while isomerization rates for *Z*→*E* isomerization were acquired starting from the PSS^{λ} with the highest amount of *Z* isomer. Comparison of all spectra revealed the most effective wavelengths in terms of a high PSS.

For **HtiF**, the procedure to establish the best wavelengths is identical, however, the *Z* isomer is the thermodynamically favored one and consequently, the terminology *E*→*Z* and *Z*→*E* isomerization has to be reversed in the upper paragraph.

For **SpiroY**, the absorbance signal changed right after dilution of the stock solution in DMSO and thus, rates for *O*→*C* isomerization towards the newly established $TEQ^{post\ 1}$ were acquired in the dark instead of *O*→*C* photoisomerization.

Additionally, the rates of isomerization *k* were determined from the time-resolved absorbance values at the π→π* or the n→π* transition by fitting the data with a mono-exponential function (Equation 1).

$$\boldsymbol{y = y_0 + A\,(1 - e^{-x/k})}$$

Equation 1

The half-life of isomerization $t_{1/2}$ was then derived from the isomerization rate *k* using Equation 2.

$$\boldsymbol{t_{1/2} = ln(2) \times k}$$

Equation 2

For isomerization of **HtiF** with 420 nm, a double-exponential fit was required using Equation 3 and the half-life of isomerization $t_{1/2}$ was derived using Equation 4.

$$y = (y_0 D - P_D) \times e^{-k_D \times x} + P_D + y_0 A + (P_A - y_0 A) \times e^{-k_A \times x}$$

Equation 3

$$t_{1/2} = \frac{ln(2)}{k}$$

Equation 4

2.5 Cycle performance

Cycle performance experiments of the isolated psUAAs and HisF-psUAA variants were performed to investigate their susceptibility to photobleaching. To this end, alternating isomerization between *Z*-enriched and *E*-enriched PSS^{λ} (*O*-enriched and *C*-enriched for **SpiroY**) was induced either by irradiation or thermally for ten cycles, where switching to *E* (*O*) and subsequently to *Z* (*C*) or inversely represents one cycle. Isomerization was induced for a time period of at least ten times the $t_{1/2}$ of isomerization that was determined for each wavelength or thermally to ensure complete establishment of the respective PSS^{λ}. UV/Vis spectra of each PSS^{λ} were measured and the absorbance at the π→π* or the n→π* transition was plotted against the number of cycles. For employed psUAA and HisF-psUAA concentrations see section 2.3.

2.6 Determination of isomer ratios via high pressure liquid chromatography

To accurately determine the isomer composition of the isolated psUAAs in the established PSS, high pressure liquid chromatography (HPLC) was performed. Therefore, a reverse-phase Eclipse XDB-C18 column (Agilent, 80 Å, 5 µm, 4.6 x 150 mm) connected to an Agilent 1200 series HPLC system was employed. Mobile phase A consisted of 0.1% trifluoroacetic acid (TFA) in H_2O, mobile phase B of 0.1% TFA in acetonitrile. The isomers were separated at 15 °C (to prolong thermal relaxation for **NH_2AzoF** and **HtiF** when diluted in 50 mM Tris/HCl pH 7.5) or 25 °C (when diluted in DMSO) with a flow rate of 1 mL/min in the following manner: isocratic elution at 5% B from 0–3 minutes, linear gradient from 5–98% B from 4–23 minutes, and isocratic elution at 98% B from 24–26 minutes. To enable a quantitative comparison of the distinct isomers, their elution was followed at an isosbestic point (**Table S4**) describing a wavelength at which the absorption of both isomers is equal. Measurements of isolated psUAAs were performed in the thermal

equilibrium as synthesized (TEQ), after irradiation with the selected wavelengths (PSS^{λ}) or after back-isomerization to thermal equilibrium post irradiation (TEQ^{post}). Isomerization was induced for a time period of at least ten times the $t_{1/2}$ of isomerization directly before the samples were subjected to HPLC. Isomer ratios of the psUAA were determined by integration of the respective isomer peaks. For HPLC measurements in both Tris buffer and DMSO, 400 µM of **AzoF**, **NH_2AzoF**, **MeAapF**, **HAapF**, **AatF**, as well as 400 µM **HtiF**, and 400 µM **SpiroY** were employed.

2.7 Estimation of isomer ratios from UV/Vis spectra

The isomer ratios of the isolated psUAAs that are based on a diazo scaffold were also estimated as described in ref[17]. The following procedure describes the PSS estimation for a psUAA where the more thermodynamically stable isomer is the *E* isomer. The baseline-corrected spectra of the psUAA in the thermal equilibrium, as well as in the respective PSS were plotted in Origin 2022 (OriginLab). Initially, the fraction of *E* isomer present in the thermal equilibrium sample (TEQ) was assumed to be 100 %. Then, the spectrum of 100 % *Z* isomer was simulated based on the PSS with the highest concentration of *Z* isomer by estimating the residual fraction of *E* in this PSS using Equation 5.

$$A_Z = \frac{A_{PSS} - A_E \times f}{1 - f}$$

Equation 5

Where A_Z is the absorbance of the 100 % *Z* isomer, A_E is the absorbance of the 100% *E* isomer, A_{PSS} is the absorbance of the photostationary state, and *f* is the fraction of *E* isomer present in the PSS. The fraction *f* was manually varied to reach a point for A_Z at which the characteristic π-π* absorption signal approximates zero under the premise that the absorbance remained positive at all wavelengths (100 % *Z* spectrum). The other PSS ratios were assigned by interpolating between the *E* and *Z* isomer spectra using Equation 6.

$$f_{PSS} = \frac{A_{PSS} - A_Z}{A_E - A_Z}$$

Equation 6

Where f_{PSS} is the fraction of *E* isomer present in the PSS and A_Z, A_E, and A_{PSS} are the absorbances at the π-π* *E* isomer absorbance maximum.

Assessment of the isomer distribution based on UV/Vis spectra was not possible for **HtiF**. In the case of **SpiroY**, it was assumed that 100% of the closed form would not exhibit any characteristic absorbance at the determined λ_{max} since the decisive conjugated π-system is disrupted in the closed form. Isomer ratios were then estimated using proportional calculations based on the "rule of three". Estimation of the isomer ratios of the HisF-psUAA variants with a psUAA that is based on a diazo switching scaffold was equally feasible, because the protein signal at 280 nm does not interfere with the absorbance at the π→π* transition (>320 nm) of the respective psUAA.

2.8 Thermal stability

Thermal stability of the psUAAs was measured in 50 mM Tris/HCl pH 7.5 at 25 °C. To this end, the PSS with the highest amount of thermodynamically less stable isomer, which is PSS^{365} for **AzoF**, PSS^{385} for **NH_2AzoF**, PSS^{365} for **MeAapF**, PSS^{340} for **HAapF**, PSS^{400} for **AatF**, PSS^{455} for **HtiF**, and PSS^{455} for **SpiroY** was induced. In case of **HAapF**, a 340 nm LED was used to establish the PSS^{340} which exhibits an even higher amount of *Z* isomer than the PSS^{365} (**Figure S9**). For UAAs with a relatively fast thermal isomerization (**NH_2AzoF**, **AatF**, **HtiF**, and **SpiroY**), the regeneration of the absorbance at the π→π* or the n→π* transition after irradiation had stopped was followed over time using UV/Vis spectroscopy. For psUAAs that exhibit a longer thermal isomerization (**AzoF**, **MeAapF**, **HAapF**), HPLC measurements were conducted at certain timepoints after irradiation had stopped. The isomer ratios were deduced from the respective peak integrals and the fraction of *Z* isomer was plotted against time. Mono-exponential fitting obtained the rate constants of thermal back-isomerization *k* (Equation 1). The thermal half-life $t_{1/2}(\Delta T)$ was then derived using the isomerization rate *k* and Equation 2.

In the case of **SpiroY**, $t_{1/2}$ (ΔT) was additionally measured starting from the TEQ after dilution from a 40 mM stock in DMSO to 40 µM in either DMSO or 50 mM Tris/HCl pH 7.5 (TEQ^{post}). The thermal half-life in buffer was sufficiently fast to be conducted via absorbance spectroscopy, while the measurement in DMSO was performed using HPLC.

2.9 Computational Design of aaRS

The computational designs were created by Rosetta:MSF:NN[42] in combination with Rosetta FastDesign.[43] The design was limited to native rotamers at each position aside from positions flagged as mutable, where native amino acids were allowed. To find an aaRS suitable for **HAapF** incorporation, the following positions were allowed to mutate: 32, 65, 108, 109, 158, and 162. To improve the performance with **AzoF**, the following positions were allowed to mutate: 67, 155, 164, 167, 176, 180, and 188. In this case, the native amino acids on positions 164, 176, and 180 scored best and therefore were not mutated to another amino acid. An additional parameter file (Aap.params), as well as exemplary flags- (FastDesign.flags) and resfile (FastDesgin.resfile) files of the Rosetta design were provided as supplementary data for **HAapF**.

2.10 Subcloning and site-directed mutagenesis

The plasmid pET28a_tmHisF_K13TAG was taken from previous work.[44] The plasmid pEVOL_AzoF-RS was provided by Peter Schultz (Scripps Research Institute, La Jolla, USA). The genes of sfGFP_wt, sfGFP_Y151TAG, AzoF-RS_{copt}, ONBY-RS,[28] Azo-RS 1, and AapF-RS, were codon-optimized for expression in *E. coli* and synthesized by ThermoFisher Scientific (GeneArt Strings and DNA fragments).

To introduce site-directed substitutions, deletions and insertions, or to prepare plasmids and gene fragments for Golden Gate Cloning[45] by equipping them with suitable restriction sites, PCR-based site-directed mutagenesis was employed based on a protocol from Finnzymes (ThermoFisher Scientific). Thereby, all employed primers (**Table S13**) were designed to initiate elongation in opposite directions while their 5' ends are directly adjacent to each other, with one or both primers containing the desired mutations or insertions. To introduce deletions, the primers were designed not to be adjacent to each other but flanking the region that should be deleted. The resulting linear amplicon was then re-circularized in a coupled phosphorylation-ligation reaction using T4 Polynucleotide Kinase and T4 DNA Ligase (ThermoFisher Scientific) according to the manufacturer's instructions. In all cases, the presence of mutations, insertions or deletions as well as the integrity of the plasmid was confirmed by DNA sequencing (MicroSynth AG).

Synthetic genes or amplified sequences were subcloned into plasmids using Golden Gate Cloning.[45] Golden Gate Cloning is a very efficient molecular cloning technique which

allows for the simultaneous assembly of multiple DNA fragments using type IIS restriction enzymes such as BsaI and BbsI. Type IIS restriction enzymes recognize a certain palindromic site and cut the DNA outside of this recognitions site, thereby creating a four nucleotide overhang independent of the cutting site sequence. Hence, once the product is ligated, the recognition sites are no longer part of it and consequently, it cannot be cleaved again. Fragments or plasmids that contain suitable recognition sites were subjected to a one-pot restriction-ligation reaction as described in Semmelmann *et al.*.[46] To verify the integrity of all plasmids generated by Golden Gate Cloning, DNA sequencing was performed (MicroSynth AG).

In principle, the pEVOL plasmid contains two functional gene expression cassettes, one under the control of an arabinose-inducible promoter (araBAD) and one that is constitutively expressed (glnS). Using suitable primers, the two plasmids were amplified in a way that they contain either the arabinose-inducible (pBAD) or the constitutive expressing (pGLNS) cassette while simultaneously lacking the respective other one. After amplification from the pEVOL_AzoF-RS plasmid,[1] pBAD and pGLNS were equipped with suitable BsaI or BbsI recognition sites for subsequent subcloning via PCR.

All ordered synthetic genes of aaRS variants were directly subcloned into the pGLNS plasmid using Golden Gate cloning. Sequences of aaRS that have already been available in the lab (AzoF-RS$_{copt}$, pEVOL_ONBY-RS$_{i}$[47] and pEVOL_AzF-RS[48] previously obtained from Scripps Research Institute, La Jolla, USA) were equipped with suitable restriction sites by amplification from their respective plasmids and subcloned into pGLNS. Further point-mutations were introduced in the aaRS sequences using site-directed mutagenesis. For the incorporation of **HAapF**, pEVOL_Azo-RS 4 was generated by exchanging the two AzoF-RS genes on pEVOL_AzoF-RS[1] with Azo-RS 4 sequences in two steps of BsaI and BbsI subcloning.

The synthetic gene of sfGFP_Y151TAG was subcloned into pBAD under the control of the arabinose inducible expression cassette. Since pBAD and pGLNS carry both a chloramphenicol resistance, the whole expression module including promoter and terminator was subcloned onto a pET24a plasmid harboring a kanamycin resistance. To this end, the T7 expression cassette was removed from the pET24a plasmid and suitable restriction sites were introduced. The entire araBAD_sfGFP_Y151TAG_rrnB cassette

was amplified from the pBAD plasmid with suitable restriction sites and subcloned into the prepared pET24a plasmid resulting in pETBAD_sfGFP_Y151TAG. Finally, the stop codon was reverted to the wild type tyrosine via site-directed mutagenesis.

2.11 Synthetase screening

Chemically competent BL21 gold DE3 cells that already contained the pETBAD_sfGFP_Y151TAG plasmid were transformed with the respective pGLNS_aaRS plasmids. As negative and positive control, pGLNS empty vector was co-transformed with pETBAD_sfGFP_Y151TAG (–) and pETBAD_sfGFP_wt (+), respectively. The cell suspension was plated on $LB_{Cm+Kana}$ agar plates containing 30 µg/ml chloramphenicol and 75 µg/ml kanamycin and incubated at 37 °C overnight. To obtain biological triplicates, three colonies of each sample were used to inoculate 1.8 mL $LB_{Cm+Kana}$ (DWP) and cells were grown to saturation at 37 °C (>20 h). The next day, 100 µL of the pre-culture were used to inoculate 1.7 mL $2xYT_{Cm+Kana}$ (DWP). Cells were grown at 37 °C while shaking at 600 rpm and as soon as an OD_{600} of >0.7 was reached, the cell suspension was transferred to a 96-well microtiter plate (MTP) with conical bottom. Gene expression was induced by addition of 0.1% L-arabinose and 0.4 mM psUAA and cultures were further incubated at 30 °C overnight. The cells were harvested by centrifugation (4000 rpm, 45 min, 20 °C) and subsequently resuspended in 150 µL 50 mM Tris/HCl pH 7.5 to reduce background fluorescence caused by the medium before 100 µL of the cell suspension were transferred to a black MTP (**Figure S14**). The sfGFP fluorescence was measured with a TECAN reader (λ_{ex} = 488 nm, λ_{em} = 530 nm, Z-position = 19912 µM, number of flashes = 16). To allow for a comparison of the expression levels between the different MTPs, the amplification was set to the same value for every sfGFP measurement.

To investigate whether the effect of a second aaRS copy on incorporation efficiency, chemically competent BL21 gold DE3 cells that already contained the pETBAD_sfGFP_Y151TAG plasmid were transformed with the pEVOL_aaRS-g plasmid and the experiment was conducted as described above.

2.12 Computational methods

System preparation. The preparation of enzyme tyrosyl-tRNA synthetase variants from *Methanocaldococcus jannaschii* (MjTyr-RS) was performed using AlphaFold2 (v2.3.2)[49] (AF2) in monomer mode. The AF2 result of model 1 was selected for aminoacyl-tRNA

synthetase (aaRS)-p and aaRS-q, whereas model 2 was used for aaRS-g to avoid the blockage of R163 from the active site (**Figure S16**). These predicted structures were then used to create the cluster model with the photoswitchable unnatural amino acids (psUAAs) incorporated into the active site by superposing the psUAA onto the tyrosine residue of the 1J1U PDB x-ray structure. The final cluster model for each psUAA and aaRS pair incorporates all residues and water molecules located within 6 Å of the psUAA (**Table S15**).

Cluster model optimization. The cluster models were optimized using the Atomic Simulation Environment (ASE)[50] Python library with the LBFGS optimizer and the AIMNET2 machine learning interatomic potential.[51] A soft harmonic constraint was applied to the peripheral anchoring carbon atoms, specifically at those where the C–C bond was cleaved to define the cluster region.[52]

Table S15. Cluster model composition for the selected psUAA and *Mj*Tyr-RS pairs.

psUAAs	*Mj*Tyr-RS	cluster model composition
**AzoF**	aaRS-g	S30, A31, G32, I33, G34, F35, E36, P37, Q48, M52, I62, I63, I64, V65, L66, A67, D68, L69, H70, A71, Y72, N74, V103, Y104, G105, F108, Q109, M134, L136, I137, A138, R139, Y151, M154, Q155, V156, N157, G158, Y159, H160, Y161, R162, G163, V164, D165, V166, A167, V168, G169, G170, E172, Q173, I176, H177, V188, **AzoF**, WAT0, WAT1, WAT2, WAT3, WAT4, WAT5, WAT6, WAT7, WAT8, WAT9, WAT10, WAT11, WAT12, WAT13, WAT14, WAT15, WAT16, WAT17, WAT18, WAT19, WAT20, WAT21, WAT22, WAT23, WAT24, WAT25, WAT26, WAT27, WAT28, WAT29, WAT30, WAT31, WAT32, WAT33, WAT34, WAT35
**NH_2AzoF**	aaRS-g	S30, A31, G32, I33, G34, F35, E36, P37, Q48, M52, I62, I63, I64, V65, L66, A67, D68, L69, H70, A71, Y72, N74, V103, Y104, G105, F108, Q109, Y114, M134, L136, I137, A138, R139, Y151, M154, Q155, V156, N157, G158, Y159, H160, Y161, R162, G163, V164, D165, V166, A167, V168, G169, G170, E172, Q173, I176, H177, V188, **NH_2AzoF**, WAT0, WAT1, WAT2, WAT3, WAT4, WAT5, WAT6, WAT7, WAT8, WAT9, WAT10, WAT11, WAT12, WAT13, WAT14, WAT15, WAT16, WAT17, WAT18, WAT19, WAT20, WAT21, WAT22, WAT23, WAT24, WAT25, WAT26, WAT27, WAT28, WAT29, WAT30, WAT31, WAT32, WAT33, WAT34, WAT35, WAT36, WAT37, WAT38
**MeAapF**	aaRS-p	S30, A31, G32, I33, G34, F35, E36, P37, Q48, I62, I63, I64, E65, L66, A67, D68, L69, H70, A71, Y72, N74, V103, Y104, G105, M109, Y114, M134, L136, I137, A138, R139, Y151, M154, Q155, V156, N157, G158, I159, H160, Y161, H162, G163, V164, D165, V166, A167, V168, G169, G170, E172, Q173, I176, H177, V188, **MeAapF**, WAT0, WAT1, WAT2, WAT3, WAT4, WAT5, WAT6, WAT7, WAT8, WAT9, WAT10, WAT11, WAT12, WAT13, WAT14, WAT15, WAT16, WAT17, WAT18, WAT19, WAT20, WAT21, WAT22, WAT23, WAT24, WAT25, WAT26, WAT27, WAT28, WAT29, WAT30, WAT31, WAT32, WAT33, WAT34, WAT35

**HAapF**	aaRS-g	S30, A31, G32, I33, G34, F35, E36, P37, Q48, I63, I64, V65, L66, A67, D68, L69, H70, A71, Y72, N74, V103, Y104, G105, F108, Q109, M134, L136, I137, A138, R139, Y151, M154, Q155, V156, N157, G158, Y159, H160, Y161, R162, G163, V164, V166, A167, V168, G169, G170, E172, Q173, I176, H177, V188, **HAapF**, WAT0, WAT1, WAT2, WAT3, WAT4, WAT5, WAT6, WAT7, WAT8, WAT9, WAT10, WAT11, WAT12, WAT13, WAT14, WAT15, WAT16, WAT17, WAT18, WAT19, WAT20, WAT21, WAT22, WAT23, WAT24, WAT25, WAT26, WAT27, WAT28, WAT29, WAT30, WAT31, WAT32, WAT33, WAT34, WAT35
**AatF**	aaRS-q	S30, A31, G32, I33, G34, F35, E36, P37, Q48, I63, I64, E65, L66, A67, D68, L69, H70, A71, Y72, N74, V103, Y104, G105, E109, M134, L136, I137, A138, R139, Y151, M154, Q155, V156, N157, A158, I159, H160, Y161, H162, G163, V164, V166, A167, V168, G169, G170, E172, Q173, I176, H177, V188, K204, **AatF**, WAT0, WAT1, WAT2, WAT3, WAT4, WAT5, WAT6, WAT7, WAT8, WAT9, WAT10, WAT11, WAT12, WAT13, WAT14, WAT15, WAT16, WAT17, WAT18, WAT19, WAT20, WAT21, WAT22, WAT23, WAT24, WAT25, WAT26, WAT27, WAT28, WAT29, WAT30, WAT31, WAT32, WAT33, WAT34, WAT35, WAT36, WAT37, WAT38
**HtiF**	aaRS-g	S30, A31, G32, I33, G34, F35, E36, P37, H45, Q48, M52, I62, I63, I64, V65, L66, A67, D68, L69, H70, A71, Y72, N74, V103, Y104, G105, F108, Q109, M134, L136, I137, A138, R139, Y151, M154, Q155, V156, N157, G158, Y159, H160, Y161, R162, G163, V164, D165, V166, A167, V168, G169, G170, E172, Q173, I176, H177, V188, V189, C190, I191, **HtiF**, WAT0, WAT1, WAT2, WAT3, WAT4, WAT5, WAT6, WAT7, WAT8, WAT9, WAT10, WAT11, WAT12, WAT13, WAT14, WAT15, WAT16, WAT17, WAT18, WAT19, WAT20, WAT21, WAT22, WAT23, WAT24, WAT25, WAT26, WAT27, WAT28, WAT29, WAT30, WAT31, WAT32, WAT33, WAT34, WAT35

2.13 Mass spectrometry

The HisF-psUAA variants were applied to an SDS-PAGE (1 µg) and stained with Coomassie G250 (SimplyBlue SafeStain, Lifetech). Protein bands were cut out, washed with 50 mM NH_4HCO_3, 50 mM NH_4HCO_3/acetonitrile (3/1), 50 mM NH_4HCO_3/acetonitrile (1/1) and subsequently lyophilized. After reduction/alkylation treatment and additional washing steps, proteins were *in gel* digested with trypsin (Trypsin Gold, mass spectrometry grade, Promega) at 37°C overnight. The resulting peptides were sequentially extracted with 50 mM NH_4HCO_3 and 50 mM NH_4HCO_3 in 50% acetonitrile. After lyophilization, peptides were reconstituted in 20 µL 1% TFA and separated by reversed-phase chromatography. An UltiMate 3000 RSLCnano System (Thermo Fisher Scientific, Dreieich) equipped with a C18 Acclaim Pepmap100 preconcentration column (100 µm i.d. x 20mm, Thermo Fisher Scientific) and an Acclaim Pepmap100 C18 nano column (75 µm i.d. x 250 mm, Thermo Fisher Scientific) was operated at flow rate of 300 nL/min and a 60 min linear gradient of 4% to 40% acetonitrile in 0.1% formic acid. The LC was online-coupled to a maXis plus UHR-QTOF System (Bruker Daltonics) via a CaptiveSpray nanoflow electrospray source. Acquisition of MS/MS spectra after CID fragmentation was performed in data-dependent mode at a resolution of 60000. The precursor scan rate was 2 Hz processing a mass range between m/z 175 and m/z 2000. A dynamic method with a fixed cycle time of 3 s was applied via the Compass 1.7

acquisition and processing software (Bruker Daltonics). Prior to database searching with Protein Scape 3.1.3 (Bruker Daltonics) connected to Mascot 2.5.1 (Matrix Science), raw data were processed in Data Analysis 4.2 (Bruker Daltonics). A customized database comprising the *T. maritima* entries from UniProt as well as manually added sequences of the mutated HisF proteins and common contaminants was used for database search with the following parameters: enzyme specificity trypsin with 2 missed cleavages allowed, precursor tolerance 10 ppm, MS/MS tolerance 0.04 Da. As general variable modifications, deamidation (0.984 Da) of asparagine and glutamine, oxidation of methionine 15.995 Da, carbamidomethylation of cysteine 57.021 Da, or propionamide modification of cysteine 71.037 Da were set. Specific variable modifications for identification of unnatural amino acids were as follows: **AzoF**: 104.037 Da **NH_2AzoF**: 119.048 Da, **MeAapF**: 108.044 Da, **HAapF**: 94.028 Da, **AthzF**: 110.989 Da, or **HtiF**: 159.998 Da. Spectra of peptides containing unnatural amino acids were inspected manually.

2.14 Expression of HisF-psUAA variants in midi-scale

For expression of HisF-psUAA proteins, cells containing pET28a_HisF_K13TAG were transformed with the plasmids containing the appropriate aaRS variants for incorporation of **AzoF**, **NH_2AzoF**, **MeAapF**, **HAapF**, **AatF**, and **HtiF** (**Table S9**). A 50 mL $LB_{Cm+Kana}$ culture was inoculated and grown at 37 °C overnight. The next day, 50 mL $TB_{Cm+Kana}$ were inoculated to an OD_{600} of 0.1 and incubated at 37 °C until an OD_{600} >1.2 was reached. Gene expression was induced by addition of 0.4 mM psUAA and 0.5 mM IPTG (+0.1 % L-arabinose if the aaRS was encoded on the pEVOL plasmid) before the culture was incubated at 30 °C overnight. The cells were harvested by centrifugation (4000 rpm, 45 min, 20 °C) and subsequently resuspended in 5 mL 50 mM Tris/HCl pH 7.5, 100 mM NaCl, 10 mM imidazole. Cells were lysed by sonification (2 min, 30%, 2 s pulse, 2 pause, followed by a heat step at 65 °C and another centrifugation step to remove *E. coli* proteins. The lysate was subjected to immobilized metal ion affinity chromatography using HisSpinTrap™ columns (GE Healthcare) following to protocol provided by the manufacturer and elution was performed twice with 250 µL 50 mM Tris/HCl pH 7.5, 100 mM NaCl, 750 mM imidazole. The buffer of the eluates was exchanged using NAP-5 columns (GE Healthcare) according to the manufacturer's instructions. Concentration of the HisF-psUAA variants was determined with the Bradford Assay before their

photochemical characterization was performed as described in sections above. For long-term storage, the proteins were dropped into liquid nitrogen and frozen at –80 °C.

2.15 Heterologous expression of HisA

HisA from *T. maritima* was produced by heterologous gene expression in *E. coli* BL21 Gold (DE3) cells (Agilent Technologies) using pET21_HisA.[53] The crude extract was subjected to a heat step (15 min at 73 °C) and the recombinant protein was purified from the soluble fraction by nickel-affinity chromatography (HisTrap FF crude 5 ml; GE Healthcare). The column was equilibrated with 50 mM potassium phosphate, 300 mM NaCl, and 1 mM imidazole, pH 7.5 and bound protein as eluted by applying a linear gradient to 300 mM imidazole. Fractions containing pure protein were pooled and dialyzed against 50 mM potassium phosphate, pH 7.5. Based on SDS-PAGE analysis, the purity of all samples was at least 90%. The proteins were dripped into liquid nitrogen and stored at –80 °C.

2.16 CD spectroscopy

Circular dichroism (CD) spectra in the far-UV range of 195–250 nm were recorded in a Jasco J-815 spectrophotometer with five accumulations and a scan speed of 50 nm/min. Spectra of 14 µM HisF-wt, 18 µM K13AzoF, 12.4 µM K13NH_2AzoF, 13.5 µM MeAapF, 15 µM K13HAapF, 15 µM K13AatF, and 7.5 µM HtiF were measured in 50 mM Tris/HCl, 100 mM NaCl (pH 7.5) in a 0.5 cm cuvette at 25 °C. The curves were smoothed using the Savitzky-Golay method[54] and data were normalized to obtain the mean residue ellipticity as described in ref[55].

2.17 Biochemical synthesis of ProFAR

ProFAR was synthesized as described elsewhere,[56] starting from 5-phospho-D-ribosyl α-1-pyrophosphate and adenosine triphosphate in 50 mM NH_4COOCH_3 pH 7.8. The reaction progress was tracked spectrophotometrically and products were purified with ion-exchange chromatography using a POROS column (HQ 20, 10 mL, Applied Biosystems) and a linear gradient of NH_4COOCH_3 (50 mM / 1 M). ProFAR concentration was determined at 300 nm (ε_{300} = 6069 $M^{-1}cm^{-1}$)[57] and ProFAR purity was assessed from the absorbance ratio A_{290}/A_{260} for each fraction. Thereby, highly concentrated and > 90% pure

(A_{290}/A_{260} = 1.1–1.2 accounts for >95% purity)[58] fractions were lyophilized and stored at – 80 °C. ProFAR identity was confirmed using the HisF assay described below.

2.18 Activity measurements

The HisF activity was measured continuously following PrFAR turnover at 300 nm [ε_{300}(PrFAR-AICAR) = 5637 $M^{-1}cm^{-1}$].[59] Reaction conditions included 50 mM Tris acetate pH 8.5, 100 mM ammonium acetate, 1 µM HisA (to convert ProFAR to PrFAR) and 7 µM ProFAR in a total volume of 250 µL unless otherwise stated. ProFAR and HisA were synthesized as previously reported.[44]

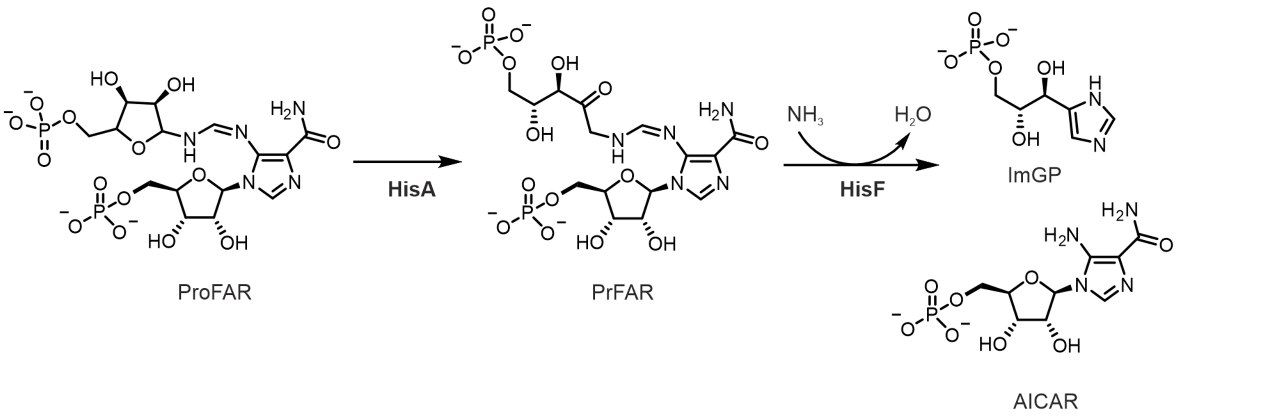

Scheme S8. Reaction assay for the determination of HisF activity. The metastable substrate PrFAR is in situ generated from the substrate analogue ProFAR using HisA. The turnover of PrFAR to ImGP and AICAR catalyzed by HisF can be monitored spectrophotometrically.

Measurements were performed in a 1 cm quartz cuvette at 25 C° The reaction was started with 50 nM HisF-wt, 33 nM K13AzoF, 50 nM K13NH$_2$AzoF, 100 nM K13MeAapF, 70 nM K13HAapF, 50 nM K13AatF, or 70 nM K13HtiF. The proteins were either used in the thermal equilibrium as isolated (TEQ$^{a.i.}$), after a certain PSS$^{\lambda}$ has been established or after back-isomerization to thermal equilibrium post irradiation (TEQ^{post}). To this end, irradiation with the appropriate wavelengths or incubation at 25 °C was conducted for a time period of at least ten times the $t_{1/2}$ of isomerization that was determined for each wavelength or thermally (**Table S11**) and was maintained over the whole measurement. To verify that the irradiation conditions are principally not harmful for the enzyme, HisF-wt was subjected to the same irradiation conditions as the variants. Measurements were performed in technical triplicates. The initial slopes were determined in a global linear fit using a concatenated linear regression including a direct weighting to consider the S.E., which is indicated as error bar. The slopes *m'* ± SE were then used to calculate the initial

velocities v/E_0 ± SE (s^{-1}) using the Lambert Beer Equation, followed by normalization with the enzyme concentration. The LRF reflects the ratio of activities between TEQ and $PSS^{\lambda 1}$ or $PSS^{\lambda 1}$ and $PSS^{\lambda 2}$. The respective LRF values were determined by selecting the data sets of both states and fitting them with a global fit analysis using a linear regression interaction model (Equation 7) and direct weighting,

$$\boldsymbol{v = m' \cdot D \cdot t}$$

Equation 7

with *A* = absorbance and *t* = time. The slope *m'* was shared in the global fit and the dummy constant *D* of the slower reaction was fixed to 1. The fitted *D* ± S.E. value of the faster reaction then corresponded to the LRF ± S.E. as defined by Equation 8.

$$\boldsymbol{LRF = \frac{v_1}{v_2}} \qquad v_1 > v_2$$

Equation 8

The p-value of the fit thereby indicates the significance of the LRF and the S.E..

References

[1] M. Bose, D. Groff, J. Xie, E. Brustad, P. G. Schultz, *J. Am. Chem. Soc.* **2006**, *128*, 388.

[2] a) C. Hoppmann, I. Maslennikov, S. Choe, L. Wang, *J. Am. Chem. Soc.* **2015**, *137*, 11218; b) C. Hoppmann, V. K. Lacey, G. V. Louie, J. Wei, J. P. Noel, L. Wang, *Angewandte Chemie (International ed. in English)* **2014**, *53*, 3932.

[3] A. A. John, C. P. Ramil, Y. Tian, G. Cheng, Q. Lin, *Organic letters* **2015**, *17*, 6258.

[4] J. Luo, S. Samanta, M. Convertino, N. V. Dokholyan, A. Deiters, *Chembiochem : a European journal of chemical biology* **2018**, *19*, 2178.

[5] Q. Xiong, T. Zheng, X. Shen, B. Li, J. Fu, X. Zhao, C. Wang, Z. Yu, *Chemical science* **2022**, *13*, 3571.

[6] T. Zheng, J. Fu, Q. Xiong, X. Shen, B. Li, X. Zhao, Z. Yu, *Chemical communications* **2023**, *59*, 1201.

[7] C. P. Janosko, O. Shade, T. M. Courtney, T. J. Horst, M. Liu, S. D. Khare, A. Deiters, *ACS omega* **2023**, *8*, 26590.

[8] D. S. Strugach, D. Hadar, M. Amiram, *ACS synthetic biology* **2023**, *12*, 2802.

[9] a) C. R. Crecca, A. E. Roitberg, *The Journal of Physical Chemistry A* **2006**, *110*, 8188; b) C. J. Brown, *Acta Crystallographica* **1966**, *21*, 146.

[10] a) H. M. D. Bandara, S. C. Burdette, *Chemical Society reviews* **2012**, *41*, 1809; b) A. A. Beharry, G. A. Woolley, *Chemical Society reviews* **2011**, *40*, 4422.

[11] V. Ladányi, P. Dvořák, J. Al Anshori, Ľ. Vetráková, J. Wirz, D. Heger, *Photochemical & photobiological sciences* **2017**, *16*, 1757.

[12] a) L. Gagliardi, G. Orlandi, F. Bernardi, A. Cembran, M. Garavelli, *Theoretical Chemistry Accounts* **2004**, *111*, 363; b) A. Mostad, C. Rømming, S. Hammarström, R. J. J. C. Lousberg, U. Weiss, *Acta Chemica Scandinavica* **1971**, *25*, 3561.

[13] C. L. Forber, E. C. Kelusky, N. J. Bunce, M. C. Zerner, *J. Am. Chem. Soc.* **1985**, *107*, 5884.

[14] Z. Ahmed, A. Siiskonen, M. Virkki, A. Priimagi, *Chemical communications* **2017**, *53*, 12520.

[15] K. Kuntze, J. Viljakka, E. Titov, Z. Ahmed, E. Kalenius, P. Saalfrank, A. Priimagi, *Photochemical & photobiological sciences* **2022**, *21*, 159.

[16] T. Dang, Z.-Y. Zhang, T. Li, *J. Am. Chem. Soc.* **2024**, *146*, 19609.

[17] J. Calbo, C. E. Weston, A. J. P. White, H. S. Rzepa, J. Contreras-García, M. J. Fuchter, *J. Am. Chem. Soc.* **2017**, *139*, 1261.

[18] a) D. Gupta, A. K. Gaur, H. Kumar, S. Singh, S. Venkataramani, *ChemPhotoChem* **2023**, *7*, e202300068; b) D. Gupta, A. K. Gaur, D. Chauhan, S. K. Thakur, V. Jeyapalan, S. Singh, G. Rajaraman, S. Venkataramani, *Inorganic Chemistry Frontiers* **2022**, *9*, 2315.

[19] a) L. M. Frija, A. J. Pombeiro, M. N. Kopylovich, *Coordination Chemistry Reviews* **2016**, *308*, 32; b) G. Mercuri, G. Giambastiani, A. Rossin, *Inorganics* **2019**, *7*, 144.

[20] W. Szymański, J. M. Beierle, H. A. V. Kistemaker, W. A. Velema, B. L. Feringa, *Chemical reviews* **2013**, *113*, 6114.

[21] T. Lougheed, V. Borisenko, T. Hennig, K. Ruck-Braun, G. A. Woolley, *Organic & biomolecular chemistry* **2004**, *2*, 2798.

[22] A. Nenov, T. Cordes, T. T. Herzog, W. Zinth, R. de Vivie-Riedle, *The Journal of Physical Chemistry A* **2010**, *114*, 13016.

[23] S. Wiedbrauk, H. Dube, *Tetrahedron Letters* **2015**, *56*, 4266.

[24] a) M. Hammarson, J. R. Nilsson, S. Li, T. Beke-Somfai, J. Andréasson, *The journal of physical chemistry. B* **2013**, *117*, 13561; b) S. Scarmagnani, Z. Walsh, C. Slater, N. Alhashimy, B. Paull, M. Macka, D. Diamond, *Journal of Materials Chemistry* **2008**, *18*, 5063.

[25] L. Kortekaas, W. R. Browne, *Chemical Society reviews* **2019**, *48*, 3406.

[26] a) R. Klajn, *Chemical Society reviews* **2014**, *43*, 148; b) L. Kortekaas, J. Chen, D. Jacquemin, W. R. Browne, *The journal of physical chemistry. B* **2018**, *122*, 6423.

[27] a) T. Cordes, T. Schadendorf, B. Priewisch, K. Rück-Braun, W. Zinth, *The Journal of Physical Chemistry A* **2008**, *112*, 581; b) T. Cordes, D. Weinrich, S. Kempa, K. Riesselmann, S. Herre, C. Hoppmann, K. Rück-Braun, W. Zinth, *Chemical Physics Letters* **2006**, *428*, 167; c) T. Cordes, B. Heinz, N. Regner, C. Hoppmann, T. E. Schrader, W. Summerer, K. Rück-Braun, W. Zinth, *Chemphyschem : a European journal of chemical physics and physical chemistry* **2007**, *8*, 1713.

[28] T. Baumann, M. Hauf, F. Richter, S. Albers, A. Möglich, Z. Ignatova, N. Budisa, *International journal of molecular sciences* **2019**, *20*, 2343.

[29] T. S. Young, I. Ahmad, J. A. Yin, P. G. Schultz, *Journal of molecular biology* **2010**, *395*, 361.

[30] B. Israeli, D. S. Strugach, S. Gelkop, S. Weber, D. S. Gozlan, M. Amiram, *Advanced Functional Materials* **2021**, *31*, 2011276.

[31] C. Hiefinger, S. Mandl, M. Wieland, A. Kneuttinger, *Methods in enzymology* **2023**, *682*, 247.
[32] M. Koh, H.-Y. Cho, C. Yu, S. Choi, K.-B. Lee, P. G. Schultz, *Bioconjugate chemistry* **2019**, *30*, 2102.
[33] S. J. Pike, A. Heliot, C. C. Seaton, *CrystEngComm* **2020**, *22*, 5040.
[34] F. Corzana, J. H. Busto, G. Jiménez-Osés, J. L. Asensio, J. Jiménez-Barbero, J. M. Peregrina, A. Avenoza, *J. Am. Chem. Soc.* **2006**, *128*, 14640.
[35] C. Hall, H. Wolfe, A. Wells, H.-C. Chien, C. Colas, A. Schlessinger, K. M. Giacomini, A. A. Thomas, *Bioorganic & medicinal chemistry letters* **2019**, *29*, 2254.
[36] H. Oubaha, N. Demitri, J. Rault-Berthelot, P. Dubois, O. Coulembier, D. Bonifazi, *The Journal of Organic Chemistry* **2019**, *84*, 9101.
[37] T. Ko, M. M. Oliveira, J. M. Alapin, J. Morstein, E. Klann, D. Trauner, *J. Am. Chem. Soc.* **2022**, *144*, 21494.
[38] K. A. Kalesh, L. P. Tan, K. Lu, L. Gao, J. Wang, S. Q. Yao, *Chemical communications* **2010**, *46*, 589.
[39] N. A. Braun, M. Ousmer, J. D. Bray, D. Bouchu, K. Peters, E. M. Peters, M. A. Ciufolini, *The Journal of organic chemistry* **2000**, *65*, 4397.
[40] J. E. Zweig, T. R. Newhouse, *J. Am. Chem. Soc.* **2017**, *139*, 10956.
[41] W. Zhao, Y. Li, S. Yang, Y. Chen, J. Zheng, C. Liu, Z. Qing, J. Li, R. Yang, *Analytical chemistry* **2016**, *88*, 4833.
[42] J. Nazet, E. Lang, R. Merkl, *PLOS ONE* **2021**, *16*, e0256691.
[43] A. Leaver-Fay, M. Tyka, S. M. Lewis, O. F. Lange, J. Thompson, R. Jacak, K. Kaufman, P. D. Renfrew, C. A. Smith, W. Sheffler et al., *Methods in enzymology* **2011**, *487*, 545.
[44] A. C. Kneuttinger, K. Straub, P. Bittner, N. A. Simeth, A. Bruckmann, F. Busch, C. Rajendran, E. Hupfeld, V. H. Wysocki, D. Horinek et al., *Cell chemical biology* **2019**, *26*, 1501-1514.e9.
[45] C. Engler, R. Kandzia, S. Marillonnet, *PloS one* **2008**, *3*, e3647.
[46] B. Rohweder, F. Semmelmann, C. Endres, R. Sterner, *BioTechniques* **2018**, *64*, 24.
[47] C. Hiefinger, G. Zinner, T. F. Fürtges, T. Narindoshvili, S. Schindler, A. Bruckmann, T. Rudack, F. M. Raushel, R. Sterner, *JACS Au* **2025**, *5*, 858.
[48] J. W. Chin, S. W. Santoro, A. B. Martin, D. S. King, L. Wang, P. G. Schultz, *J. Am. Chem. Soc.* **2002**, *124*, 9026.
[49] J. Jumper, R. Evans, A. Pritzel, T. Green, M. Figurnov, O. Ronneberger, K. Tunyasuvunakool, R. Bates, A. Žídek, A. Potapenko et al., *Nature* **2021**, *596*, 583.
[50] A. Hjorth Larsen, J. Jørgen Mortensen, J. Blomqvist, I. E. Castelli, R. Christensen, M. Dułak, J. Friis, M. N. Groves, B. Hammer, C. Hargus et al., *Journal of physics. Condensed matter* **2017**, *29*, 273002.
[51] D. Anstine, R. Zubatyuk, O. Isayev, *AIMNet2: A Neural Network Potential to Meet your Neutral, Charged, Organic, and Elemental-Organic Needs*, **2024**.
[52] S. Dasgupta, J. M. Herbert, *The journal of physical chemistry. B* **2020**, *124*, 1137.
[53] F. List, M. C. Vega, A. Razeto, M. C. Häger, R. Sterner, M. Wilmanns, *Chemistry & biology* **2012**, *19*, 1589.
[54] A. Savitzky, M. J. E. Golay, *Analytical chemistry* **1964**, *36*, 1627.
[55] S. M. Kelly, T. J. Jess, N. C. Price, *Biochimica et biophysica acta* **2005**, *1751*, 119.
[56] V. J. Davisson, I. L. Deras, S. E. Hamilton, L. L. Moore, *The Journal of Organic Chemistry* **1994**, *59*, 137.
[57] T. J. Klem, V. J. Davisson, *Biochemistry* **1993**, *32*, 5177.
[58] D. W. Smith, B. N. Ames, *Journal of Biological Chemistry* **1964**, *239*, 1848.
[59] S. Beismann-Driemeyer, R. Sterner, *The Journal of biological chemistry* **2001**, *276*, 20387.
